# Global patterns and a latitudinal gradient of flower disparity: perspectives from the angiosperm order Ericales

**DOI:** 10.1111/nph.17195

**Published:** 2021-03-04

**Authors:** Marion Chartier, Maria von Balthazar, Susanne Sontag, Stefan Löfstrand, Thomas Palme, Florian Jabbour, Hervé Sauquet, Jürg Schönenberger

**Affiliations:** ^1^ Department of Botany and Biodiversity Research University of Vienna Rennweg 14 Vienna 1030 Austria; ^2^ Institut de Systématique, Evolution, Biodiversité Muséum National d'Histoire Naturelle CNRS Sorbonne Université EPHE Université des Antilles 57 rue Cuvier, CP39 Paris 75005 France; ^3^ National Herbarium of New South Wales Royal Botanic Gardens and Domain Trust Mrs Macquaries Road Sydney NSW 2000 Australia; ^4^ Evolution and Ecology Research Centre School of Biological, Earth and Environmental Sciences University of New South Wales Kensington NSW 2033 Australia

**Keywords:** angiosperms, biodiversity, Ericales, flower morphology, latitudinal gradient, morphospace, disparity, diversity

## Abstract

- Morphological diversity (disparity) is an essential but often neglected aspect of biodiversity. Hence, it seems timely and promising to re‐emphasize morphology in modern evolutionary studies. Disparity is a good proxy for the diversity of functions and interactions with the environment of a group of taxa. In addition, geographical and ecological patterns of disparity are crucial to understand organismal evolution and to guide biodiversity conservation efforts.
- Here, we analyse floral disparity across latitudinal intervals, growth forms, climate types, types of habitats, and regions for a large and representative sample of the angiosperm order Ericales.
- We find a latitudinal gradient of floral disparity and a decoupling of disparity from species richness. Other factors investigated are intercorrelated, and we find the highest disparity for tropical trees growing in African and South American forests.
- Explanations for the latitudinal gradient of floral disparity may involve the release of abiotic constraints and the increase of biotic interactions towards tropical latitudes, allowing tropical lineages to explore a broader area of the floral morphospace. Our study confirms the relevance of biodiversity parameters other than species richness and is consistent with the importance of species interactions in the tropics, in particular with respect to angiosperm flowers and their pollinators.

## Introduction

Life on Earth is distributed unevenly due to varied geological and climatic conditions over time and space. In addition to these abiotic conditions, dynamics of speciation, extinction, migration, and biotic interactions likely play important roles in shaping species richness and species composition in different regions and communities (Gaston, 2000; Jablonski *et al*., 2017; Schluter & Pennell, 2017). Generally, species richness decreases with altitude, ocean depth, and latitude (Hillebrand, 2004; Vamosi & Vamosi, 2010; Kerkhoff *et al*., 2014; Brown, 2014; Tomašových *et al*., 2016; Jablonski *et al*., 2017). In particular, the origin and unevenness of the latitudinal gradient of species richness has generated extensive debates, and many potential explanatory factors have been proposed, including temperature, climate stability, biotic interactions, and available energy (e.g. mean summer temperature). As a general trend, all of these factors increase towards tropical latitudes (Pianka, 1966; Rohde, 1992; Mittelbach *et al*., 2007; Brown, 2014; Mannion *et al*., 2014; Belmaker & Jetz, 2015; Fine, 2015; Jablonski *et al*., 2017; Pontarp *et al*., 2018).

In addition to species number, biodiversity includes aspects such as phylogenetic diversity, morphological diversity, dominance and rarity of species, as well as the diversity of their ecosystem functions (Hillebrand *et al*., 2018; Stevens & Tello, 2018). On a global scale, our knowledge about these additional aspects is fragmentary at best (Gaston, 2000). In particular, we still have only a very limited understanding of the geographic and ecological distribution of functional and of morphological diversities (for plants, see e.g. Lupia, 1999; Swenson, 2012; Cornwell *et al*., 2014; Chartier *et al*., 2014, 2017; Zanne *et al*., 2014; Hillebrand *et al*., 2018; Mander, 2018; Weiser *et al*., 2018). Functional diversity summarizes traits predicting growth and survival rates (for plants: Swenson & Enquist, 2007; Swenson *et al*., 2012; Cornwell *et al*., 2014), whereas morphological diversity, also called disparity, is used to quantify and compare the variability of organisms belonging to a clade, or a group of taxa (Foote, 1999; Erwin, 2007; Minelli, 2015). Disparity is calculated from a multidimensional set of morphological traits and can be estimated by different indices such as, for example, the *range* (the largest difference between two taxa in a group), the *total variance* (the sum of variances of all characters), or the *mean character difference* (the average difference among taxa in a group; Wills *et al*., 1994; Foote, 1999; Ciampaglio *et al*., 2001; Erwin, 2007). The choice of disparity index depends on sample size, number and type of traits, and on the proportion of missing data in the morphological matrix (Ciampaglio *et al*., 2001). Furthermore, the interpretation of disparity patterns strongly depends on the phylogenetic and geographic scale investigated, and, importantly, on the biological functions of the traits that disparity estimates are based on.

For angiosperms, a central aspect of structural and functional diversity lies in the richness of biotic interactions and reproductive strategies, both of which are largely tied to their reproductive units (i.e. their flowers). Flowers produce and protect the gametes, they are the place for pollination and fertilization, and, finally, they produce fruits and seeds that disperse and propagate. Most angiosperms are pollinated by animals, and their sexual reproduction is thus tightly linked to plant–pollinator interactions. Changes in floral morphology, therefore, directly affect fitness and can also lead to speciation through reproductive isolation (Grant, 1994; Harder & Barrett, 2006; Baack *et al*., 2015; Reyes *et al*., 2015).

Floral disparity and its distribution have rarely been quantified (reviewed in Chartier *et al*., 2014). For the large, diverse, and globally distributed angiosperm order Ericales (Rose *et al*., 2018), we have previously shown that, with some exceptions, clade disparity generally increases with clade species richness. We also found that floral disparity was not correlated with clade crown age (Chartier *et al*., 2017). The two families accounting for most of the disparity in the order were Lecythidaceae (16% partial disparity; Brazil nut family) and Sapotaceae (14% partial disparity; shea tree family), corresponding to 3% and 9%, respectively, of the order’s species richness (Chartier *et al*., 2017). Plants in both tropical families typically grow as trees, the flowers of which are most probably pollinated by diverse types of animals (Kubitzki, 2004). It is thus likely that, in addition to species richness, patterns of floral disparity in the order are partly shaped by ecological and geographical factors. Here, we investigate whether there is a latitudinal gradient of floral disparity in Ericales. As already outlined herein, we might expect such a gradient because biotic interactions are more diverse, and species richness is higher in the tropics. In addition, we investigate and compare the variation of floral disparity among climate types, geographic regions, ecosystems (type of habitat), and life modes (plant growth form) to find other potential factors explaining global patterns of floral disparity in Ericales.

## Materials and Methods

All analyses were performed using the software R v.3.5.1 (R Core Team, 2018). Functions are referred to in the following format: *function name* {package name}. A more detailed version of these methods is available in Supporting Information Methods S1.

### Taxon sampling

We used the taxon sampling from Chartier *et al*. (2017). This data set describes 380 species belonging to 274 genera (79.5% of the 346 genera of Ericales), sampled across the 22 families of Ericales (Schönenberger *et al*., 2005; Rose *et al*., 2018). Our aim was to give the best representation possible of each taxonomic group, and of the morphological variation found in the whole order.

### Morphological matrix

To estimate morphological diversity (disparity), we used the morphological data set from Chartier *et al*. (2017). This data set consists of 36 morphological characters describing the anthetic flower for all species sampled. The data were scored using the database PROTEUS (Sauquet, 2019). The morphological matrix contains a total of 12 512 data entries (13.4% missing data) and is available in the online supplementary material of Chartier *et al*. (2017).

### Factor matrix

All sampled species were additionally coded for the four following factors: *growth form*, *habitat*, *climate*, and *region*. In this paper, we use abridged expressions such as ‘floral disparity of trees’, which should be understood as ‘floral disparity in species displaying an arborescent growth form’.

For each factor, the assignment of each species to one or more categories was made retrieving information from the literature cited in Chartier *et al*. (2017), and by crossing this information with the maps and descriptions from Cox (2001), Peel *et al*. (2007), and Loarie *et al*. (2009). This new data set is available as Dataset S1 and stored in the online database PROTEUS (Sauquet, 2019), with at least one bibliographic reference linked to each entry. It contains 1 800 new data entries (3.6% missing data).

We divided the factor *growth form* into the five categories occurring in Ericales (Kubitzki, 2004): (1) ‘trees’, (2) ‘shrubs’, (3) ‘lianas and climbers’, (4) ‘herbs and aquatic herbs’, and (5) ‘root parasites’.

We defined *habitat* factor categories by taking the biome descriptions from Loarie *et al*. (2009) and simplifying them into the three habitat states: (1) ‘forests’, (2) ‘open habitats’, and (3) ‘wet habitats’ (including mangroves and flooded forests/grassland/savannahs).

**Fig. 1:**
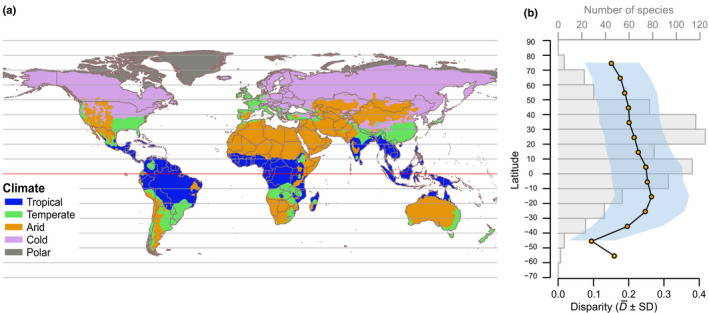
Climate categories and latitudinal gradient. (a) Köppen–Geiger climate classification simplified to five categories. Figure adapted from Peel *et al*. (2007). (b) Estimated latitudinal distribution (number of species) of 347 ericalean species (grey bars) and the corresponding floral disparity $\bar{D}$ (yellow dots; SD shown in light blue) per 10° longitudinal categories.

For the factor *climate* (Fig. 1a), we used the Köppen–Geiger climate classification based on temperature and precipitation, applying the five main categories described in Peel *et al*. (2007): (1) ‘tropical’, (2) ‘arid’, (3) ‘temperate’, (4) ‘cold’, and (5) ‘polar’ (see Methods S1, Section 1.2). Tropical high‐elevation species were coded as temperate.

Finally, we divided the factor *region* into (1) ‘North America’, (2) ‘Eurasia’, (3) ‘South America’, (4) ‘Africa’, (5) ‘Indo‐Pacific’, and (6) ‘Australia’. We followed the revised biogeographical delimitations of floral kingdoms as suggested by Cox (2001) for continent delimitations. Each species was assigned to its native region(s) only.

### Disparity

We computed floral disparity for the different factor categories of taxa (e.g. ‘trees’ from factor *growth form*) using the morphological matrix. From this matrix, we first created a distance matrix by calculating a dissimilarity index for each pair of taxa: the *mean character difference* (D), following Sneath & Sokal (1973) and Foote (1999). D is a version of the Gower index, suited for data sets like ours that contain at the same time continuous, categorical ordered, categorical unordered, and binary data. The detailed calculation of D is given in Chartier *et al*. (2017). Disparity $\bar{D}$ was then estimated for a category as the *mean pairwise dissimilarity* $\bar{D}$ among all taxa from that category (Foote, 1999) by averaging distances in the distance matrix for all taxa belonging to that category. The mean pairwise dissimilarity is less sensitive to large differences in group sizes than other disparity estimations are, such as the range (Ciampaglio *et al*., 2001); this makes it well suited to our data.

There are two types of polymorphism in our data: polymorphism in the morphological matrix (2.2%), and polymorphism in the factor matrix (16.5%). First, as our calculation method of $\bar{D}$ cannot take polymorphism into account in the morphological data set, and since the percentage of polymorphism in this matrix is very low, a morphological matrix without polymorphism was randomly sampled and the distance matrix was recomputed prior to each computation of $\bar{D}$ and each test (see later). This did not impact our results (data not shown). Second, some species belong to several factor categories (e.g. some species grow in ‘temperate’ as well as in ‘cold’ areas). When computing disparity for these categories, such species were included in each of these categories (but not when performing tests; see later).

For each factor, we compared $\bar{D}$ among factor categories (e.g. among all *growth form* categories) with one‐way permutation ANOVAs on the morphological distance matrix. This analysis consists of comparing the *F*‐ratio of the data set to the distribution of the *F*‐ratio calculated for 9999 permutations of the data set. For each permutation, a random morphology (i.e. a row in the matrix) is assigned to each species without replacement. For the *F*‐ratio formula, see Hawkins (2014, p. 167). In our case, a permutation test is preferable to an ANOVA or a Kruskal–Wallis test because we compare pairwise distances whereby each species contributes to multiple distance values, creating a lack of independence among values and inflating the degrees of freedom. As *post hoc* tests, we made pairwise comparisons of $\bar{D}$ among factor categories with permutation tests on central tendencies following Bonnini *et al*. (2014). For a pair of categories, this test consists of calculating the difference (here noted *T*) between the average *D* of each of the two categories and comparing this difference with the distribution of *T* calculated for 9999 permutations of the data set without replacement (as described earlier). We applied a Bonferroni correction for multiple comparisons to these *post hoc* tests. To deal with polymorphism in the factor matrix (the grouping variables of these tests), a factor matrix without polymorphism was randomly sampled and each permutation ANOVA and corresponding *post hoc* tests performed 100 times; *P*‐values and statistics for each test are thus presented as an average ± SD over these 100 calculations. To save execution time, calculations were run in parallel on multiple computer cores using packages foreach (Microsoft Corporation & Weston, 2020), parallel (R Core Team, 2018) and doparallel (Microsoft Corporation & Weston, 2019). Finally, for these tests, we excluded the category ‘root parasites’ (*n* = 2) from factor *growth form*. Scripts are available upon request from M. Chartier.

### Associations among factor categories

We performed a series of chi‐squared tests to investigate associations among categories belonging to different factors and detect ecological/biogeographic trends in Ericales (e.g. Do arborescent species more often grow in tropical areas? That is, is there an association between categories ‘trees’ from factor *growth form*, and ‘tropical’ from factor *climate*?).

To meet the chi‐squared test criteria, and for these analyses only, we merged the categories ‘polar’ (*n* = 7 species) and ‘cold’ (*n* = 56) from factor *climate* and we excluded the category ‘root parasites’ (*n* = 2) from factor *growth form*. Associations were not tested among categories belonging to the same factors.

We performed the chi‐squared tests using *chisq.test* {stats}. For significant tests (*P* < 0.05), the strength of the association was estimated from the Pearson residuals (PR; Hawkins, 2014). Multiple correlations are usually visualized using a correlation table (Methods S1, Section 1.3). To visualize multiple correlations more easily and detect clusters of associated factor categories, we plotted these categories with a nonmetric multidimensional scaling applied to a distance matrix computed from the PR values among categories, using *metaMDS* {vegan} with the Bray–Curtis distance (Anderson, 2001; Oksanen *et al*., 2017; Methods S1, Section 1.3). This allowed us to draw an association network in which significantly positively associated categories fall close to each other and are linked by red lines, whereas significantly negatively associated categories fall far from each other and are linked by blue lines (Fig. 3). Intercorrelated categories appear linked together on that graph (clusters).

### Variation of disparity accounting for associations among factor categories

Some factor categories are significantly associated with one another (see the Results section). Comparing disparity among categories for each factor independently might thus lead to ambiguous interpretations about the link between these factors and variation in disparity.

We solved this issue by first keeping one factor constant while looking at the variation of disparity for the others (e.g. whether there was a difference among the *climate* categories if we looked at ‘trees’ only). This was, however, only possible in a few cases that we report in the text, since category sizes become small once split and once polymorphism is sampled; all trends are shown in Notes S1, Section 2.3. Second, we furthermore calculated disparity for the two clusters of associated factor categories identified from the association network representation (Fig. 3, see later). $\bar{D}$ was in that case calculated for a given cluster by including all species belonging to the intersection of each factor and, within factors, the union of each category. For example, if a cluster is composed of the categories ‘tropical’ and ‘temperate' (from factor *climate*), and ‘shrubs’ (from factor *growth form*), all 'tropical shrubs' and 'temperate shrubs' were included in the calculation of $\bar{D}$ for that cluster. This is a strict representation of these clusters, as more species might present some of but not all the characteristics of each cluster. We compared disparity between the two clusters with a permutation test on central tendencies as described earlier herein, with 99 999 permutations without replacement.

### Latitudinal distribution of species and disparity

We estimated the latitudinal distribution of the species from the data set by extracting location records (latitude and longitude) from the Global Biodiversity Information Facility online database (https://www.gbif.org/) using *occ_search* {rgbif} (Chamberlain, 2017; Methods S1, Section 1.4). Distribution maps (Notes S2, Section 3) were then plotted using the package maptools (Bivand & Lewin‐Koh, 2017) and manually checked for atypical and nonnative records by using data from the literature and online trustworthy websites (such as the International Union for Conservation of Nature website, http://www.iucnredlist.org/). This allowed us to estimate the presence/absence of 347 (91%) of the study species in each 10° latitude interval across the globe (see also Notes S1, Section 2.5).

Disparity ($\bar{D}$) was then calculated for the species occurring in each given latitude interval. Finally, we tested for the correlations between latitude and species richness, latitude and disparity, and species richness and disparity (for each latitudinal interval) with Pearson correlation tests using *cor.test* {stats} and *lm* {stats}. For these tests, latitude values were treated as absolute values, to represent distances (north or south) from the Equator.

An additional permutation test was performed to show that the observed latitudinal variation in disparity was not due to the latitudinal variation in species number (Notes S1, Section 2.4).

## Results

### Variation of floral disparity

**Fig. 2:**
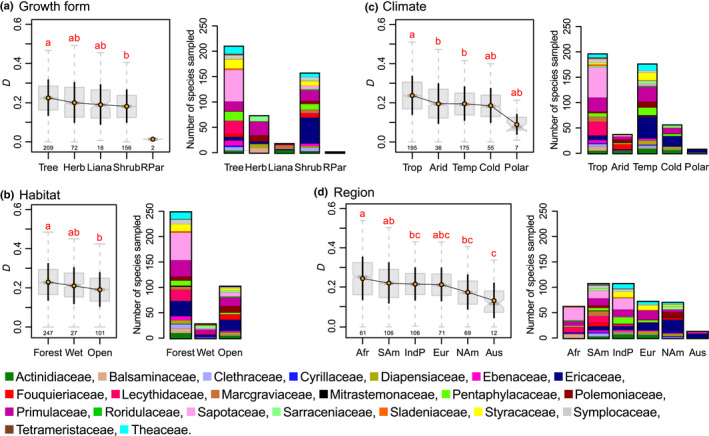
Overall variation of floral disparity among categories of (a) *growth form* (b) *climate* (c) *habitat*, and (d) *region* in Ericales. *D* is the mean pairwise difference. For each boxplot, sample size is given below each box, and disparity $\bar{D}\pm \text{SD}$ is indicated by orange dots and black error bars. *Post hoc* test results are depicted by red letters; categories that are significantly different are labelled with a different letter. The coloured bar‐plots indicate the number of species sampled per family (according to Angiosperm Phylogeny Group IV; Stevens, 2017) in each factor category. For (a), growth form, the category ‘root parasites’ was not included in the statistical analyses as it contains only two species. Abbreviations: a. Growth form: Herb, herbs and aquatic herbs; Liana, lianas and climbers; RPar, root parasitic; b. Climate: Trop, tropical; Temp, temperate; c. Habitat: Wet, wet habitat; Open, open habitat; d. Region: NAm, North America; Eur, Eurasia; SAm, South America; Afr, Africa; IndP, Indo‐Pacific; Aus, Australia.

Floral disparity differed significantly among categories of growth forms (excluding the two root parasitic species from the analysis; permutation ANOVA (±SD): *F* = 485.43 ± 92.59, *P* = 7.0e^−5^ ± 1.43e^−4^), habitat types (*F* = 366.00 ± 75.26, *P* = 9.55e^−4^ ±  1.50e^−3^), climate types (*F* = 382.51 ± 67.98, *P* = 7.0e^−5^ ± 1.26e^−4^), and regions (*F* = 101.43 ± 67.98, *P* = 0.017 ± 0.008). *Post hoc* tests are summarized by red letters in Fig. 2 and the main trends of variation are described in the following.

#### Growth form

Overall (i.e. when including the entire data set in the analysis), disparity (± SD) decreased slightly from ‘trees’ ($\bar{D}=0.225\pm 0.090$) to ‘herbs and aquatic herbs’ ($\bar{D}=0.201\pm 0.101$), ‘lianas and climbers’ ($\bar{D}=0.189\pm 0.101$), and ‘shrubs’ ($\bar{D}=0.182\pm 0.082$; Fig. 2a). The two root parasitic species sampled (*Mitrastemon matudae* and *Mitrastemon yamamotoi*) were not included in the analyses and only differed from each other in their number of carpels. To get around potential correlations among factors (e.g. *growth form* and *climate*), we compared the disparity of growth forms within each category of the other factors. For example, we investigated whether, when looking at ‘tropical’ species only, trees were still showing more disparity than the other growth form categories. We did so for each category of the factors *climate*, *habitat* and *region* (Notes S1, Section 2.3). The general pattern of disparity variation among growth forms was recovered within categories ‘forests’ (from factor *habitat*) and ‘South America’ (from factor *region*). Although not significantly, the tendency for ‘trees’ to display the highest disparity was retrieved within all factor categories (Notes S1, Section 2.3).

#### Habitat

Overall, floral disparity was highest in ‘forests’ ($\bar{D}=0.231\pm 0.093$), intermediate in ‘wet habitats’ ($\bar{D}=0.211\pm 0.091$), and lowest in ‘open habitats’ ($\bar{D}=0.192\pm 0.085$; Fig. 2b). This result was only recovered within category ‘South America’ (from factor *region*; Notes S1, Section 2.3).

#### Climate

Overall, ‘tropical’ species ($\bar{D}=0.238\pm 0.097$) displayed the highest level of floral disparity, followed by species distributed in ‘arid’ ($\bar{D}=0.196\pm 0.101$) and ‘temperate’ ($\bar{D}=0.196\pm 0.085$) areas (Fig. 2c). Disparity in ‘cold’ ($\bar{D}=0.186\pm 0.086$) and ‘polar’ areas ($\bar{D}=0.090\pm 0.050$) did not significantly differ from the other categories. The decrease of floral disparity from tropical to temperate climate categories held within categories ‘trees’ (from factor *growth form*) and ‘Africa’ (from factor *region*) and was only a tendency for category ‘forests’ (factor *habitat*; Notes S1, Section 2.3). Within category ‘South America’ (factor *region*), floral disparity was higher for tropical species than for arid species. Although not significantly, the tendency for disparity to decrease from tropical, to temperate, to cold and polar climate categories was retrieved within all factor categories but one (Notes S1, Section 2.3).

#### Region

Overall, disparity was highest for ‘Africa’ species ($\bar{D}=0.245\pm 0.107$). ‘South America’ ($\bar{D}=0.222\pm 0.101$), ‘Indo‐Pacific’ ($\bar{D}=0.217\pm 0.081$), and ‘Eurasia’ ($\bar{D}=0.215\pm 0.083$) species displayed similar lower levels of disparity. ‘North America’ ($\bar{D}=0.176\pm 0.086$) and ‘Australia’ ($\bar{D}=0.134\pm 0.087$) species displayed the lowest levels of disparity (Fig. 2d). This trend was only recovered for the category ‘forests’ of factor *habitat* (Notes S1, Section 2.3).

### Variation of floral disparity when combining factor categories

**Fig. 3:**
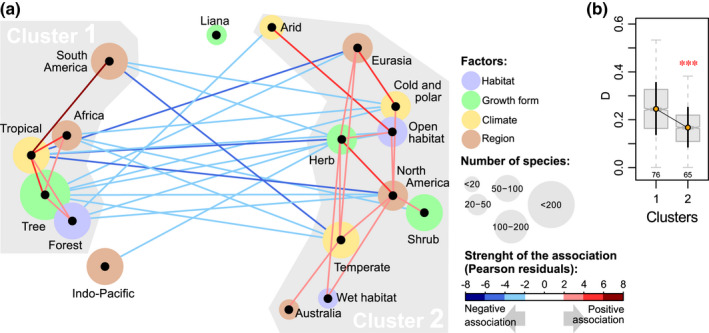
(a) Association network and (b) disparity for two clusters of associated categories in Ericales. The graph in (a) is used to visualize the results of chi‐squared tests assessing the multiple associations among factor categories in our data set. Factor categories that are significantly associated are linked by a line whose colour represents the strength and direction of the association (interpreted from the values of Pearson residuals). This representation is equivalent to a classical correlation table (Supporting Information Methods S1, Section 1.3). Our results show that some categories are associated with each other and form two distinct groups that we call cluster 1 and cluster 2. The disparity of these clusters is given in (b). *D* is the mean character differences between two taxa; sample size is given below each box, and disparity $\bar{D}\pm \text{SD}$ is indicated by orange dots and black error bars; ***, significant difference.

We found significant associations among categories for each pair of factors (Table 1). *Post hoc* test details are illustrated in Fig. 3a.

**Table 1:** Chi‐squared tests for the association among the categories of factors *growth form*, *habitat*, *climate type*, and *region*.

Comparison	*χ ^2^*	df	*P*‐value
*Growth form*–*climate type*	80.99	9	1.03e^−13^
*Growth form*–*region*	100.12	15	1.24e^−14^
*Growth form*–*habitat*	49.34	6	6.39e^−9^
*Climate type*–*region*	263.29	15	< 2.20e^−16^
*Climate type*–*habitat*	64.13	6	6.49e^−12^
*Region*–*habitat*	47.52	10	7.59e^−7^

Our data show two clusters of associated categories that describe two large groups of species sharing particular ecological/biogeographic trends in Ericales (Fig. 3a). Cluster 1 corresponds to species belonging to the categories ‘forests’ (factor *habitat*), ‘tropical’ (factor *climate*), ‘trees’ (factor *growth form*), and ‘Africa’ or ‘South America’ (factor *region*). Cluster 2 corresponds to species belonging to the categories ‘open habitat’ or ‘wet habitat’ (factor *habitat*), ‘temperate’, ‘arid’, or ‘cold’ and ‘polar’ (factor *climate*), ‘herbs and aquatic herbs’ or ‘shrubs’ (factor *growth form*), and ‘North America’ or ‘Eurasia’ (factor *region*). Species strictly representing cluster 1 (*n* = 76) showed significantly higher (26%) floral disparity ($\bar{D}=0.247\pm 0.108$) than those (*n* = 65) representing cluster 2 ($\bar{D}=0.169\pm 0.082$; permutation test on central tendency: *T* = 0.0782, *P* = 0; Fig. 3b). Note that there was no significant association in our data set for categories ‘lianas and climbers’ and ‘Indo‐Pacific’ to any other category.

### Latitudinal distribution of species richness and disparity

**Fig. 4:**
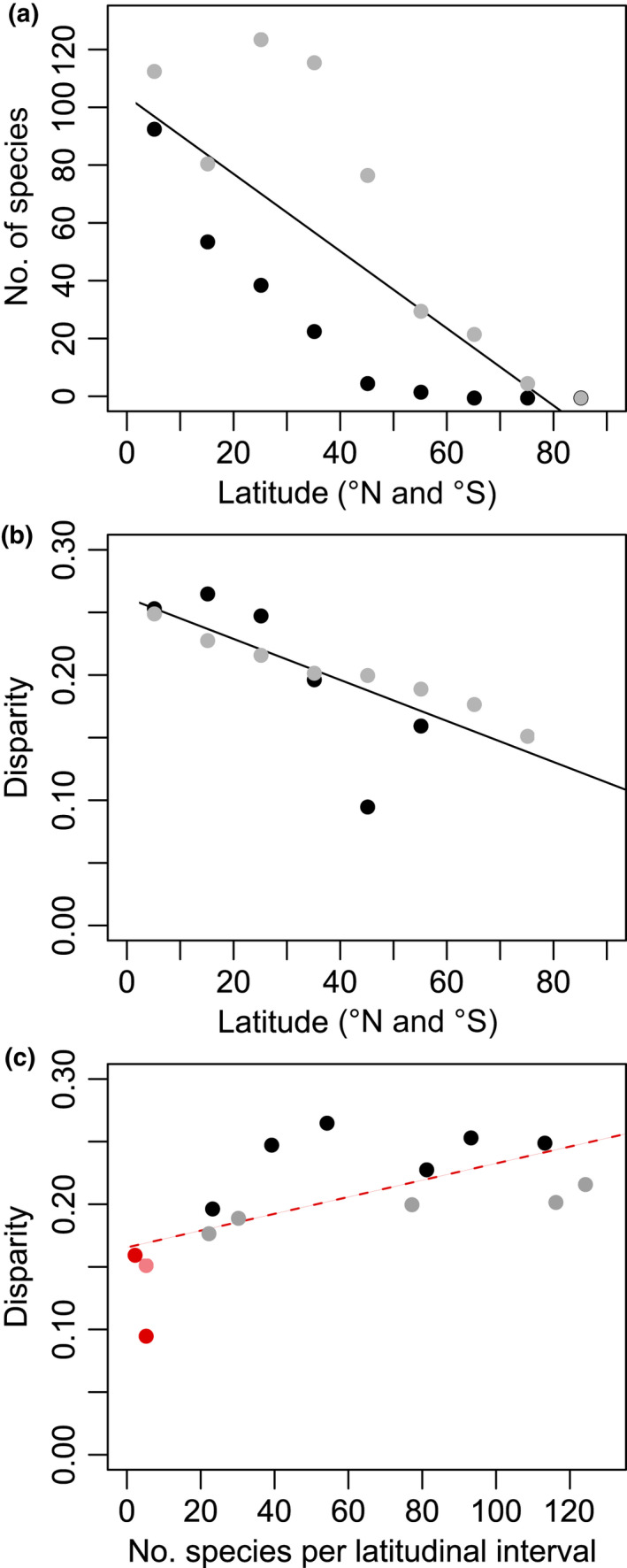
Relationships among number of species per latitudinal interval, floral disparity, and latitude (distance to equator) for 347 species of Ericales. (a) Number of species vs latitude; (b) disparity vs latitude; (c) disparity vs number of species. Black lines indicate significant correlation; red dashed line indicates correlation only significant when the three latitudinal intervals containing five species or less (red dots) are included. Absolute values were used for latitude, to pool data from the Northern Hemisphere (grey/light red dots) and the Southern Hemisphere (black/red dots).

The estimated species richness and floral disparity both significantly decreased towards the poles (Figs 1b, 4a,b). Species richness peaked in the subtropical area of the Northern Hemisphere and near the Equator, between latitudes 40° and 20° (113 species), and between latitudes 0° and 10° (124 species; Fig. 1b), and steeply decreased with latitude (*r* = −0.78, *P* < 10^−5^; linear regression: intercept 103.7, slope coefficient −1.336; Fig. 4a). On the other hand, disparity peaked in the Southern Hemisphere, between latitudes −10° and −20° ($\bar{D}=0.266\pm 0.105$; Fig. 1b). It decreased with latitude (*r* = −0.77, *P* = 0.001; linear regression: intercept 0.26, slope coefficient −0.002), with a weak decrease towards the North Pole and a steeper decrease towards the South Pole (Figs 1b, 4b). This correlation held when removing the three latitudinal intervals containing five species or less (*r* = −0.90, *P* < 10^−3^).

There was no clear correlation between disparity and species richness. A weak positive correlation is due to three latitudinal intervals each containing only five species or less (*r* = 0.63, *P* = 0.015), and this correlation disappears when these intervals are not included (*r* = 0.33, *P* = 0.329; Fig. 4c). The permutation test we performed also showed that the increase of disparity near the Equator was not due to the higher number of species present at these latitudes (Notes S1, Section 2.4).

## Discussion

Our results indicate that, in the order Ericales, floral disparity is significantly higher in the tropics than in other climate zones. Both floral disparity (morphological diversity) and species richness increase with lower latitudes. However, floral disparity is highest in southern tropical seasonal forests, whereas species richness is higher in northern tropical and subtropical latitudes (Fig. 1). In a previous study, we used the same morphological data set to investigate changes in disparity across floral modules and among ericalean lineages (Chartier *et al*., 2017). We showed that flower morphology differed among ericalean clades, that these clades filled the morphospace in a mosaic pattern, and that clade floral disparity increased with clade size, albeit with notable exceptions; for example, Balsaminaceae (touch‐me‐not family) and Sapotaceae (shea tree family). Disparity was not correlated to clade crown age, and there was no phylogenetic pattern of distribution of disparity among families, suggesting that there are other factors that drive variations in floral disparity in the Ericales (Chartier *et al*., 2017). The present analyses show that different categories of *growth form*, *region*, *climate type* and *habitat* show slightly different levels of disparity (Fig. 2). These factor categories are intercorrelated, which renders their respective effects on disparity variations difficult to separate at this taxonomic scale and given the structure of the order Ericales (see subsequent discussion). Nevertheless, there is a strong signal that, in Ericales, tropical trees growing in forests of Africa and South America (among them the speciose and very diverse family Lecythidaceae) show higher floral disparity than other Ericalean representatives do (Fig. 3).

Contrary to species richness, disparity is a complex and subjective measure of biodiversity, as it can be estimated from many different combinations of traits. As a consequence, trends in disparity variation will not always reflect the same evolutionary or biogeographic processes and will strongly depend on the ecological or physiological function of the measured traits. For example, no latitudinal gradient was found for the disparity of moth wing ornamentation in the New World, because this trait is under strong selective pressure to match resting backgrounds and avoid predators at all latitudes (Ricklefs, 2009). For plants, there is also no latitudinal gradient in pollen ornamentation disparity (Mander, 2018); currently, it is still unclear which of the measured morphological pollen traits are adaptive and thus whether variation in these traits is driven by chance, taxonomy, or reflects evolutionary processes (Lupia, 1999; Mander, 2016, 2018). By contrast, it has been found that tree functional diversity is higher at low latitude and in tropical seasonal forests across North and South America (Swenson & Enquist, 2007; Swenson *et al*., 2012; but see Lamanna *et al*., 2014). The measured traits predict plant growth and survival rates, and thus reflect the demographic dynamics of plant communities. These results indicate that an increase in functional diversity may be promoted in regions where abiotic selective constraints are weaker, and where biotic interaction rates and niche partitioning are more important, triggering morphological differentiation (Swenson & Enquist, 2007; Swenson *et al*., 2012).

Our analysis of floral trait points towards a role of climate as well as latitude in floral disparity patterns, probably linked to biotic interactions. For example, the most variable traits in Ericales flowers are petal union and stamen types, both linked to functional aspects of pollination biology (Chartier *et al*., 2017). Biotic interactions directly impacting floral evolution are mainly due to pollinators. About 88% of angiosperms are pollinated by animals, and this proportion has been estimated to be as high as 99% at tropical latitudes (Regal, 1982; Bawa, 1990; Ollerton *et al*., 2011). Several studies investigating plant clades and pollination networks have also brought forward evidence for an increase in plant–pollinator interaction dynamics in the tropics. For example, it has been shown that the number of different pollination systems increases towards the tropics, probably because tropical areas contain a broader diversity of functional groups of pollinators, including taxa such as bats, birds, or primates (Ollerton *et al*., 2006; but see Schleuning *et al*., 2012). In addition, it has been shown that interactions with pollinators are more specialized in the tropics (Trojelsgaard & Oleson, 2013). These combined factors explain possible selection for a higher number of floral traits adapted to specific pollinators in the tropics. Among the many different pollination systems that have been described in Ericales, some are found across all latitudes (e.g. pollen/nectar collecting bees, flies), but many are indeed unique to the tropics (bats, Euglossini bees, squirrels/flying squirrels), or at least more diverse in the tropics (moths, birds, hummingbirds, mammals; Sazima *et al*., 1993; Endress, 1996; Yumoto *et al*., 2000; Kubitzki, 2004). Exceptions to this pattern might occur in biodiversity hotspots (South Africa, the Mediterranean area), although we do not observe a particular peak in floral disparity for the corresponding latitudes in our data set (Fig. 1). Note that wind pollination is of lesser importance in Ericales (it is found, for example, in Ericaceae (heath family) in the genus *Erica* and the tribe Empetreae, and in Actinidiaceae (kiwifruit tree family) in the genus *Actinidia*; Kubitzki, 2004).

The general increase in disparity towards tropical latitudes that we observe in our data may be partly due to the diverse pollination systems in the largely tropical families Lecythidaceae (bees, bats, beetles; Kubitzki, 2004), Sapotaceae (insects, bats, squirrels/flying squirrels; see later), Primulaceae p.p. (oil bees; Buchmann, 1987). In contrast to this, it has also been shown elsewhere that species‐rich tropical lineages (or assemblages) can show very little floral variation if all or most of their species are pollinated by animals from the same functional pollinator group. This is for instance the case in the tropical trees from the large genus *Myrcia* (Myrtaceae; Vasconcelos *et al.*, 2019) bearing morphologically homogeneous, inconspicuous, and unspecialized flowers pollinated by bees. Delmas *et al.* (2020) also showed that tropical and temperate/subtropical assemblages of woody species in Australia mostly produce small whitish generalist flowers, probably pollinated by insects including thrips, flies, and small beetles. In our data set, Sapotaceae also mostly bear small white flowers (Kubitzki, 2004), but this is one of the most variable families in the order when looking at other floral traits than colour (Chartier *et al*., 2017). As far as known, this family is pollinated by bats (Cleghorn, 1922; van der Pijl, 1936; Nathan *et al*., 2009), squirrels and flying squirrels (Yumoto *et al*., 2000), and insects (Basga *et al*., 2018) and shows high variation in, for example, petal and petal whorl numbers, stamen and stamen whorl numbers, and types of staminodes.

The presence of generalist systems in the tropics, leading to the evolution of lineages bearing homogeneous (e.g. small and white) flowers is not incompatible with an overall pollination‐driven increase of floral disparity in the tropics. In our data set, the morphospace area occupied by cold/temperate species and the area occupied by tropical species largely overlap, the area occupied by tropical species being larger (Notes S1, Section 2.2). The morphological diversity of tropical ericalean flowers encompasses the diversity of the order as a whole and exceeds that of nontropical species. This is in agreement with the general observation that floral diversity is broadest in the tropics (e.g. Endress, 1996). It also implies that there are no specific floral morphologies related to cold/temperate zones, pointing towards the absence of large‐scale patterns of morphological convergence. Our data, rather, indicate a release of constraints in the tropics, expressed in the occupation of large areas of the floral morphospace by certain phylogenetic lineages. In particular, two tropical families increase the total area of the ericalean floral morphospace: Lecythidaceae, a medium‐sized family presenting the highest floral disparity in Ericales; and Sapotaceae, a very speciose homogeneous group, but whose unique combinations of floral features place the family in the periphery of the morphospace (Chartier *et al*., 2017).

The patterns of disparity variation that we observed at the order level were not significant or could not be properly tested within families or within factor categories with our sampling effort as some factor categories are distributed unevenly across the order. For example, for some factors, only one category is represented in a given family (e.g. all Sapotaceae and Lecythidaceae are tropical trees, all Marcgraviaceae are distributed in South America). In addition, some families are too small to observe any pattern even if sampled completely (seven families contain fewer than 12 species, e.g. Tetrameristaceae, Roridulaceae and Fouquieriaceae). Nevertheless, even if in many cases ericalean families are limited to a narrow range of strategies (with regard to the factors we investigated here), we observe broad trends in biodiversity variation that emerge from these patterns at a larger phylogenetic scale. This has also been shown for the latitudinal gradient in vascular plant species number (Wieser et al., 2018). Even though we cannot test for the effect of phylogenetic relationships on disparity based on the present data because several deeper nodes of Ericales are presently unresolved or unsupported (Schönenberger *et al*., 2005; Rose *et al*., 2018), exploring such effects could be approached in the future by focusing on well‐supported subclades (e.g. the ericoids or the primuloids; Rose *et al*., 2018) or on individual families.

The drawback of working at large taxonomic scales is, unfortunately, the present lack of ecological information (particularly about pollination) that could help us to understand the mechanisms leading to these broad‐scale patterns. Our data on Ericales, however, suggest that a well‐suited clade for studying these mechanisms at a finer scale would be Primulaceae (primrose family), because of its large size (2788 species) and high morphological variability. Primulaceae represent 22.1% of Ericales species, and contribute 14% to the order's floral disparity (Chartier *et al*., 2017). In addition, the family presents sufficient variation in climate types, growth form, habitat, and is widely distributed (Dataset S1). However, even at this scale, the lack of ecological data for most species would remain a limiting factor for these analyses.

In our data, the species displaying the highest degree of floral disparity are those with an arborescent form distributed in the African and South American tropical forests (Fig. 3). There is, however, no apparent link between growth form and floral disparity, and the slightly higher floral disparity of trees may be an artefact due to the strong association between the states ‘trees’ and ‘tropical’ (Fig. 3). Indeed, nearly 60% of the tree species included in our data set grow in tropical forests, and within the tropics > 60% of Ericales species are trees. This makes it difficult to disentangle the effects of growth type and climate type on the variation of floral disparity. In addition, since there is currently no reliable phylogeny that includes all the species sampled (suprafamilial relationships are unsupported; Schönenberger *et al*., 2005; Rose *et al*., 2018), we did not correct for a phylogenetic effect when studying the relationships between factors and disparity. There is no distinct pattern of disparity variation in Ericales; for example, early diverging lineages do not seem to show more or less disparity than the rest of the order. Nevertheless, disparity varies greatly among ericalean families (Chartier *et al*., 2017). The high disparity found for trees could, for example, be due to the contribution of the family Lecythidaceae. When Lecythidaceae are pruned from the data set, our main results do not change but lose statistical significance as the category ‘tropical’ climate then only tends to show the highest disparity. In these adapted analyses, there is also no more trend for any *growth form* to show different levels of morphological disparity. The effect of climate on floral disparity thus appears to be more robust than the effect of growth form. Further evidence for the importance of the effect of climate lies in the decrease in disparity from tropical to temperate climate categories. Categories cold and polar do not display significantly different disparity from any other climate category in our dataset (although they clearly tend to show lower disparity), most likely because these categories are very often associated to category ‘temperate’ in the data (polymorphism).

In angiosperms, the flower is the structure dedicated to sexual reproduction, and a shift in floral features can provoke reproductive isolation by different mechanisms (Waser & Ollerton, 2006). We might thus expect floral disparity to be correlated with species number in a clade or a region. Our data show that this is not always the case (Fig. 4c; Notes S1, Section 2.4; Chartier *et al*., 2017). For example, we find that African species tend to display the highest level of floral disparity, although species number is significantly higher for the South American and Indo‐Pacific regions in our data set. The decoupling of disparity and species number in a clade, a region, or through time is quite common (Roy & Foote, 1997; Lupia, 1999; Eble, 2000; Roy *et al*., 2001; Neige, 2003; Oyston *et al*., 2015).

Clearly, disparity is an important component of biodiversity and is worth being considered in any attempt to measure biodiversity. When calculated on floral traits, disparity may also provide useful approximations for the diversity of ecological relationships (e.g. plant–pollinator interactions) and might help understand evolutionary patterns (e.g. pollination‐mediated selection in a biogeographic context). Floral disparity in a given geographical area might also be a particularly useful parameter for assessing the conservation value of the area, as disparity not only reflects an important part of the local plant community, but as it isalso a possible proxy for a community’s ecological dynamics via plant–pollinator interactions.

## Author contributions

MC, MvB and JS designed the work, SS, MvB, MC, SL, FJ and JS generated the data, HS designed the online database (PROTEUS) used to record and store the data, and MC analysed the data with help from TP. MC, MvB and JS wrote the paper with significant contributions from the other authors. All authors gave final approval for publication.

## Supporting information


**Dataset S1** Factor matrix with corresponding legend, raw data and references.

**Methods S1** Supplementary methods of the study (SI sections 1.1 to 1.5).
**Notes S1** Supplementary results of the study (SI sections 2.1 to 2.5).
**Notes S2** Distribution maps of the study species (SI section 3).
Please note: Wiley Blackwell are not responsible for the content or functionality of any Supporting Information supplied by the authors. Any queries (other than missing material) should be directed to the *New Phytologist* Central Office.

## Data Availability

The data set supporting this article is given in Supporting Information (Dataset S1). The data are also stored in the online database PROTEUS (http://eflower.myspecies.info/; Sauquet, 2019). Correspondence and material requests should be addressed to MC and JS.
