# Supplementary material for: Global patterns and a latitudinal gradient of flower disparity: perspectives from the angiosperm order Ericales
Source: New Phytol. 2021 Mar 4;230(2):821–31. doi: 10.1111/nph.17195 (PMC8048689; doi:10.1111/nph.17195)
Supplement: Supplementary file 2 — Methods S1 Supplementary methods of the study (SI sections 1.1 to 1.5). Notes S1 Supplementary results of the study (SI sections 2.1 to 2.5). Notes S2 Distribution maps of the study species (SI section 3). Please note: Wiley Blackwell are not responsible for the content or functionality of any Supporting Information supplied by the authors. Any queries (other than missing material) should be directed to the New Phytologist Central Office. [file NPH-230-821-s002.pdf]

New Phytologist - Supplementary Information

Global patterns and a latitudinal gradient of flower disparity: perspectives from the angiosperm order Ericales.

Marion CHARTIER, Maria VON BALTHAZAR, Susanne SONTAG, Stefan LÖFSTRAND, Thomas PALME, Florian JABBOUR, Hervé SAUQUET, Jürg SCHÖNENBERGER.

Article acceptance date: 13 December 2020

SUPPLEMENTARY INFORMATION

1. Methods S1 2
1.1. Taxon sampling and scoring of the morphological dataset 2
1.2. Growth form, habitat, climate, and geographic region 2
1.3. Creation of the association network. 3
1.4. Latitudinal distribution of species richness and disparity 4
1.5. Potential methodological shortcomings 4
2. Note S1 - Supplemental results 6
2.1. Morphological description and comparison of factor categories 6
Character states coding: 7
Factor growth form 9
Factor habitat 15
Factor climate 19
Factor region 25
2.2. Visualisation of factor categories in the morphospace 32
2.3. Variation of disparity when isolating factor categories. 34
Growth form 35
Habitat 36
Climate 37
Region 38
2.4. Permutation test (latitudinal disparity gradients of disparity vs. species richness) 39
2.5. Latitudinal distribution of all Ericales from GBIF. 40
3. Note S2 - Distribution maps of the study species 41
4. References 99

1. Methods S1

All analyses were performed using the software R v.3.5.1 (R Core Team, 2018). Functions are referred to in the following format: *function name*{*package name*}.

1.1. Taxon sampling and scoring of the morphological dataset

To analyse morphological diversity (disparity), we used the morphological dataset and taxon sampling from Chartier *et al.* (2017). This dataset describes 380 species belonging to 274 genera (79.5 % of all Ericales genera) covering all major clades of the order identified in taxonomic/phylogenetic studies (Rose *et al.*, 2018; Schönenberger *et al.*, 2005). It is composed of 36 morphological characters, describing the perianth (12 characters), the androecium (13 characters), the gynoecium (six characters), and general features (e.g. flower sex and size, five characters) of the anthetic flower. We recorded the dataset in the database PROTEUS (Sauquet, 2019). It contains a total of 12,512 data entries, with 13.4 % missing data. We took a strict exemplar approach for scoring traits, which means that we only scored a trait state for a given species if we could confirm that it was observed in this species (that is, we did not use any general family descriptions or make any assumptions that all species of a genus share the same character states). In addition, each data record is directly linked with the reference from which we have retrieved the information. The total morphological data set, including references and a detailed description of all characters and character states, is available in the online supplemental information of Chartier *et al.* (2017).

1.2. Growth form, habitat, climate, and geographic region

All species from the morphological dataset were coded also for the four following factors using the exact same scoring approach as outlined above: *growth form*, *habitat*, *climate type*, and *region*. In this manuscript, we use abridged expression such as « floral disparity of trees », or « disparity in cold areas ». Each of these expressions should be understood as for instance « floral disparity in species displaying an arborescent growth form », or « floral disparity in species distributed in cold areas ».

For each factor, the assignment of each species to one or more categories was made retrieving information from the literature cited in Chartier *et al.* (2017) for each species, and by crossing this information with the maps and descriptions from (Cox, 2001; Peel *et al.*, 2007; Loarie *et al.*, 2009). This new dataset is available as Supplementary Information and stored in the online database Proteus (Sauquet, 2019), with at least one bibliographic reference linked to each entry. It contains 1,800 new data entries (3.6 % missing data).

We divided *growth forms* into the five categories occurring in Ericales Kubitzki (2004): (1) *trees*, (2) *shrubs*, (3) *lianas and climbers*, (4) *herbs (including aquatic herbs)*, and (5) *root parasites*.

We defined *habitat* categories by taking the biome descriptions from Loarie *et al.* (2009), and simplifying them into the three habitat states: (1) *forests*, (2) *open habitats*, and (3) *wet habitats* (the latter including mangroves and flooded grassland/savannas).

For *climate type* (Fig. 4 a), we used the Köppen-Geiger climate classification based on temperature and precipitation applying the five main categories described in Peel *et al.* (2007): (1) *tropical* (temperature of the coldest month ≥ 18 °C, either rainforest, monsoon, or savannah), (2) *arid* (desert and hot or cold steppes), (3) *temperate* (temperature of the hottest month > 10 °C, temperature of the coldest month between 0 °C and 18 °C, either dry summer, dry winter, or without dry season), (4) *cold* (temperature of the hottest month > 10 °C and temperature of the coldest month ≤ 0 °C, with dry summer or dry winter), and (5) *polar* (temperature of the hottest month < 10 °C, either tundra or frost).

Finally, we divided *regions* into (1) *North America*, (2) *Eurasia*, (3) *South America*, (4) *Africa*, (5) *Indo-Pacific*, and (6) *Australia* (Fig. 4 b). We followed the revised biogeographical delimitations of Floral Kingdoms as suggested by Cox (2001), dividing the Holarctic Kingdom into North America and Eurasia to account for continent delimitations. Each species was assigned to its native region only.

As pointed out above, our taxon sampling aimed to cover all major ericalean clades and most genera with the goal to optimize the estimation of disparity for the order as a whole (Chartier *et al.*, 2017). For this study, we thus assume that the distribution of the sampled species for each factor category is also representative for the order.

1.3. Creation of the association network.

Multiple correlations/associations are usually visualized on a correlation table (Fig. S1a). Instead, we propose to use an ordination, on which associated categories are grouped together, and significantly associated categories are linked by lines whose colour represent the strength of the association (here red for a positive association, blue for a negative association, Fig. S1b). This type of representation allows visualizing multiple associations very easily.

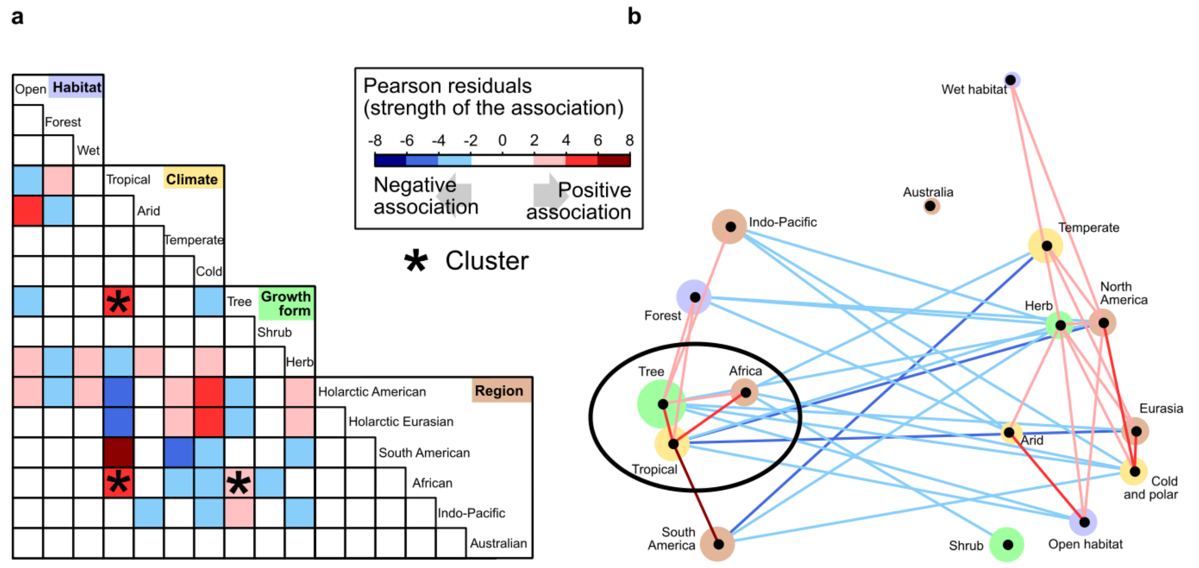

Figure S1. a correlation table displaying positive (in red) and negative (in blue) significant associations among categories, obtained after a series of chi-square tests and based on the value of Pearson residuals. As an example, a group of three categories that are significantly positively associated is highlighted by black stars. **b** association network representing the same associations from the same dataset. Significantly associated categories are linked by a line, whose colour indicates the strength of the association. The cluster highlighted in **a** by stars is shown in **b** with a black circle.

For significant chi-squared tests, the strength of the association can be estimated from the Pearson residuals (PR). *PR* > 2 indicates more observations than expected and *PR* < -2 indicates fewer observations than expected (Hawkins, 2014). To easily visualize clusters of associated factor categories, we transformed the *PR* into a distance index, d_{PR}, varying between 0 and 1, so that, for a pair of factors:

$$d_{\mathrm{PR}} = (PR + m)/2m$$

with *m* the maximum absolute Pearson residual value from the analyses (in our case, PR ranged from -5.98 to 7.28 so *m* was set to 8). This way, d_{PR} is minimum when two categories are associated more often than expected (i.e. when their *PR* is maximum), *d* is maximum when two categories are associated less often than expected (i.e. when their *PR* is minimum), and close to 0.5 when two categories are not associated. We created a distance matrix containing d_{PR} for all pairs of categories, with a value of d_{PR} set to 0.5 for pairs of categories belonging to the same factor. This matrix was then visualized with a non-metric multidimensional scaling (nMDS) using the function *metaMDS*{*vegan*} (Oksanen *et al.*, 2017). The nMDS is a method consisting in representing at best distances from a distance matrix in a number of dimensions specified by the user. The way we calculated *d* and using this method leads associated categories to fall close together in the representation, allowing an easy graphical identification of clusters of associated factor categories. The

strength of the association (*PR* values) is depicted by the colour of lines linking the categories that are significantly associated (interpretations of this type of graph should be made based on those lines rather than on the distances between dots on the graph).

1.4. Latitudinal distribution of species richness and disparity

We estimated the latitudinal distribution of the species from the dataset by extracting data from the Global Biodiversity Information Facility online database (GBIF, https://www.gbif.org/). For each species, location records (latitude and longitude) were extracted and filtered using the function *occ_search*{*rgbif*} (Chamberlain, 2017). Records where retained if they had "no geospatial issue", and if they belonged to the categories "HUMAN_OBSERVATION", "LITERATURE", "PRESERVED_SPECIMEN", or "OBSERVATION". Distribution maps (Supplementary Information) were then plotted using the package *maptools* (Bivand & Lewin-Koh, 2017) and all of these maps were manually checked for atypical and non-native records by using data from the literature and online trustworthy websites (such as the IUCN website, http://www.iucnredlist.org). This allowed us to estimate the presence/absence of 347 (91 %) of the study species in each ten-degree latitude interval across the globe.

Disparity ($\overline{\boldsymbol{D}}$) was then calculated for the species occurring in each given latitude interval. Finally, we tested for the correlations between latitude and species richness, latitude and disparity, and species richness and disparity (for each latitudinal interval) with Pearson correlation tests using the functions *cor.test*{*stats*} and *lm*{*stats*}.

1.5. Potential methodological shortcomings

[1] GBIF data only provide an underestimation of species richness. We corrected each distribution by hand and deleted the observations that were outside the natural distribution range of the study species. The distribution of some species might be underestimated, as we did not add records that were not in GBIF.

[2] To verify if the GBIF sample of Ericales we worked with is representative of the whole order, we extracted the latitudinal distribution of all Ericales (7,152 species) from GBIF and compared it to the distribution of species from our dataset (350 species). The two distributions were very similar, with a discrepancy in the northern hemisphere, where our sample contained proportionally more species than there were in the total distribution. This is could be due to the fact that our sample contains numerous cultivated species that, in the non-corrected dataset, are recorded in Europe and North America (e.g. *Actinidia* or *Camelia*). Correlation between these two distributions (number of species per latitudinal interval): r^2=0.91, $p<10^{-6}$; linear regression: slope=0.77 (probably because of the bias in the northern hemisphere), intercept=1.27.

[3] The gradient of disparity could be due to the increase in sample size near the tropical latitudes (although disparity is an index that is quite robust to sample size differences). The rarefaction analysis below shows that our result holds even when correcting for sample size:

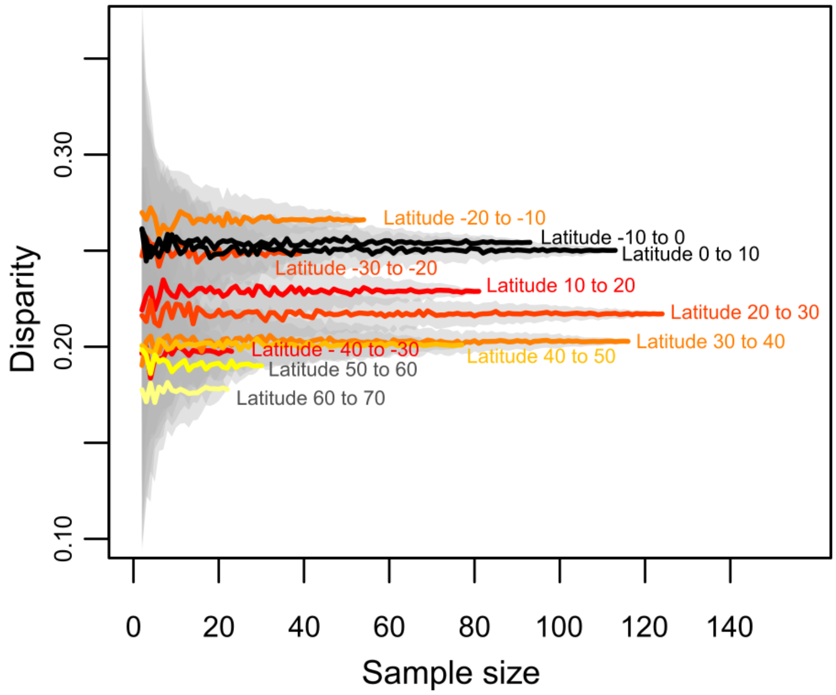

Figure S2. Disparity, averaged for each latitudinal slice over 200 random samples of the dataset. Sample sizes (x axis) range from 2 species to the maximum number of species possibly sampled for each latitudinal slice. In grey: standard deviation.

2. Note S1 - Supplemental results

2.1. Morphological description and comparison of factor categories

We used non parametric multivariate analyses of variance (npMANOVAS) to compare the morphological traits of each factor category, with the function *adonis*{*vegan*} in R and the same test used as posthoc, with a Bonferroni correction. Each factor category was then described using barplots as follow: *green* barplots give the distribution (frequency) of all morphological character states for a given factor category. *Gray* barplots give the frequencies of the same morphological states for the *rest of the dataset*. In case of polymorphism, species are included in each category they belong to (here in the green and in the gray barplots).
Such a representation allows visualising what makes a factor category different from the other categories (e.g. what differentiates *trees* from the other *growth forms*).
The number of species sampled per factor category is given in the first cell of each plate.

Legend for the following figures: barplots displaying the frequency of each character state for each factor state. In green: number of species of a given category (for example "*trees"*) displaying a given character state (for example "bisexual flowers only"). In gray: number of species of all the other categories from the same factor (for example "shrubs" and "lianas" and "herbs") displaying the same character state.

Character states coding:

Functional sex:
- a = bisexual flowers only
- b = unisexual flowers only
- c = both

Structural sex:
- a = bisexual flowers only
- b = unisexual flowers only
- c = both

Nectaries and oil:
- a = absence
- b = presence

Flower length:
- a = small (<1cm)
- b = medium (1 to 3 cm)
- c = large (>3cm)

Flower diameter:
- a = small (<1cm)
- b = medium (1 to 3 cm)
- c = large (>3cm)

Perianth differentiation:
- a = no/weak differentiation
- b = continuous differentiation
- c = marked differentiation

Sepal phyllotaxis:
- a = whorled
- b = spiral

Petal phyllotaxis:
- a = whorled
- b = spiral

Sepal aestivation:
- a = apert
- b = imbricate
- c = valvate

Petal aestivation:
- a = apert
- b = imbricate
- c = valvate

Perianth symmetry:
- a = polysymmetric
- b = monosymmetric
- c = disymmetric
- d = asymmetric

Sepal union:
- a = free/basally united
- b = more than 10% union

Petal union:
- a = free/basally united
- b = more than 10% union

Distal filament union:
- a = absent
- b = present

Androecium phyllotaxis:
- a = whorled
- b = stamens in pairs (whorled)
- c = ring primordium
- d = fascicles

Number of androecium whorls:
- a = one
- b = two
- c = more than two

Androecium symmetry:
- a = polysymmetric
- b = monosymmetric

Filament union:
- a = free/basally united
- b = more than 10% united

Anther union:
- a = not united
- b = united

Anther orientation:
- a = introrse
- b = latrorse
- c = extrorse
- d = apical

Anther attachment:
- a = basifixed
- b = dorsifixed
- c = ventrifixed

Anther dehiscence:
- a = longitudinal slit
- b = apically poricidal
- c = basally poricidal
- d = common stomium of confluent thecae
- e = transverse (horizontal) slit

Filament insertion to corolla:
- a = no fusion or basally fused
- b = lower half inserted
- c = upper half inserted

Filament fusion to corolla:
a = no fusion or basally attached
b = more than 10% fusion

Staminodes:
a = fused to form a corolla-like structure
b = stamen-like
c = hood-like structure
d = scale-like
e = petaloid
f = triangular, wooly or hairy, applied to the style at anthesis
g = no staminodes
h = petal-like

Ovary position:
a = inferior
b = superior

Gynoecium symmetry:
a = polysymmetric
b = monosymmetric
c = disymmetric

Style union:
a = free or basally united
b = more than 10% union

Placentation:
a = axile
b = free-central
c = parietal

Number of ovules per carpel:
a = one
b = two
c = three to ten
d = more than ten

Ovule integuments:
a = one (unitegmic)
b = two (bitegmic)

Factor growth form

There were significant floral morphological differences among growth forms (npMANOVA: F = 7.60, $r^2 = 0.05$, $p < 10^{-03}$). According to posthoc tests, only *lianas and climbers* did not significantly differ from the other growth forms.

```
##                    Df SumsOfSqs  MeanSqs F.Model      R2 Pr(>F)
##                     3    0.5994 0.199815  7.6001 0.04812  1e-04 ***
## Residuals         451   11.8573 0.026291         0.95188
## Total             454   12.4568                  1.00000
## ---
## Signif. codes:  0 '***' 0.001 '**' 0.01 '*' 0.05 '.' 0.1 ' ' 1

## [1] "POSTHOC Corrected alpha:  0.008333"
```

```
upper diagonal: r²
lower diagonal: p
##       Herb  Lian   Shr   Tre
## Herb    NA 0.031 0.023 0.018
## Lian 0.037    NA 0.008 0.016
## Shr  0.002 0.233    NA 0.036
## Tre  0.002 0.009 0.001    NA
```

```
upper diagonal: F
lower diagonal: significance level (* = significant p-value, ns = non significant
p-value).
##      Herb  Lian   Shr    Tre
## Herb <NA> 2.793  5.33  5.161
## Lian   ns  <NA> 1.324  3.742
## Shr     *    ns  <NA> 13.720
## Tre     *    ns     *     NA
```

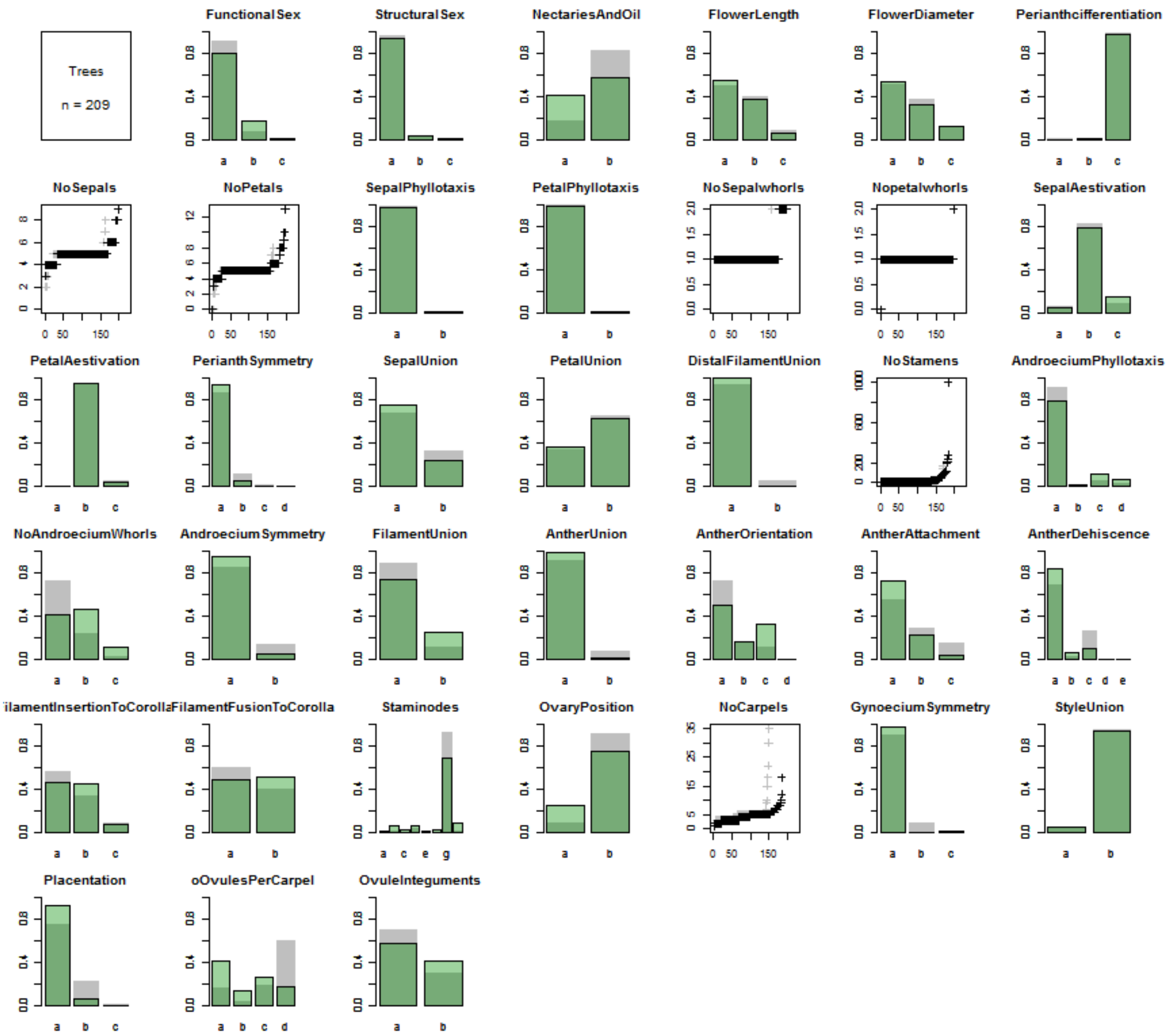

E.g.: in green: distribution of the morphological character states for trees; in gray: for the other growth forms present in the dataset.

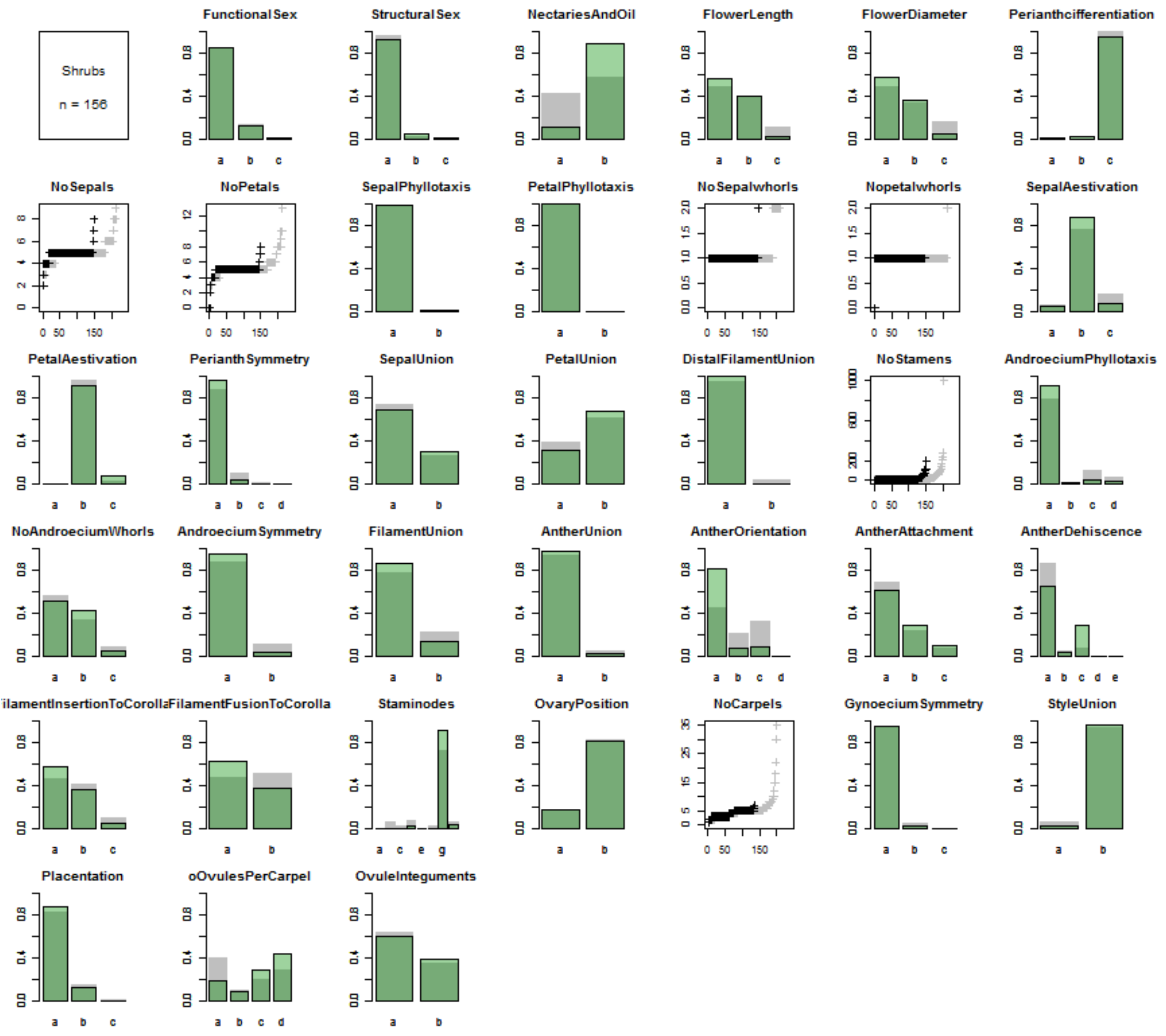
Shrubs
n = 156
FunctionalSex
StructuralSex
NectariesAndOil
FlowerLength
FlowerDiameter
Perianthcifferentiation
NoSepals
NoPetals
SepalPhyllotaxis
PetalPhyllotaxis
NoSepalwhorls
Nopetalwhorls
SepalAestivation
PetalAestivation
PerianthSymmetry
SepalUnion
PetalUnion
DistalFilamentUnion
NoStamens
AndroeciumPhyllotaxis
NoAndroeciumWhorls
AndroeciumSymmetry
FilamentUnion
AntherUnion
AntherOrientation
AntherAttachment
AntherDehiscence
FilamentFusionToCorolla
Staminodes
OvaryPosition
NoCarpels
GynoeciumSymmetry
StyleUnion
Placentation
oOvulesPerCarpel
OvuleIntegunents

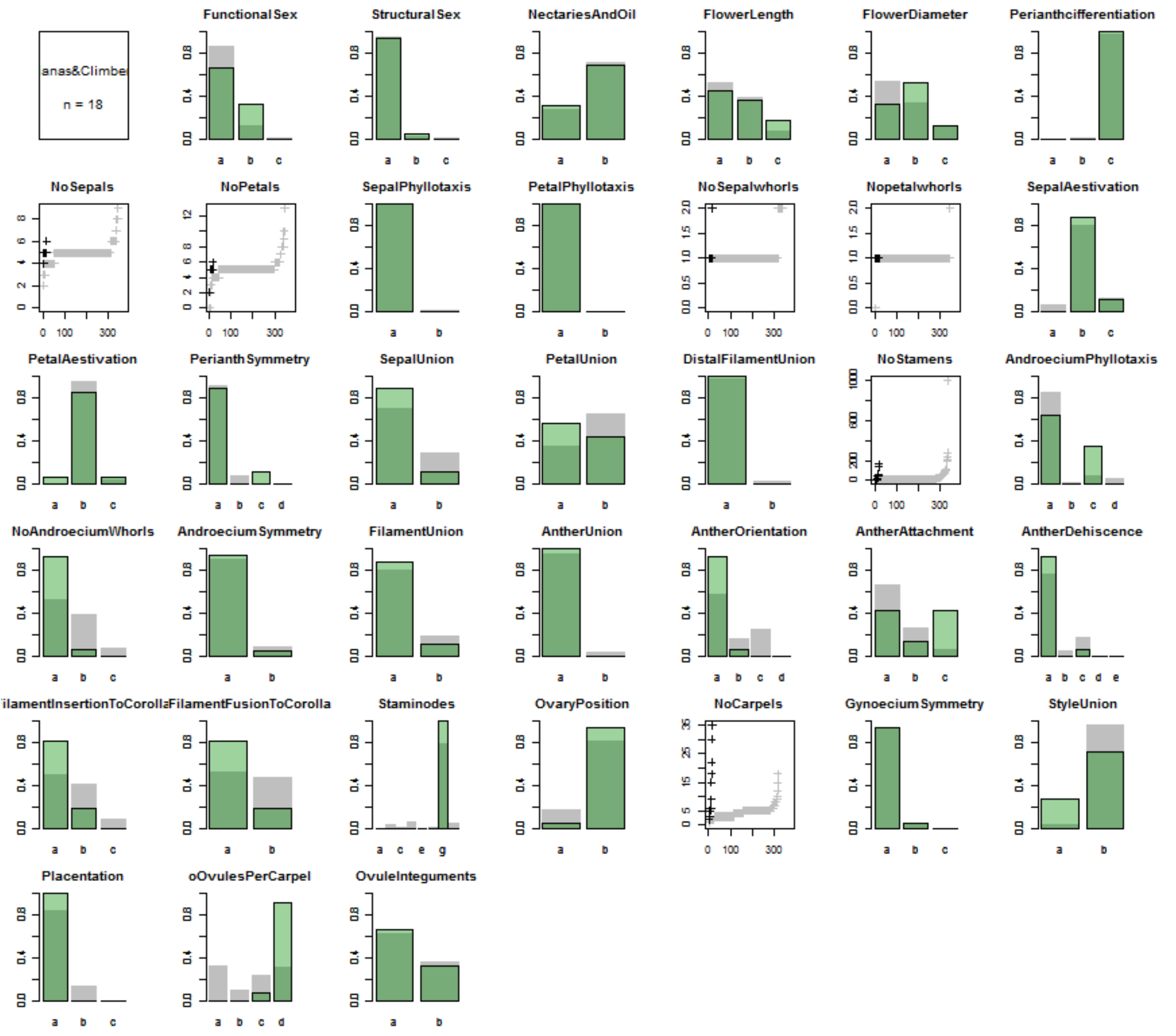
anas&Climbe
n = 18
FunctionalSex
StructuralSex
NectariesAndOil
FlowerLength
FlowerDiameter
Perianthcifferentiation
NoSepals
NoPetals
SepalPhyllotaxis
PetalPhyllotaxis
NoSepalwhorls
Nopetalwhorls
SepalAestivation
PetalAestivation
PerianthSymmetry
SepalUnion
PetalUnion
DistalFilamentUnion
NoStamens
AndroeciumPhyllotaxis
NoAndroeciumWhorls
AndroeciumSymmetry
FilamentUnion
AntherUnion
AntherOrientation
AntherAttachment
AntherDehiscence
ilamentInsertionToCorolla
FilamentFusionToCorolla
Staminodes
OvaryPosition
NoCarpels
GynoeciumSymmetry
StyleUnion
Placentation
oOvulesPerCarpel
OvuleInteguments

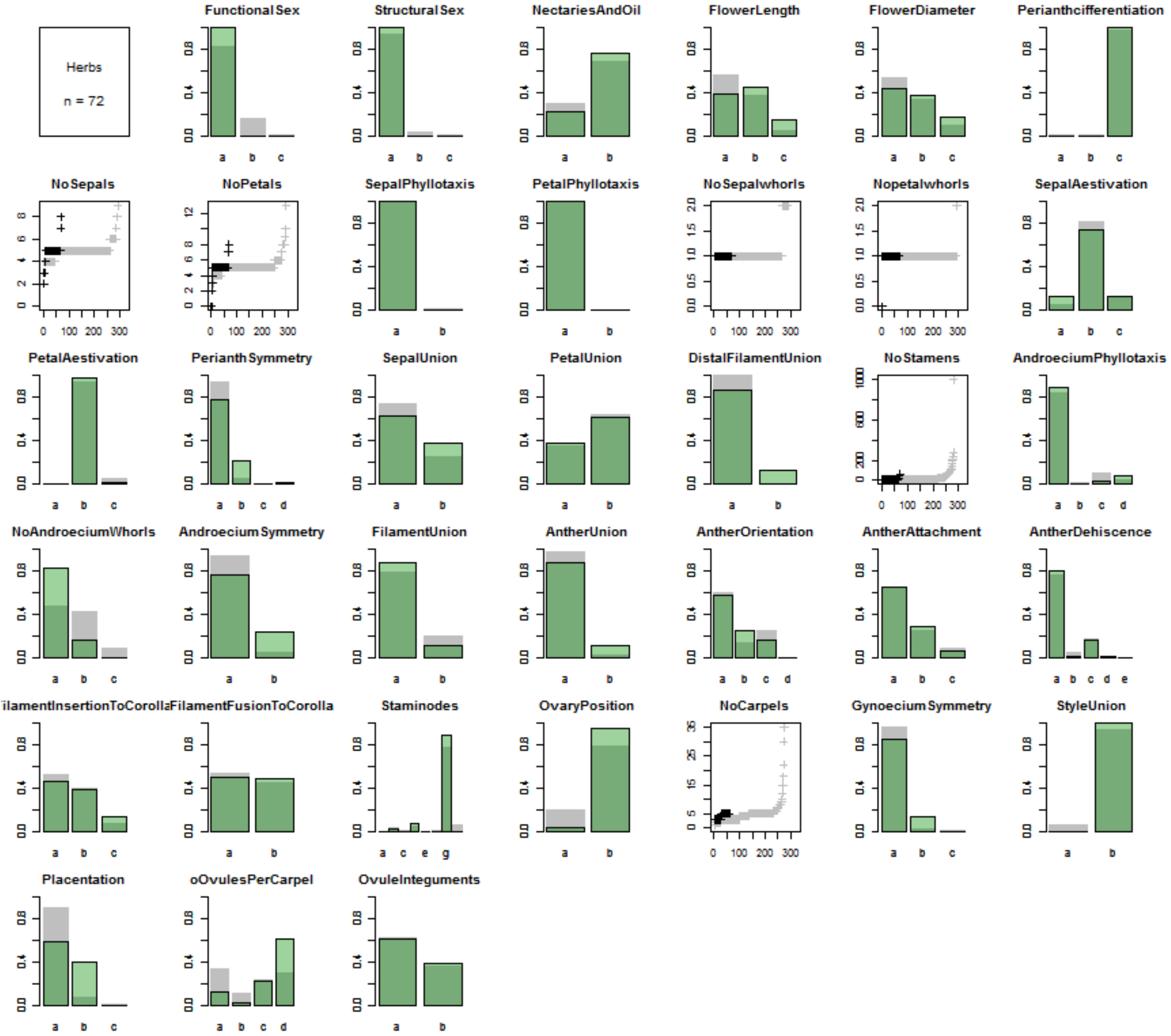
Herbs
n = 72
FunctionalSex
StructuralSex
NectariesAndOil
FlowerLength
FlowerDiameter
Perianthcifferentiation
NoSepals
NoPetals
SepalPhyllotaxis
PetalPhyllotaxis
NoSepalwhorls
Nopetalwhorls
SepalAestivation
PetalAestivation
PerianthSymmetry
SepalUnion
PetalUnion
DistalFilamentUnion
NoStamens
AndroeciumPhyllotaxis
NoAndroeciumWhorls
AndroeciumSymmetry
FilamentUnion
AntherUnion
AntherOrientation
AntherAttachment
AntherDehiscence
ilamentInsertionToCorolla
FilamentFusionToCorolla
Staminodes
OvaryPosition
NoCarpels
GynoeciumSymmetry
StyleUnion
Placentation
oOvulesPerCarpel
OvuleInteguments

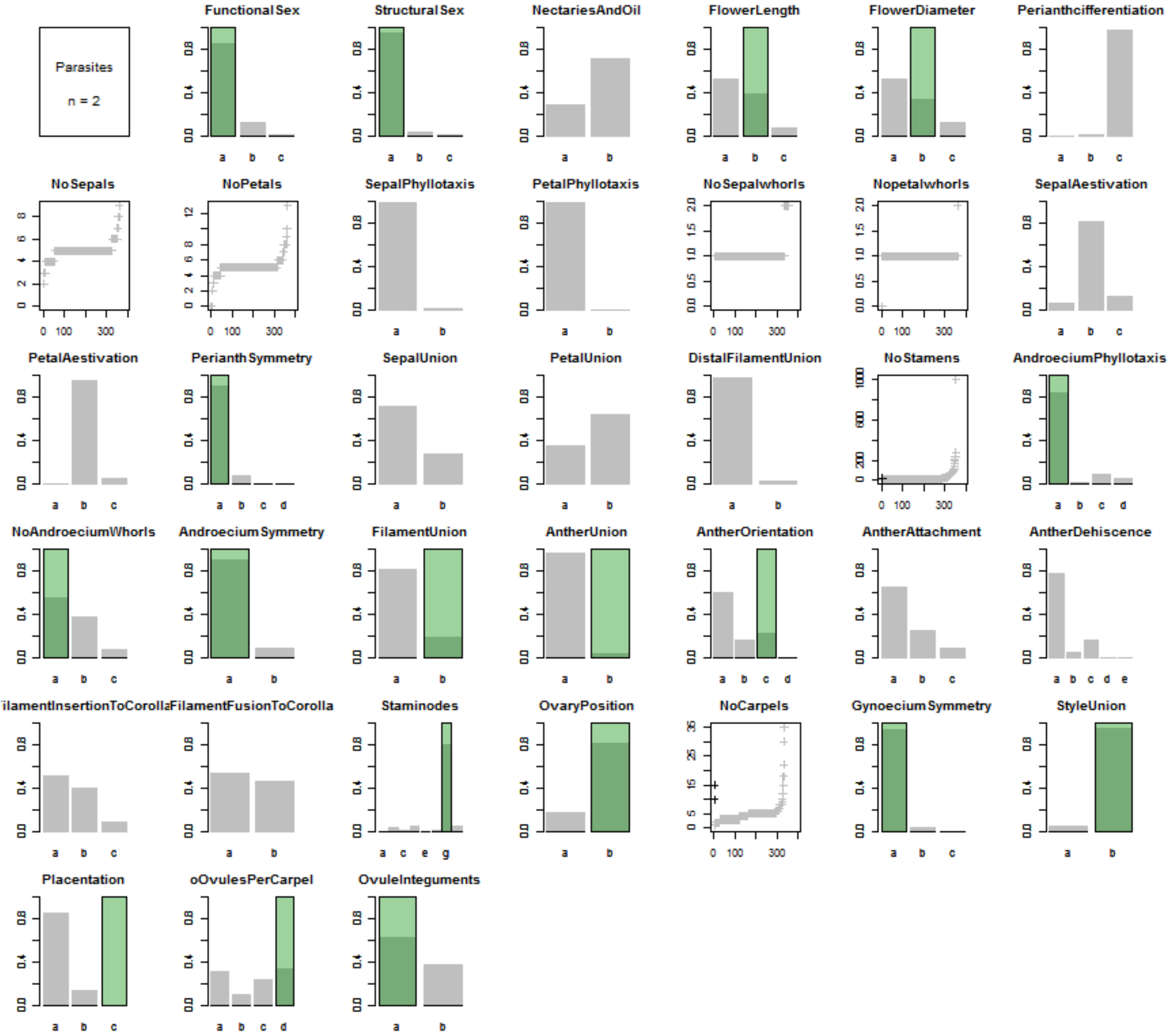
Parasites
n = 2
FunctionalSex
StructuralSex
NectariesAndOil
FlowerLength
FlowerDiameter
Perianthcifferentiation
NoSepals
NoPetals
SepalPhyllotaxis
PetalPhyllotaxis
NoSepalwhorls
Nopetalwhorls
SepalAestivation
PetalAestivation
PerianthSymmetry
SepalUnion
PetalUnion
DistalFilamentUnion
NoStamens
AndroeciumPhyllotaxis
NoAndroeciumWhorls
AndroeciumSymmetry
FilamentUnion
AntherUnion
AntherOrientation
AntherAttachment
AntherDehiscence
ilamentInsertionToCorolla
FilamentFusionToCorolla
Staminodes
OvaryPosition
NoCarpels
GynoeciumSymmetry
StyleUnion
Placentation
oOvulesPerCarpel
OvuleInteguments

Factor habitat

There were significant floral morphological differences among types of habitat (npMANOVA: F = 2.36, $r^2 = 0.01$, p = 0.0182). According to posthoc tests, only *forests* and *open habitats* significantly differed.

```
##                     Df SumsOfSqs  MeanSqs F.Model      R2 Pr(>F)
##                      2    0.1272 0.063599  2.3568 0.01258 0.0182 *
## Residuals          370    9.9847 0.026986         0.98742
## Total              372   10.1119                  1.00000
## ---
## Signif. codes:  0 '***' 0.001 '**' 0.01 '*' 0.05 '.' 0.1 ' ' 1


## [1] "POSTHOC Corrected alpha:  0.016667"

upper diagonal: r²
lower diagonal: p
##       For   Ope   Wet
## For    NA 0.016 0.005
## Ope 0.001    NA 0.014
## Wet 0.225 0.123    NA

upper diagonal: F
lower diagonal: significance level (* = significant p-value, ns = non signi
ficant p-value).
##      For   Ope   Wet
## For <NA> 5.707 1.381
## Ope    *  <NA> 1.825
## Wet   ns    ns    NA
```

The morphological characteristics of each habitat category can be read on the graphs below.

Open habitats differ from the rest of the dataset in having a higher proportion of flowers with apert petals, of flowers with united petals, of stamens inserted in one whorl, of free filaments, of introrse anthers, of dorsifixed and ventrifixed (instead of basifixed) anthers, of superior ovaries, of numerous carpels, of more than 10 ovules per carpel and of unitegmic ovules.

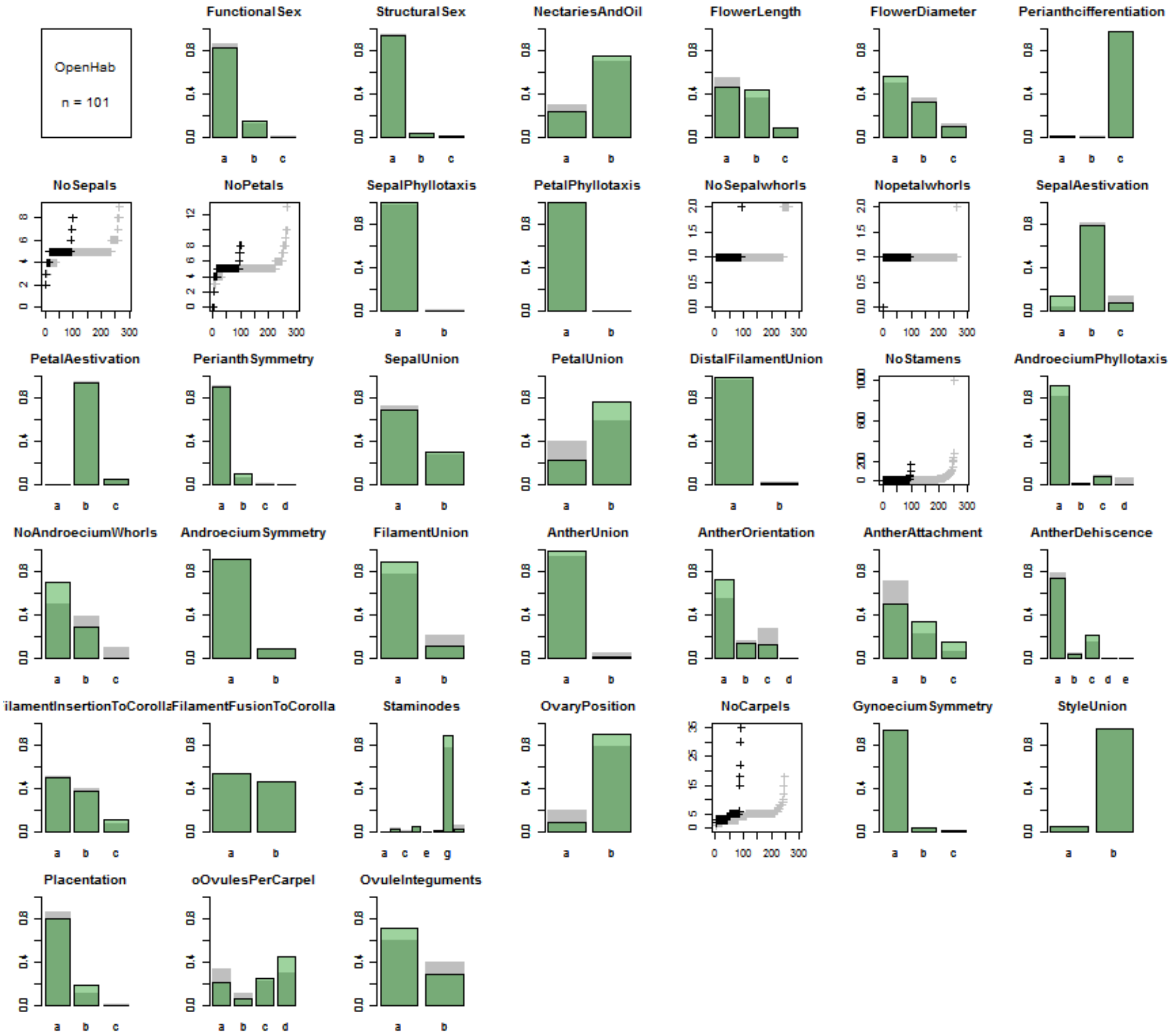
OpenHab
n = 101
FunctionalSex
StructuralSex
NectariesAndOil
FlowerLength
FlowerDiameter
Perianthcifferentiation
NoSepals
NoPetals
SepalPhyllotaxis
PetalPhyllotaxis
NoSepalwhorls
Nopetalwhorls
SepalAestivation
PetalAestivation
PerianthSymmetry
SepalUnion
PetalUnion
DistalFilamentUnion
NoStamens
AndroeciumPhyllotaxis
NoAndroeciumWhorls
AndroeciumSymmetry
FilamentUnion
AntherUnion
AntherOrientation
AntherAttachment
AntherDehiscence
FilamentInsertionToCorolla
FilamentFusionToCorolla
Staminodes
OvaryPosition
NoCarpels
GynoeciumSymmetry
StyleUnion
Placentation
oOvulesPerCarpel
OvuleIntegruments

Forests differ from the rest of the dataset in having a higher proportion of species whose flowers

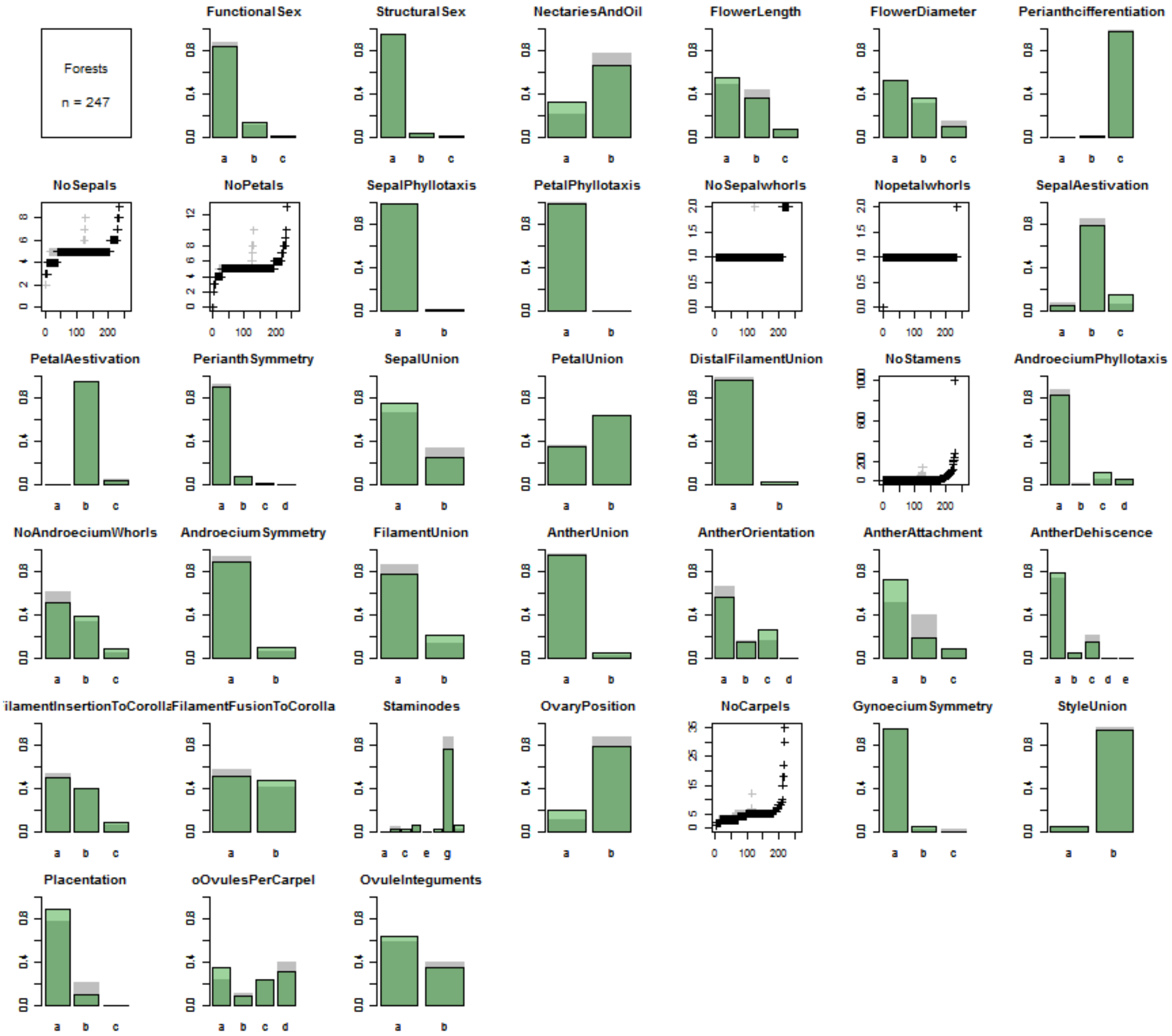

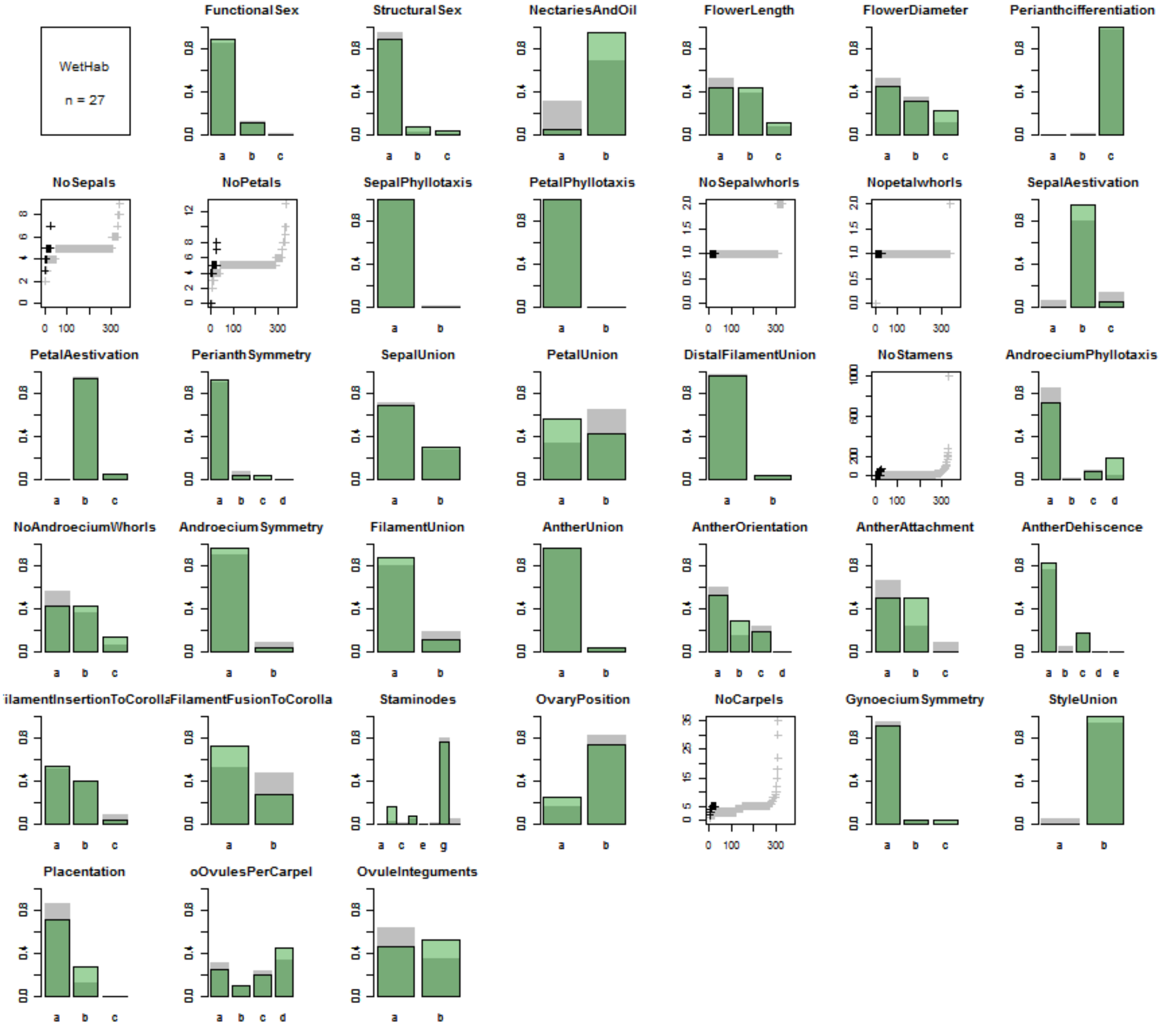
WetHab
n = 27
FunctionalSex
StructuralSex
NectariesAndOil
FlowerLength
FlowerDiameter
Perianthcifferentiation
NoSepals
NoPetals
SepalPhyllotaxis
PetalPhyllotaxis
NoSepalwhorls
Nopetalwhorls
SepalAestivation
PetalAestivation
PerianthSymmetry
SepalUnion
PetalUnion
DistalFilamentUnion
NoStamens
AndroeciumPhyllotaxis
NoAndroeciumWhorls
AndroeciumSymmetry
FilamentUnion
AntherUnion
AntherOrientation
AntherAttachment
AntherDehiscence
ilamentInsertionToCorolla
FilamentFusionToCorolla
Staminodes
OvaryPosition
NoCarpels
GynoeciumSymmetry
StyleUnion
Placentation
oOvulesPerCarpel
OvuleInteguments

Factor climate

There were significant floral morphological differences among climate categories (npMANOVA: F = 3.75, $r^2 = 0.03$, $p < 10^{-03}$). According to posthoc tests, only species growing under *tropical* climate significantly differed from the other categories (all but *arid*). The morphological characteristics of each *climate* category can be read on the graphs below.

```
##                     Df SumsOfSqs  MeanSqs F.Model      R2 Pr(>F)
##                      4    0.3862 0.096548  3.7527 0.03153  1e-04 ***
## Residuals          461   11.8605 0.025728         0.96847
## Total              465   12.2467                  1.00000
## ---
## Signif. codes:  0 '***' 0.001 '**' 0.01 '*' 0.05 '.' 0.1 ' ' 1

## [1] "POSTHOC Corrected p-value: Corrected p-value:  0.005"
```

```
upper diagonal: r²
lower diagonal: p
##         Ari  Cold   Tem   Pol  Trop
## Ari      NA 0.029 0.011 0.093 0.015
## Cold 0.041    NA 0.003 0.054 0.034
## Tem  0.059 0.660    NA 0.021 0.039
## Pol  0.007 0.015 0.012    NA 0.037
## Trop 0.011 0.001 0.001 0.001    NA
```

```
upper diagonal: F
lower diagonal: significance level (* = significant p-value, ns = non
significant p-value).
##        Ari  Cold   Tem   Pol   Trop
## Ari  <NA> 2.699 2.315 4.216  3.600
## Cold   ns  <NA> 0.617 3.444  8.861
## Tem    ns    ns  <NA> 3.743 14.697
## Pol    ns    ns    ns  <NA>  7.778
## Trop   ns     *     *     *     NA
```

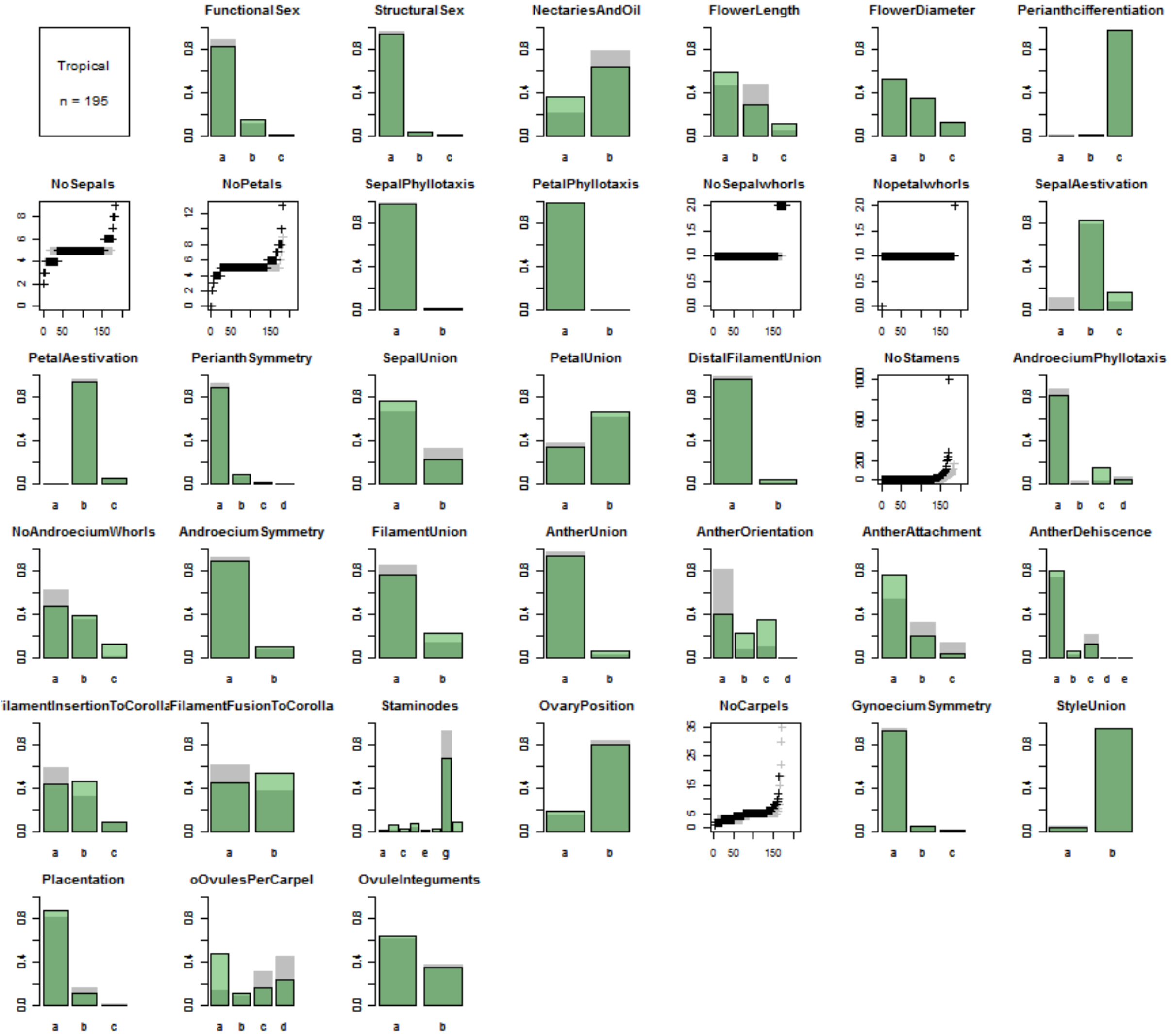
Tropical
n = 195
FunctionalSex
StructuralSex
NectariesAndOil
FlowerLength
FlowerDiameter
Perianthcifferentiation
NoSepals
NoPetals
SepalPhyllotaxis
PetalPhyllotaxis
NoSepalwhorls
Nopetalwhorls
SepalAestivation
PetalAestivation
PerianthSymmetry
SepalUnion
PetalUnion
DistalFilamentUnion
NoStamens
AndroeciumPhyllotaxis
NoAndroeciumWhorls
AndroeciumSymmetry
FilamentUnion
AntherUnion
AntherOrientation
AntherAttachment
AntherDehiscence
ilamentInsertionToCorolla
FilamentFusionToCorolla
Staminodes
OvaryPosition
NoCarpels
GynoeciumSymmetry
StyleUnion
Placentation
oOvulesPerCarpel
OvuleIntegument

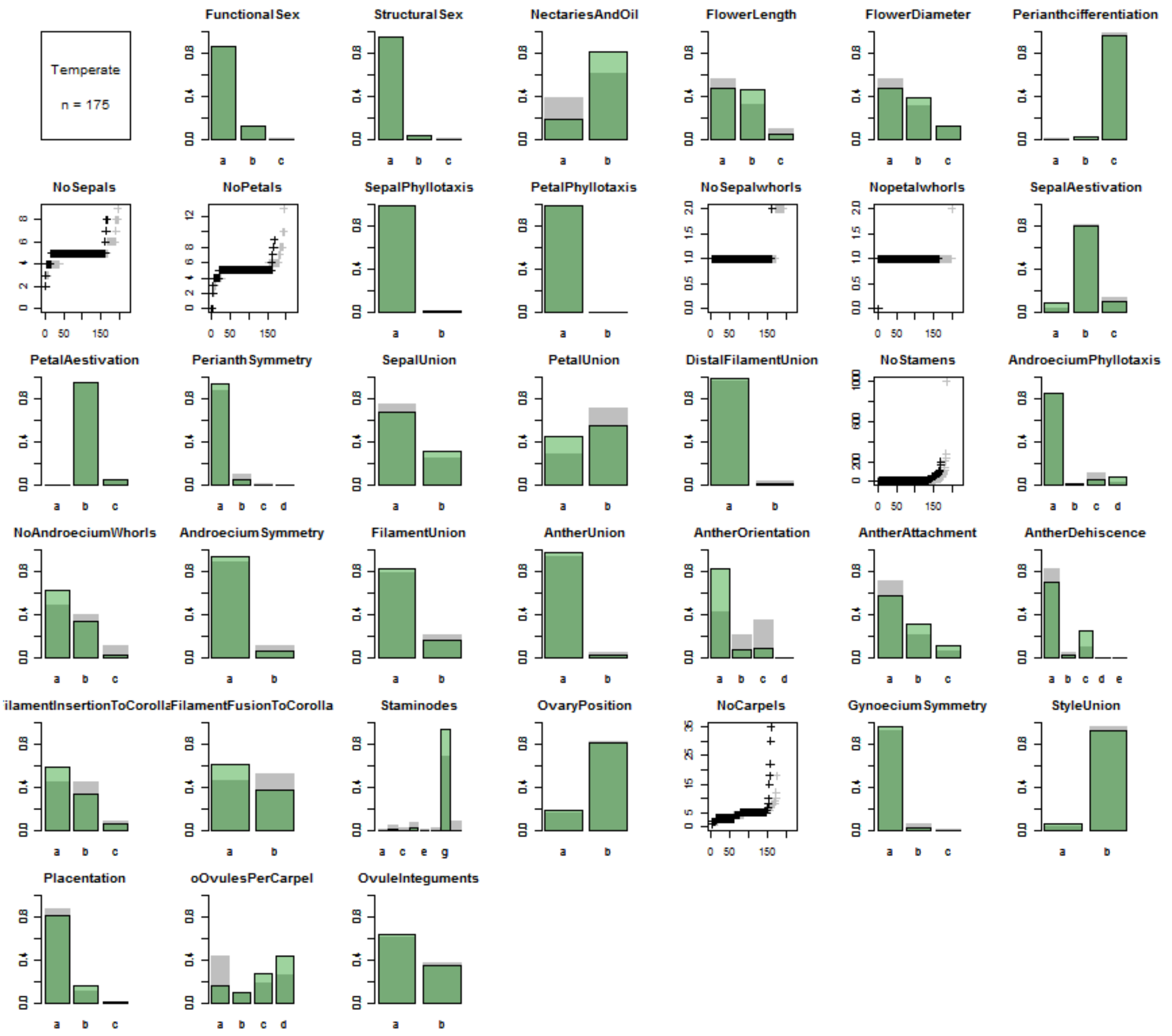
Temperate
n = 175
FunctionalSex
StructuralSex
NectariesAndOil
FlowerLength
FlowerDiameter
Perianthcifferentiation
NoSepals
NoPetals
SepalPhyllotaxis
PetalPhyllotaxis
NoSepalwhorls
Nopetalwhorls
SepalAestivation
PetalAestivation
PerianthSymmetry
SepalUnion
PetalUnion
DistalFilamentUnion
NoStamens
AndroeciumPhyllotaxis
NoAndroeciumWhorls
AndroeciumSymmetry
FilamentUnion
AntherUnion
AntherOrientation
AntherAttachment
AntherDehiscence
ilamentInsertionToCorolla
FilamentFusionToCorolla
Staminodes
OvaryPosition
NoCarpels
GynoeciumSymmetry
StyleUnion
Placentation
oOvulesPerCarpel
OvuleIntegurnents

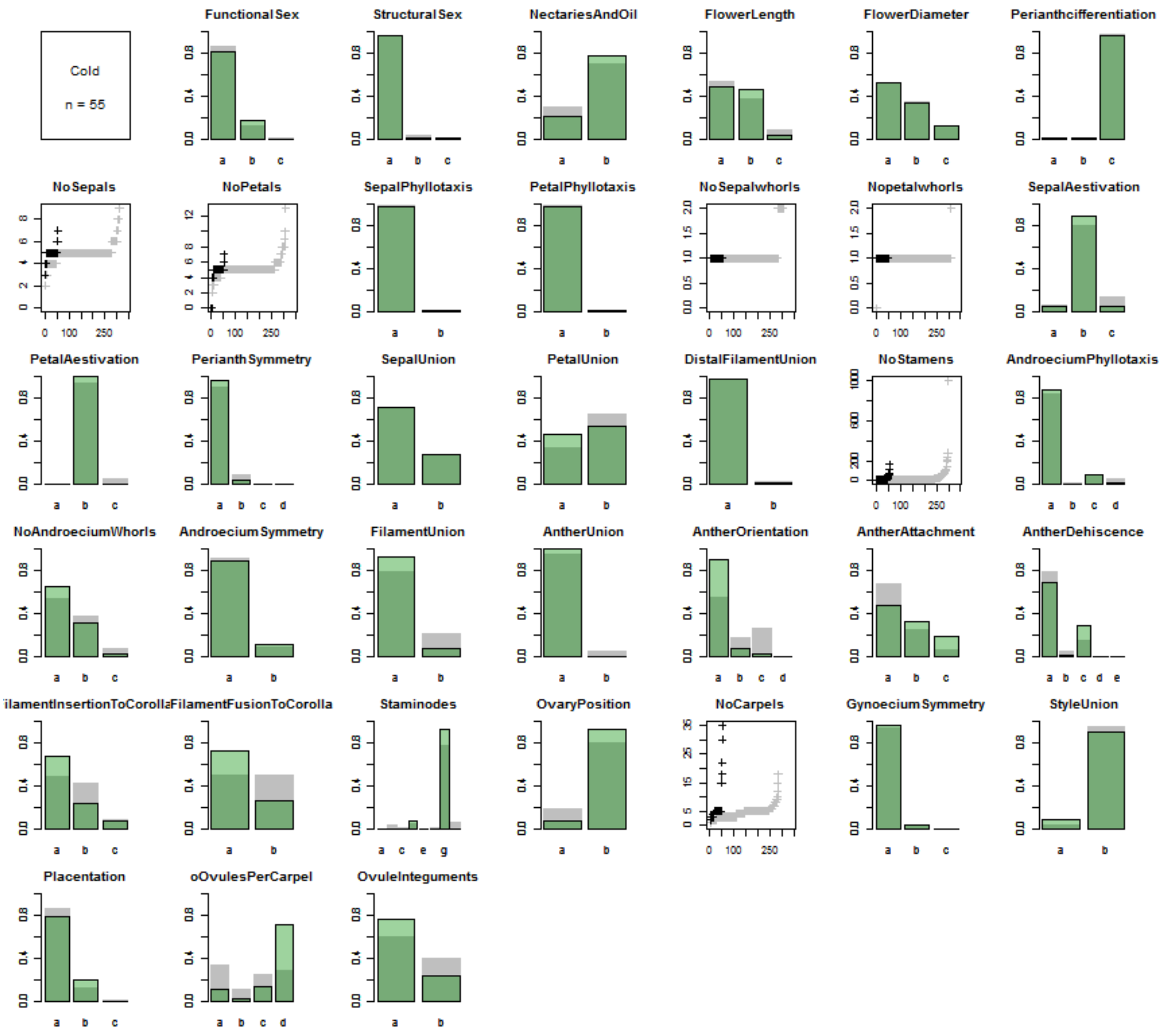
Cold
n = 55
FunctionalSex
StructuralSex
NectariesAndOil
FlowerLength
FlowerDiameter
Perianthcifferentiation
NoSepals
NoPetals
SepalPhyllotaxis
PetalPhyllotaxis
NoSepalwhorls
Nopetalwhorls
SepalAestivation
PetalAestivation
PerianthSymmetry
SepalUnion
PetalUnion
DistalFilamentUnion
NoStamens
AndroeciumPhyllotaxis
NoAndroeciumWhorls
AndroeciumSymmetry
FilamentUnion
AntherUnion
AntherOrientation
AntherAttachment
AntherDehiscence
FilamentInsertionToCorolla
FilamentFusionToCorolla
Staminodes
OvaryPosition
NoCarpels
GynoeciumSymmetry
StyleUnion
Placentation
oOvulesPerCarpel
OvuleIntegumentsments

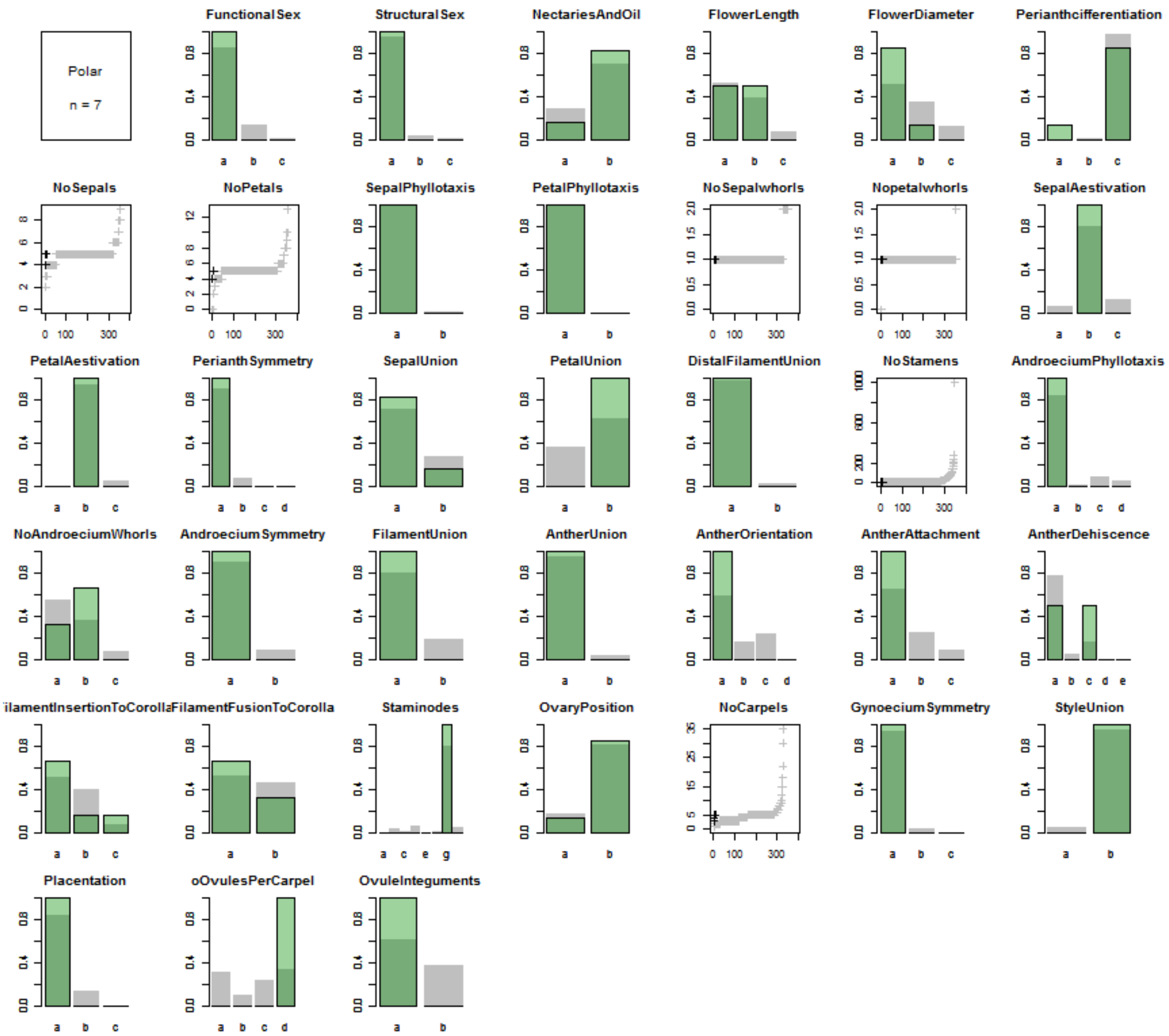
Polar
n = 7
Functional Sex
Structural Sex
NectariesAndOil
FlowerLength
FlowerDiameter
Perianthcifferentiation
NoSepals
NoPetals
SepalPhyllotaxis
PetalPhyllotaxis
No Sepalwhorls
Nopetalwhorls
SepalAestivation
PetalAestivation
Perianth Symmetry
SepalUnion
PetalUnion
DistalFilamentUnion
No Stamens
AndroeciumPhyllotaxis
NoAndroeciumWhorls
Androecium Symmetry
FilamentUnion
AntherUnion
AntherOrientation
AntherAttachment
AntherDehiscence
ilamentInsertionToCorolla
FilamentFusionToCorolla
Staminodes
OvaryPosition
NoCarpels
Gynoecium Symmetry
StyleUnion
Placentation
oOvulesPerCarpel
OvuleInteguments

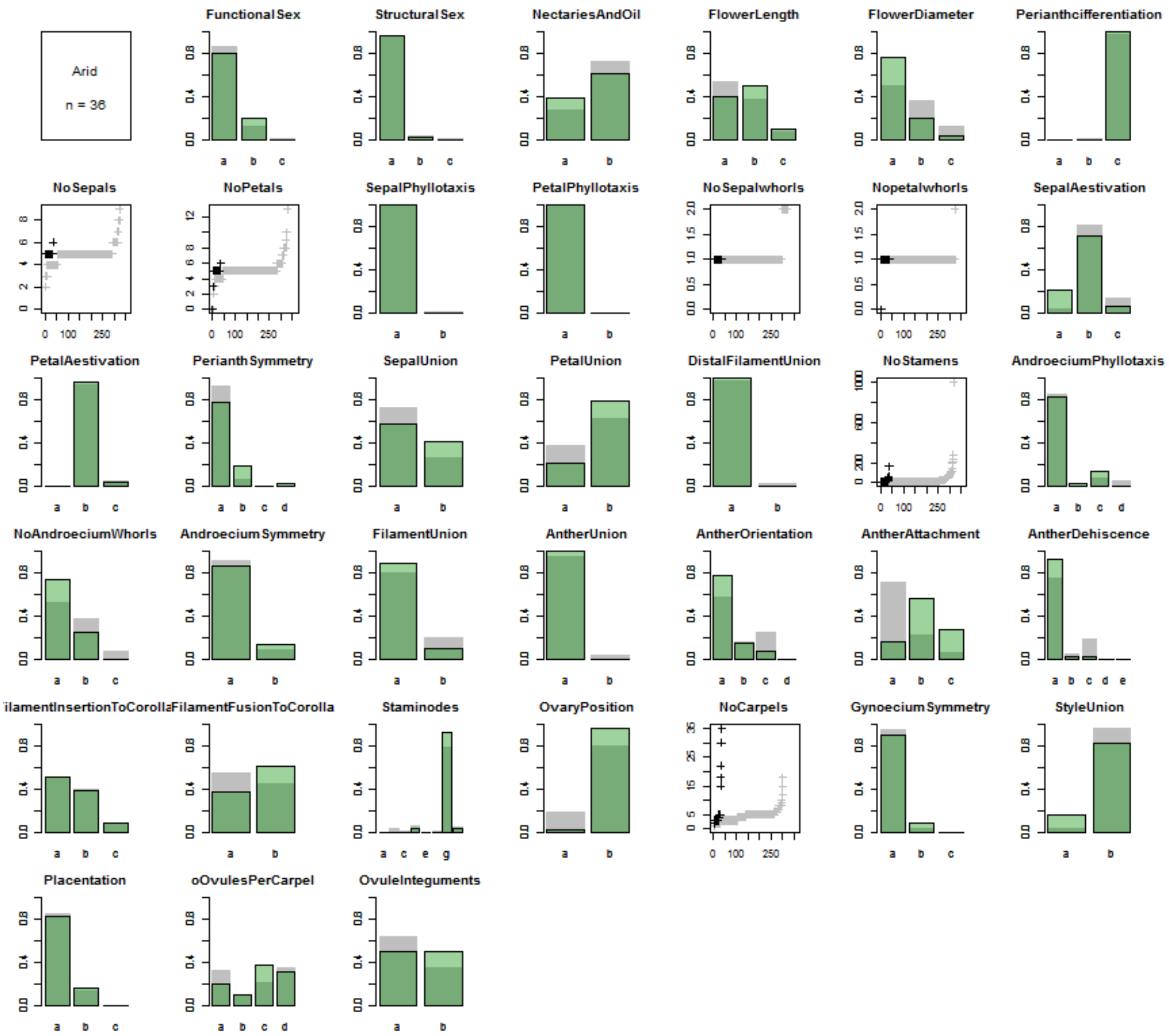
Arid
n = 36
FunctionalSex
StructuralSex
NectariesAndOil
FlowerLength
FlowerDiameter
Perianthcifferentiation
NoSepals
NoPetals
SepalPhyllotaxis
PetalPhyllotaxis
NoSepalwhorls
Nopetalwhorls
SepalAestivation
PetalAestivation
PerianthSymmetry
SepalUnion
PetalUnion
DistalFilamentUnion
NoStamens
AndroeciumPhyllotaxis
NoAndroeciumWhorls
AndroeciumSymmetry
FilamentUnion
AntherUnion
AntherOrientation
AntherAttachment
AntherDehiscence
ilamentInsertionToCorolla
FilamentFusionToCorolla
Staminodes
OvaryPosition
NoCarpels
GynoeciumSymmetry
StyleUnion
Placentation
oOvulesPerCarpel
OvuleIntegument

Factor region

There were significant floral morphological differences among regions (npMANOVA: F = 3.3, r^2 = 0.04, p < 10^{-03}). According to posthoc tests, species from *Africa* where the ones that differed from the most other regions. *North America* and *Australia* differed from Indo-Pacific. The morphological characteristics of each *region* category can be read on the graphs below.

```
##                    Df SumsOfSqs  MeanSqs F.Model      R2 Pr(>F)
##                     5    0.4311 0.086222     3.3 0.03806  1e-04 ***
## Residuals         417   10.8952 0.026127         0.96194
## Total             422   11.3263                  1.00000
## ---
## Signif. codes:  0 '***' 0.001 '**' 0.01 '*' 0.05 '.' 0.1 ' ' 1
```

```
## [1] "POSTHOC Corrected alpha:  0.003333"
```

```
upper diagonal: r²
lower diagonal: p
##       Afr   Eur   Ind   NAm   SAm   Aus
## Afr    NA 0.057 0.037 0.108 0.034 0.087
## Eur 0.001    NA 0.007 0.026 0.007 0.055
## Ind 0.001 0.290    NA 0.033 0.006 0.038
## NAm 0.001 0.007 0.001    NA 0.020 0.030
## SAm 0.001 0.241 0.297 0.014    NA 0.024
## Aus 0.001 0.004 0.003 0.047 0.042    NA
```

```
upper diagonal: F
lower diagonal: significance level (* = significant p-value, ns = non
significant p-value).
##      Afr   Eur   Ind    NAm   SAm   Aus
## Afr <NA> 7.902 6.285 15.575 5.795 6.805
## Eur    *  <NA> 1.179  3.639   1.3 4.711
## Ind    *    ns  <NA>  5.852  1.15 4.561
## NAm    *    ns     *   <NA> 3.514 2.469
## SAm    *    ns    ns     ns  <NA> 2.784
## Aus    *    ns     *     ns    ns    NA
```

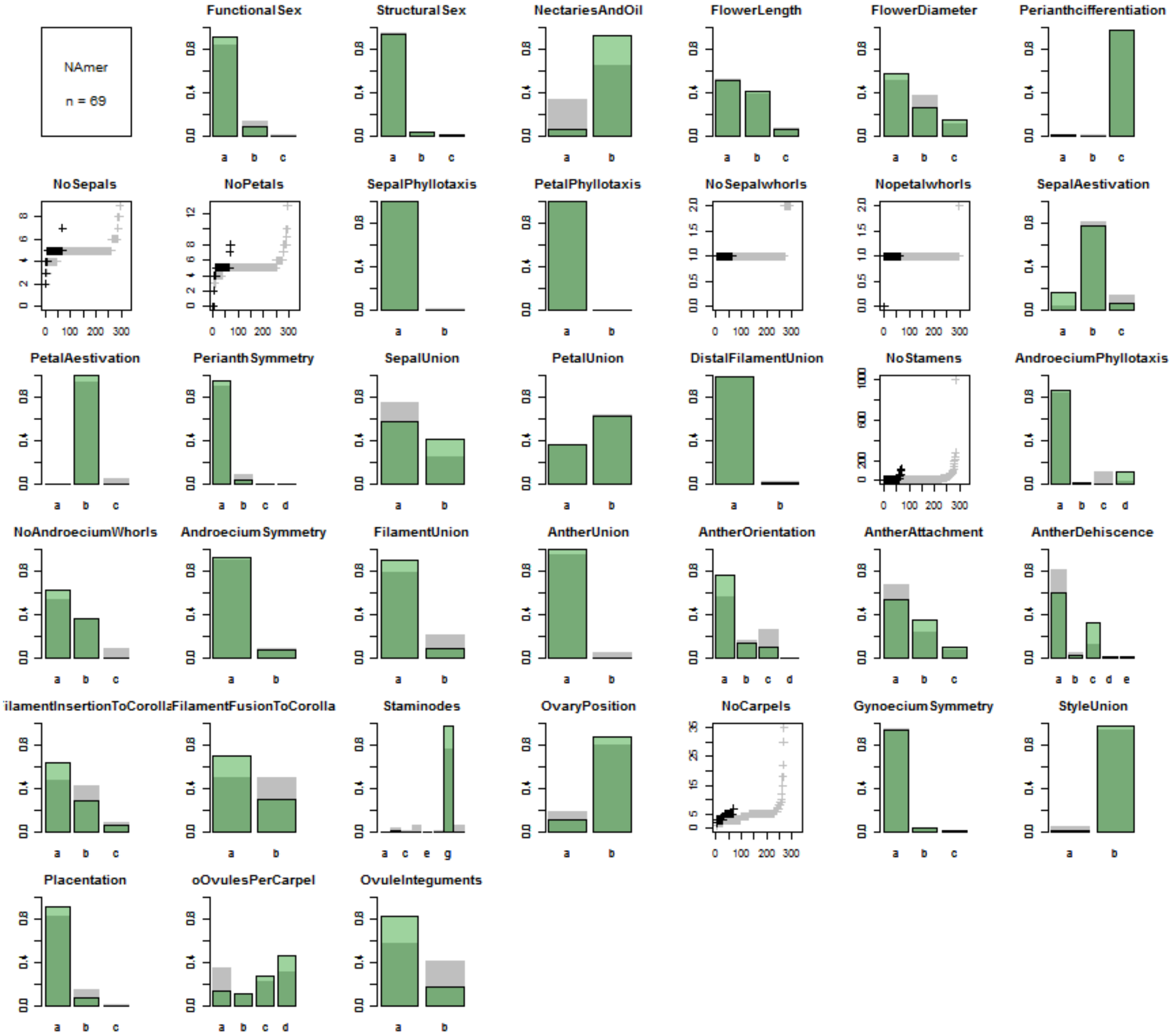
NAmer
n = 69
FunctionalSex
StructuralSex
NectariesAndOil
FlowerLength
FlowerDiameter
Perianthcifferentiation
NoSepals
NoPetals
SepalPhyllotaxis
PetalPhyllotaxis
NoSepalwhorls
Nopetalwhorls
SepalAestivation
PetalAestivation
PerianthSymmetry
SepalUnion
PetalUnion
DistalFilamentUnion
NoStamens
AndroeciumPhyllotaxis
NoAndroeciumWhorls
AndroeciumSymmetry
FilamentUnion
AntherUnion
AntherOrientation
AntherAttachment
AntherDehiscence
ilamentInsertionToCorolla
FilamentFusionToCorolla
Staminodes
OvaryPosition
NoCarpels
GynoeciumSymmetry
StyleUnion
Placentation
oOvulesPerCarpel
OvuleInteguments

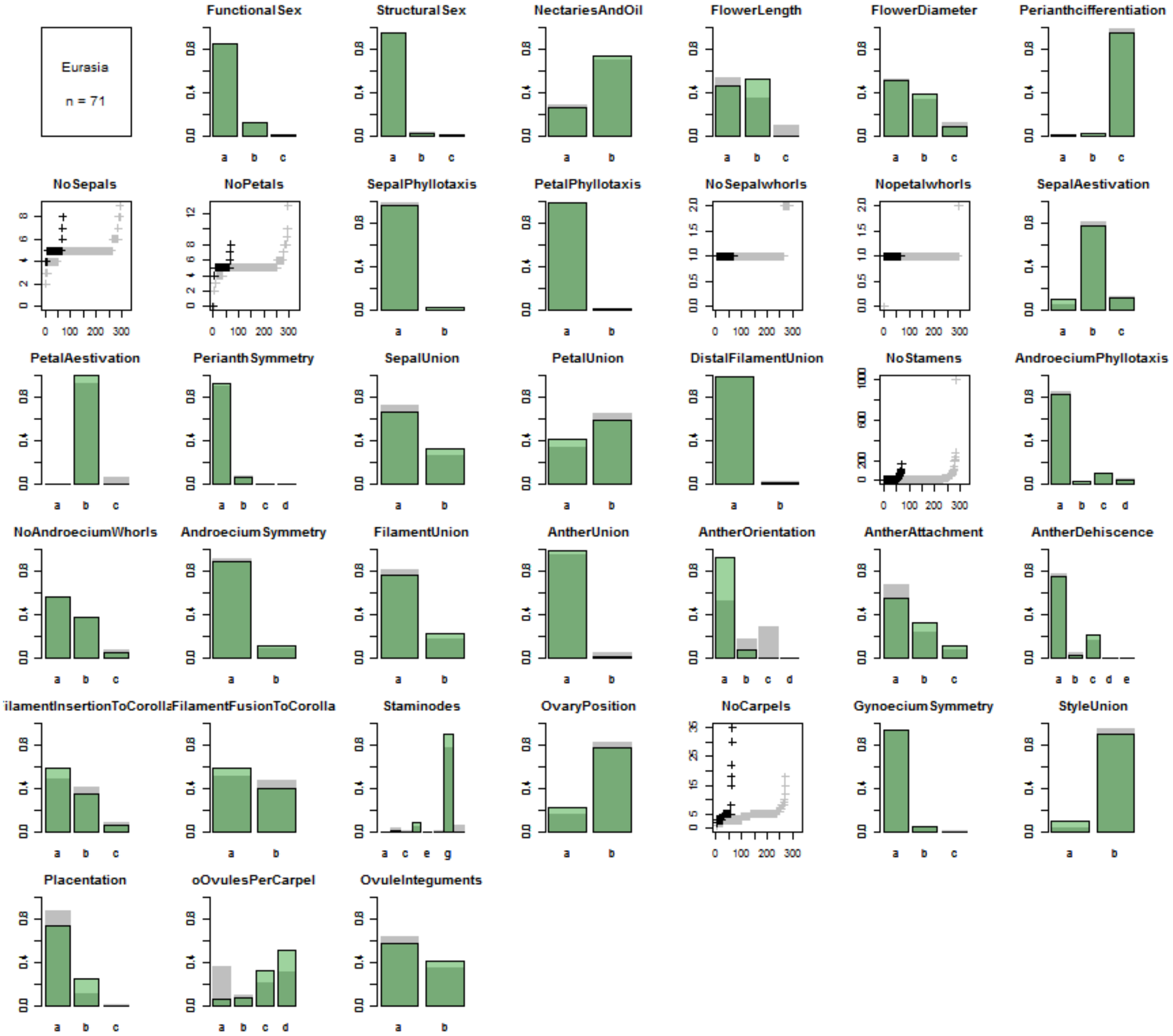
Eurasia
n = 71
FunctionalSex
StructuralSex
NectariesAndOil
FlowerLength
FlowerDiameter
Perianthcifferentiation
NoSepals
NoPetals
SepalPhyllotaxis
PetalPhyllotaxis
NoSepalwhorls
Nopetalwhorls
SepalAestivation
PetalAestivation
PerianthSymmetry
SepalUnion
PetalUnion
DistalFilamentUnion
NoStamens
AndroeciumPhyllotaxis
NoAndroeciumWhorls
AndroeciumSymmetry
FilamentUnion
AntherUnion
AntherOrientation
AntherAttachment
AntherDehiscence
ilamentInsertionToCorolla
FilamentFusionToCorolla
Staminodes
OvaryPosition
NoCarpels
GynoeciumSymmetry
StyleUnion
Placentation
oOvulesPerCarpel
OvuleIntegument

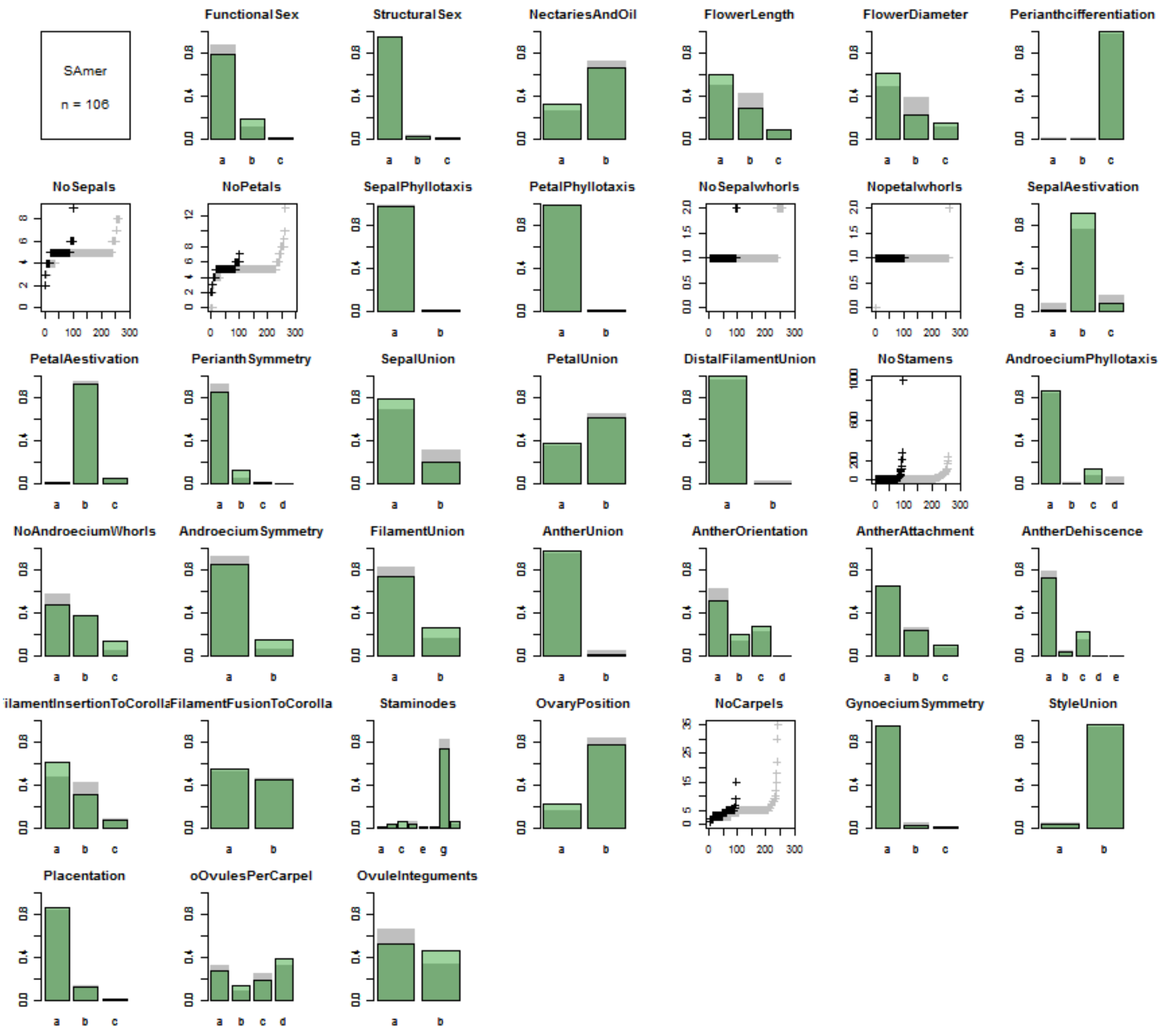
SAmer
n = 106
FunctionalSex
StructuralSex
NectariesAndOil
FlowerLength
FlowerDiameter
Perianthcifferentiation
NoSepals
NoPetals
SepalPhyllotaxis
PetalPhyllotaxis
NoSepalwhorls
Nopetalwhorls
SepalAestivation
PetalAestivation
PerianthSymmetry
SepalUnion
PetalUnion
DistalFilamentUnion
NoStamens
AndroeciumPhyllotaxis
NoAndroeciumWhorls
AndroeciumSymmetry
FilamentUnion
AntherUnion
AntherOrientation
AntherAttachment
AntherDehiscence
ilamentInsertionToCorolla
FilamentFusionToCorolla
Staminodes
OvaryPosition
NoCarpels
GynoeciumSymmetry
StyleUnion
Placentation
oOvulesPerCarpel
OvuleInteguments

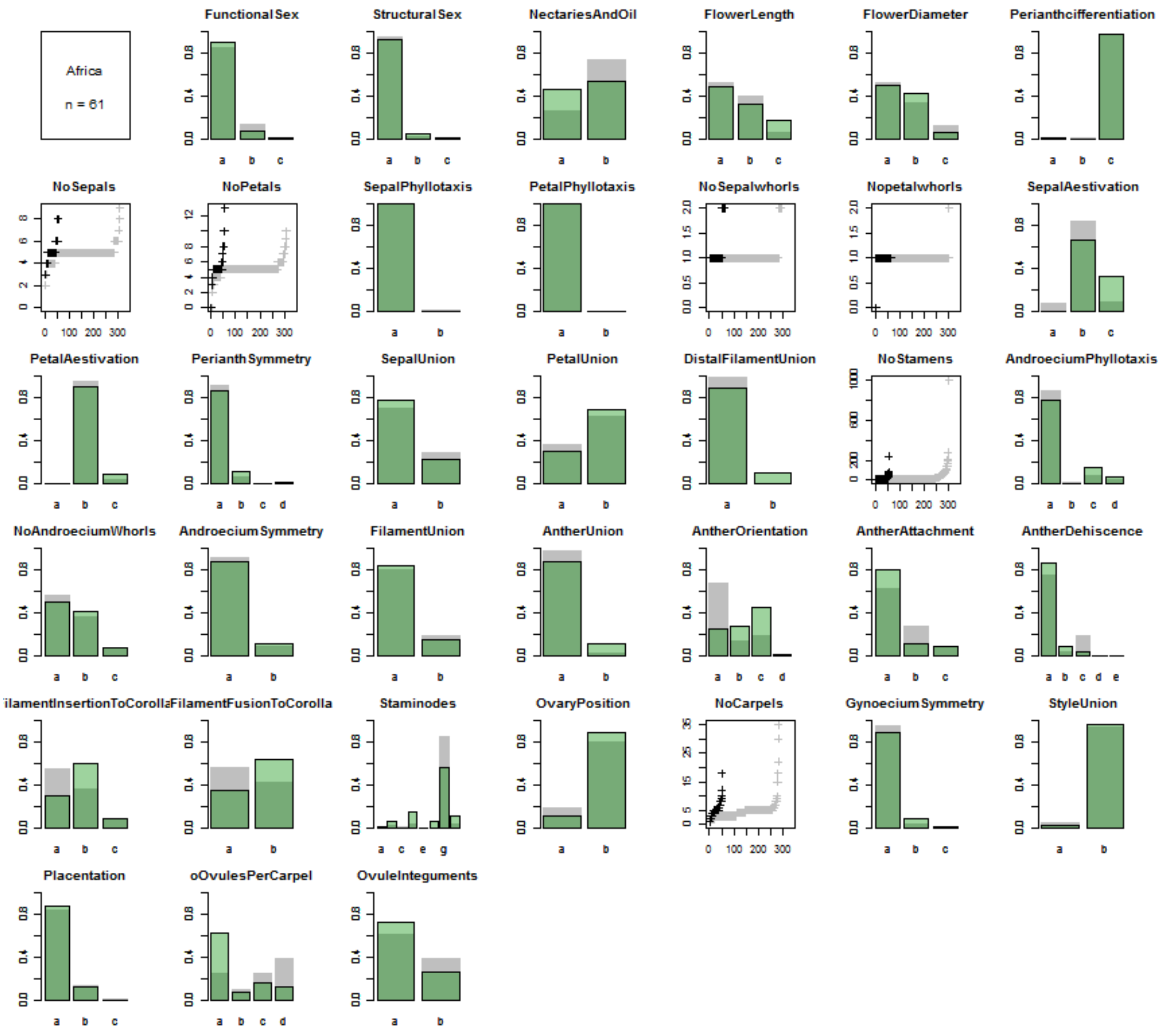
Africa
n = 61
FunctionalSex
StructuralSex
NectariesAndOil
FlowerLength
FlowerDiameter
Perianthcifferentiation
NoSepals
NoPetals
SepalPhyllotaxis
PetalPhyllotaxis
NoSepalwhorls
Nopetalwhorls
SepalAestivation
PetalAestivation
PerianthSymmetry
SepalUnion
PetalUnion
DistalFilamentUnion
NoStamens
AndroeciumPhyllotaxis
NoAndroeciumWhorls
AndroeciumSymmetry
FilamentUnion
AntherUnion
AntherOrientation
AntherAttachment
AntherDehiscence
ilamentInsertionToCorolla
FilamentFusionToCorolla
Staminodes
OvaryPosition
NoCarpels
GynoeciumSymmetry
StyleUnion
Placentation
oOvulesPerCarpel
OvuleIntegumentsv

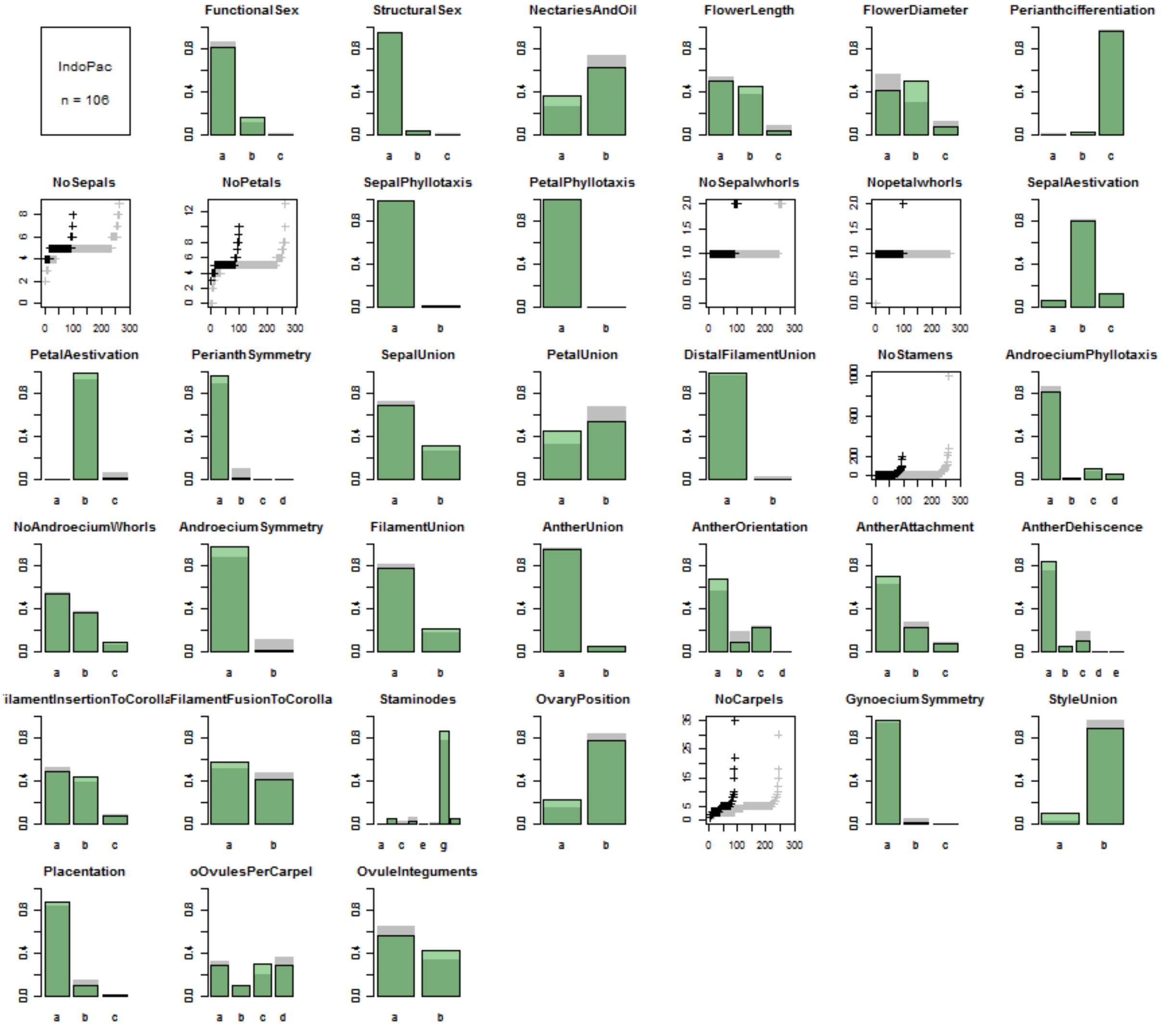
IndoPac
n = 106
FunctionalSex
StructuralSex
NectariesAndOil
FlowerLength
FlowerDiameter
Perianthcifferentiation
NoSepals
NoPetals
SepalPhyllotaxis
PetalPhyllotaxis
NoSepalwhorls
Nopetalwhorls
SepalAestivation
PetalAestivation
PerianthSymmetry
SepalUnion
PetalUnion
DistalFilamentUnion
NoStamens
AndroeciumPhyllotaxis
NoAndroeciumWhorls
AndroeciumSymmetry
FilamentUnion
AntherUnion
AntherOrientation
AntherAttachment
AntherDehiscence
ilamentInsertionToCorolla
FilamentFusionToCorolla
Staminodes
OvaryPosition
NoCarpels
GynoeciumSymmetry
StyleUnion
Placentation
oOvulesPerCarpel
OvuleInteguments

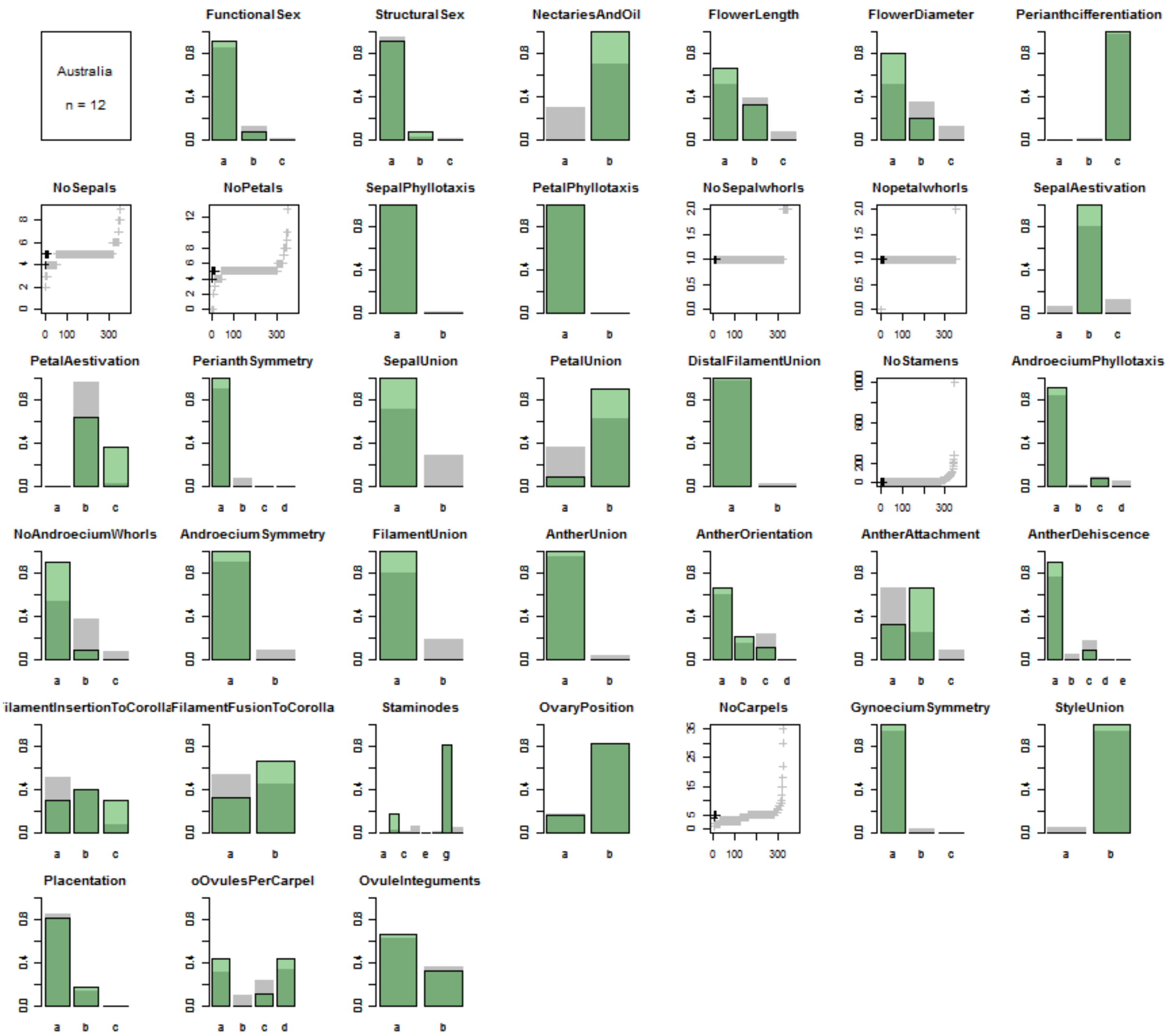
Australia
n = 12
FunctionalSex
StructuralSex
NectariesAndOil
FlowerLength
FlowerDiameter
Perianthcifferentiation
NoSepals
NoPetals
SepalPhyllotaxis
PetalPhyllotaxis
NoSepalwhorls
Nopetalwhorls
SepalAestivation
PetalAestivation
PerianthSymmetry
SepalUnion
PetalUnion
DistalFilamentUnion
NoStamens
AndroeciumPhyllotaxis
NoAndroeciumWhorls
AndroeciumSymmetry
FilamentUnion
AntherUnion
AntherOrientation
AntherAttachment
AntherDehiscence
ilamentInsertionToCorolla
FilamentFusionToCorolla
Staminodes
OvaryPosition
NoCarpels
GynoeciumSymmetry
StyleUnion
Placentation
oOvulesPerCarpel
OvuleInteguments

2.2. Visualisation of factor categories in the morphospace

In the following figures, the position in the floral morphospace of each factor category is highlighted by black dots. Gray dots represent the rest of the dataset. The following representation of the morphospace was obtained with a principal coordinate analysis, taking as input the original morphological dissimilarity matrix. The significance of the correlation between distances in the distance matrix and distances in the morphospace ordination presents the following statistics: Pearson's $r = 0.71$, $p < 0.001$. All details about the creation of the representation of this morphospace can be found in Chartier et al. (2017).

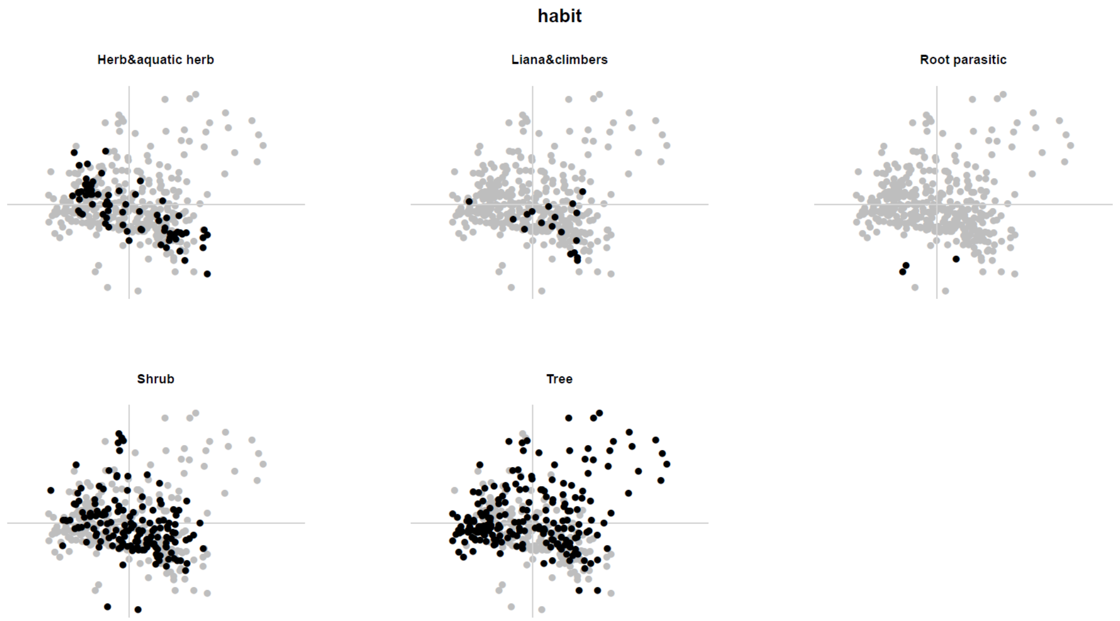

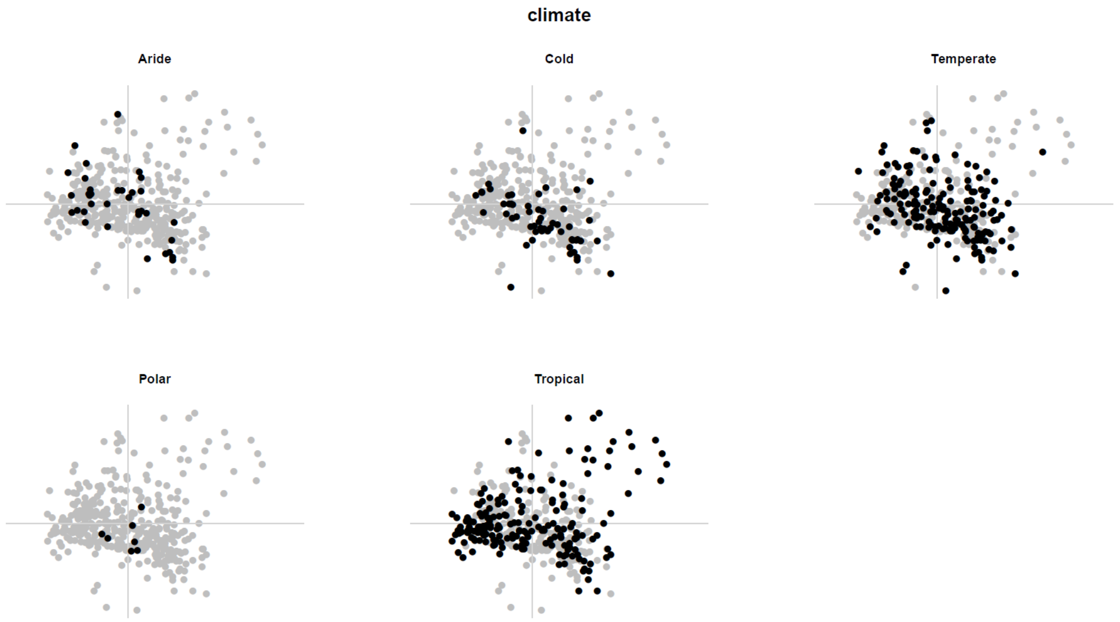

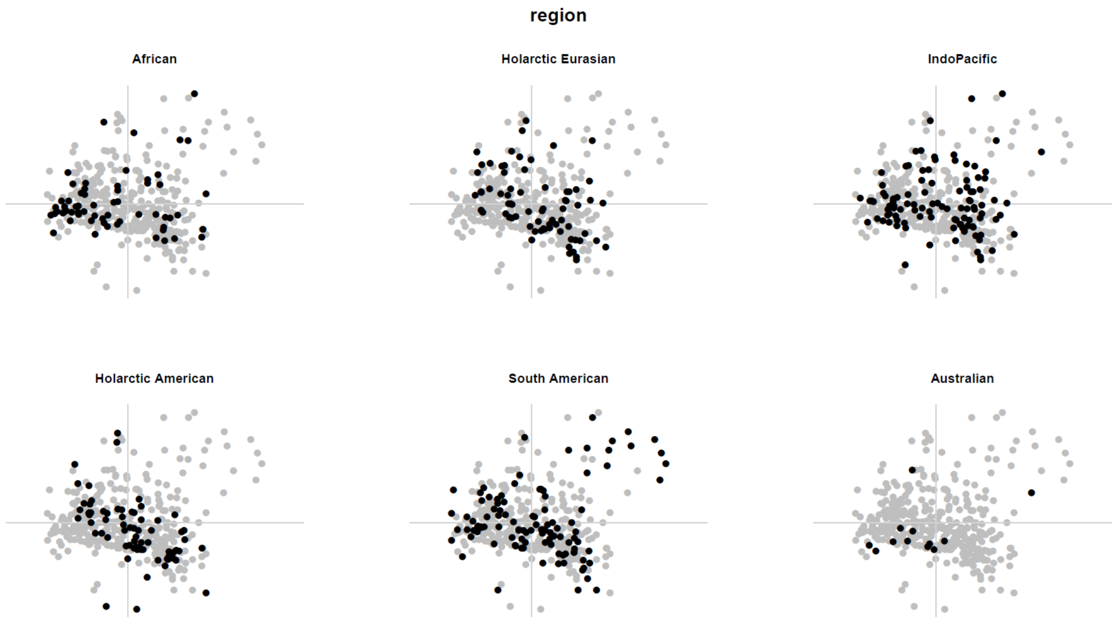
region
African
Holarctic Eurasian
IndoPacific
Holarctic American
South American
Australian

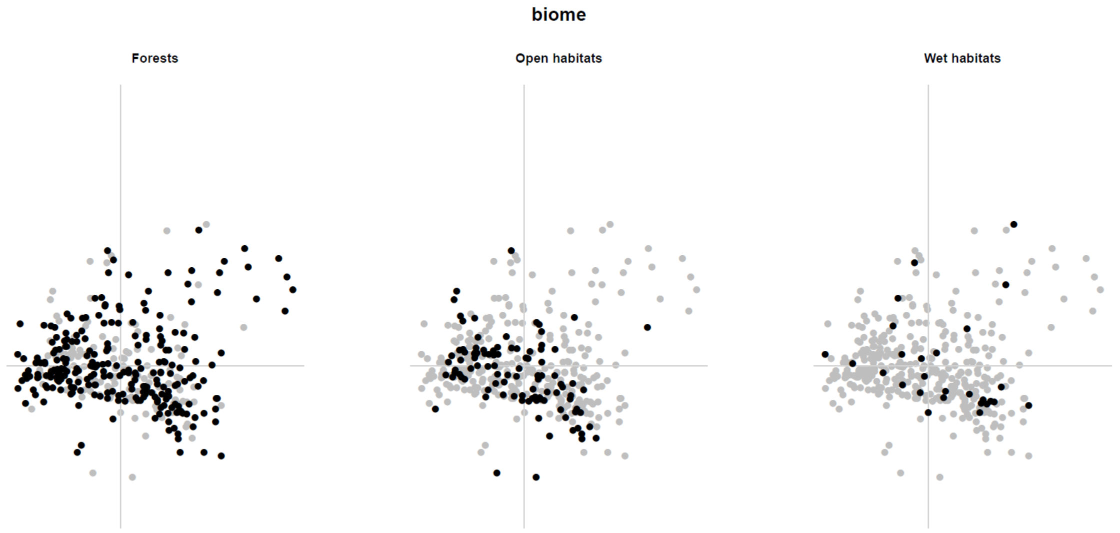
biome
Forests
Open habitats
Wet habitats

2.3. Variation of disparity when isolating factor categories.
Investigation of disparity variation among the different categories (e.g. *tree*, *shrub*, *herbs*) of one factor (e.g. *growth form*) for each category of the other factors.

In the following graphs: D = mean character difference between two taxa, $\overline{D}$ = mean pairwise differences for a group of taxa. For each boxplot, sample size is given below each box and disparity (the average $\overline{D}$ ± SD) is represented by orange circles and orange error bars. Post hoc test results are summarized in red. When the overall Kruskal-Wallis test was significant ($p \leq 0.5$), letters indicate groups that are not significantly different according to posthoc tests. NS means that the overall test was not significant ($p > 0.05$) and therefore no posthoc test was made.

Note that sample size is given as number of species, but that disparity ($\overline{D}$) is calculated for pairs of species. This means that, for n species, disparity is calculated by averaging $(n^2 - n) \div 2$ values.

Growth form

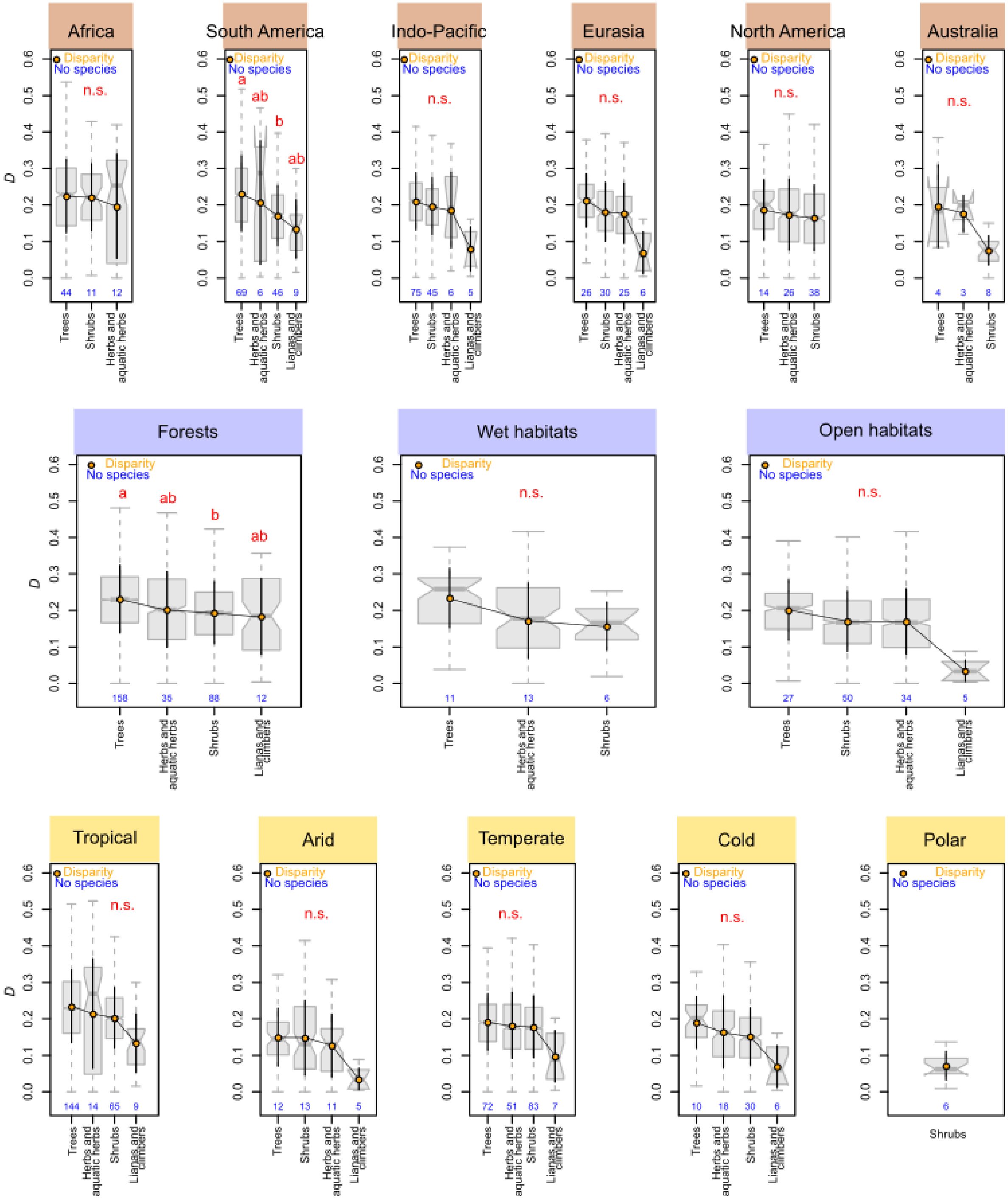
Africa
South America
Indo-Pacific
Eurasia
North America
Australia
Forests
Wet habitats
Open habitats
Tropical
Arid
Temperate
Cold
Polar
Disparity
No species
n.s.
a
ab
b
D
Trees
Shrubs
Herbs and aquatic herbs
Lianas and climbers

Habitat

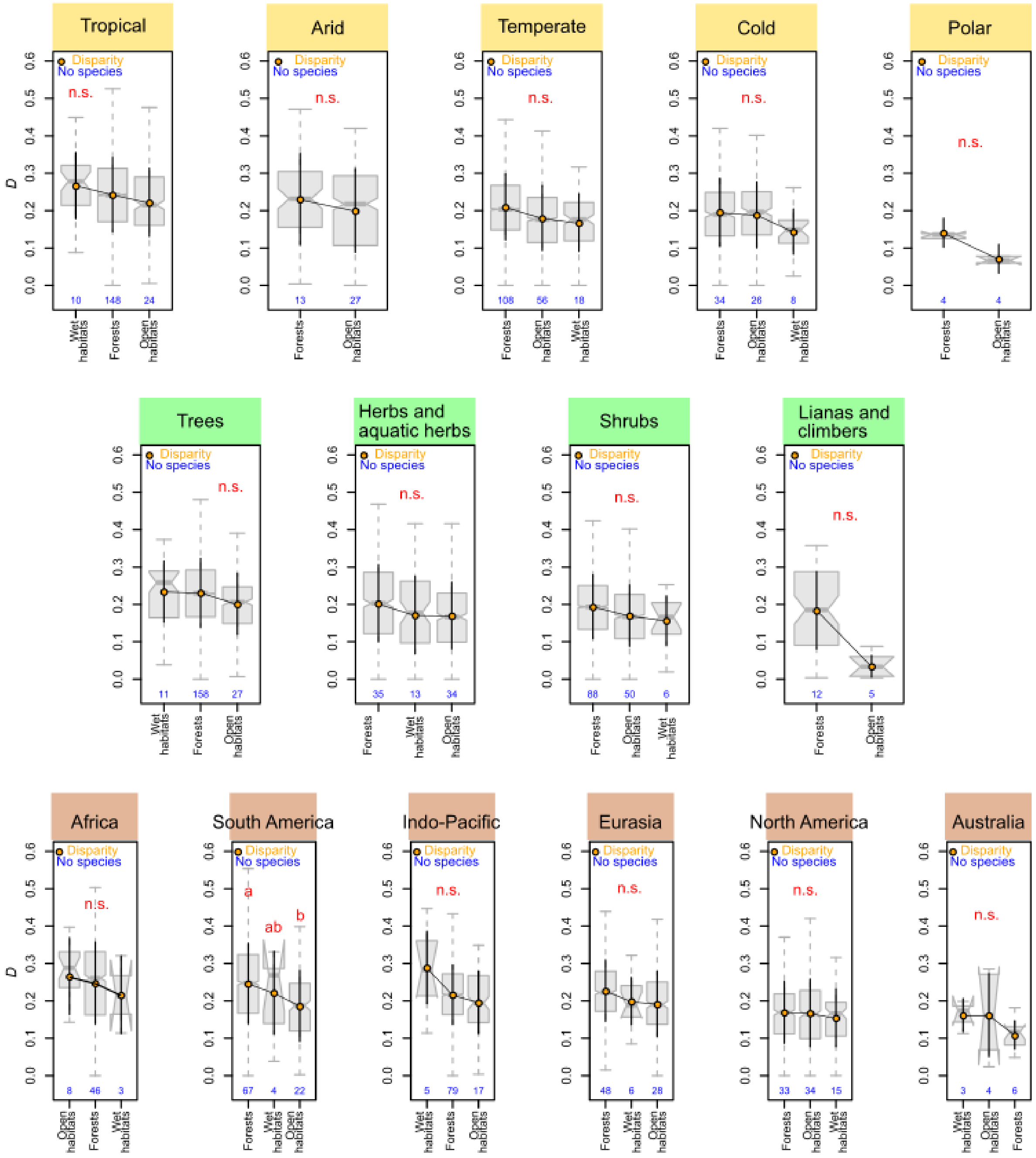

Tropical
Arid
Temperate
Cold
Polar
Trees
Herbs and aquatic herbs
Shrubs
Lianas and climbers
Africa
South America
Indo-Pacific
Eurasia
North America
Australia
Disparity
No species
D
n.s.
a
ab
b
0.0
0.1
0.2
0.3
0.4
0.5
0.6
Wet habitats
Forests
Open habitats
10 148 24
13 27
108 56 18
34 28 8
4 4
11 158 27
35 13 34
88 50 6
12 5
8 46 3
67 4 22
5 79 17
48 6 28
33 34 15
3 4 6

Climate

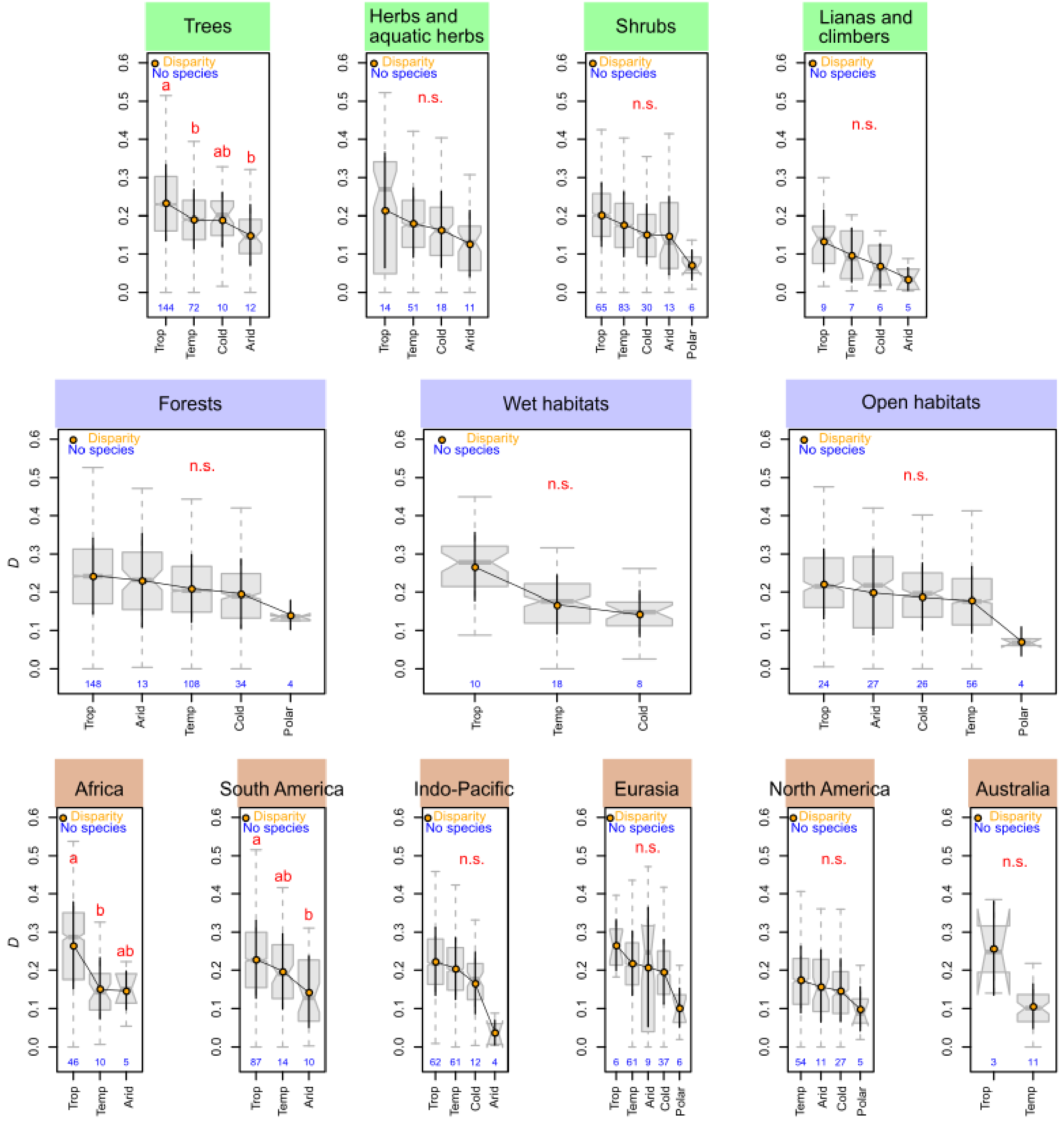

Trees
Herbs and aquatic herbs
Shrubs
Lianas and climbers
Forests
Wet habitats
Open habitats
Africa
South America
Indo-Pacific
Eurasia
North America
Australia
Disparity
No species
D
n.s.
Trop
Temp
Cold
Arid
Polar

Region

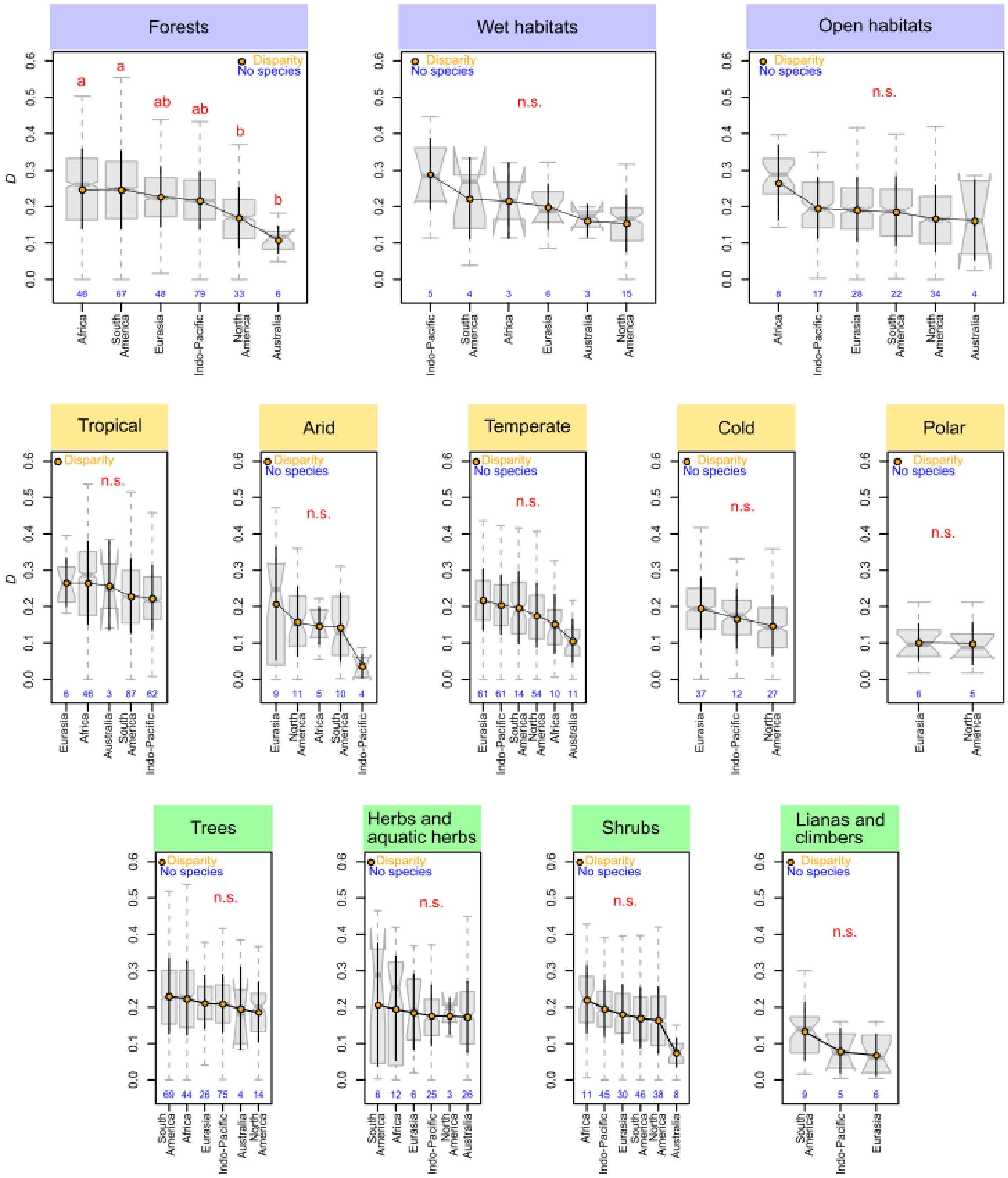
Forests
Wet habitats
Open habitats
Tropical
Arid
Temperate
Cold
Polar
Trees
Herbs and aquatic herbs
Shrubs
Lianas and climbers
Disparity
No species
D
a
a
ab
ab
b
b
n.s.
Africa
South America
Eurasia
Indo-Pacific
North America
Australia

2.4. Permutation test (latitudinal disparity gradients of disparity vs. species richness)

In this analysis, we show that the higher disparity found at low latitudes is not due to the fact that there are more species in the tropics, but that it is rather due to the fact that these tropical species are morphologically more diverse.

To test this, we randomized floral morphologies among species 10,000 times, and recalculated disparity for each latitudinal interval in each case (each 10° latitudinal interval containing the real number of species, but the morphologies of these species being randomly mixed without replacement).

If the higher disparity at low latitudes is not due to the higher number of species in these areas, we expect that once the morphologies are randomized, disparity gets homogenized across all latitudes and there is no more disparity gradient even if a species number gradient is still present. In this case, the distribution of *r* (the coefficients of correlation of a Pearson correlation test between latitude and disparity), should follow a normal distribution centered to zero (0 is the value of *r* in the case of no correlation).

If, on the contrary, if the higher disparity at low latitudes is due to the fact that there are more species at low latitudes, we expect a negative correlation between latitude and disparity in all cases, and the distribution of *r* should be asymmetrical towards -1 (-1 is the value of *r* in the case of a perfect negative correlation).

The distribution of *r* once the data are randomized is shown below. It is centered to 0, and in 99% cases, *r* is higher than the value or *r* (-0.77) in the unaltered dataset. This shows that, in our sampling, the higher disparity at lower altitudes is not an effect of higher species numbers in these areas.

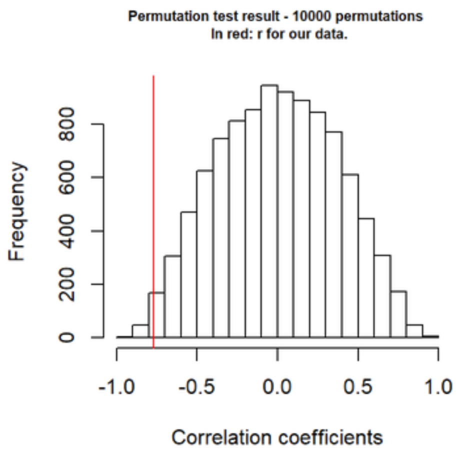


```
## [1] "Proportion of cases where r is inferior or equal to r in our data (p-value of this permutation test):
0.008"
```

2.5. Latitudinal distribution of all Ericales from GBIF.

The graph below shows the latitudinal distribution of Ericales species when taking into account all species available in GBIF (n=7152, gray area). The red line shows the distribution of the uncorrected GBIF data for the species sampled in this study (n=347). The blue line shows the distribution for the species sampled for this study after manually correcting the raw GBIF distribution data to only keep native distribution areas.

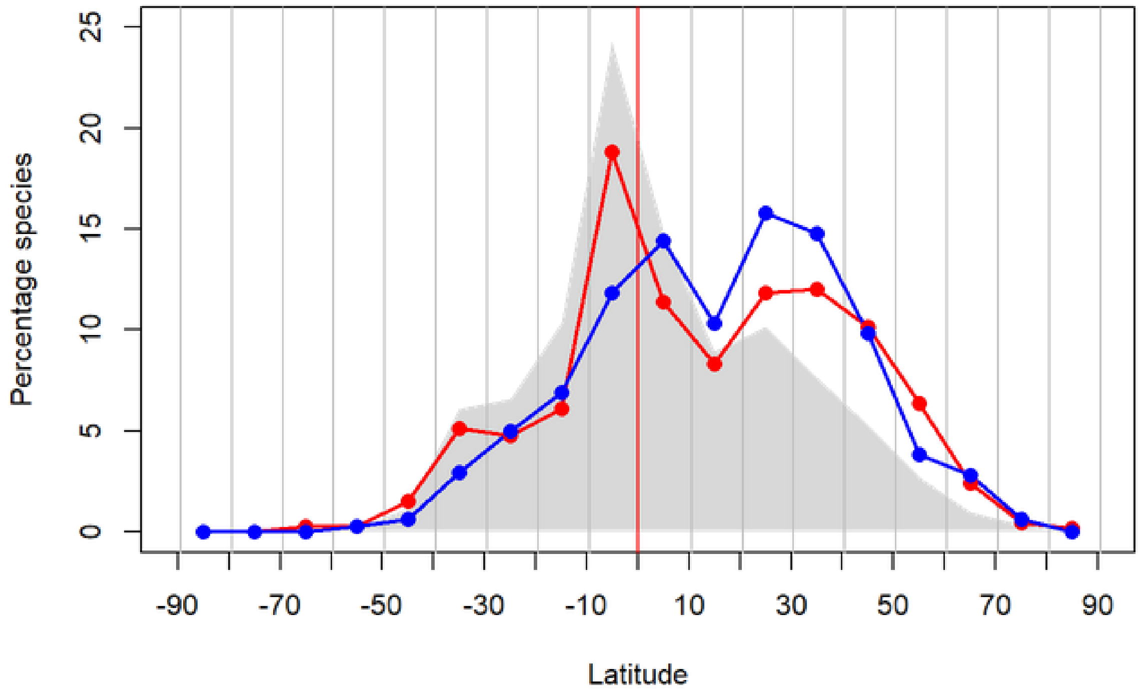

There are data for 7,152 ericalean species in GBIF, c.a. 65% of the order. A discrepancy between the distribution of the full Ericales dataset extracted from GBIF (gray area on the graph above) and our data without correction (in red, 347 species) is mainly present in the Northern hemisphere with more species in our sample compared to the total Ericales recorded in GBIF between latitudes 20 and 60. Apart from that difference, the shapes of the two curves are similar, and both show a latitudinal gradient. The shape of the curve changes significantly once the data is manually corrected (blue curve).

3. Note S2 - Distribution maps of the study species

Manually corrected distribution of the study species. Original occurence data were extracted from the database GBIF, and distributions manually corrected using floras and other scientific litterature (references are stored in the database Proteus (http://eflower.myspecies.info/)).

Acanthogilia gloriosa

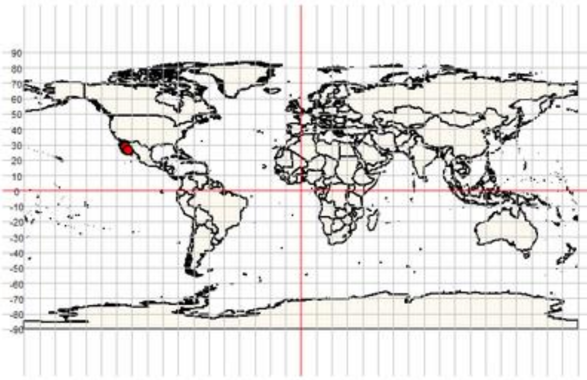

Acrostemon hirsutus

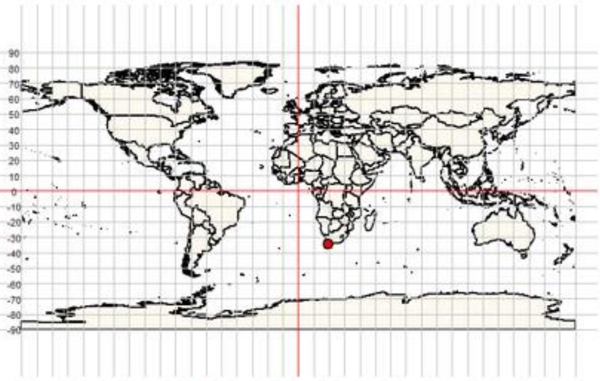

Acrotriche divaricata

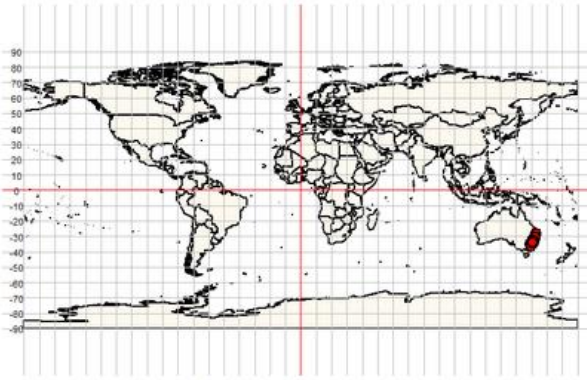

Actinidia arguta

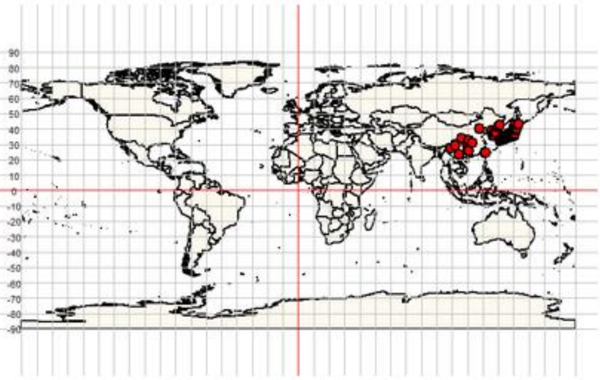

Actinidia callosa

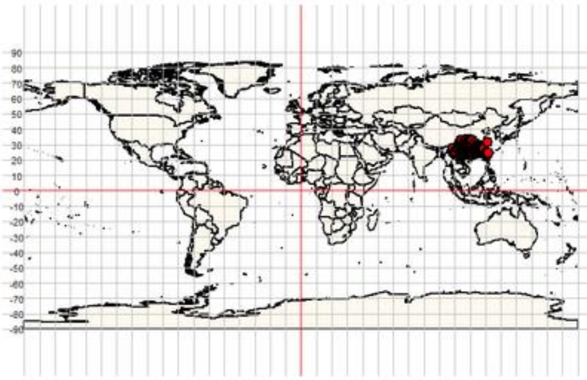

Actinidia chinensis

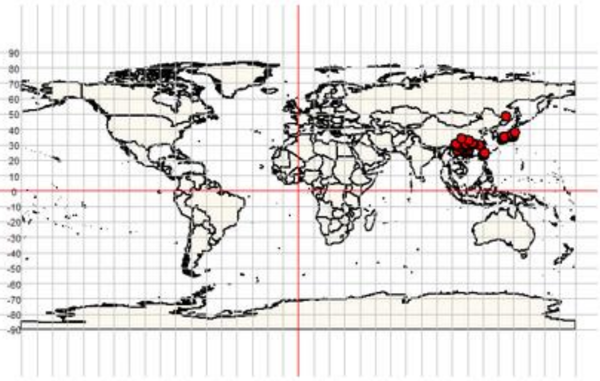

Actinidia kolomikta

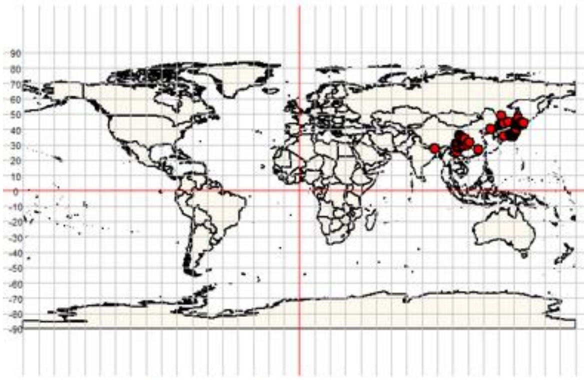

Actinidia melanandra

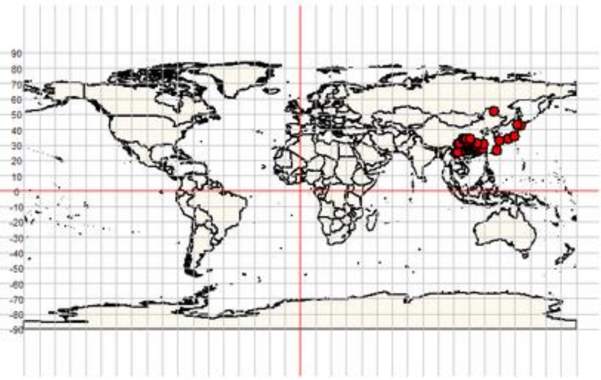

Adinandra formosana

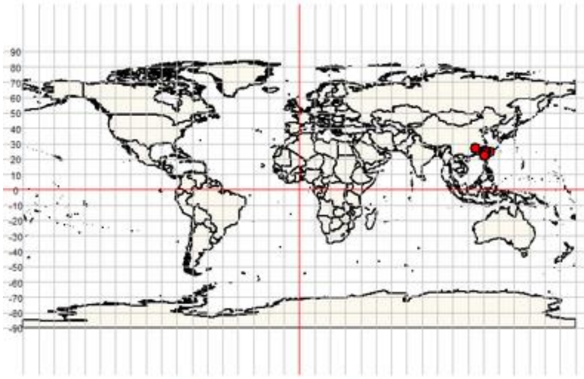

Agapetes serpens

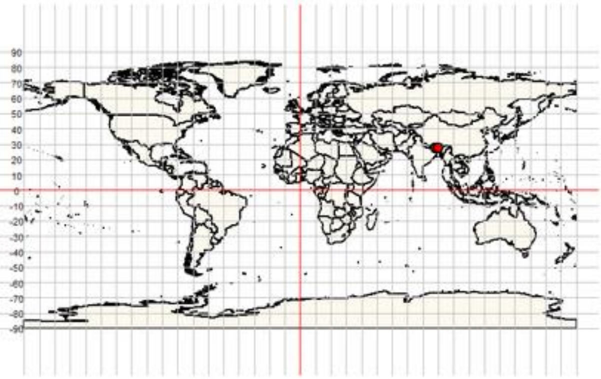

Agarista albiflora

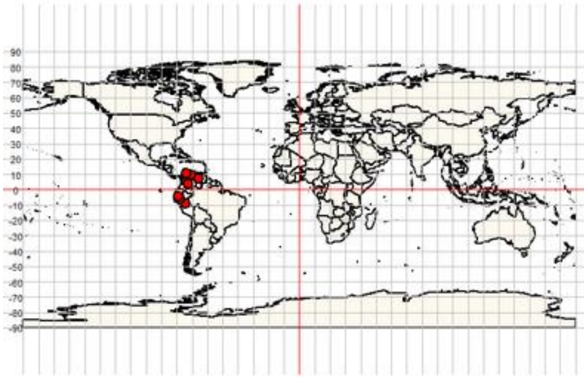

Agarista sleumeri

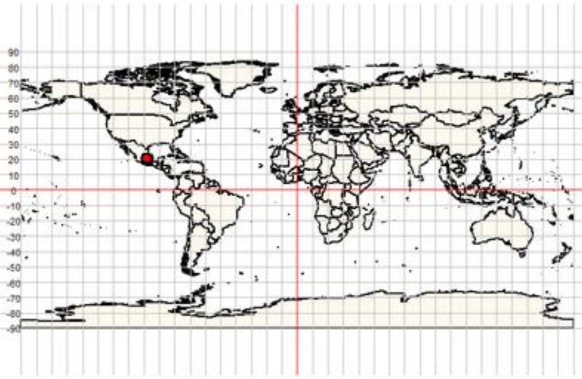

Aliciella latifolia

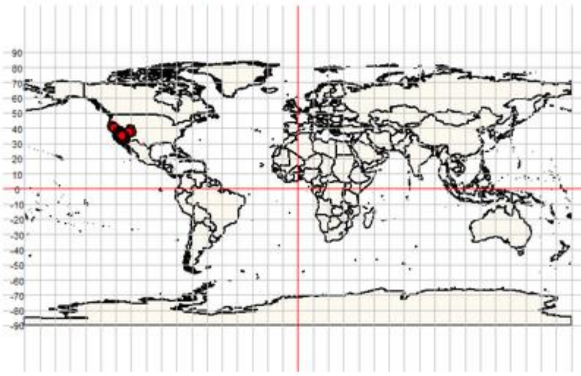

Allantoma lineata

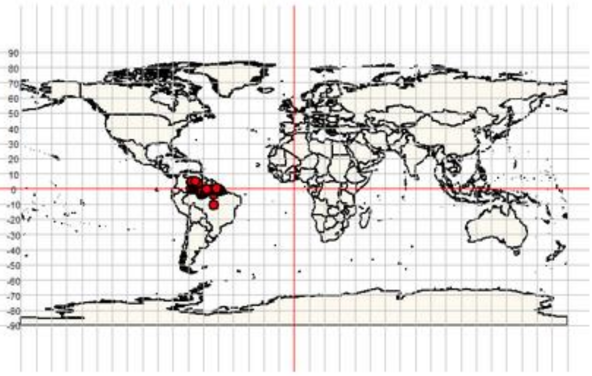

Allophyllum divaricatum

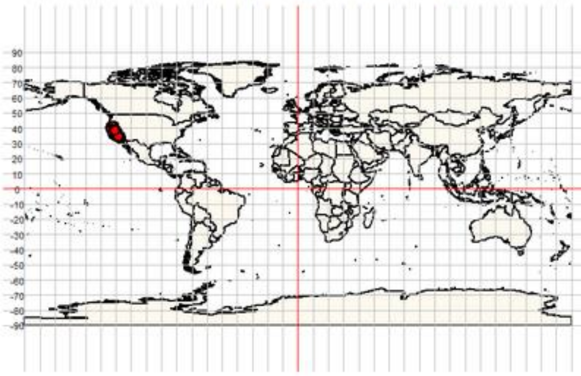

Allotropa virgata

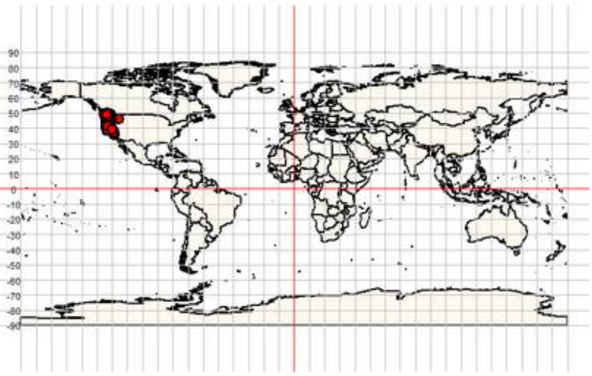

Alniphyllum fortunei

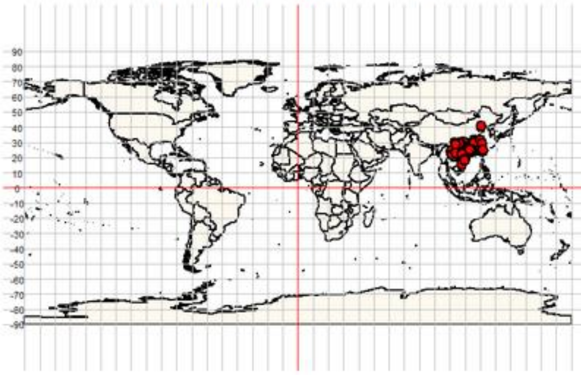

Amblyanthopsis membranacea

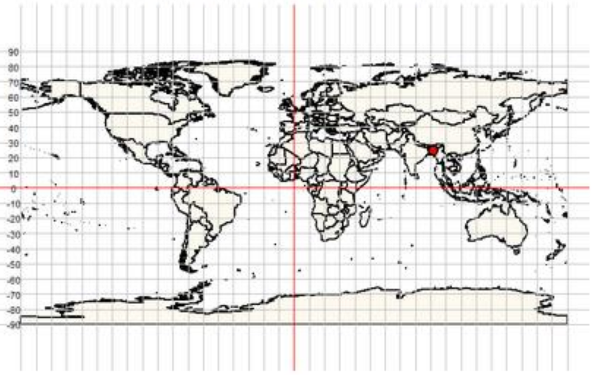

Amblyanthus glandulosus

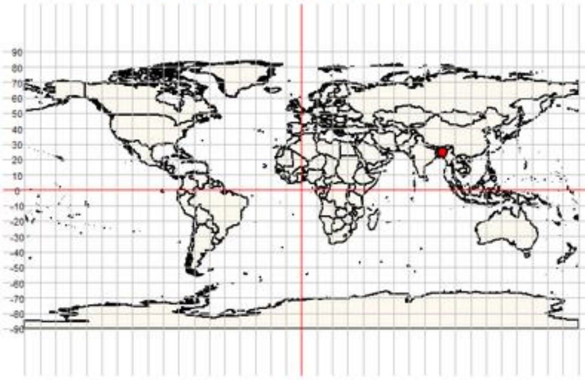

Anagallis arvensis

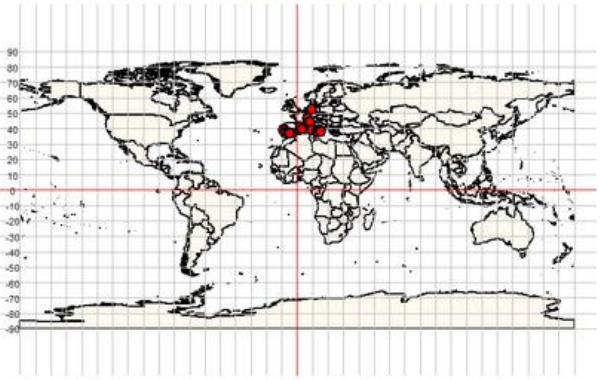

Anagallis minima

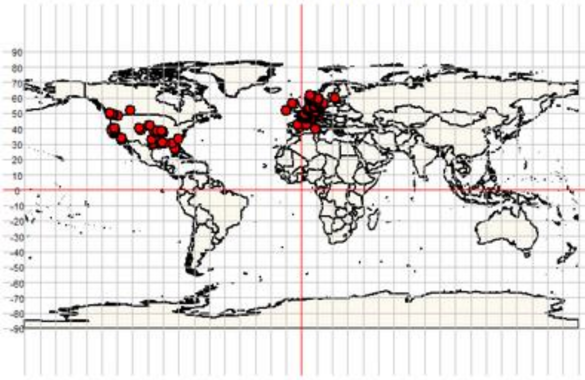

Andersonia brevifolia

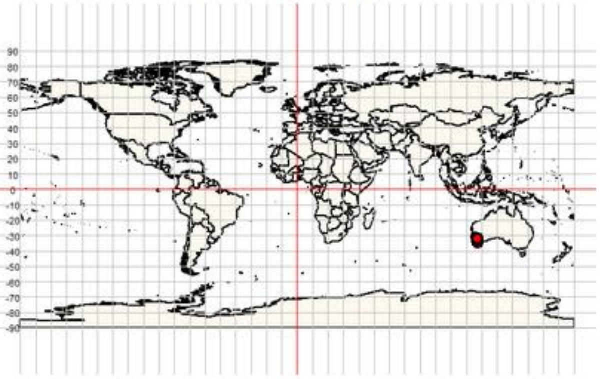

Andromeda polifolia

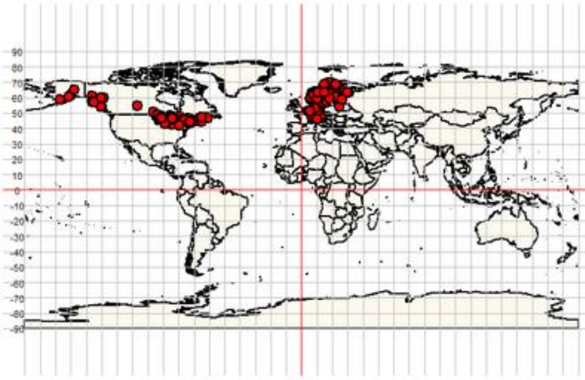

Androsace maxima

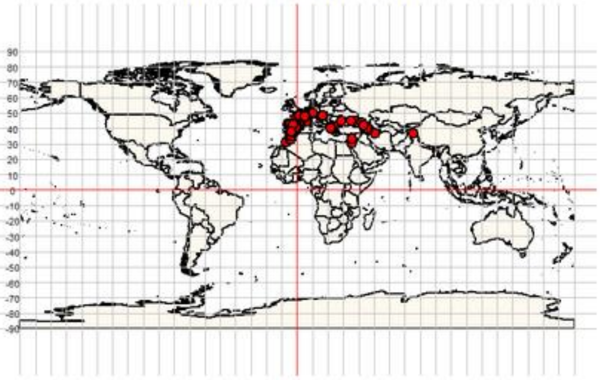

Anneslea fragrans

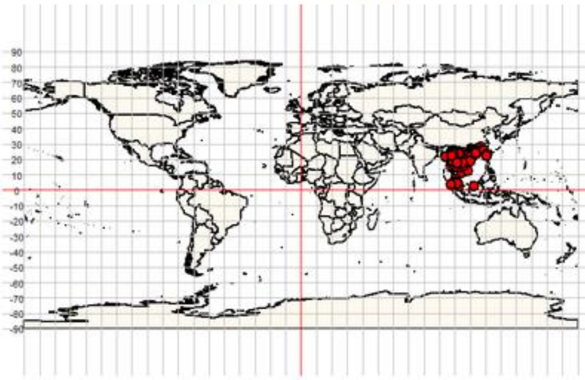

Anomalanthus marlothii

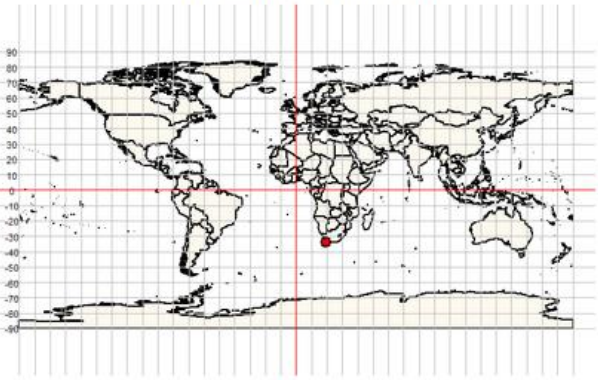

Apterosperma oblata

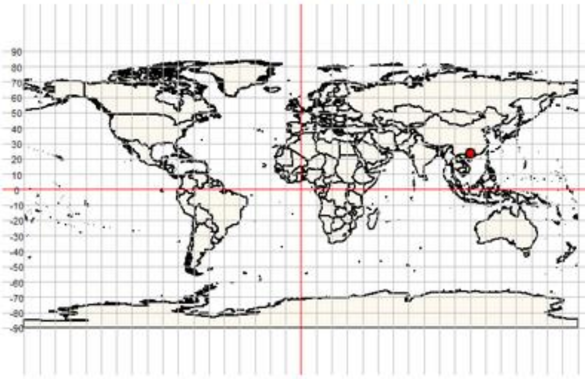

Arbutus menziesii

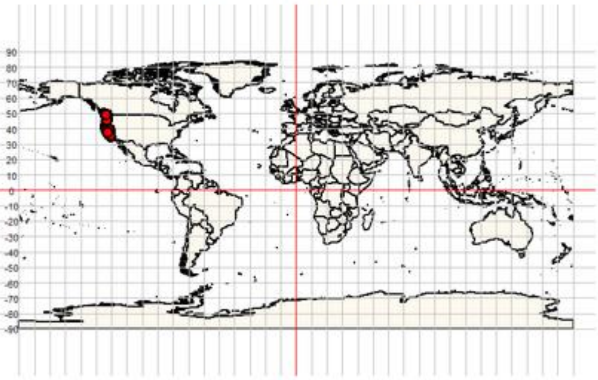

Archboldiodendron calosericeum

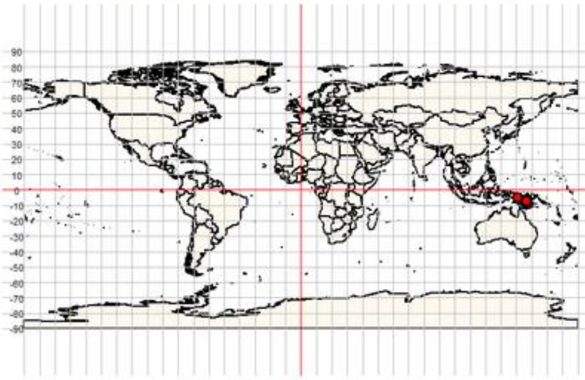

Ardisia calycosa

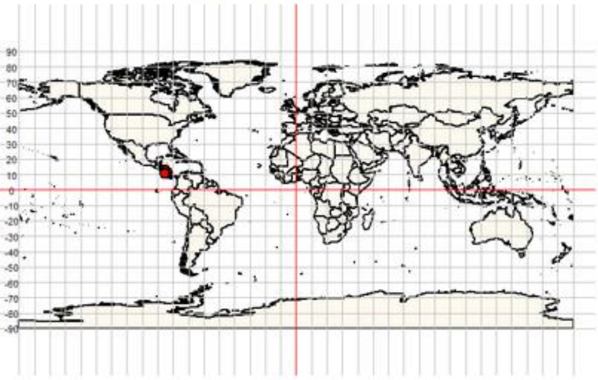

Ardisiandra primuloides

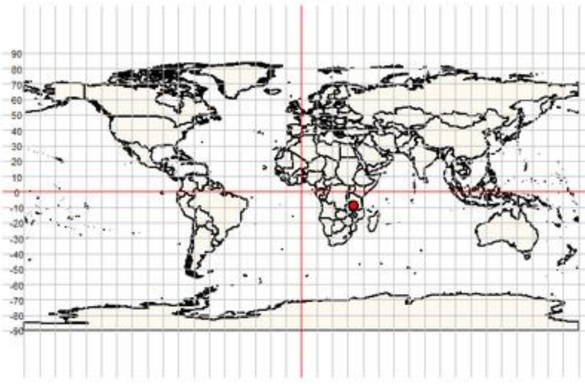

Argania spinosa

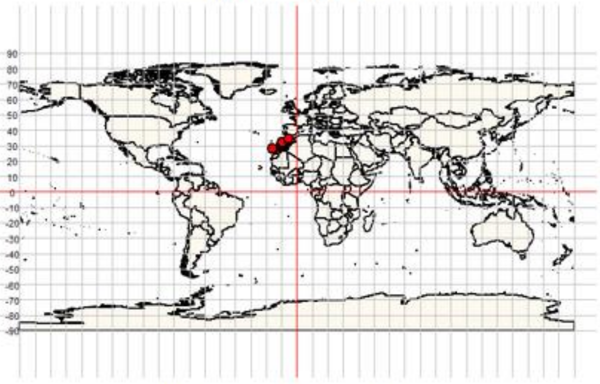

Asteranthos brasiliensis

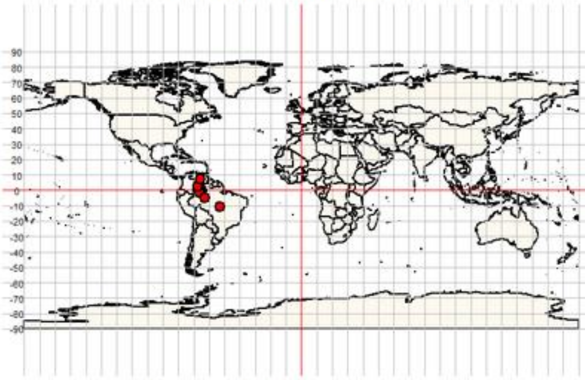

Aubregrinia taiensis

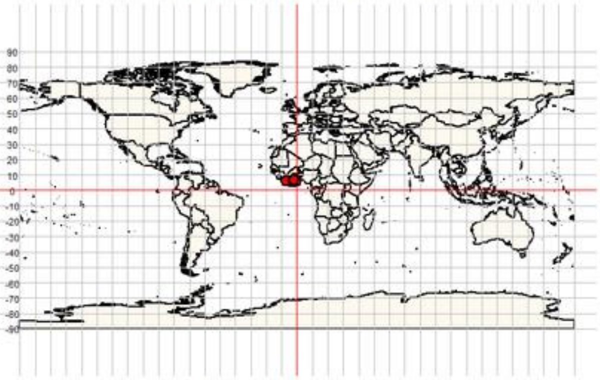

Autranella congolensis

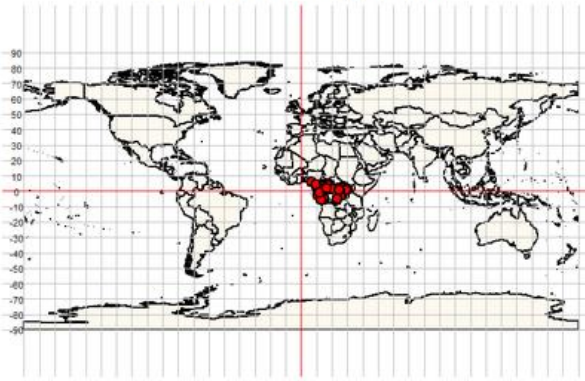

Baillonella toxisperma

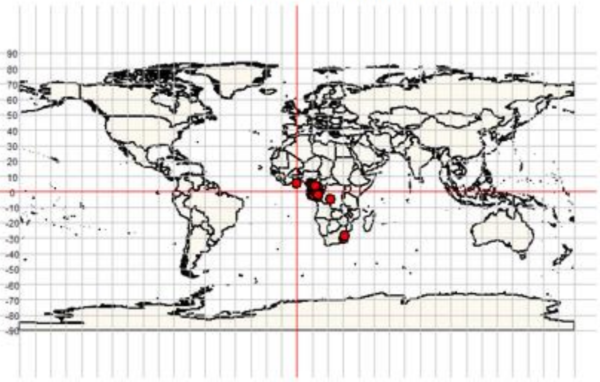

Barringtonia asiatica

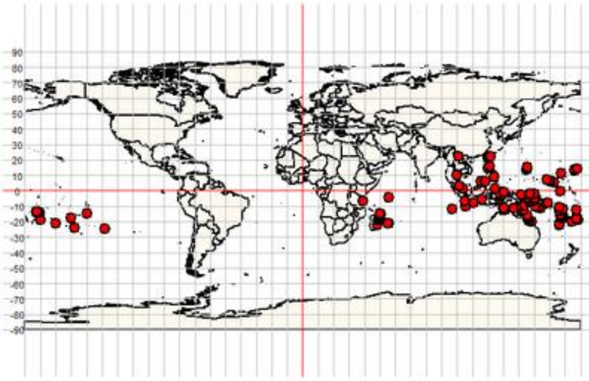

Barringtonia racemosa

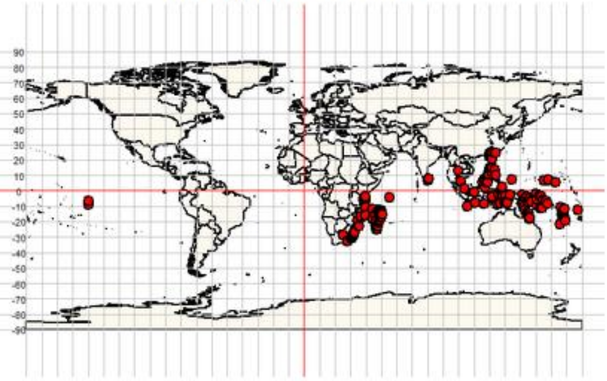

Beccariella sebertii

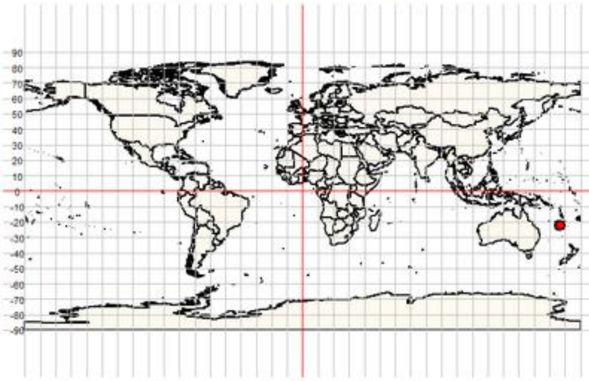

Bejaria racemosa

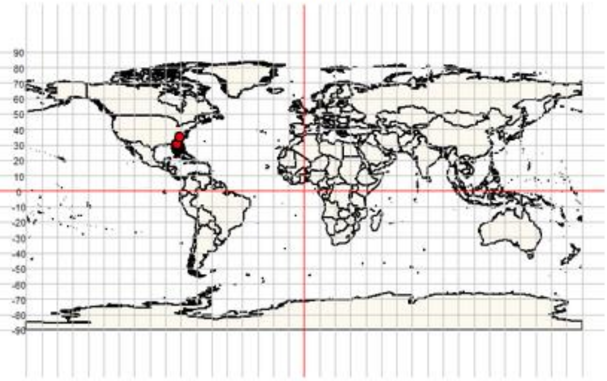

Berneuxia thibetica

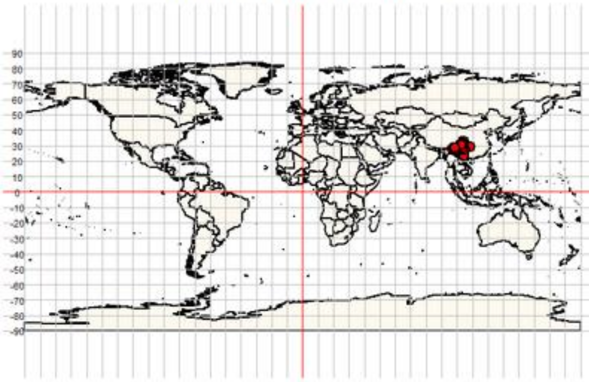

Bertholletia excelsa

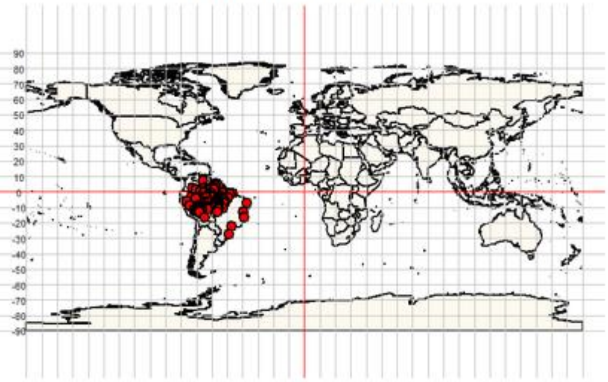

Bonellia albiflora

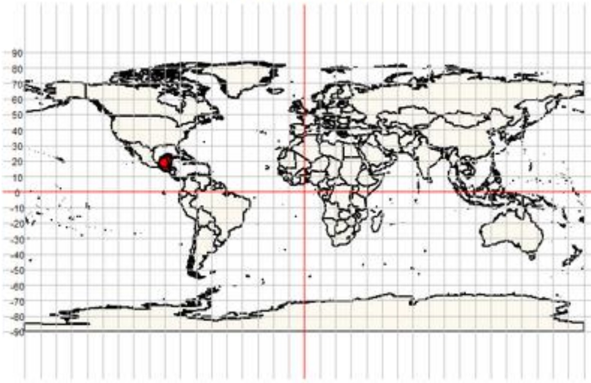

Bonplandia geminiflora

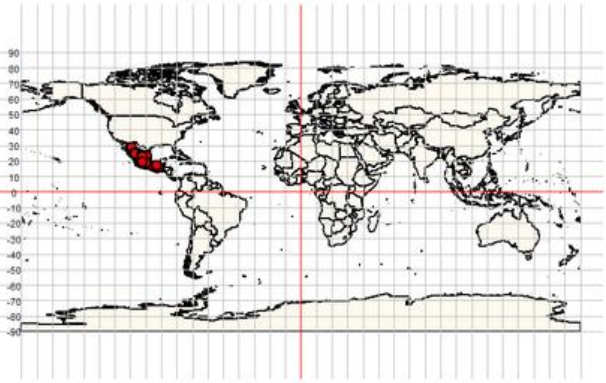

Breviea sericea

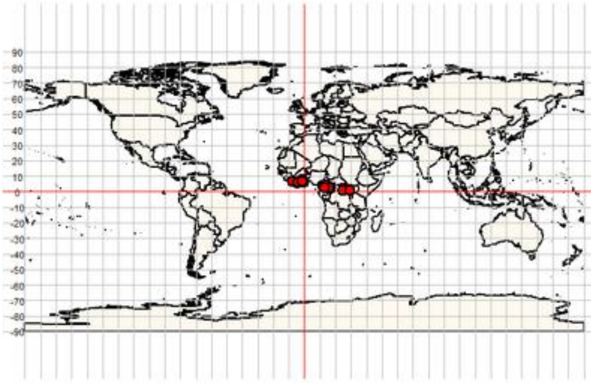

Bruinsmia styracoides

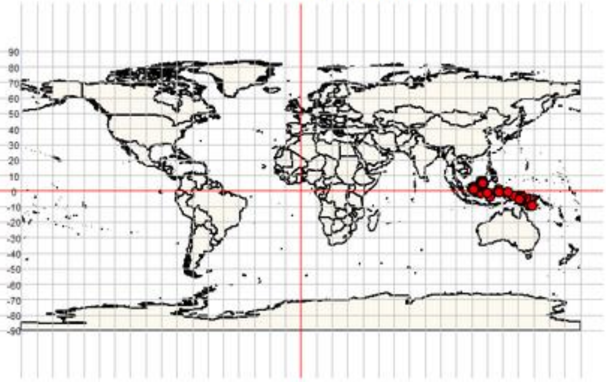

Bryantiella palmeri

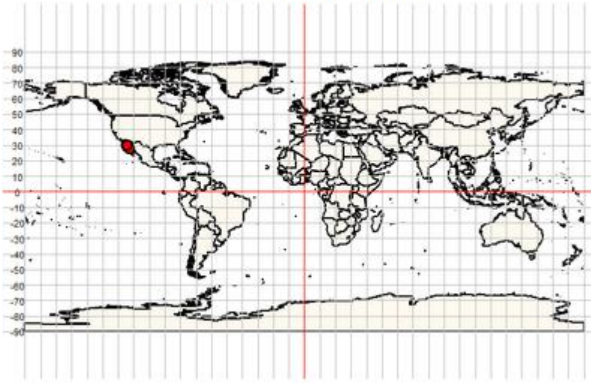

Bryocarpum himalaicum

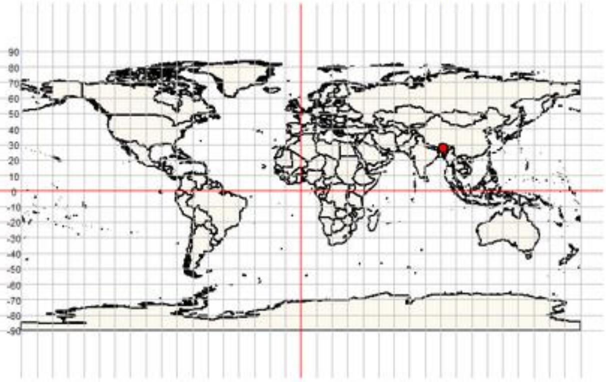

Calluna vulgaris

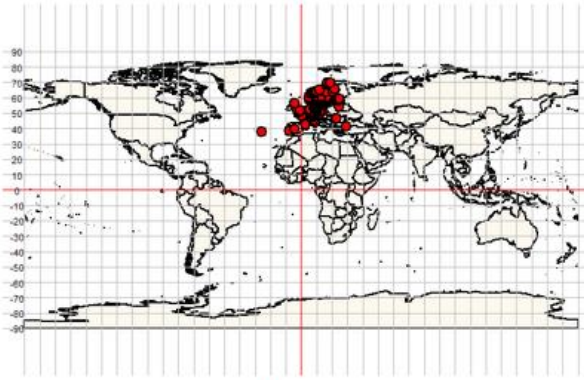

Camellia japonica

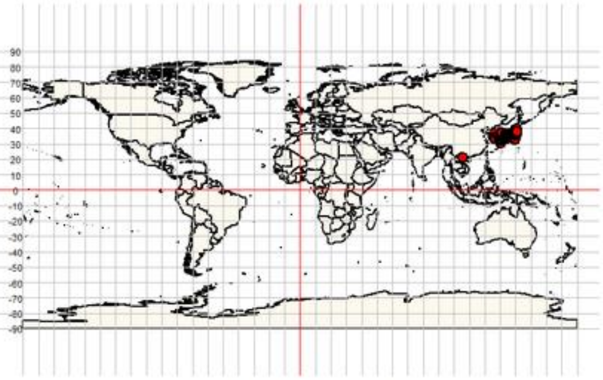

Camellia sinensis

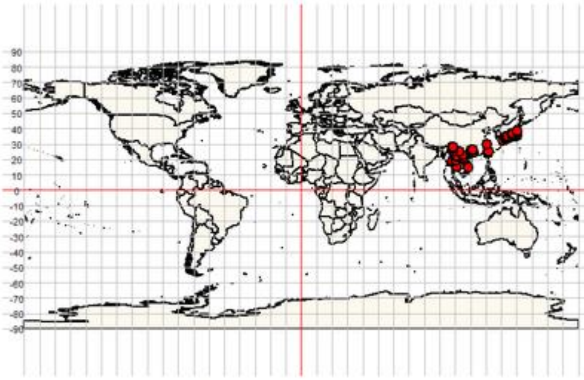

Cantua buxifolia

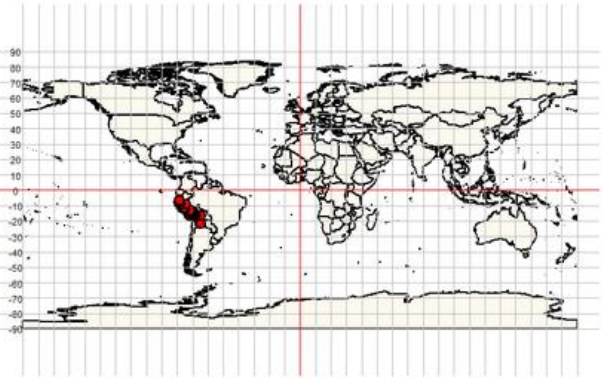

Cantua flexuosa

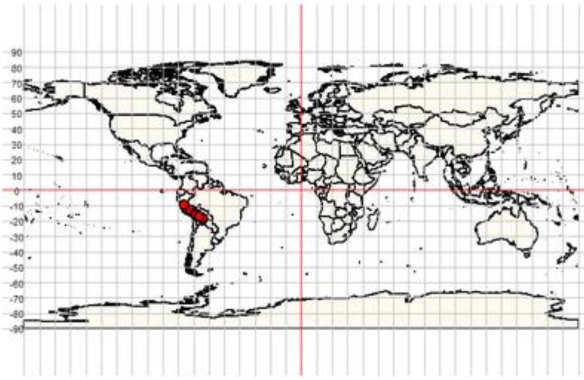

Capurodendron nodosum

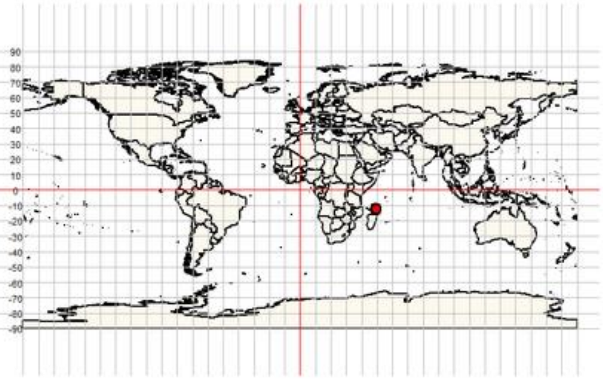

Careya arborea

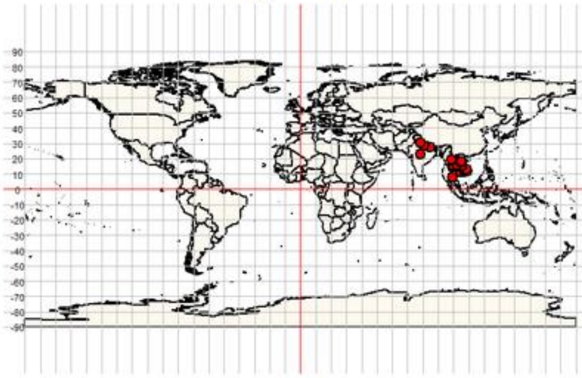

Cariniana micrantha

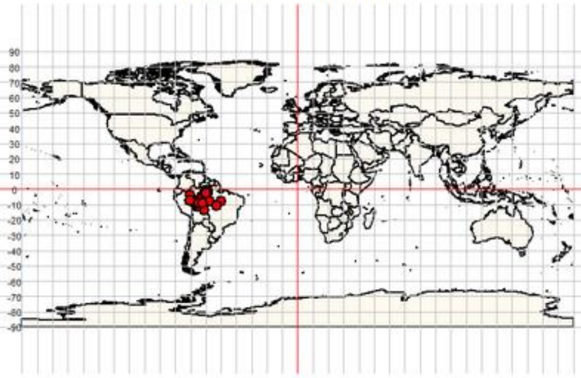

Cassiope mertensiana

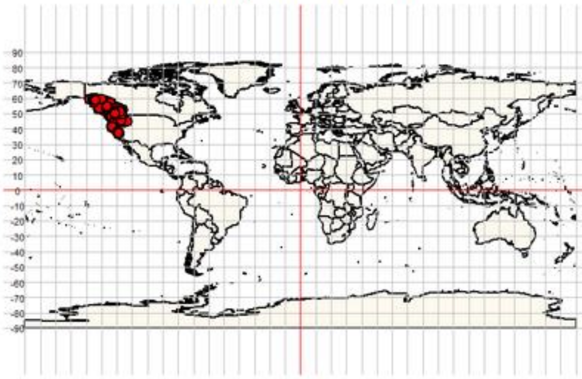

Ceratiola ericoides

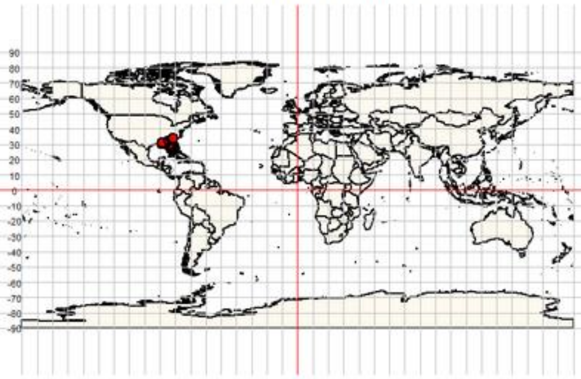

Chamaedaphne calyculata

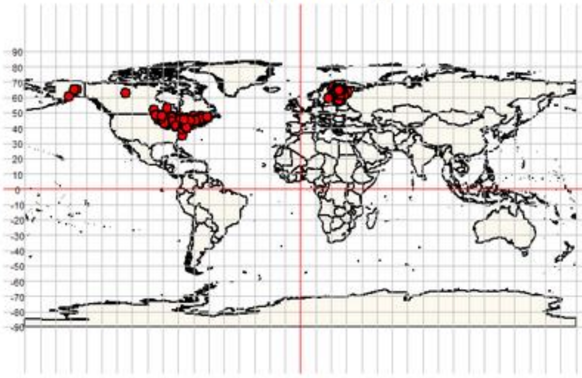

Changiostyrax dolichocarpus

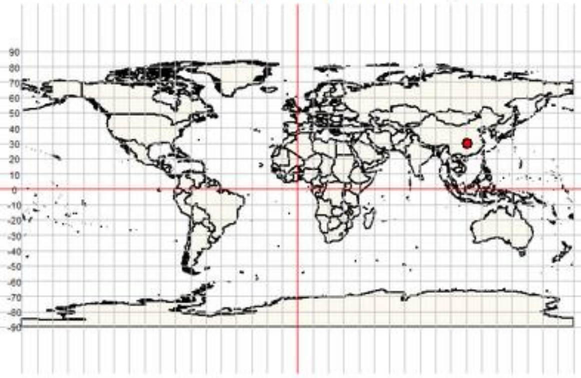

Chimaphila umbellata

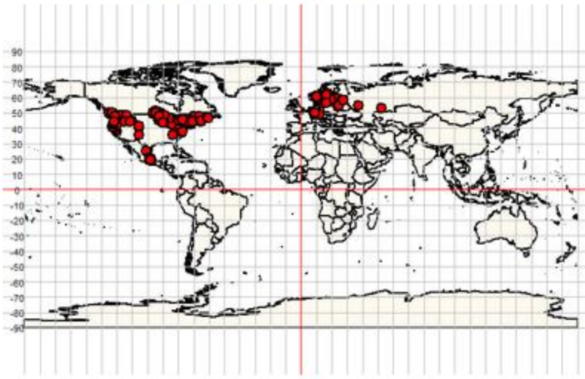

Chromolucuma rubriflora

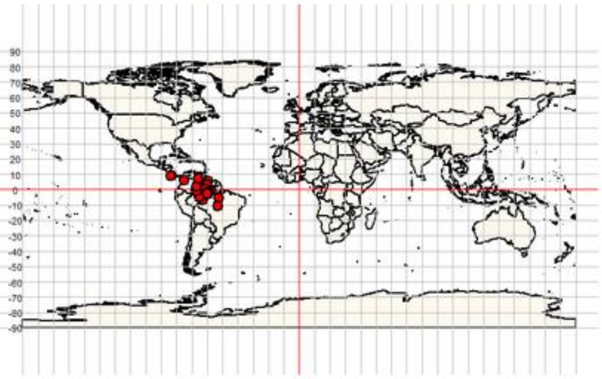

Chrysophyllum flexuosum

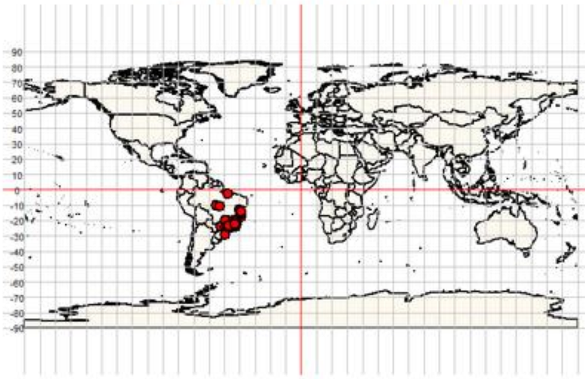

Chydenanthus excelsus

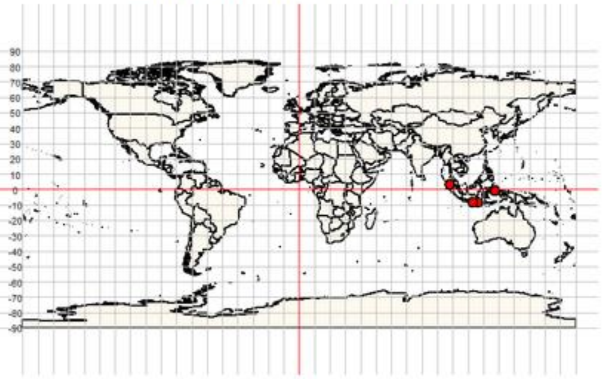

Clavija eggersiana

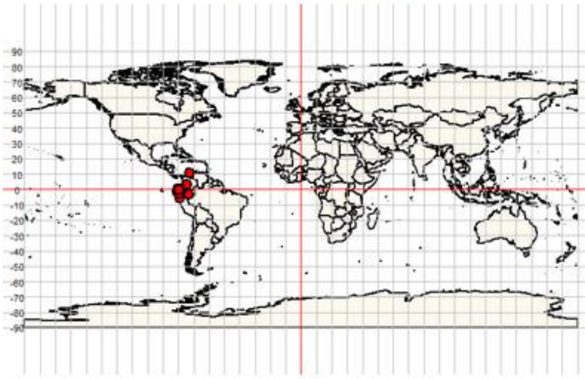

Clematoclethra scandens

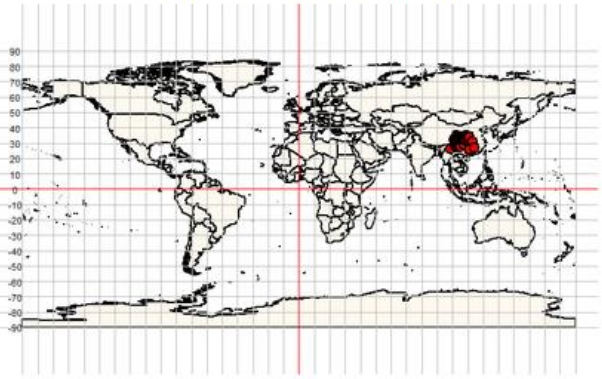

Clethra alnifolia

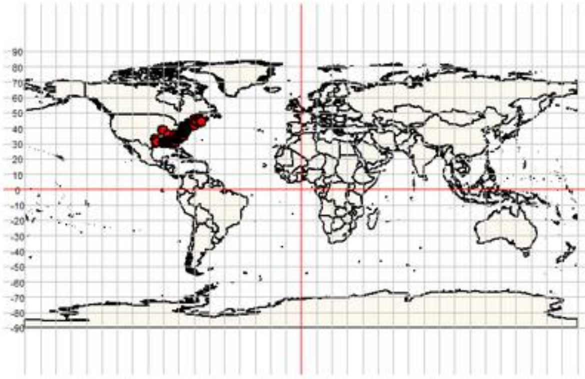

Clethra arborea

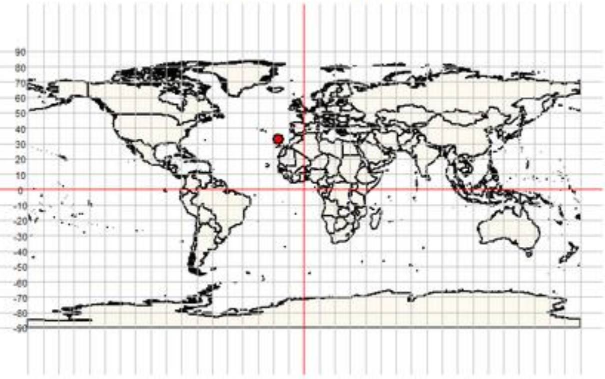

Clethra barbinervis

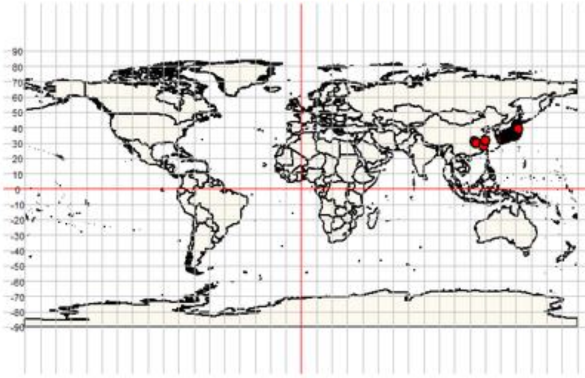

Clethra cubensis

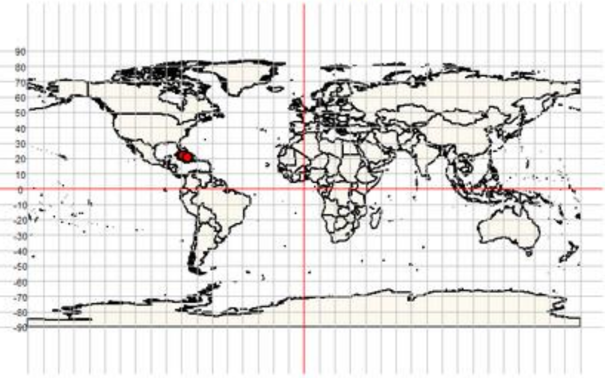

Clethra delavayi

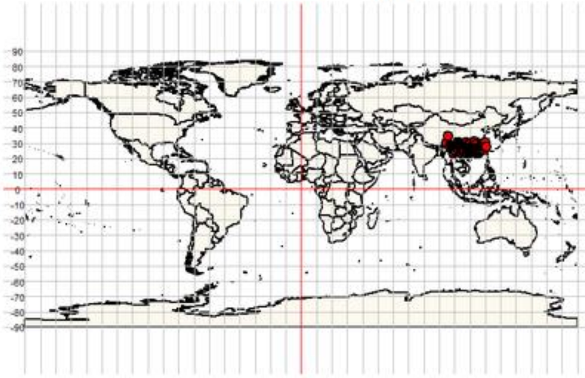

Clethra fimbriata

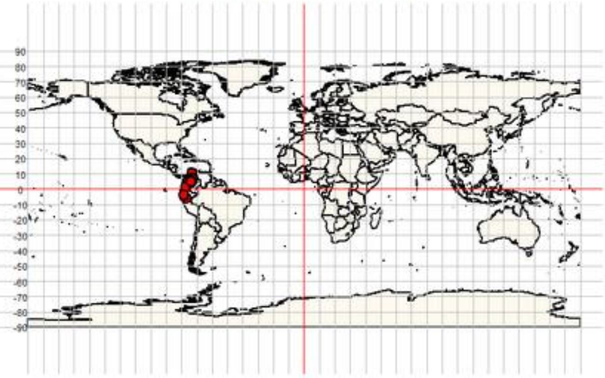

Clethra mexicana

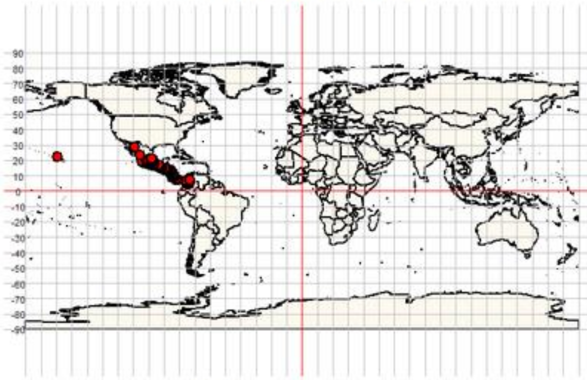

Clethra revoluta

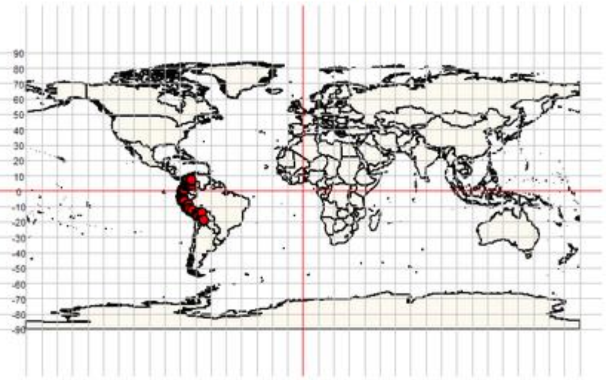

Cleyera japonica

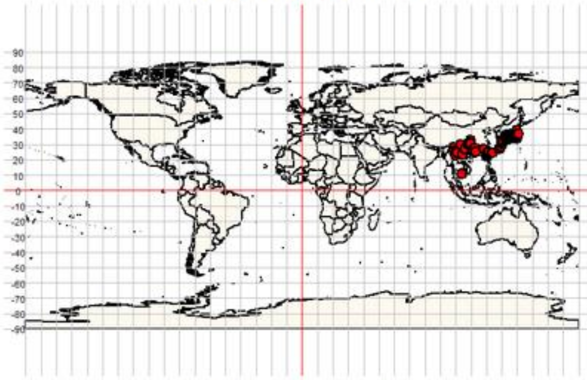

Cleyera pachyphylla

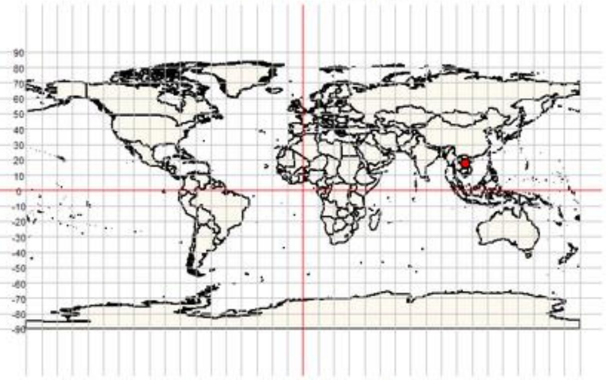

Cliftonia monophylla

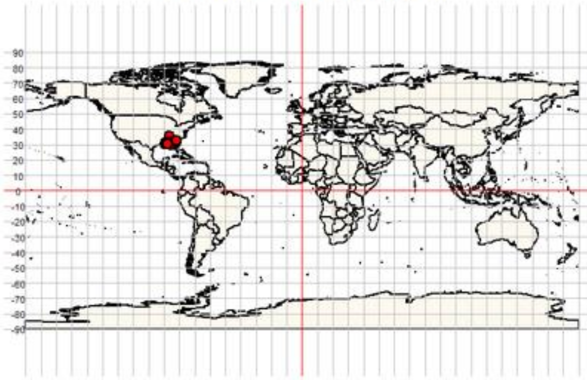

Cobaea scandens

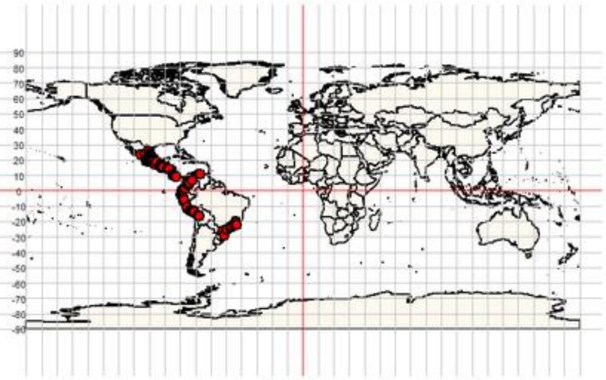

Conandrium rhynchocarpum

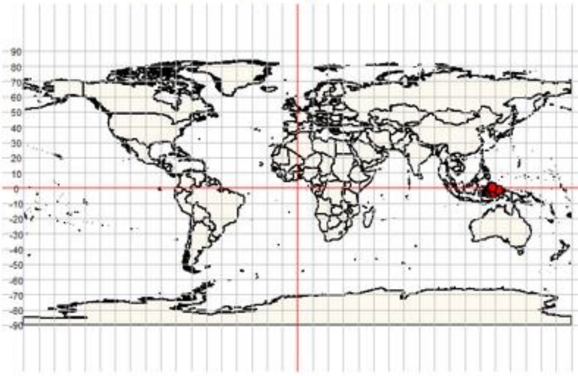

Cordyloblaste henschelii

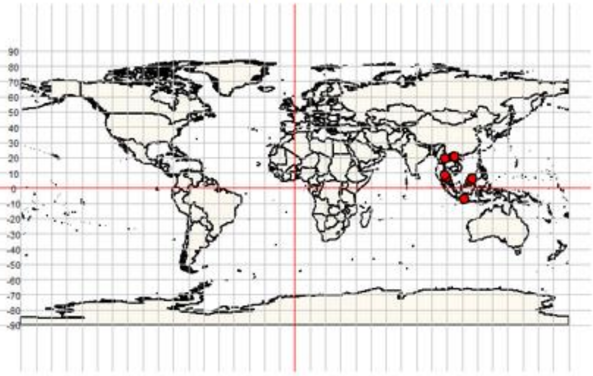

Cordyloblaste pendula

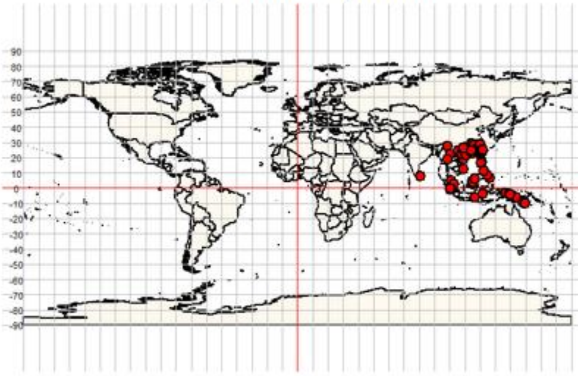

Corema conradii

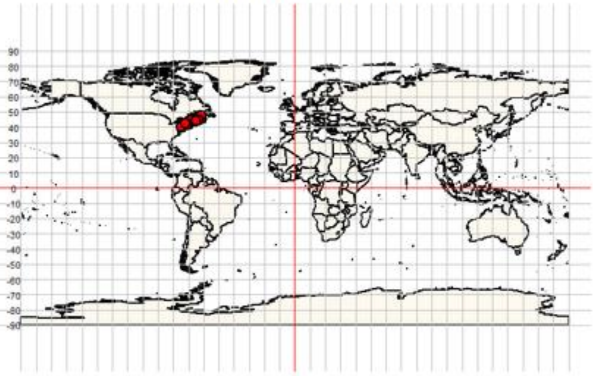

Coris monspeliensis

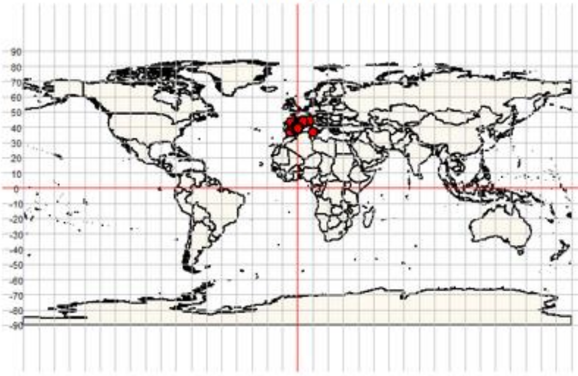

Corythophora amapaensis

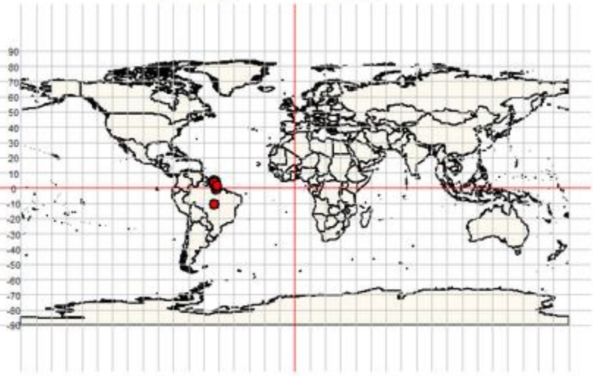

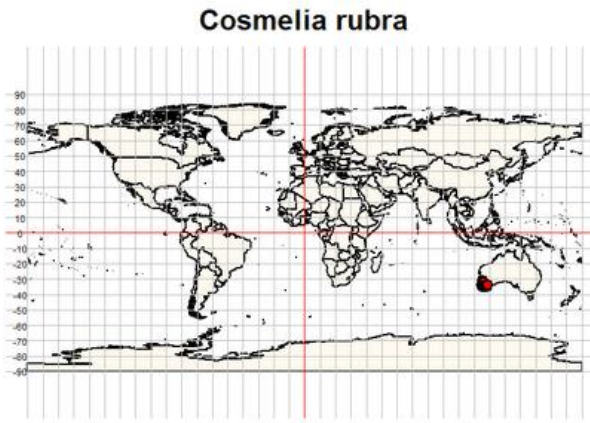
Cosmelia rubra

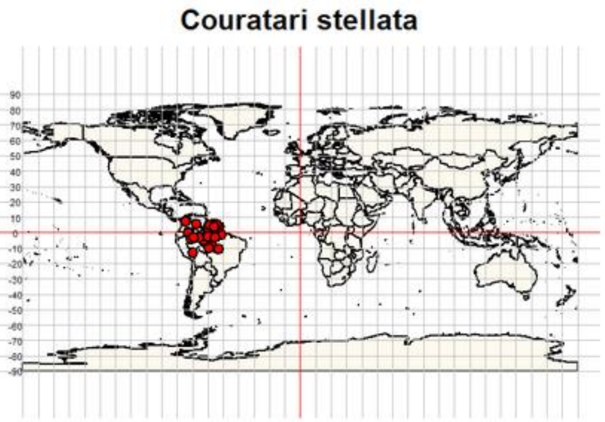
Couratari stellata

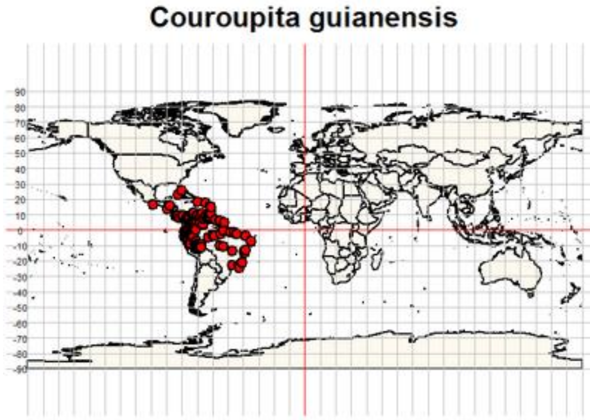
Couroupita guianensis

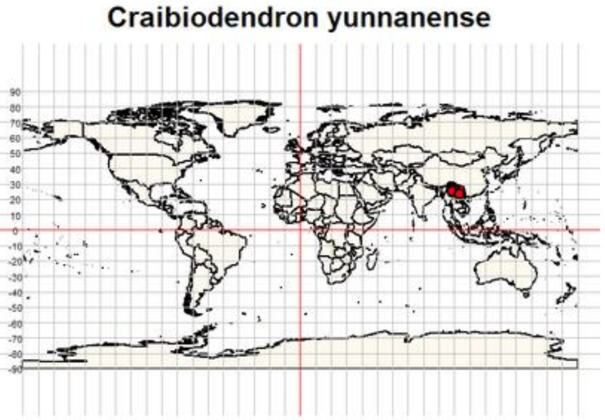
Craibiodendron yunnanense

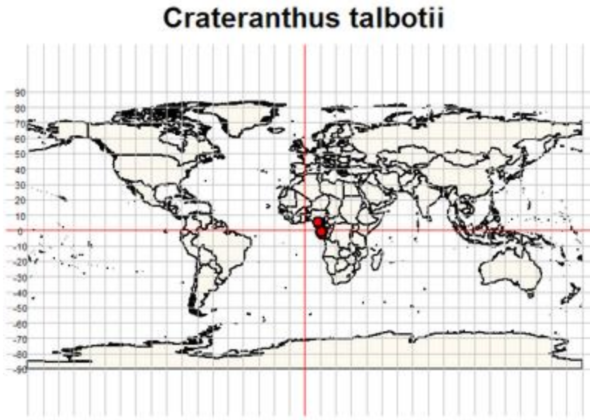
Crateranthus talbotii

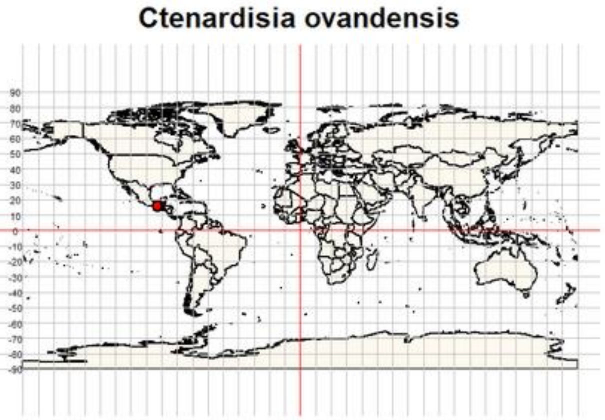
Ctenardisia ovandensis

Cybianthus candamoensis

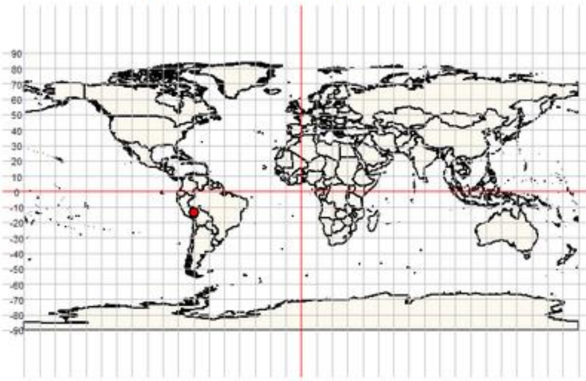

Cyclamen persicum

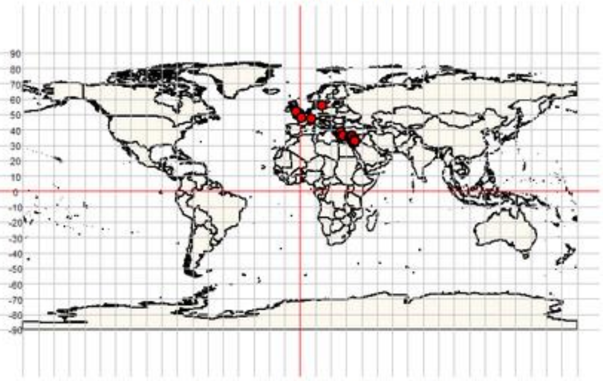

Cyrilla racemiflora

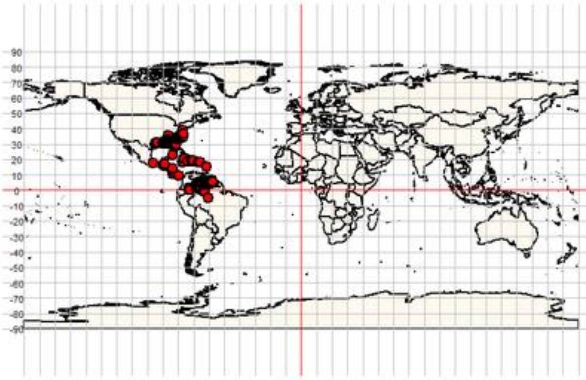

Daboecia cantabrica

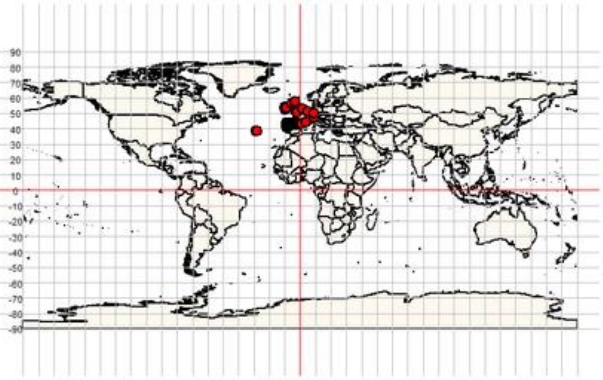

Darlingtonia californica

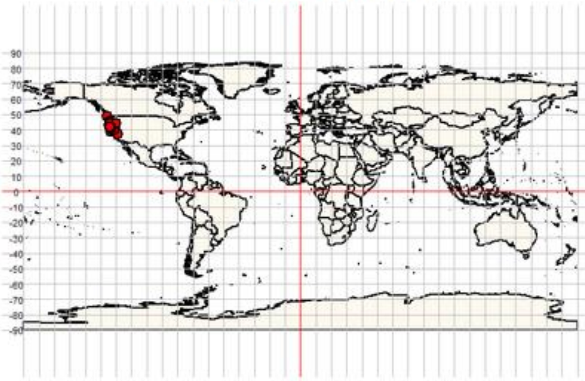

Deherainia lageniformis

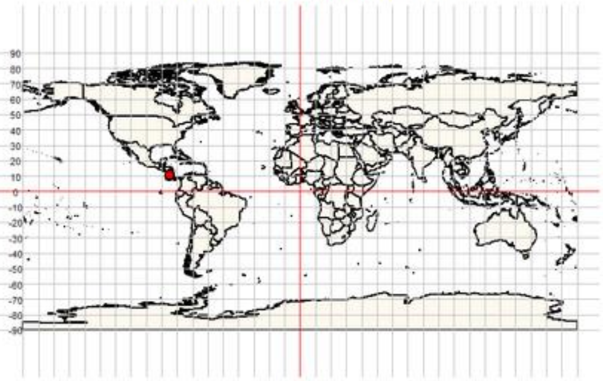

Delpydora macrophylla

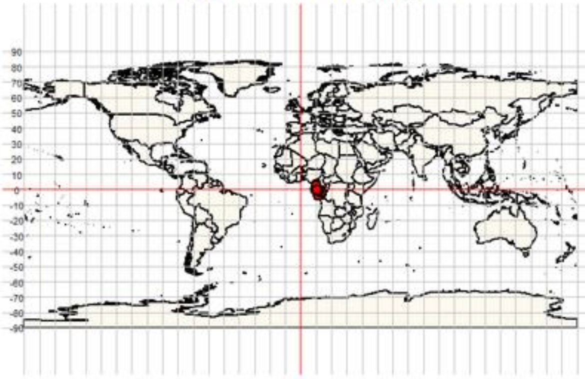

Diapensia himalaica

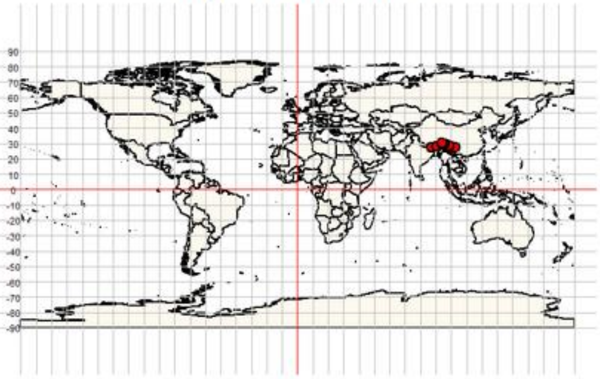

Diapensia lapponica

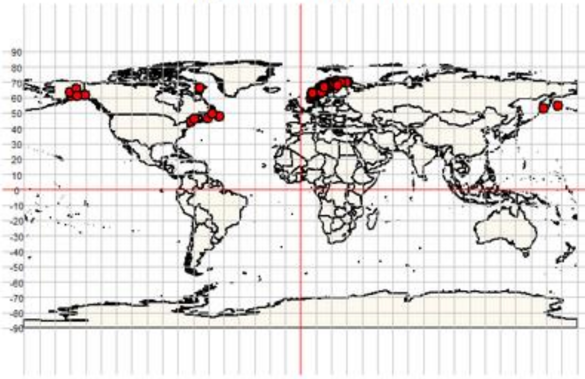

Diapensia purpurea

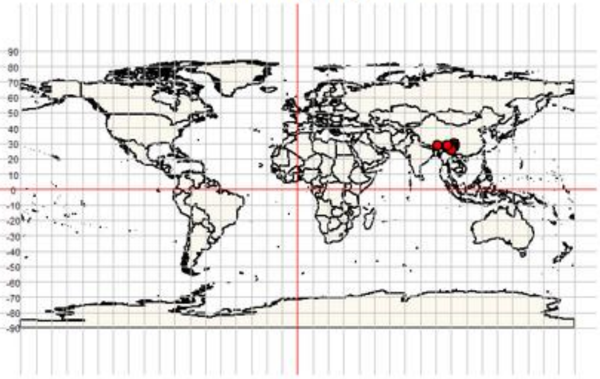

Dionysia odora

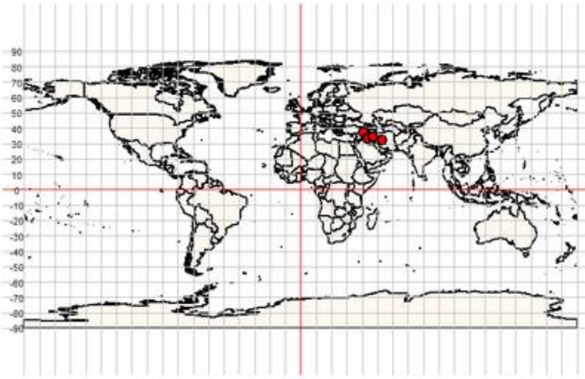

Diospyros caribaea

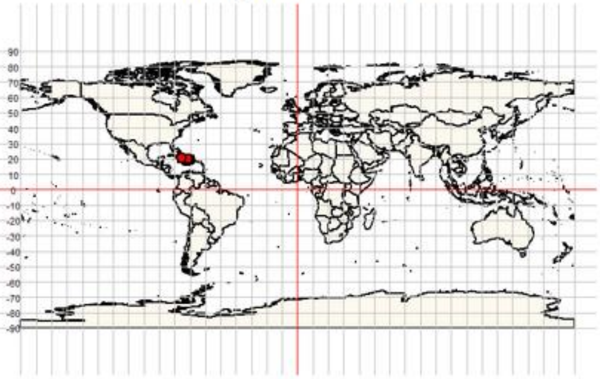

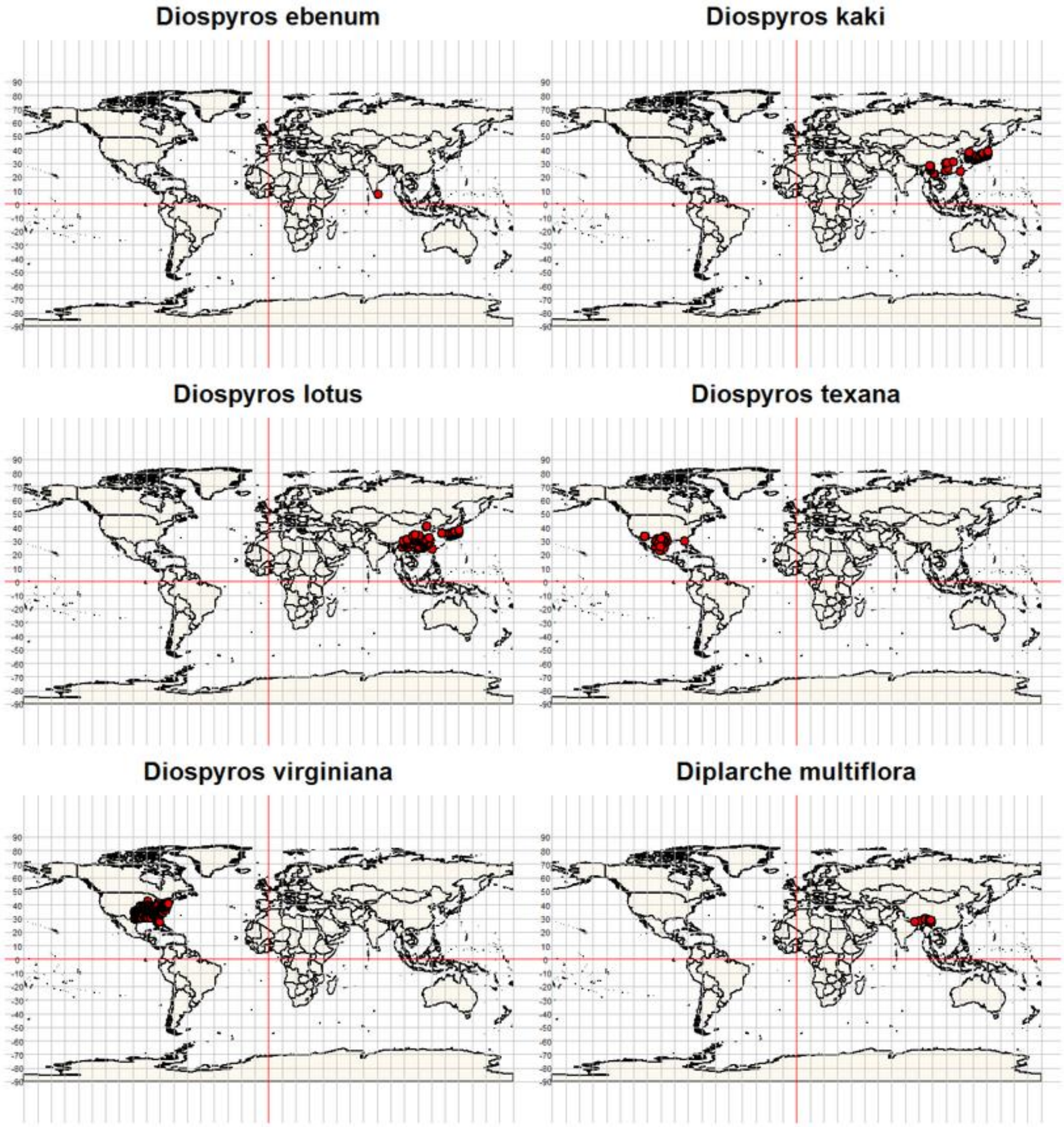
Diospyros ebenum
Diospyros kaki
Diospyros lotus
Diospyros texana
Diospyros virginiana
Diplarche multiflora

Diploknema butyracea

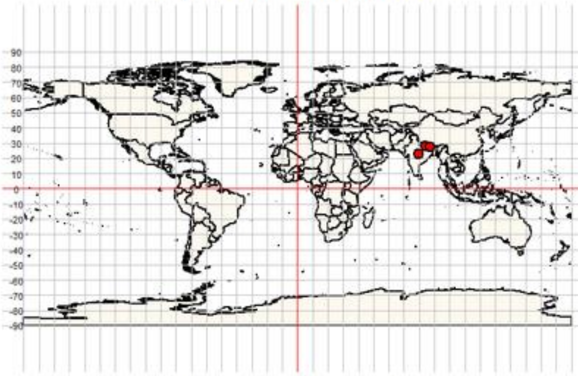

Diploon cuspidatum

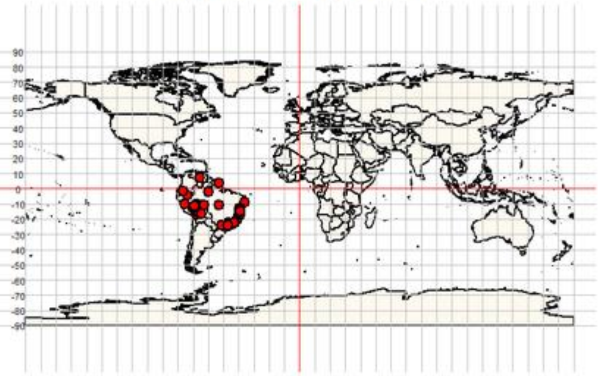

Diplycosia pinifolia

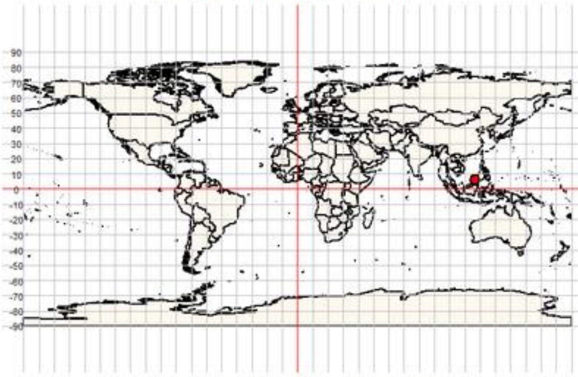

Dodecatheon alpinum

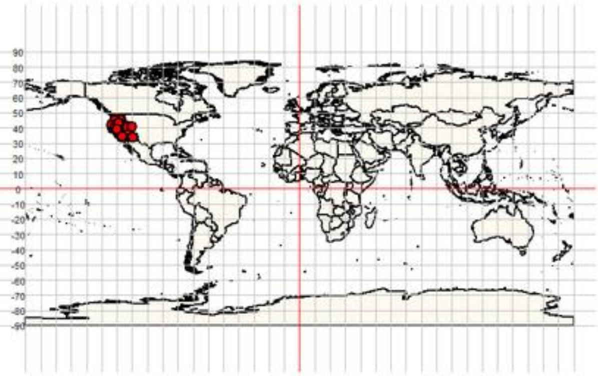

Dracophyllum latifolium

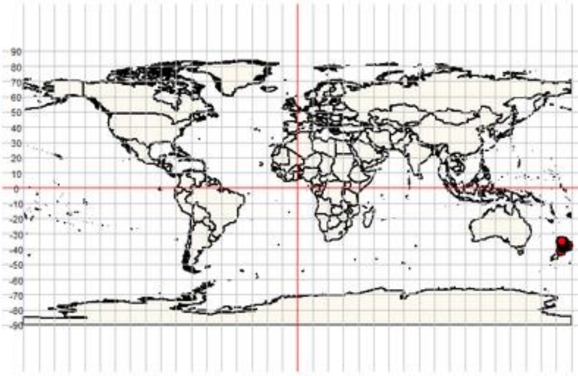

Eberhardtia tonkinensis

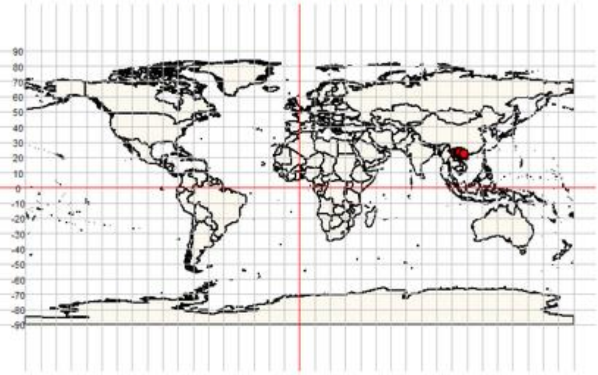

Ecclinusa ulei

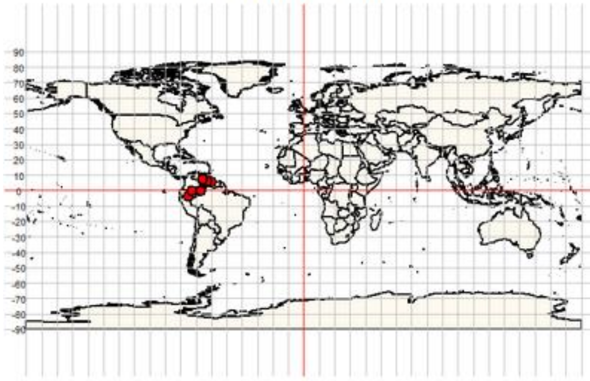

Elaeoluma glabrescens

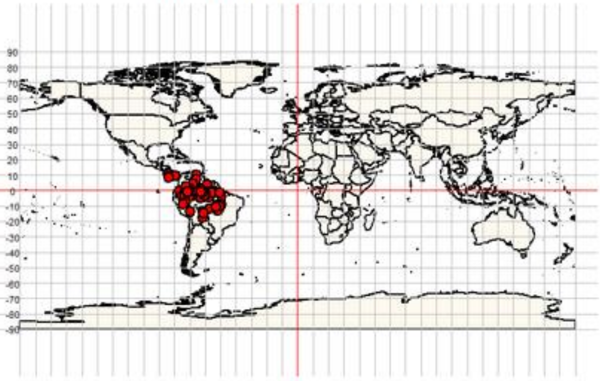

Elliottia pyroliflora

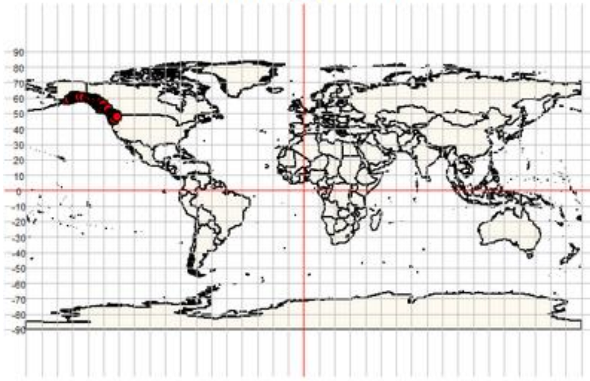

Embelia ribes

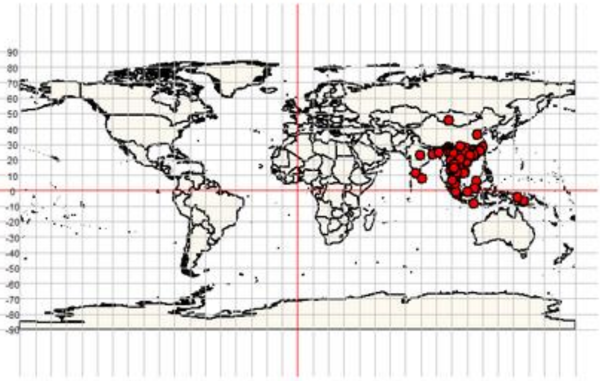

Englerophytum stelechantha

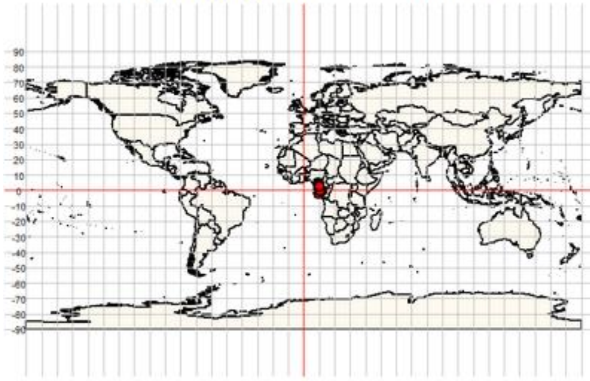

Enkianthus deflexus

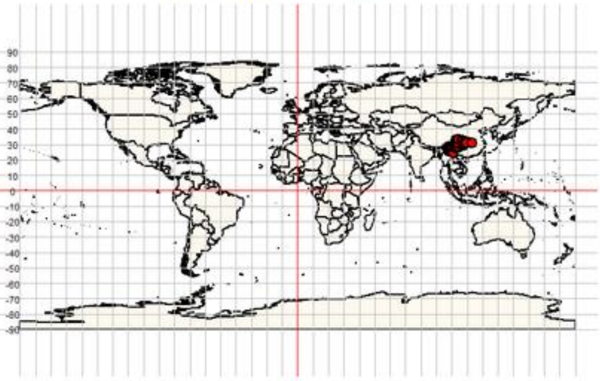

Epacris impressa

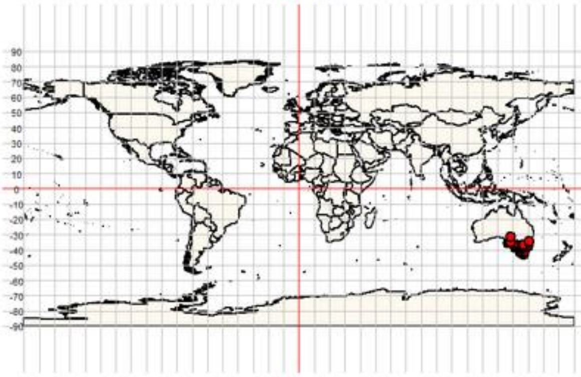

Epigaea repens

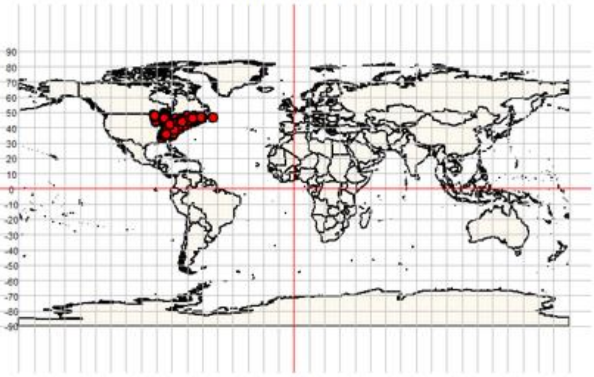

Eriastrum densifolium

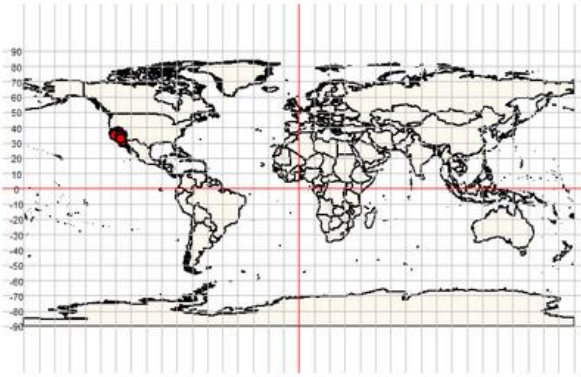

Erica tetralix

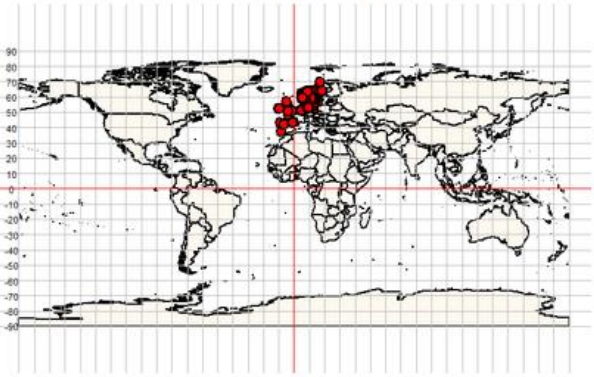

Eschweilera amazoniciformis

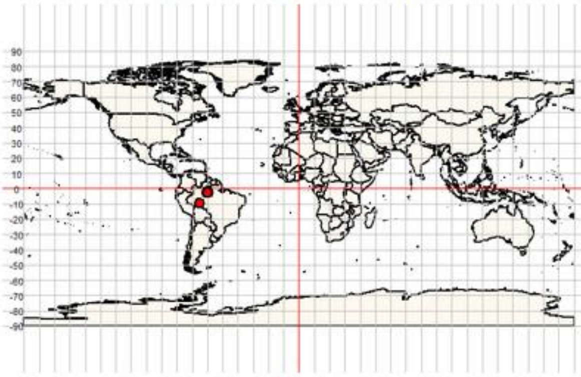

Eschweilera ovata

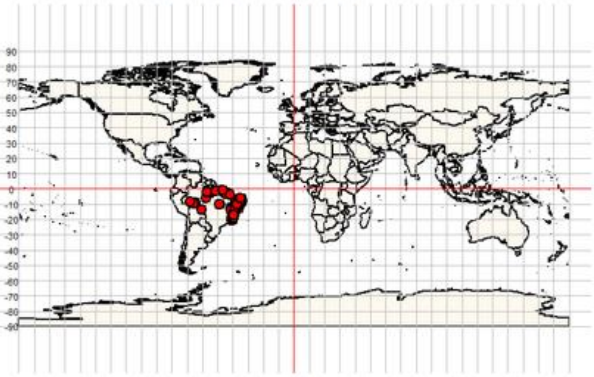

Eubotrys racemosa

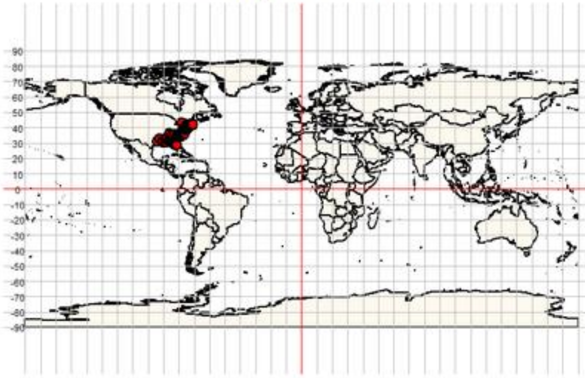

Euclea crispa

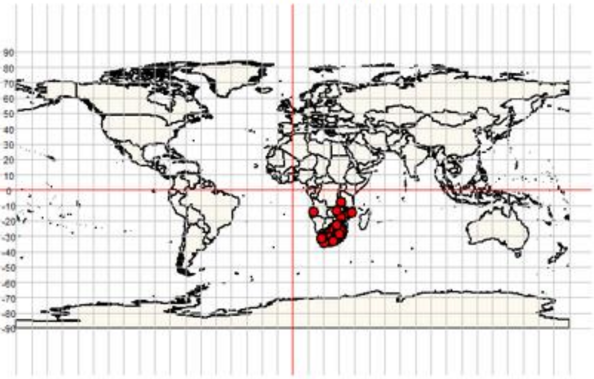

Eurya emarginata

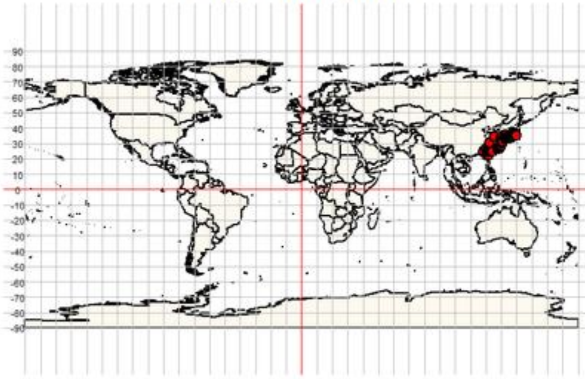

Eurya japonica

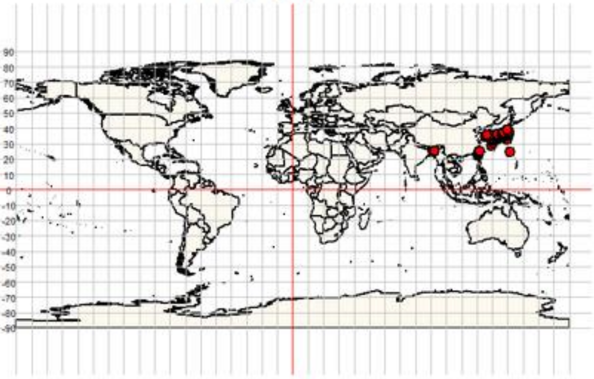

Euryodendron excelsum

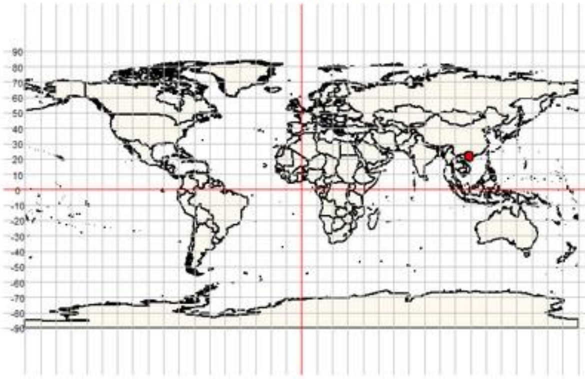

Faucherea longipedicellata

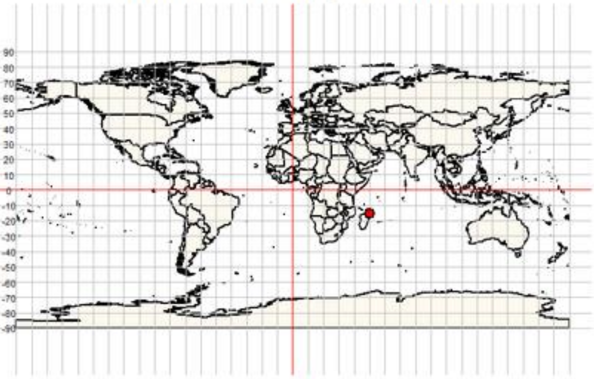

Faucherea thouvenotii

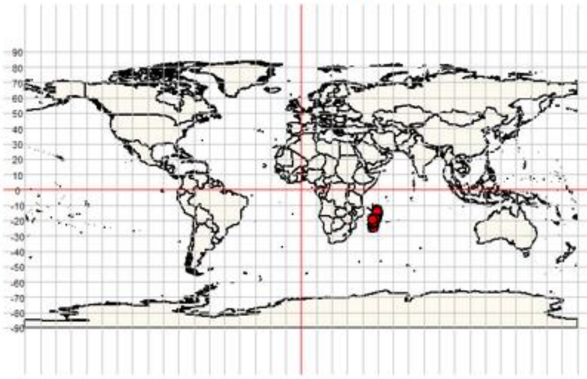

Ficalhoa laurifolia

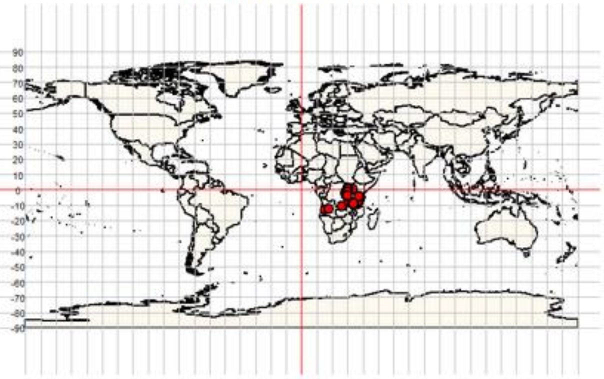

Foetidia obliqua

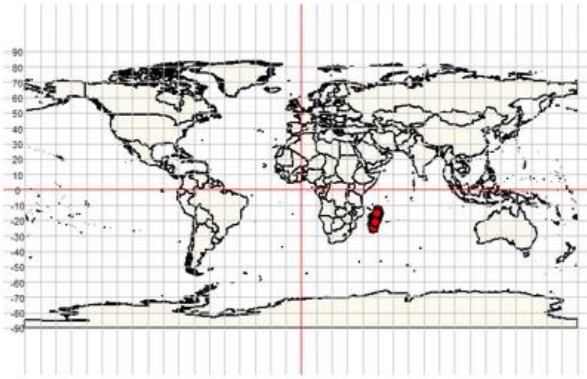

Fouquieria burragei

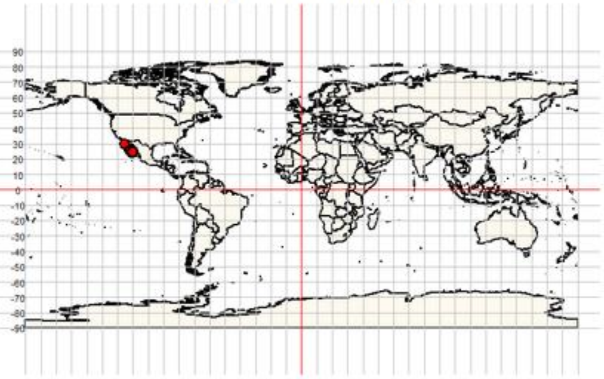

Fouquieria columnaris

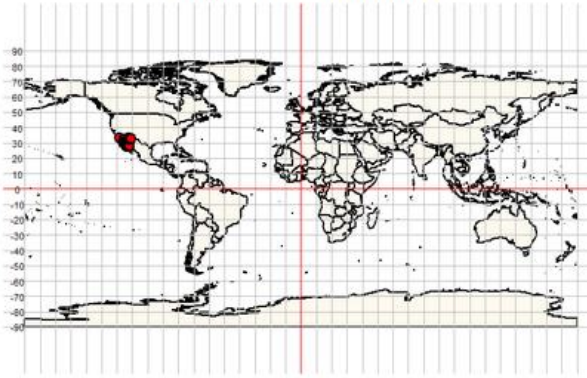

Fouquieria diguetii

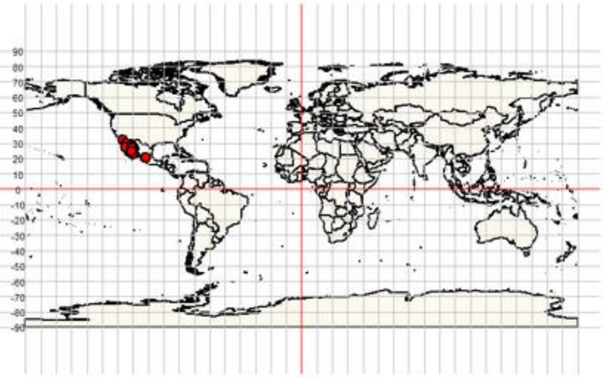

Fouquieria fasciculata

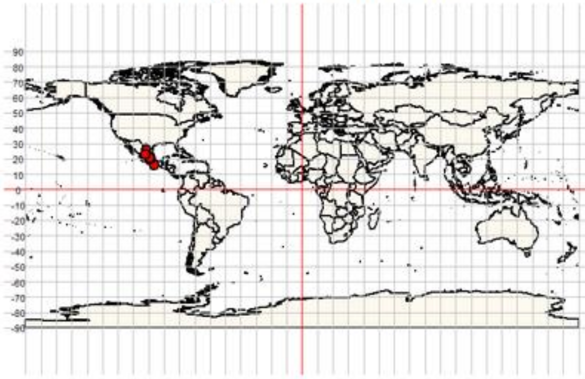

Fouquieria formosa

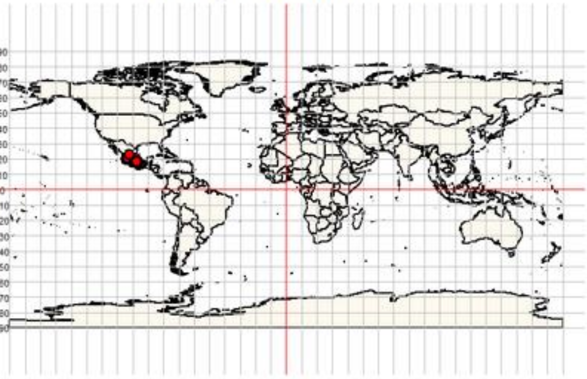

Fouquieria leonilae

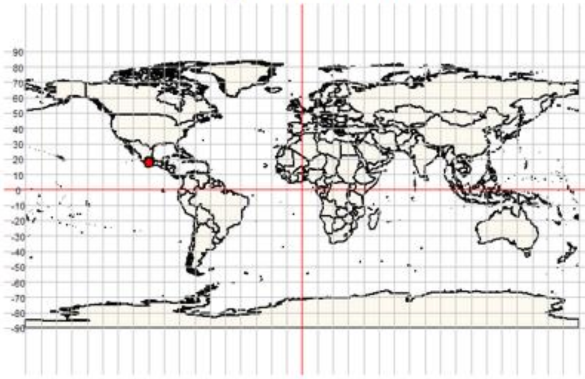

Fouquieria ochoterenae

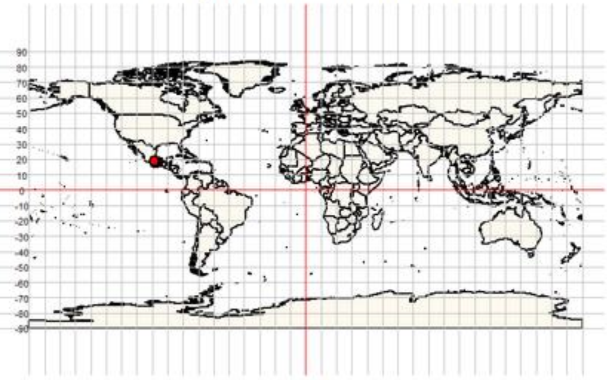

Fouquieria purpusii

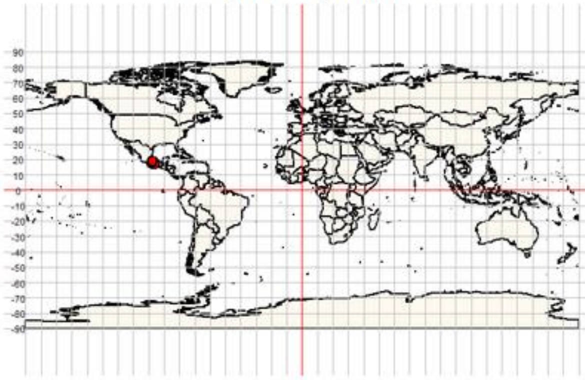

Fouquieria shrevei

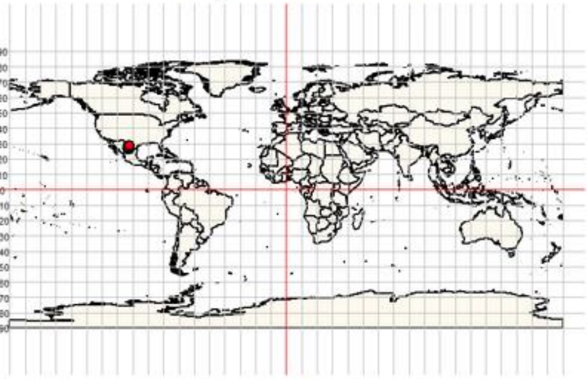

Fouquieria splendens

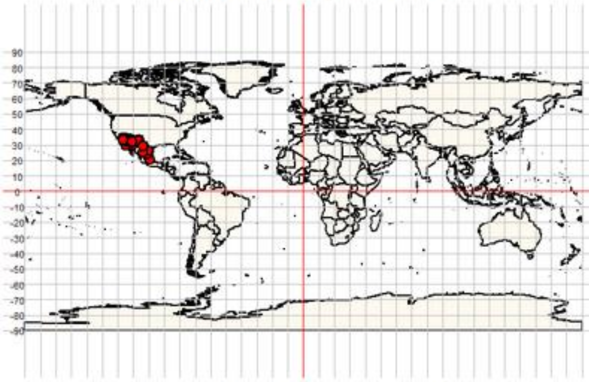

Franklinia alatamaha

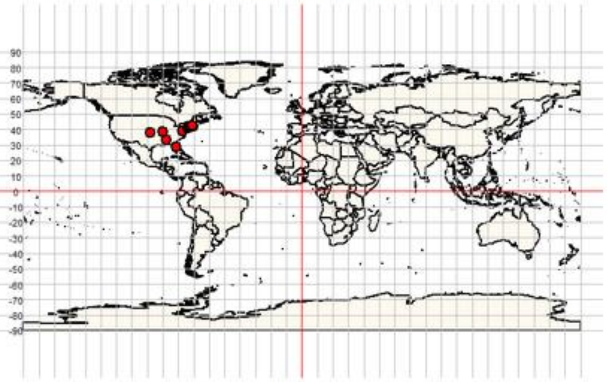

Freziera cuatrecasasii

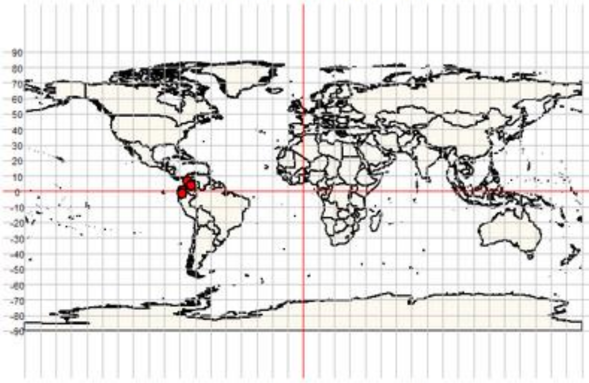

Galax urceolata

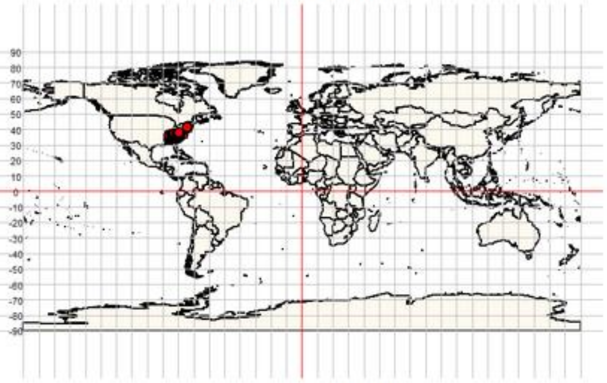

Geissanthus cogolloi

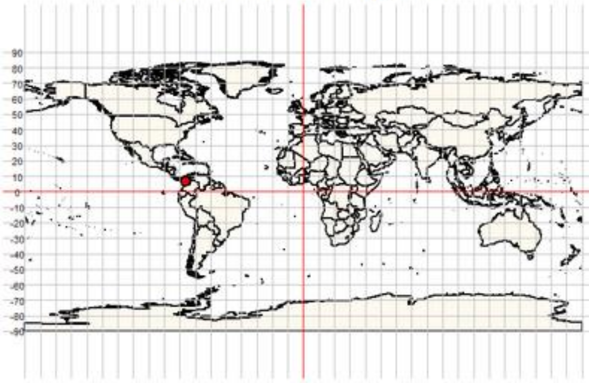

Gilia achilleifolia

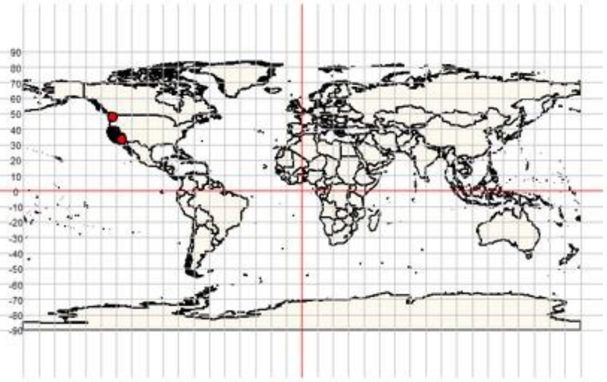

Gilia capitata

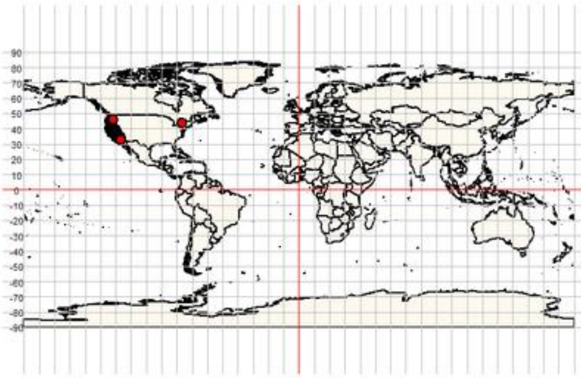

Glaux maritima

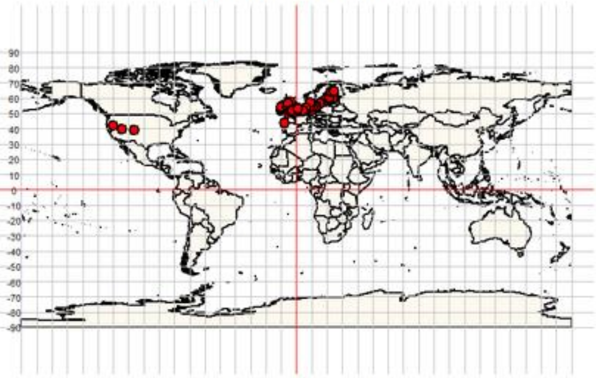

Gluema ivorensis

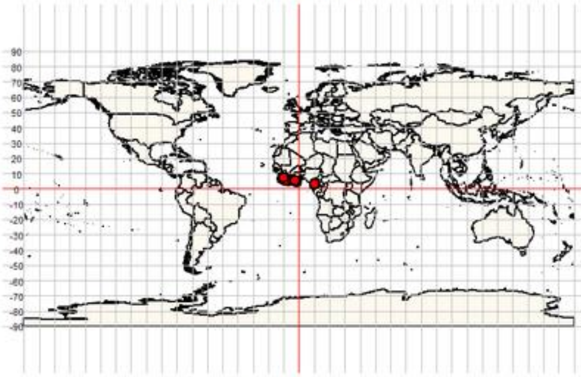

Gordonia lasianthus

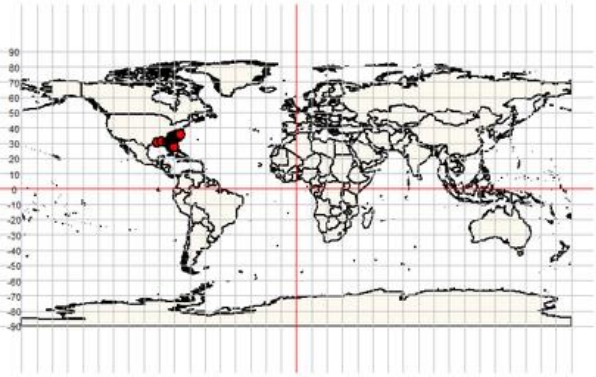

Grias peruviana

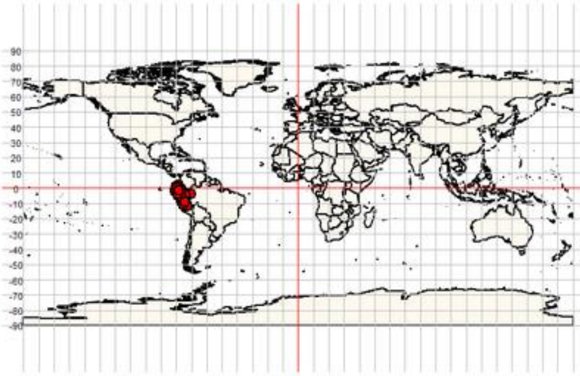

Gustavia macarenensis

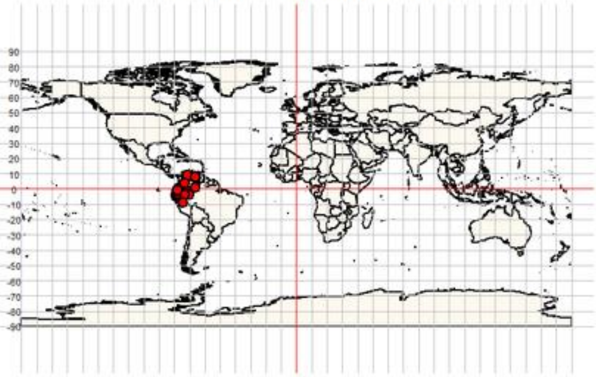

Halesia carolina

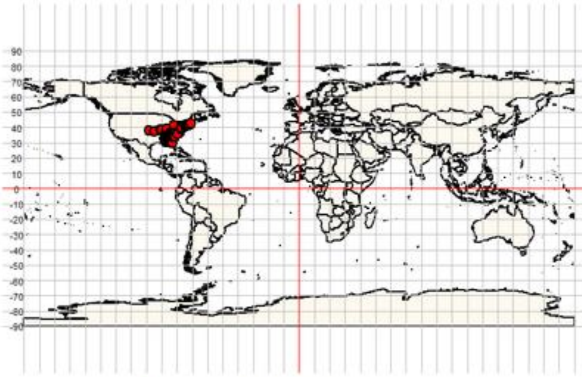

Halesia macgregorii

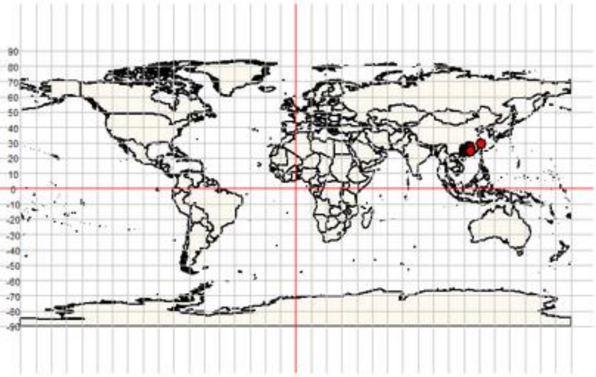

Harrimanella hypnoides

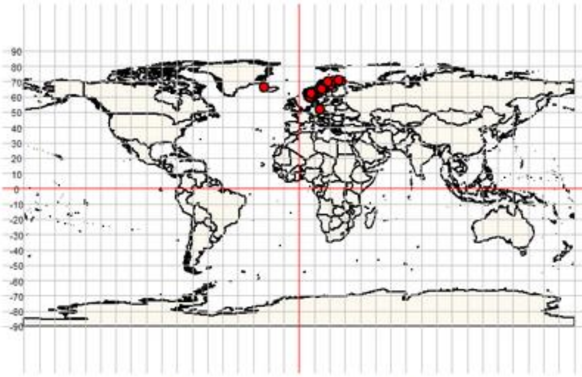

Hartia sinensis

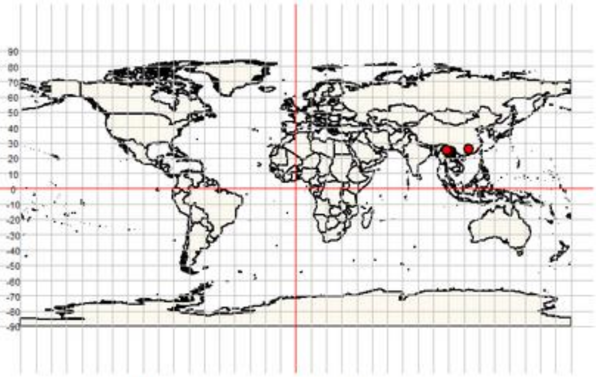

Heberdenia excelsa

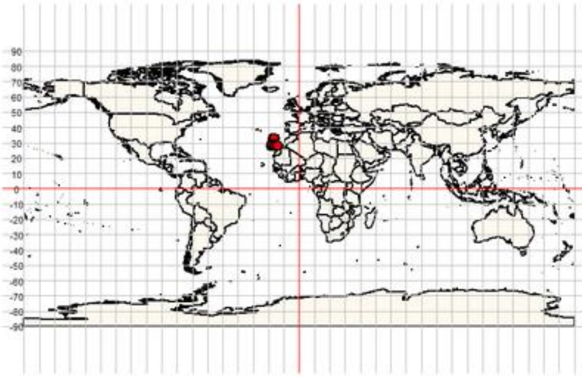

Heliamphora heterodoxa

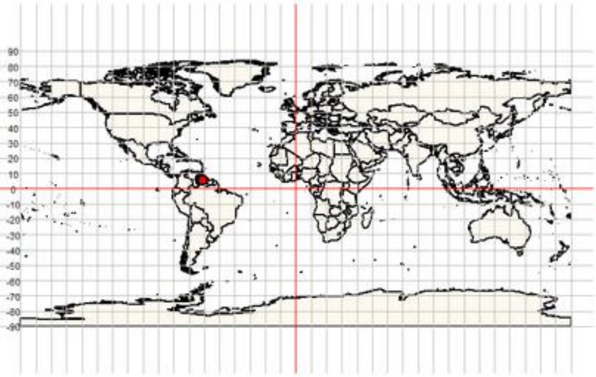

Heliamphora minor

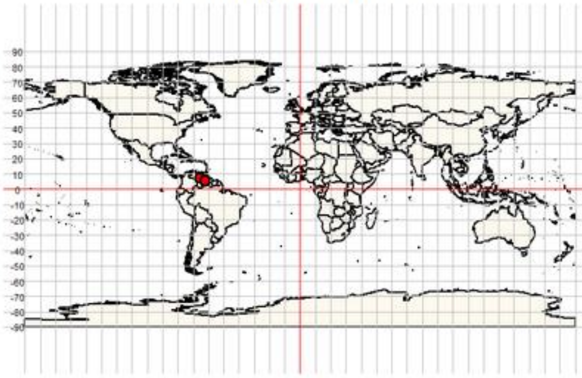

Heliamphora nutans

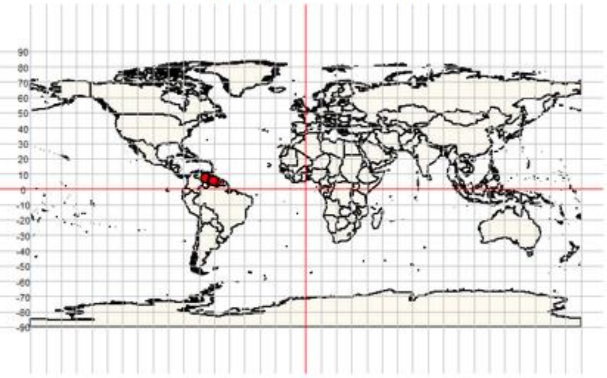

Heliamphora tatei

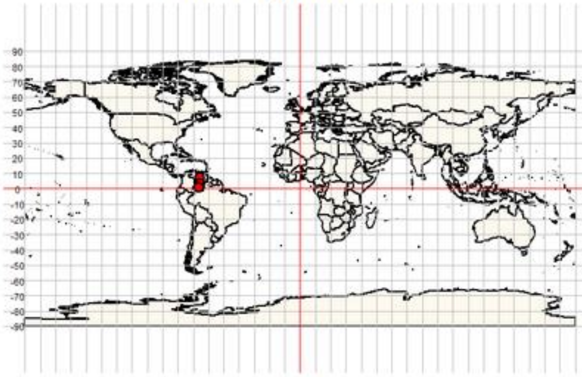

Hottonia inflata

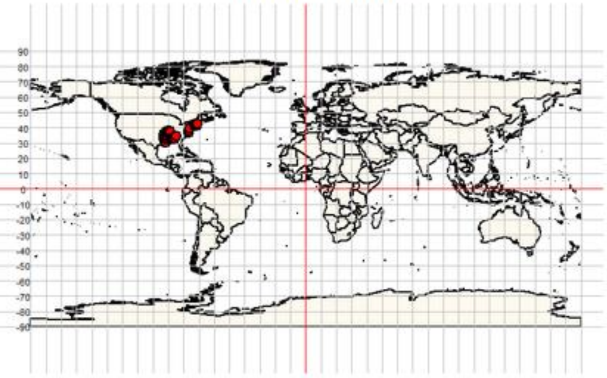

Huodendron tibeticum

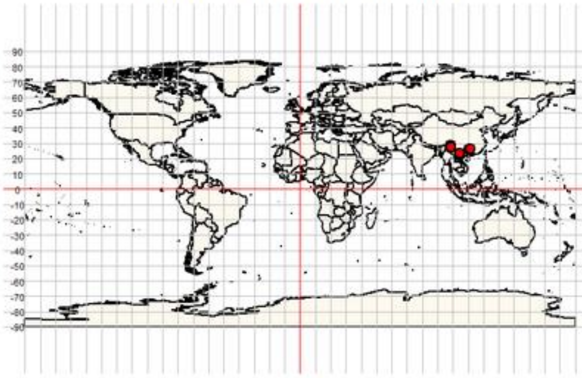

Hydrocera triflora

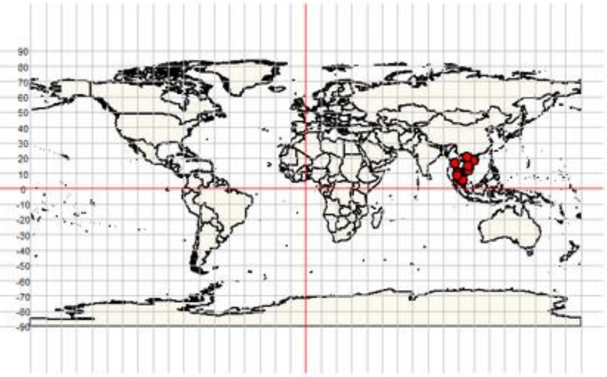

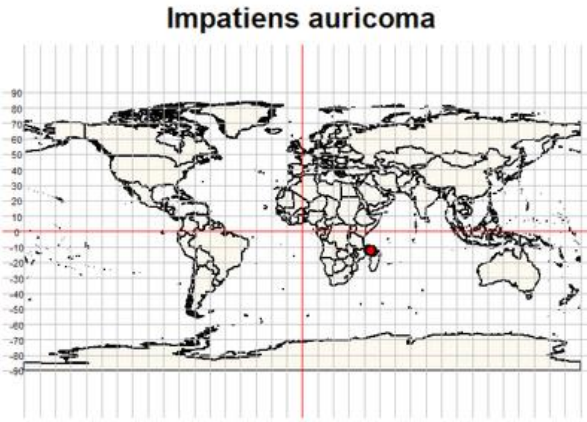
Impatiens auricoma

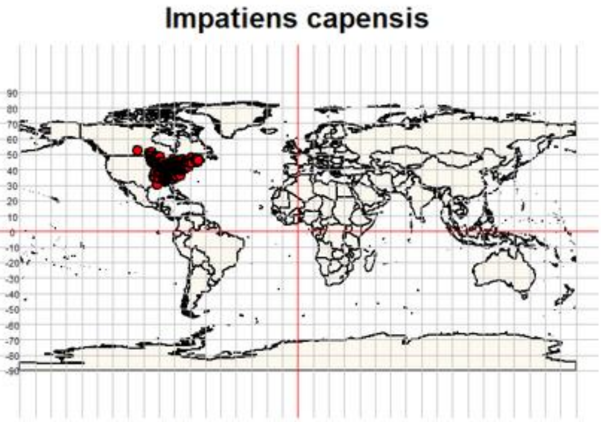
Impatiens capensis

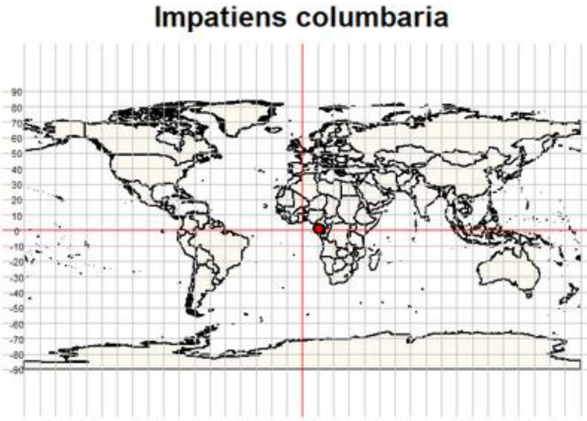
Impatiens columbaria

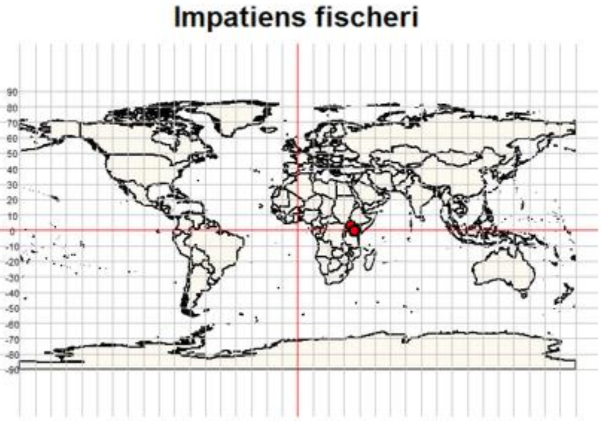
Impatiens fischeri

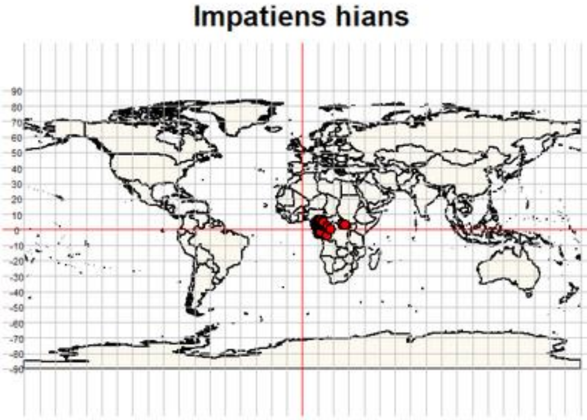
Impatiens hians

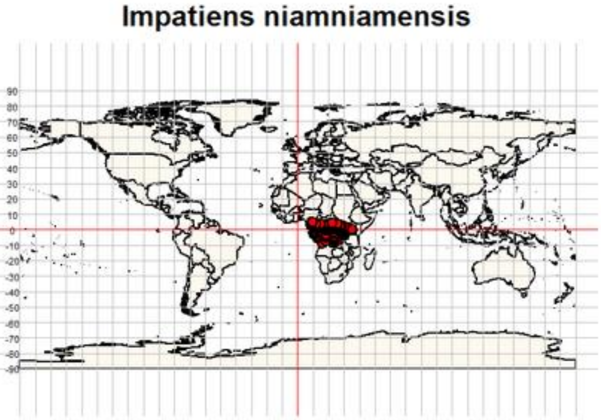
Impatiens niamniamensis

Impatiens parviflora

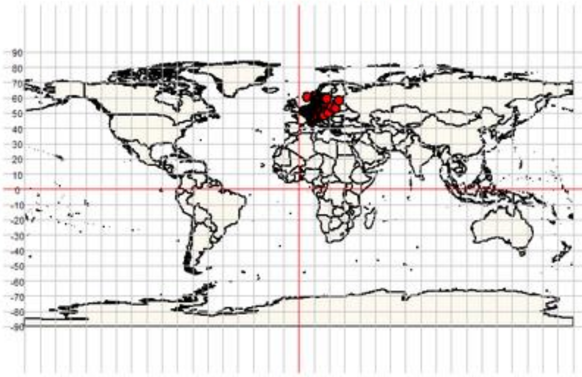

Impatiens pseudoviola

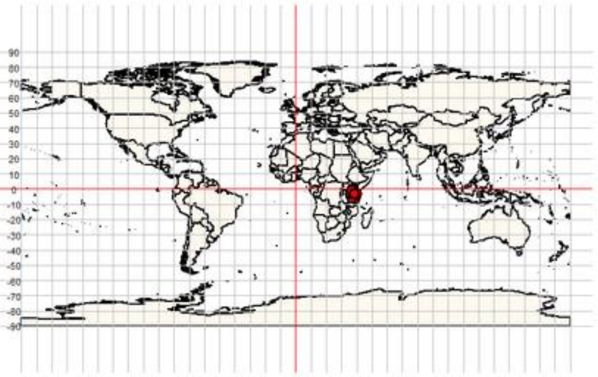

Ipomopsis tenuifolia

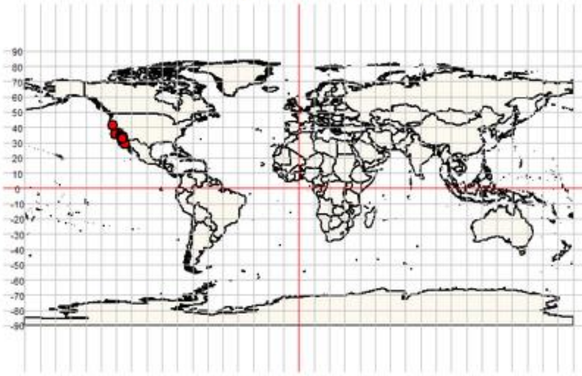

Isonandra lanceolata

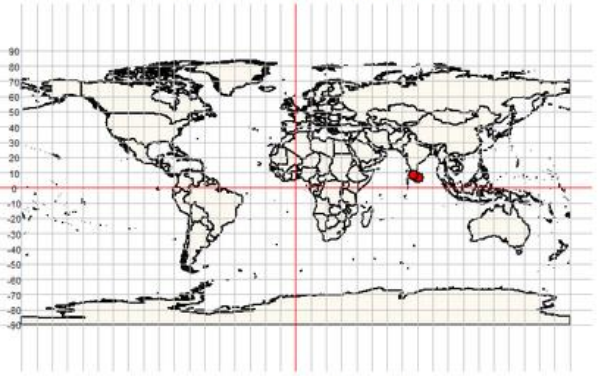

Jacquinia armillaris

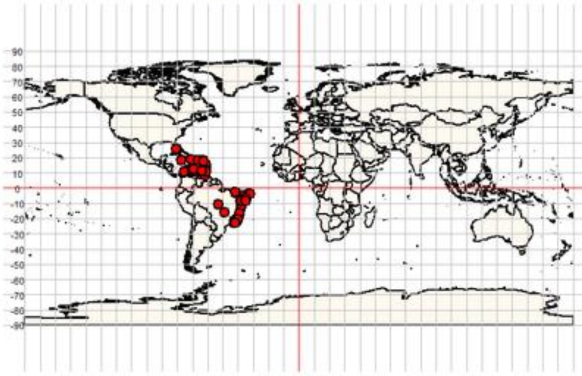

Kalmia buxifolia

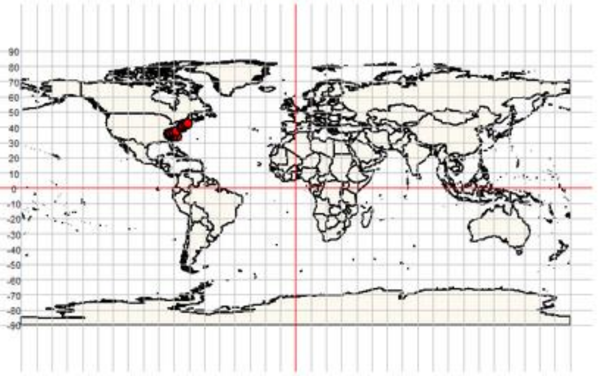

Kalmiopsis leachiana

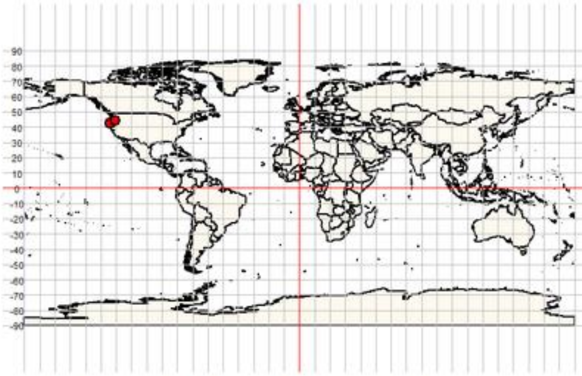

Labisia pumila

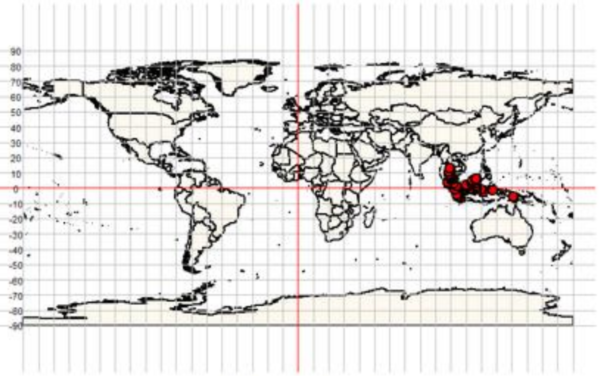

Labourdonnaisia madagascariensis

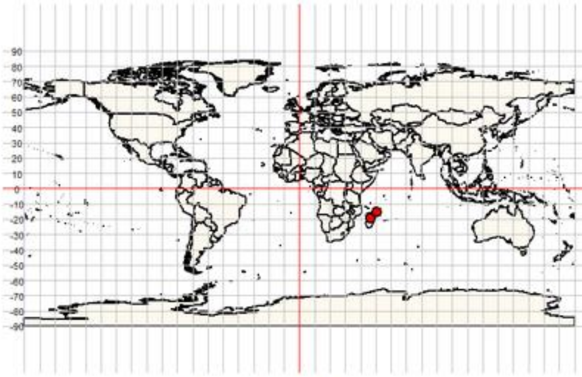

Labramia costata

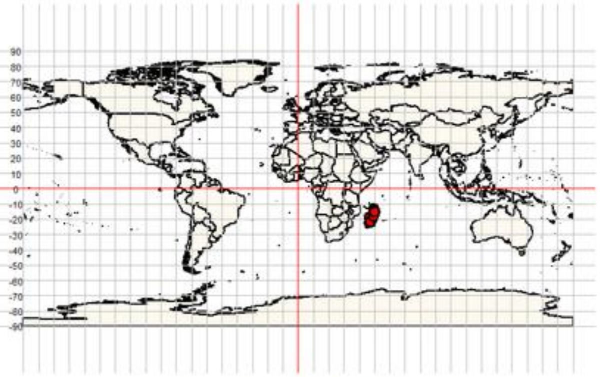

Laplacea alpestris

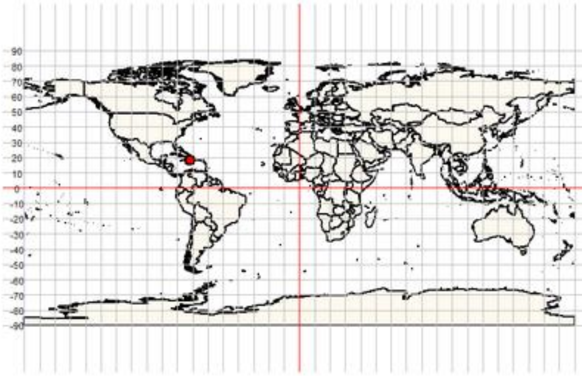

Lebetanthus myrsinites

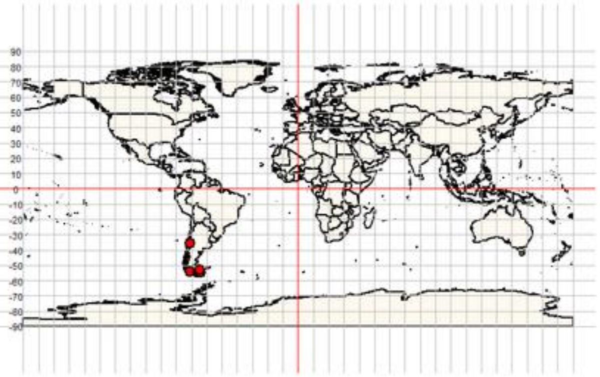

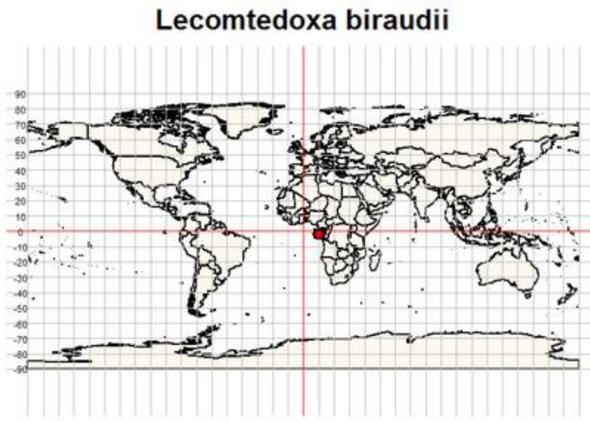
Lecomtedoxa biraudii

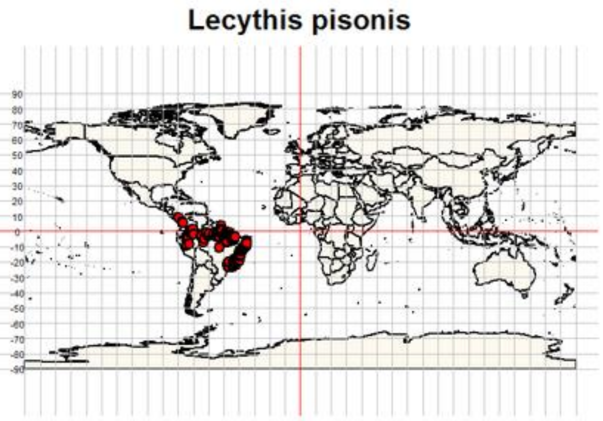
Lecythis pisonis

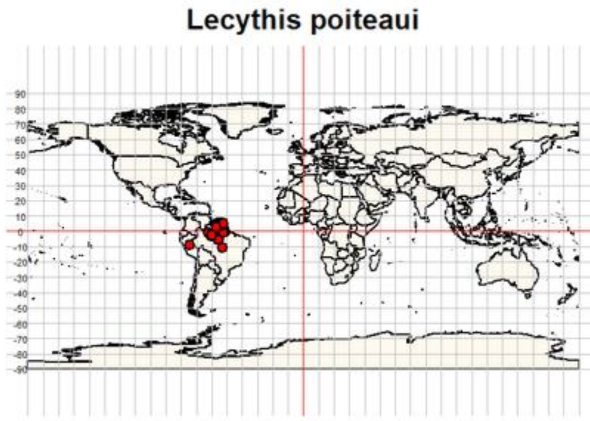
Lecythis poiteaui

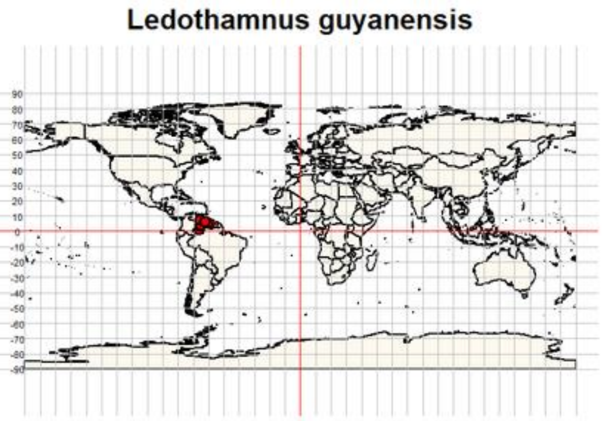
Ledothamnus guyanensis

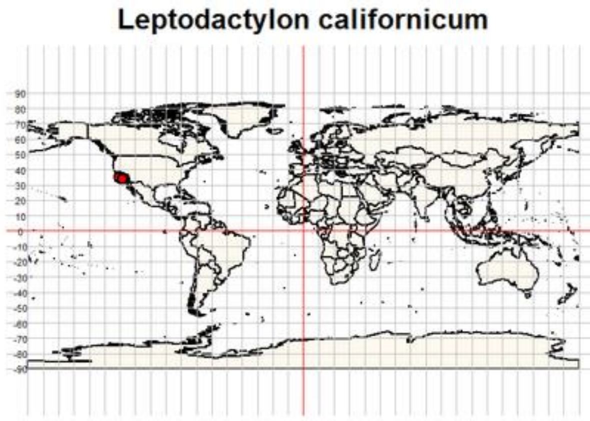
Leptodactylon californicum

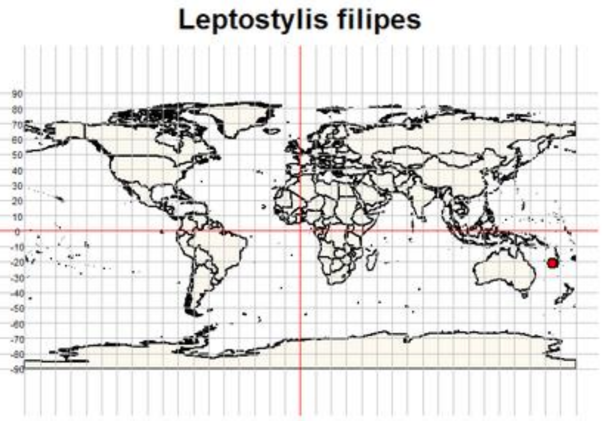
Leptostylis filipes

Letestua durissima

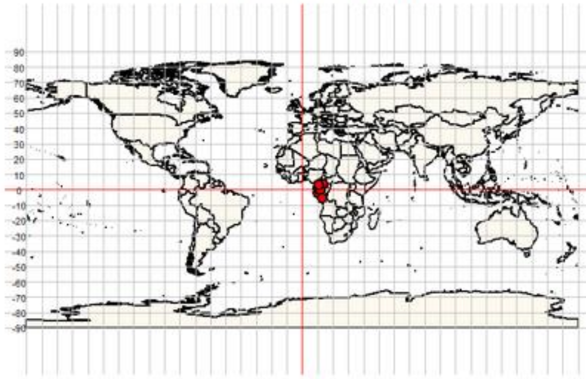

Leucopogon verticillatus

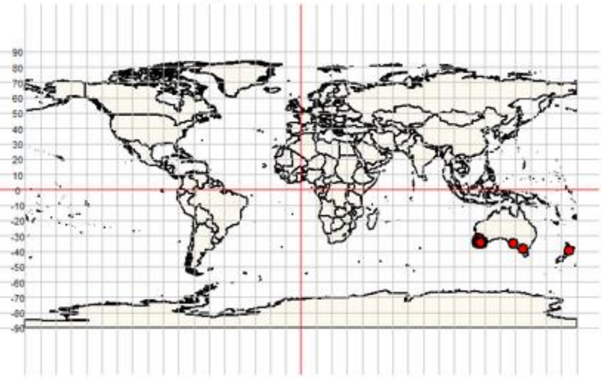

Leucothoe fontanesiana

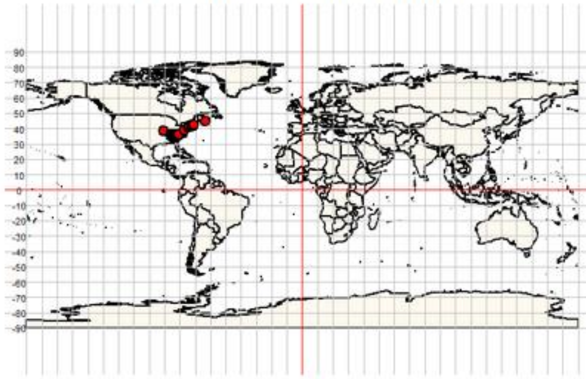

Linanthus jepsonii

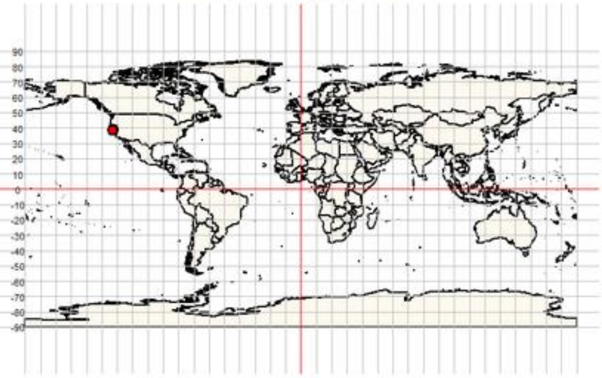

Lissocarpa benthamii

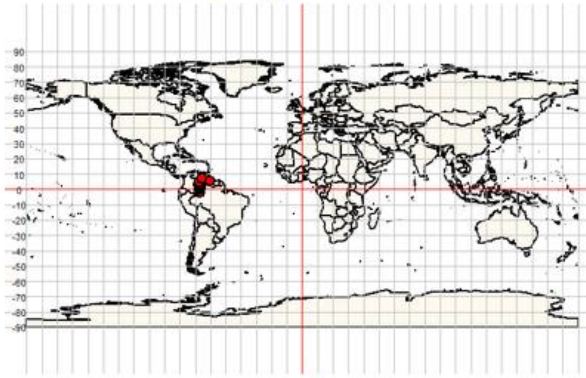

Lissocarpa guianensis

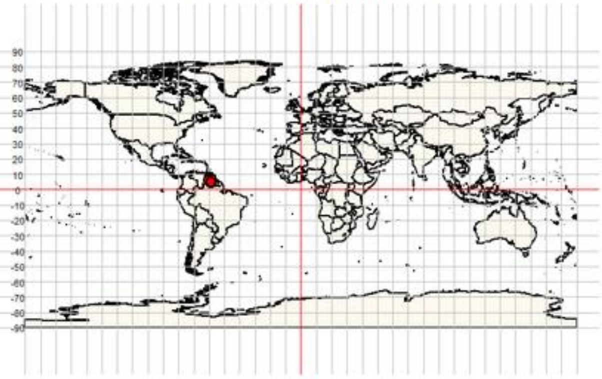

Lissocarpa uyat

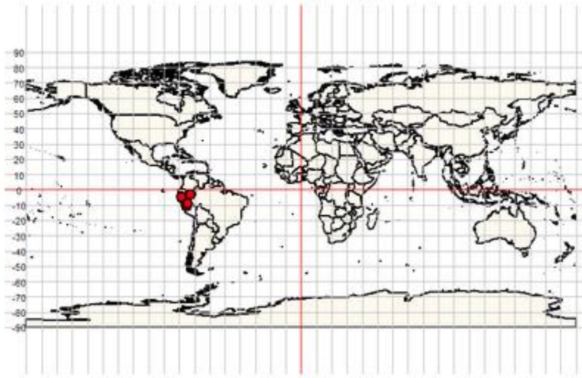

Lyonia ovalifolia

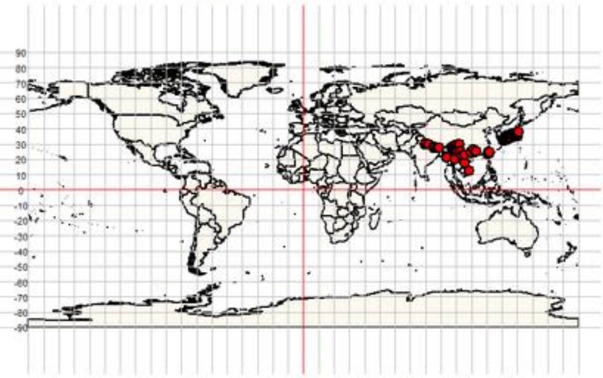

Lysimachia clethroides

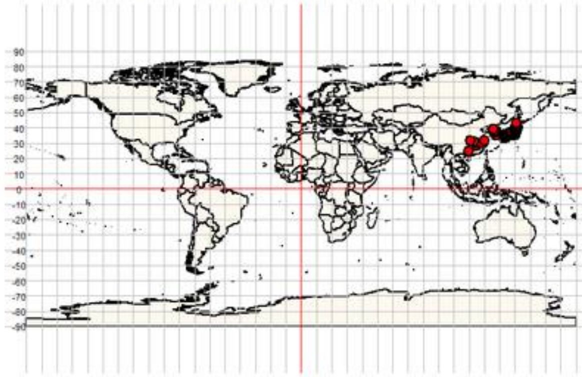

Lysimachia nummularia

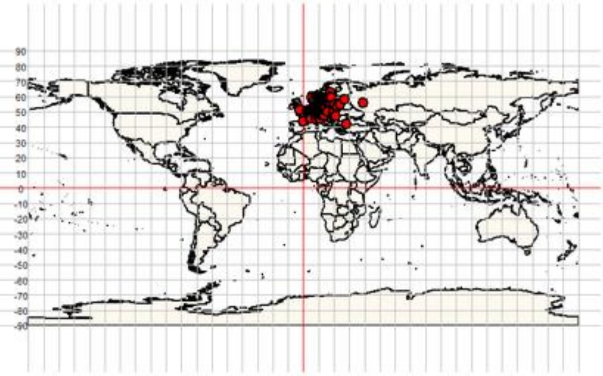

Lysimachia ruhmeriana

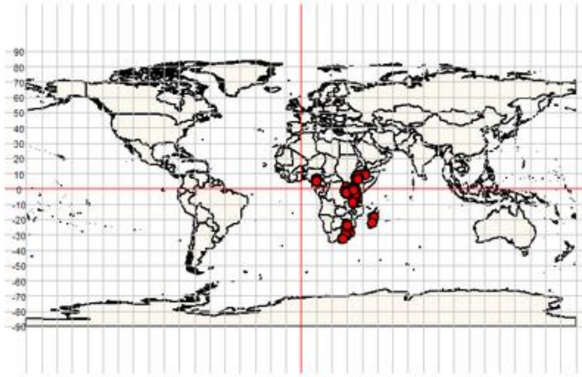

Lysimachia vittiformis

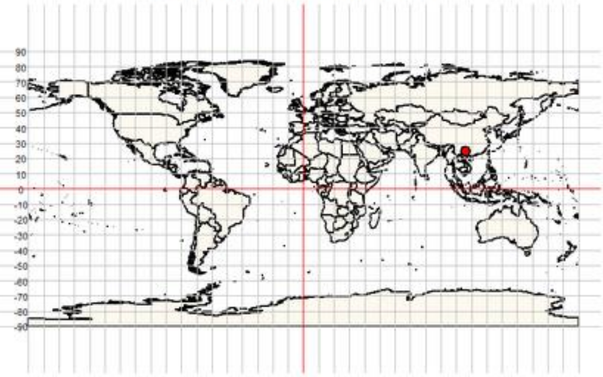

Lysimachia vulgaris

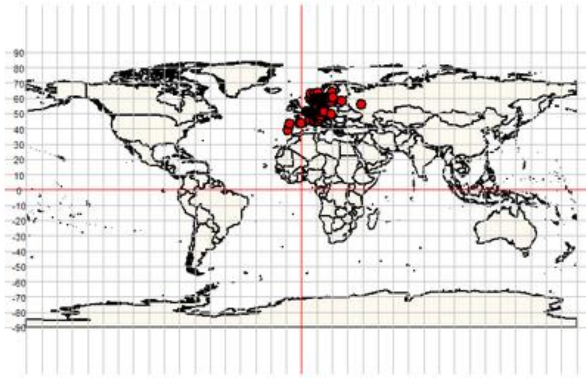

Madhuca hainanensis

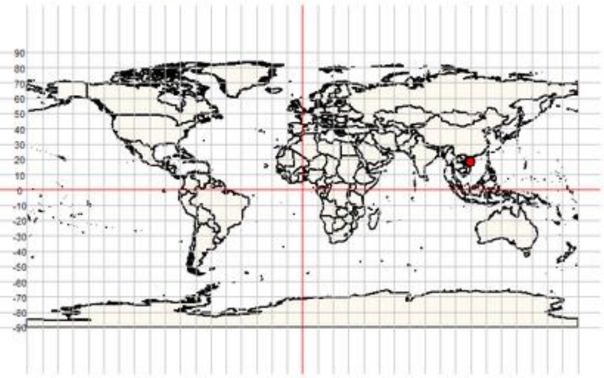

Madhuca microphylla

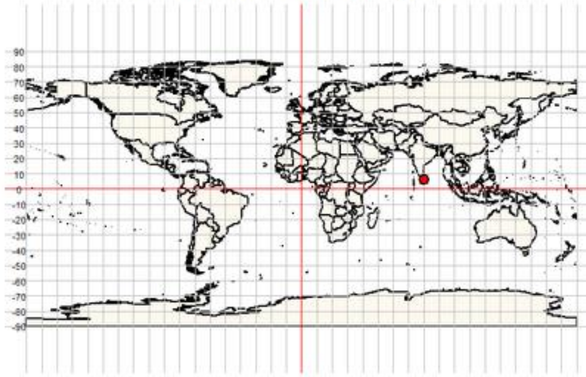

Maesa japonica

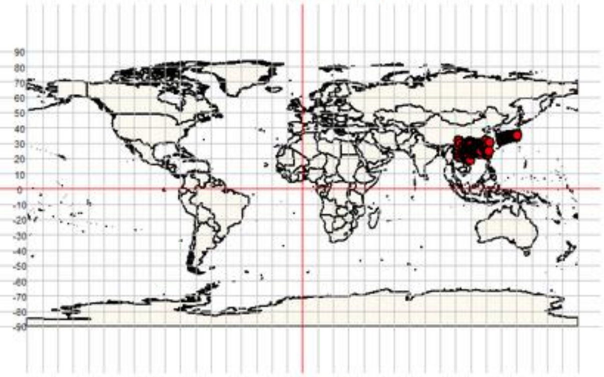

Magodendron venefici

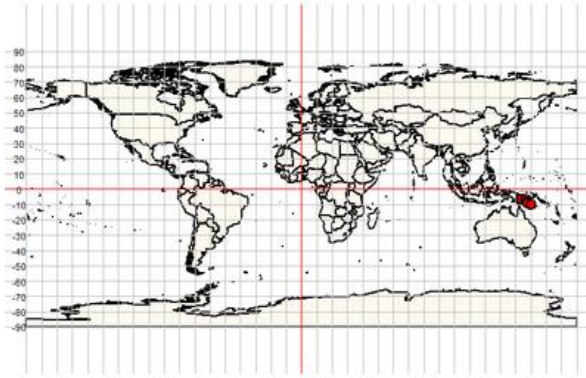

Malachodendron ovatum

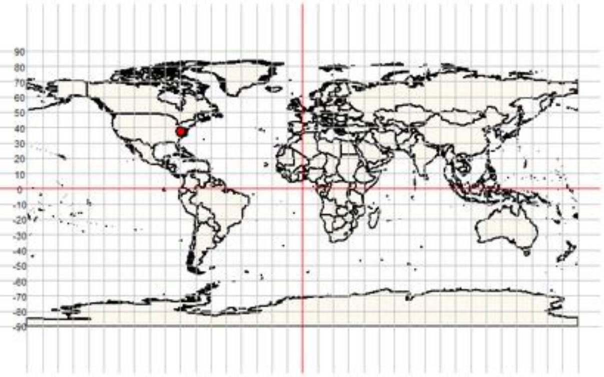

Manilkara zapota

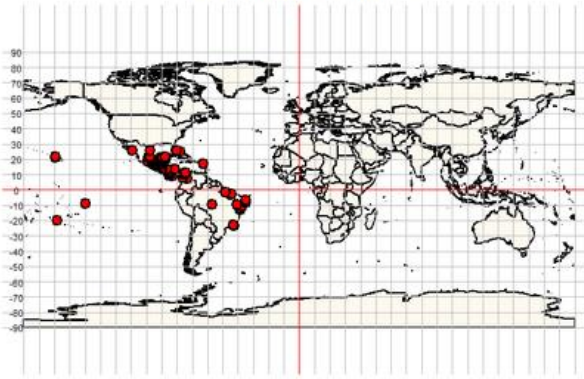

Marcgravia rectiflora

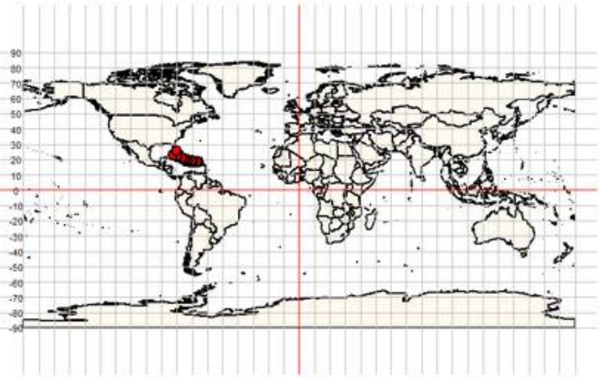

Marcgravia umbellata

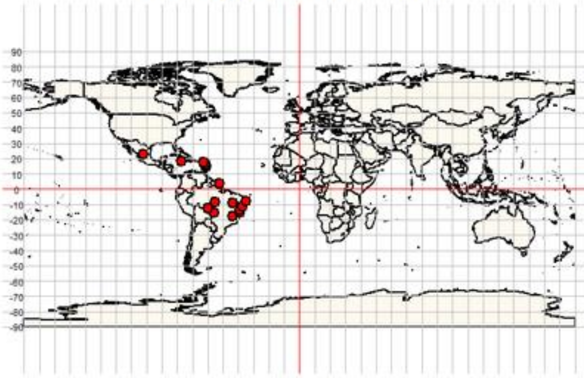

Marcgraviastrum pauciflorum

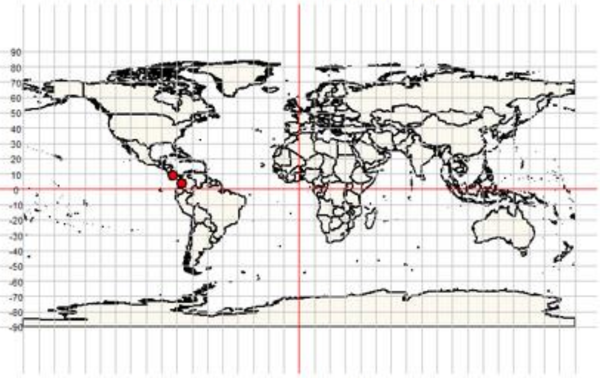

Marcgraviastrum subsessile

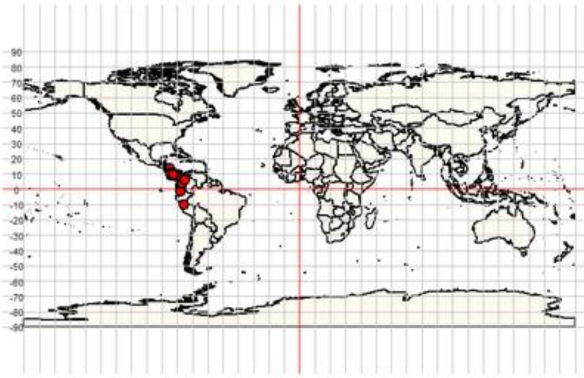

Melliodendron xylocarpum

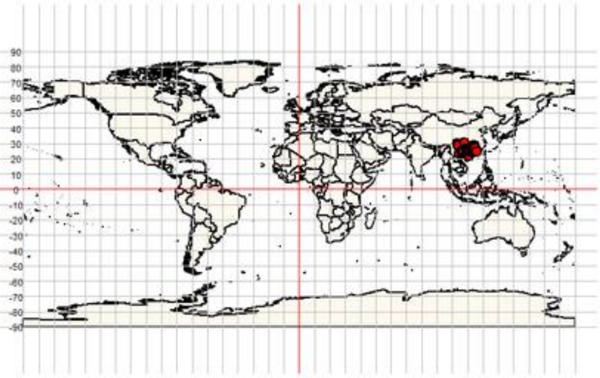

Menziesia ferruginea

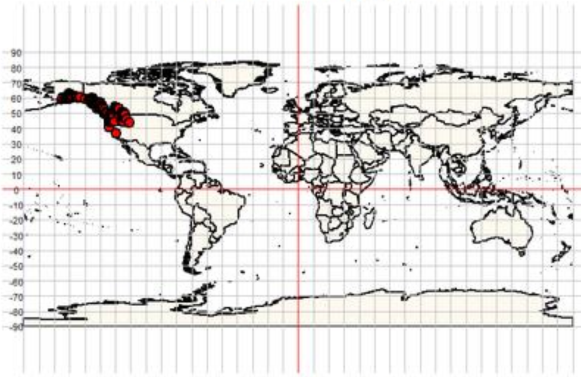

Micropholis cayennensis

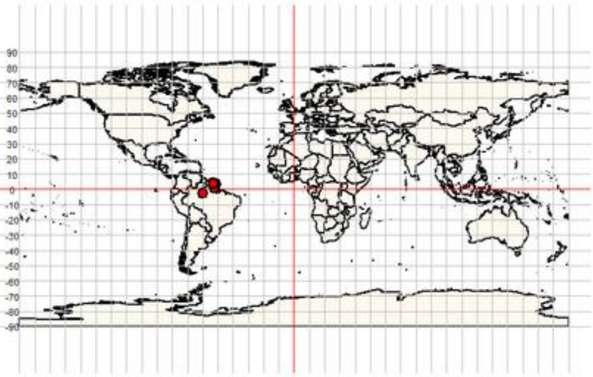

Micropholis crotonoides

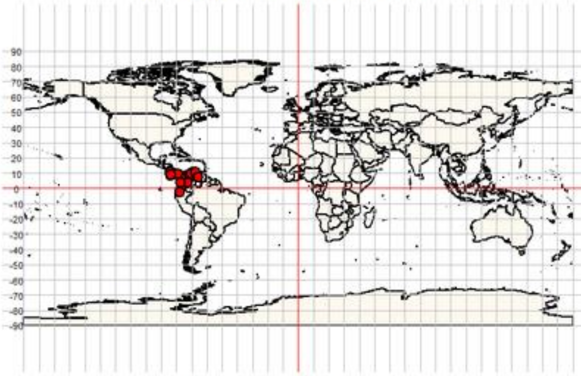

Mimusops elengi

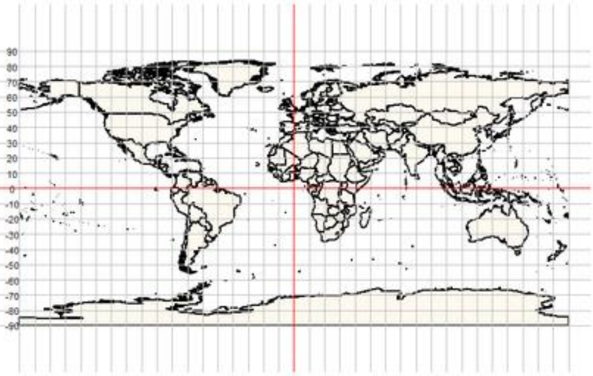

Mitrastemon matudae

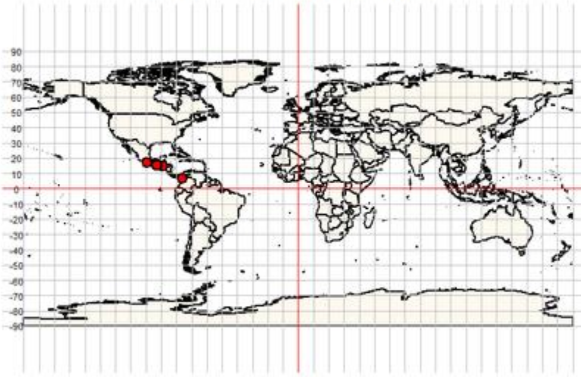

Mitrastemon yamamotoi

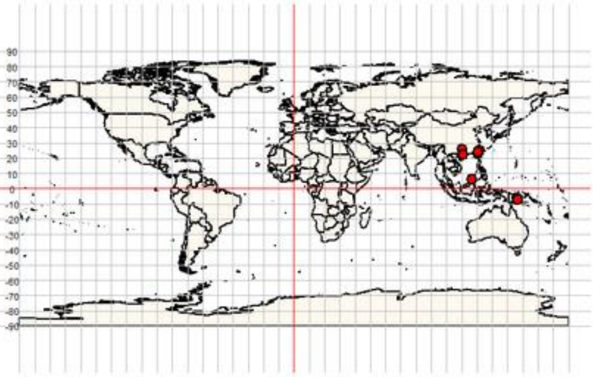

Monoporus spathulatus

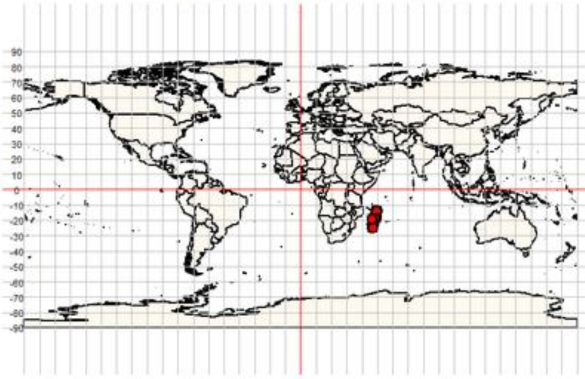

Myrsine laetevirens

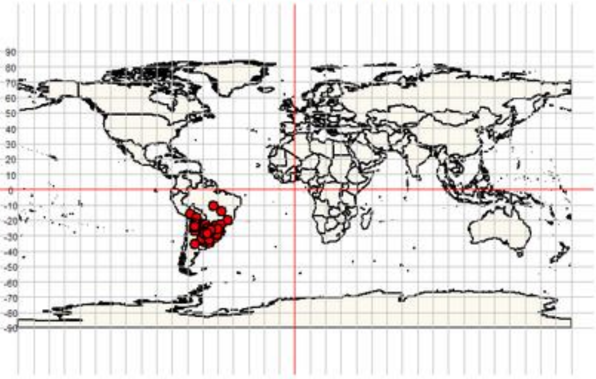

Napoleonaea vogelii

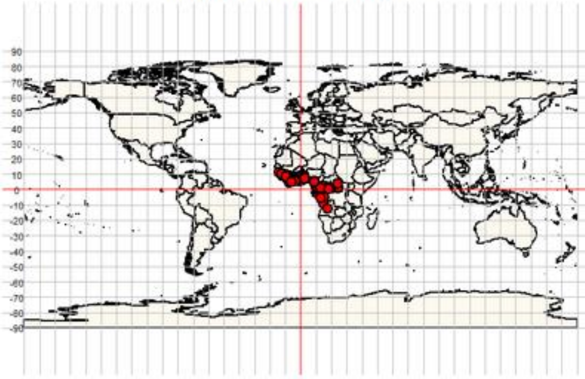

Neohemsleya usambarensis

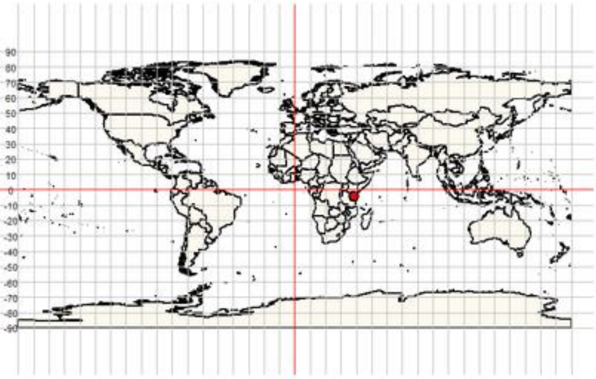

Neolemonniera ogouensis

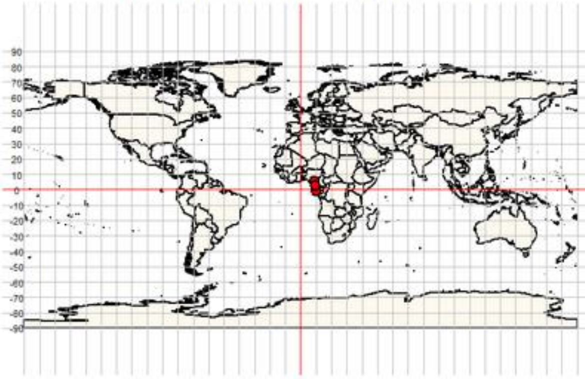

Neomezia cubensis

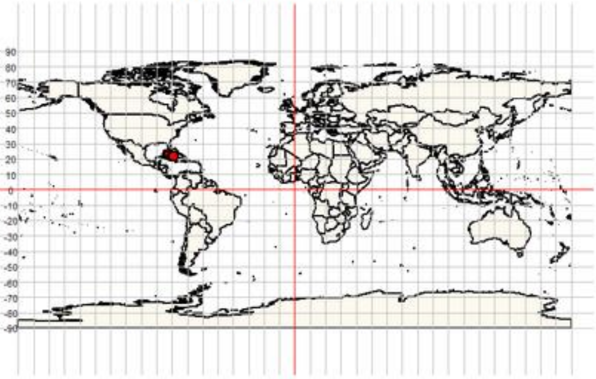

Niemeyera chartacea

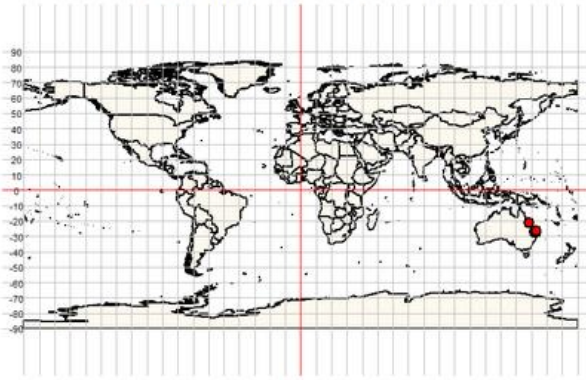

Norantea guianensis

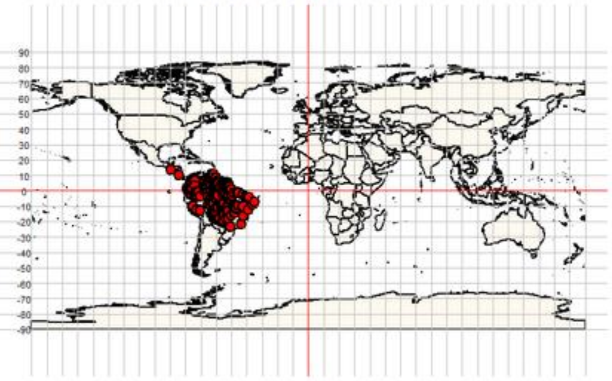

Northia seychellana

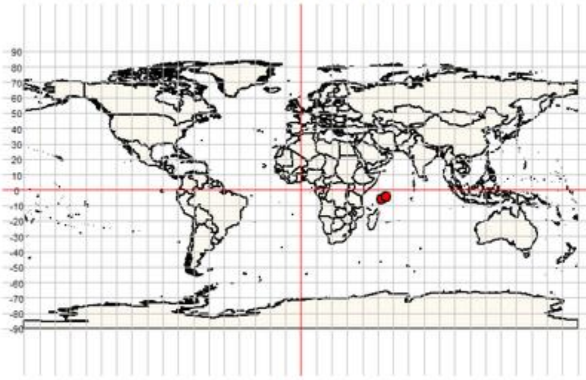

Omphalocarpum procerum

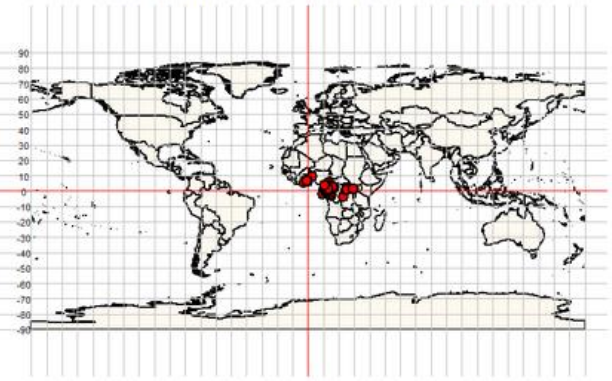

Omphalogramma vinciflorum

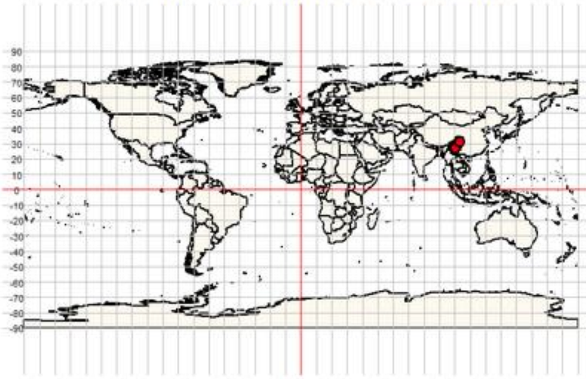

Oncostemum hieroglyforme

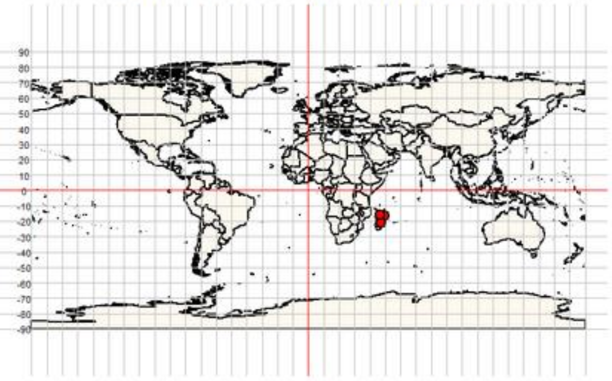

Orthilia secunda

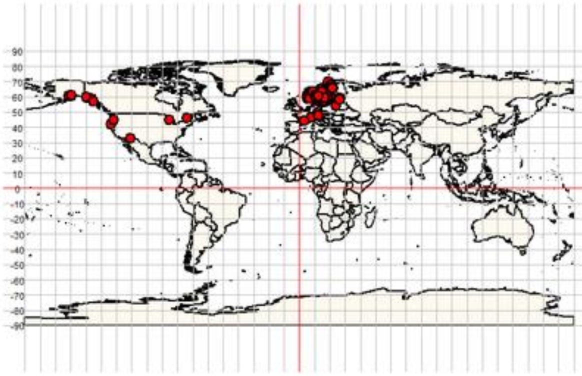

Oubanguia africana

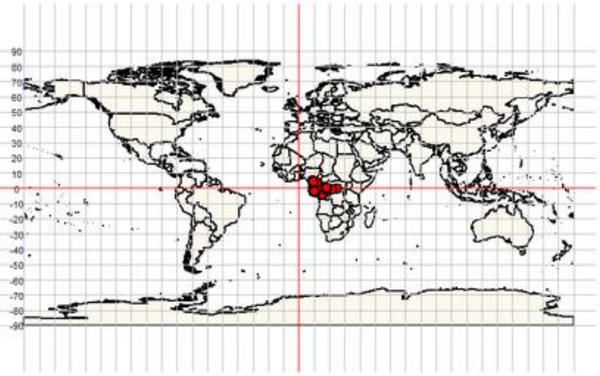

Oxydendrum arboreum

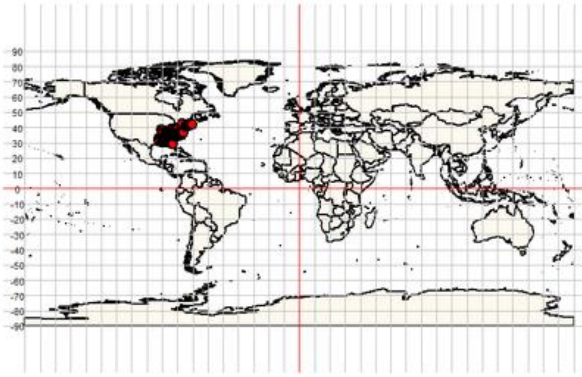

Palaquium beccarianum

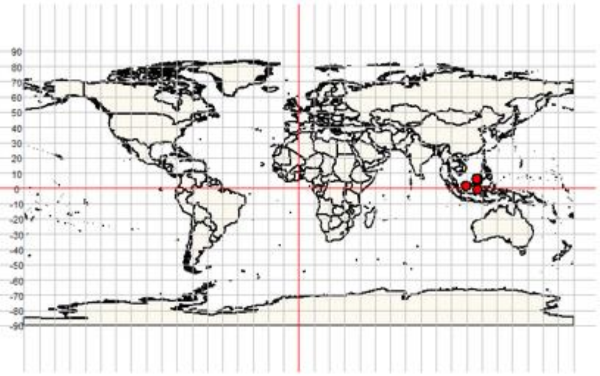

Palaquium formosanum

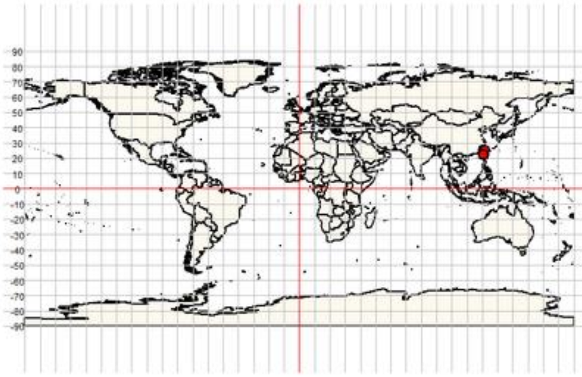

Palaquium obovatum

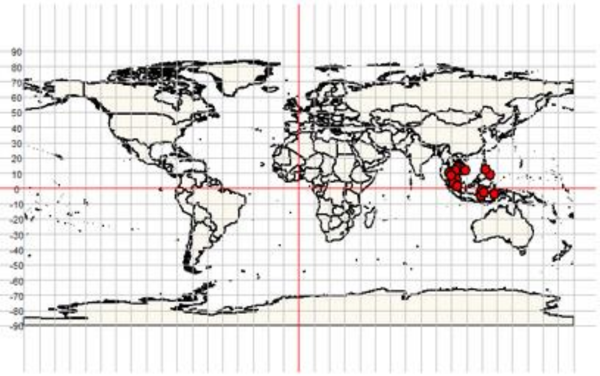

Pamphilia pedicellata

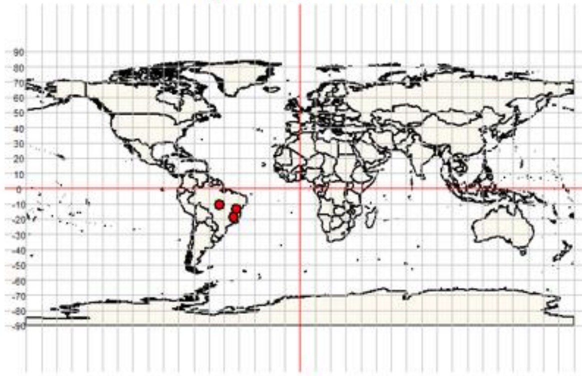

Parastyrax lacei

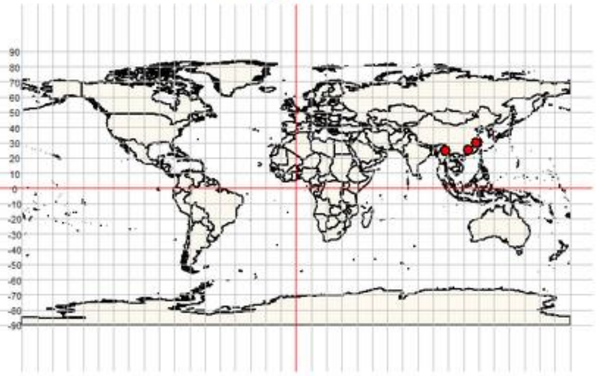

Parathesis glabra

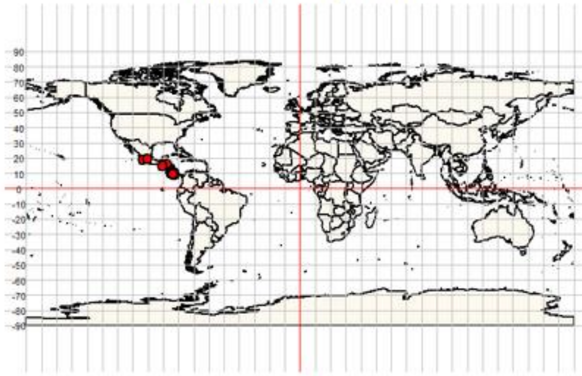

Payena lucida

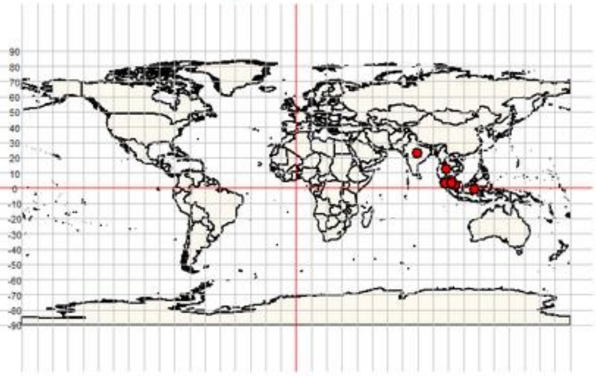

Pelletiera wildpretii

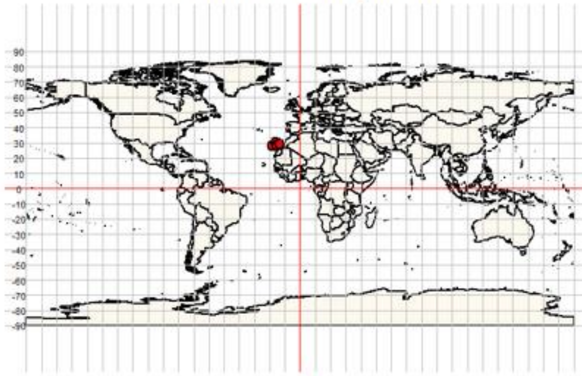

Pelliciera rhizophorae

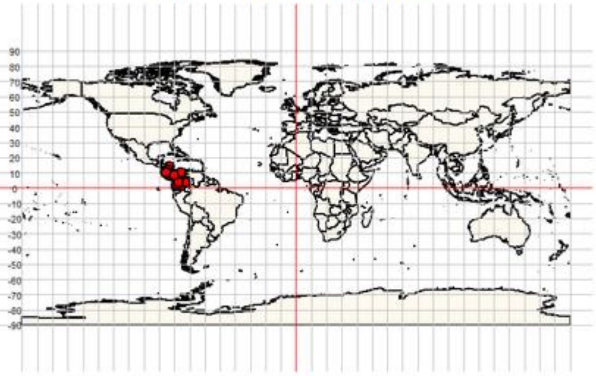

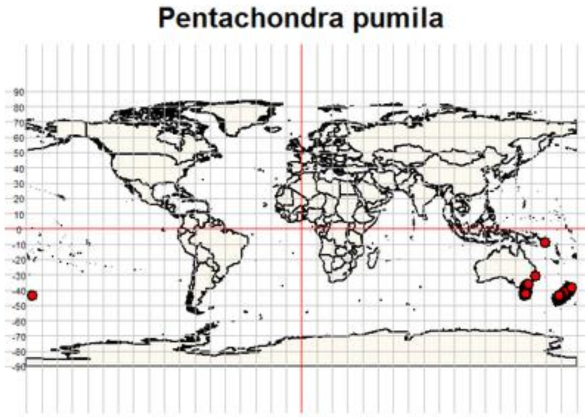
Pentachondra pumila

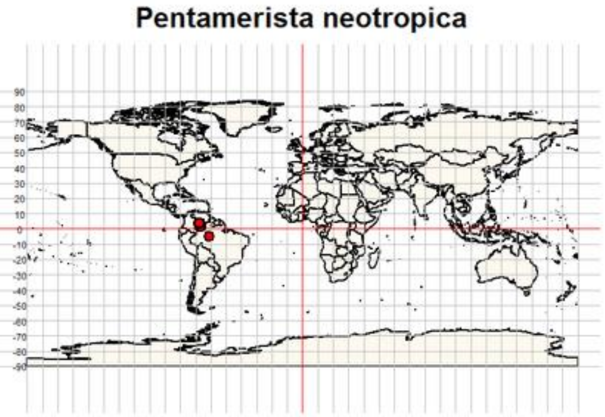
Pentamerista neotropica

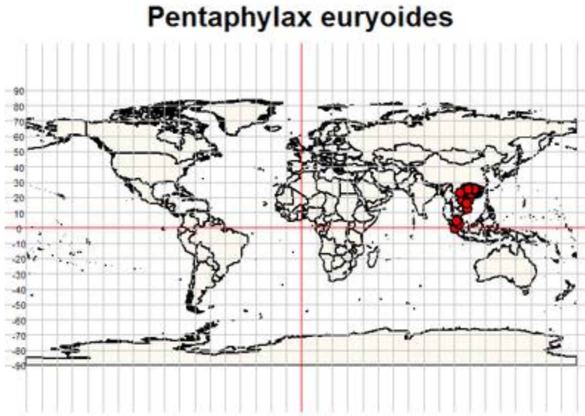
Pentaphylax euryoides

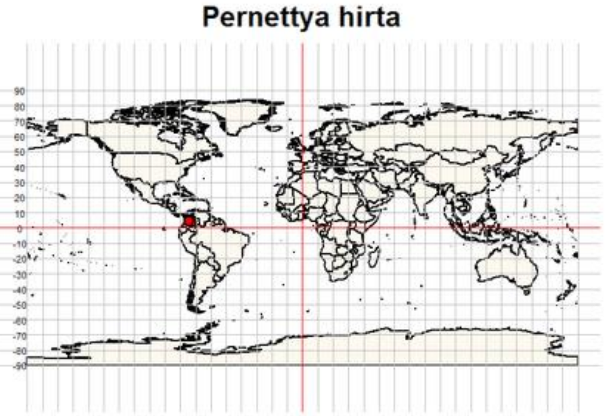
Pernettya hirta

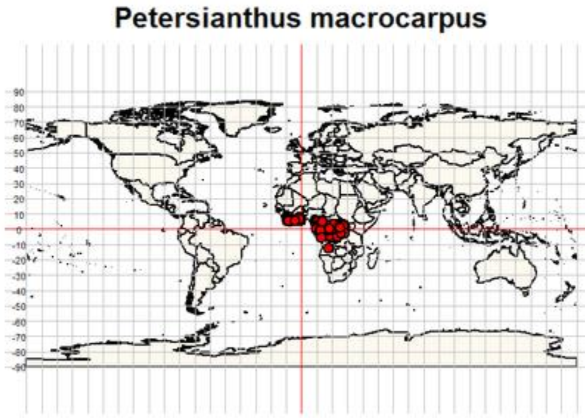
Petersianthus macrocarpus

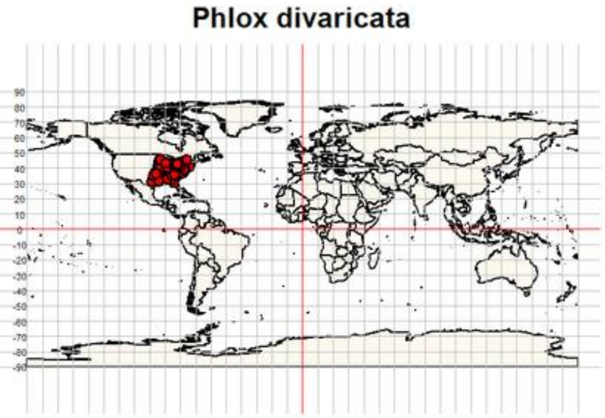
Phlox divaricata

Phyllodoce caerulea

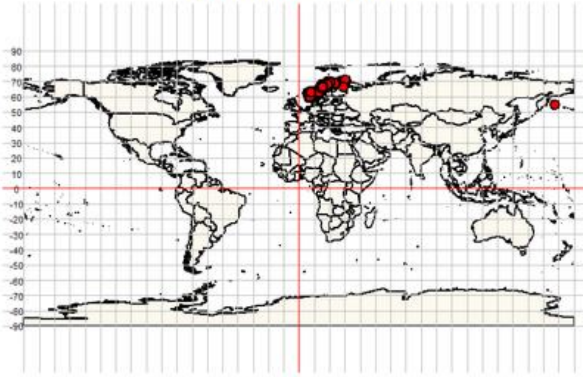

Pichonia balansana

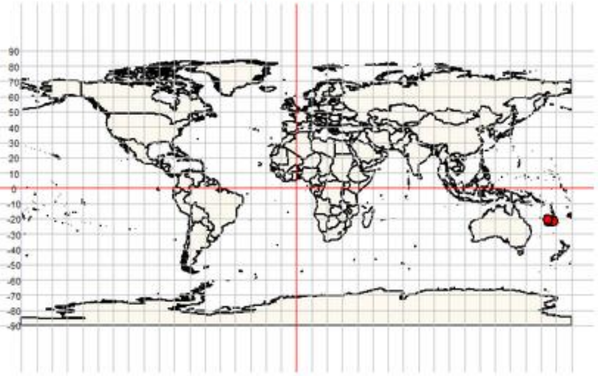

Pieris cubensis

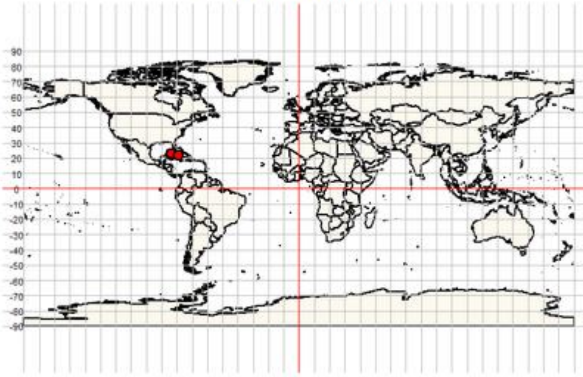

Pierrina zenkeri

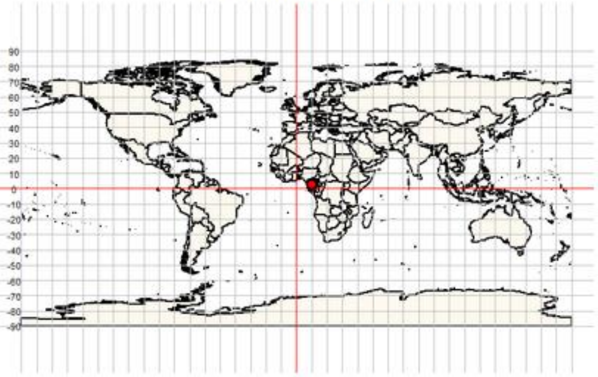

Planchonella kuebiniensis

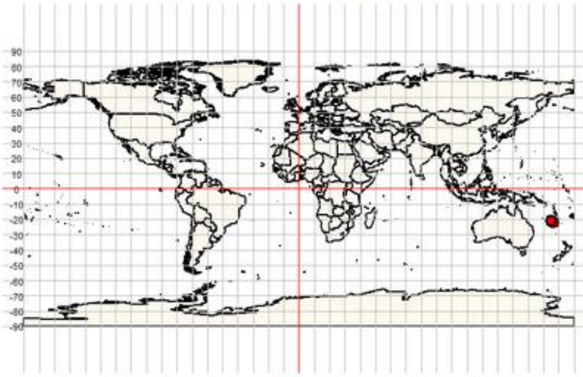

Planchonella obovata

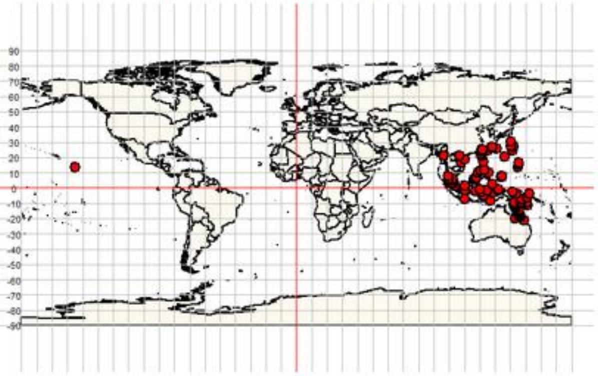

Planchonia careya

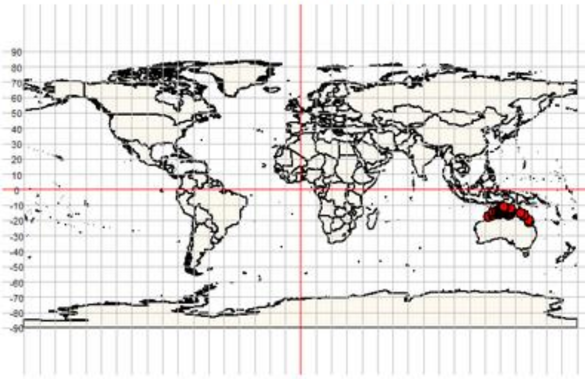

Pleiomeris canariensis

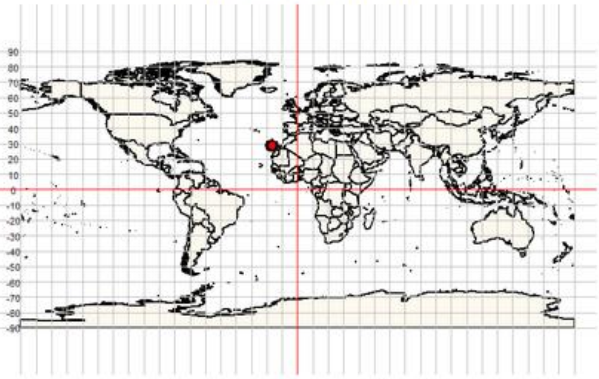

Polemonium caeruleum

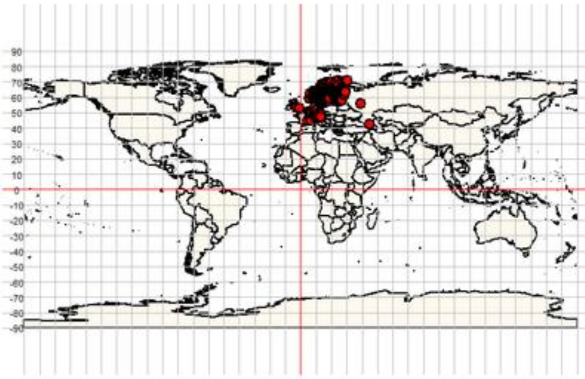

Polemonium reptans

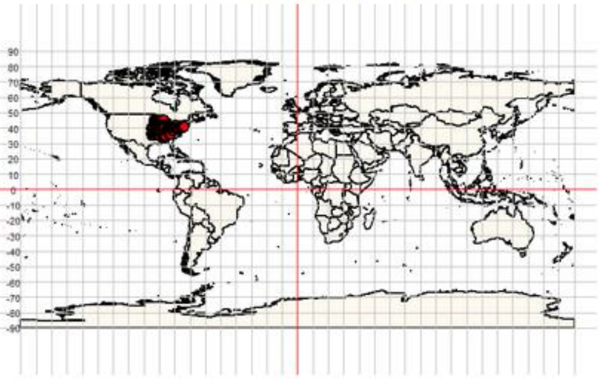

Polyspora axillaris

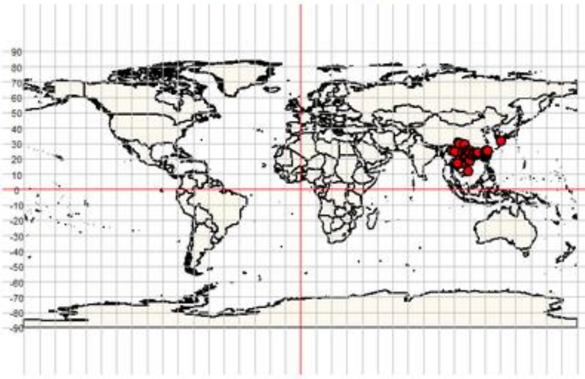

Pouteria bangii

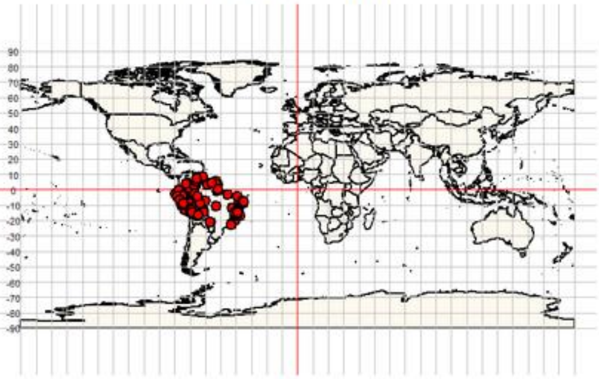

Pouteria campechiana

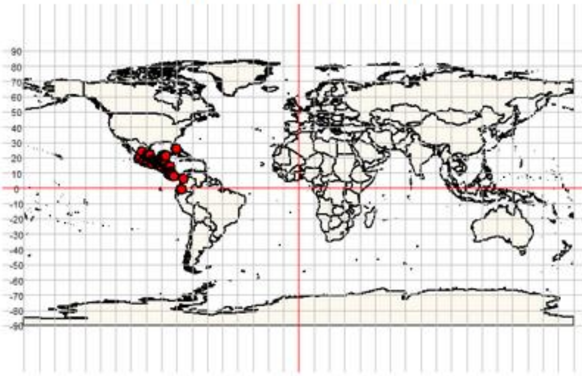

Pouteria foveolata

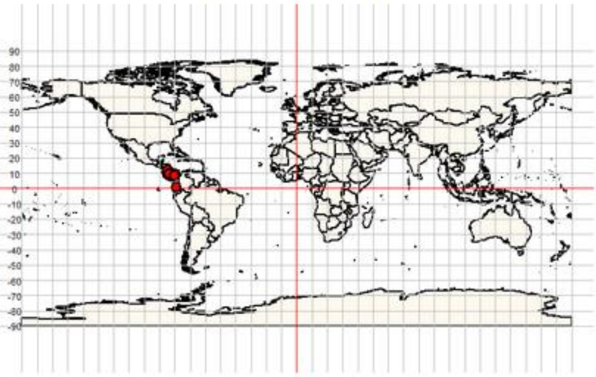

Pouteria ramiflora

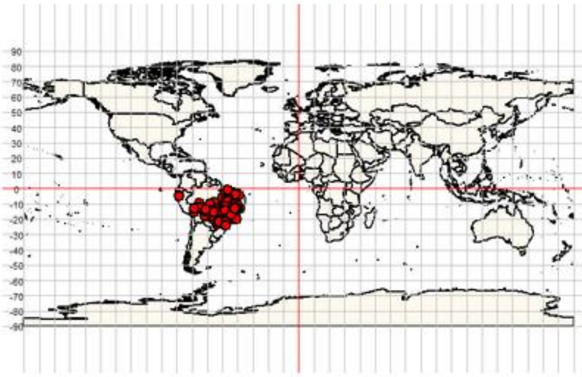

Pradosia colombiana

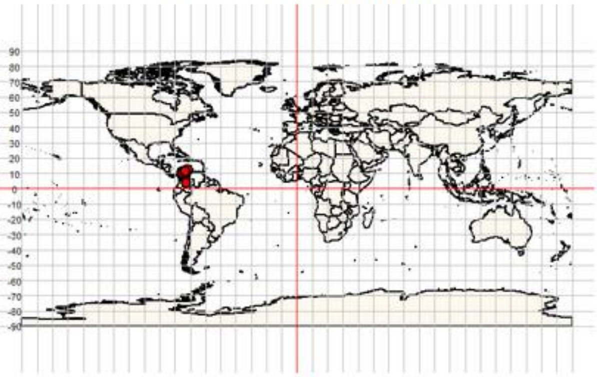

Primula veris

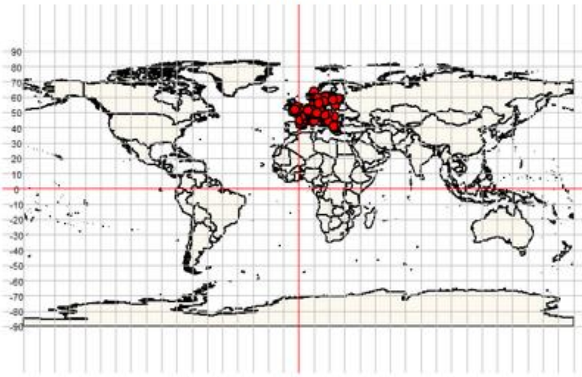

Prionotes cerinthoides

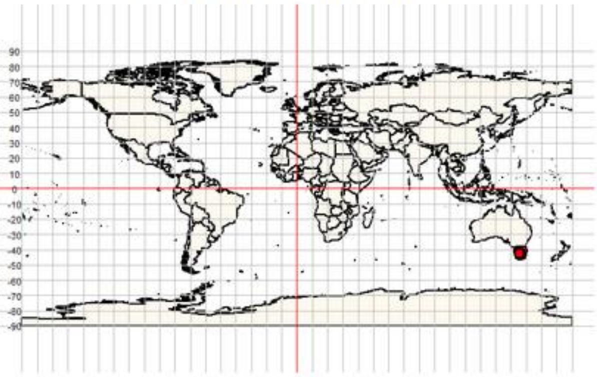

Pterostyrax corymbosus

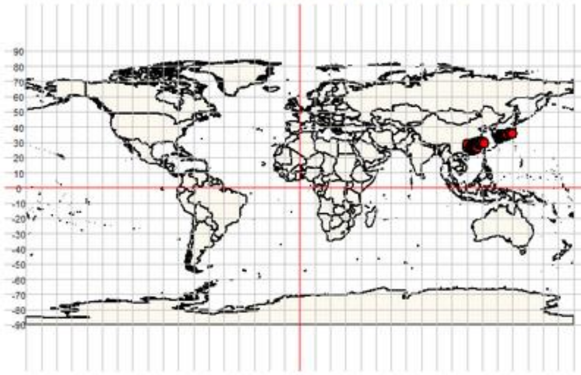

Pterostyrax hispidus

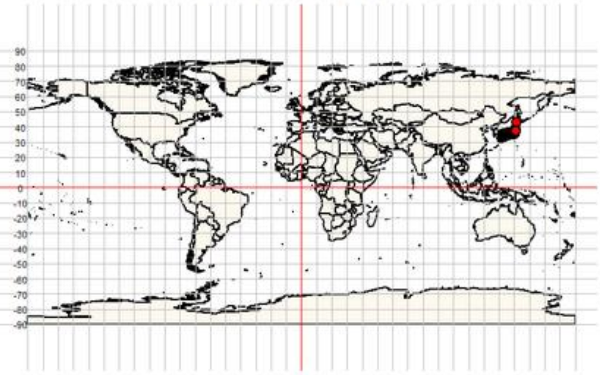

Purdiaea nutans

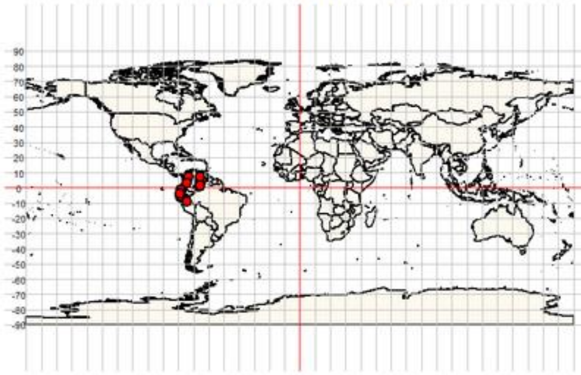

Purdiaea stenopetala

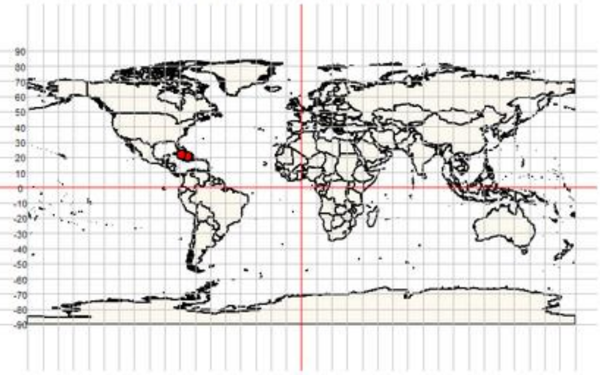

Pycnandra benthamii

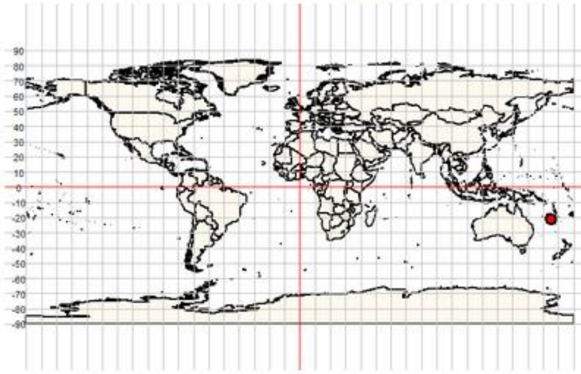

Pyrenaria spectabilis

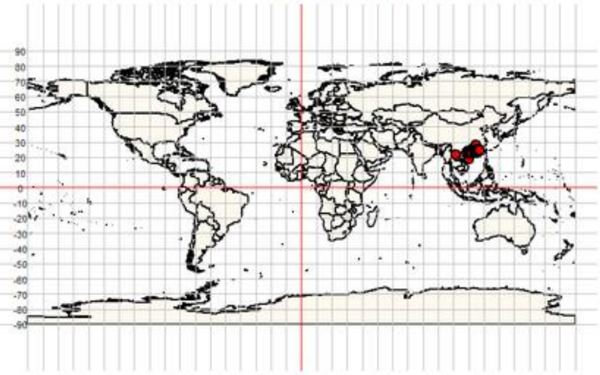

Pyrola rotundifolia

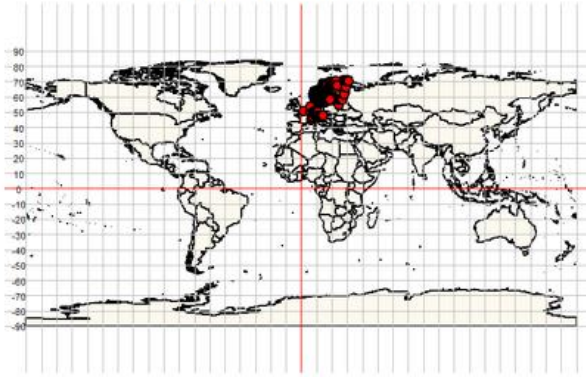

Pyxidanthera barbulata

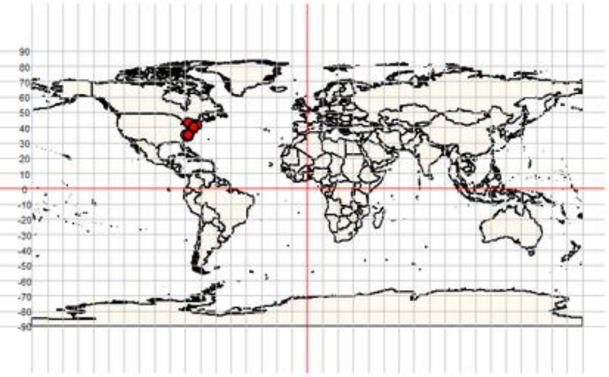

Rehderodendron macrocarpum

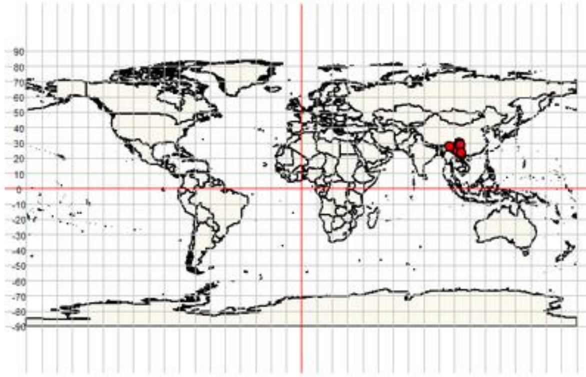

Rhaptopetalum geophylax

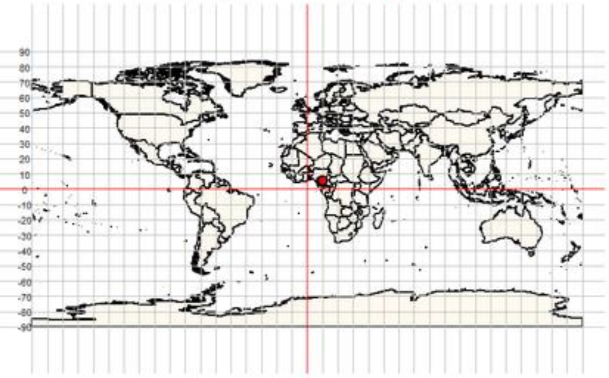

Rhododendron simsii

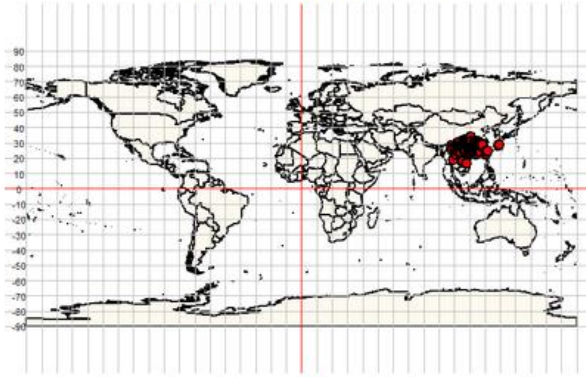

Roridula dentata

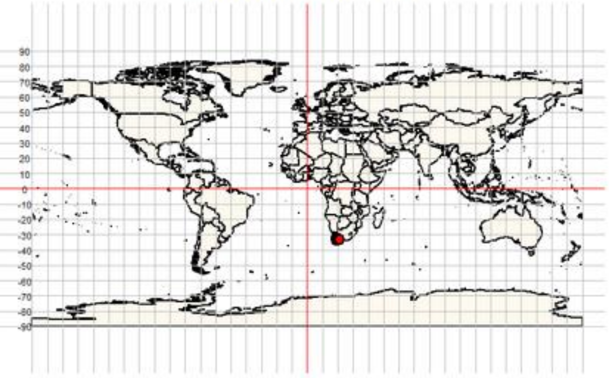

Roridula gorgonias

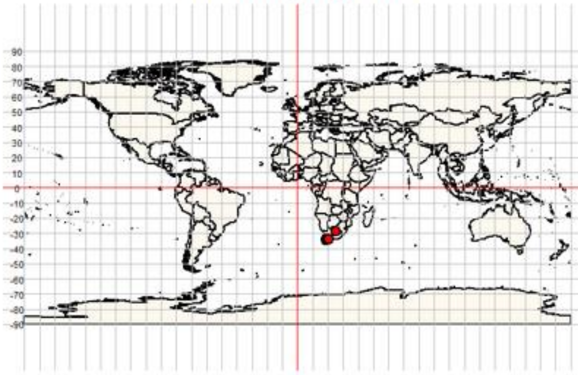

Ruyschia phylladenia

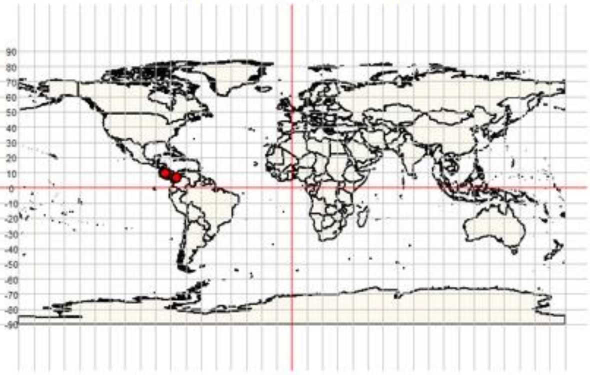

Samolus repens

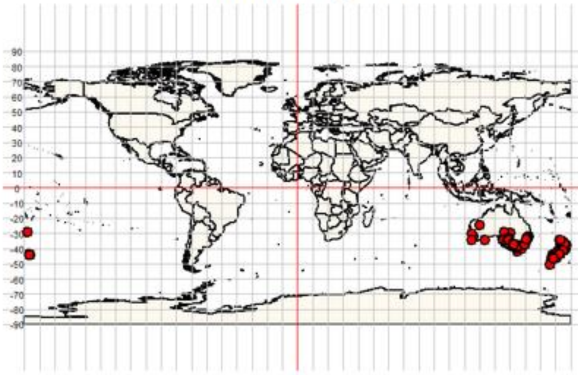

Samolus valerandi

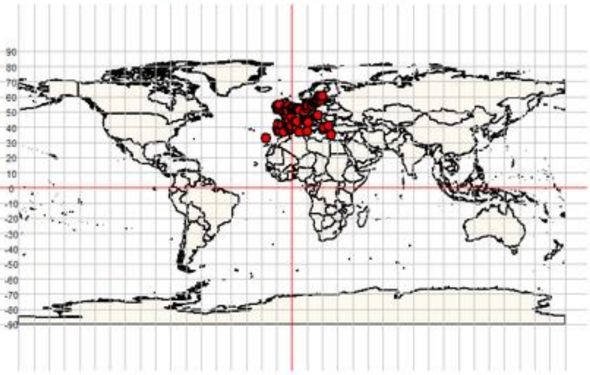

Sarcaulus brasiliensis

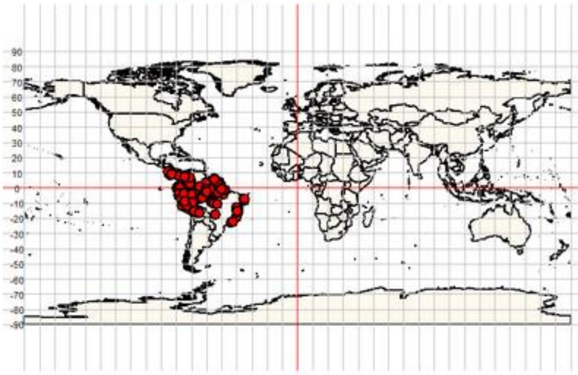

Sarcopera sessiliflora

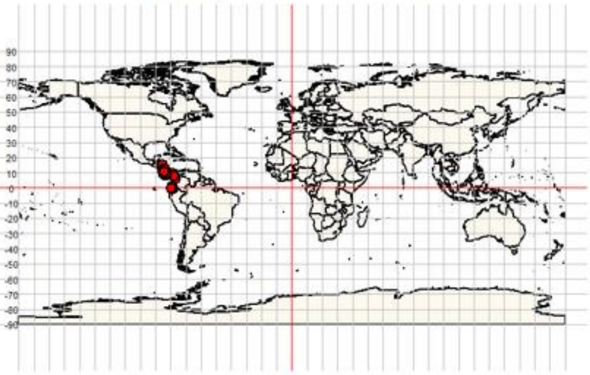

Sarcosperma laurinum

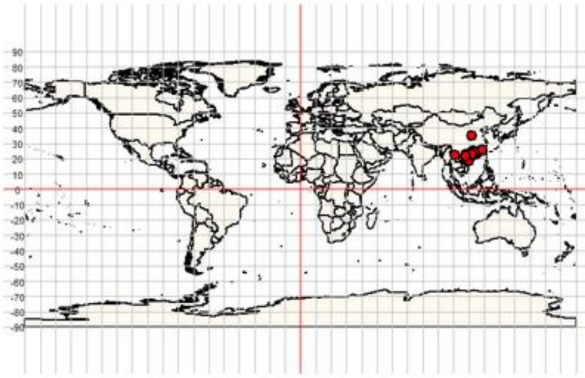

Sarracenia flava

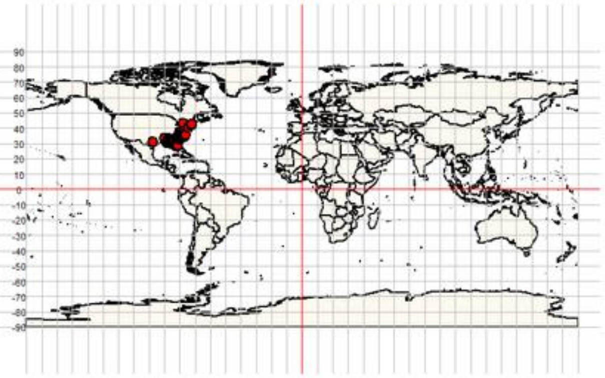

Sarracenia minor

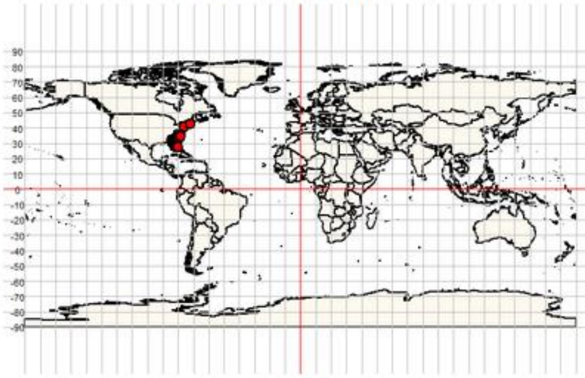

Sarracenia purpurea

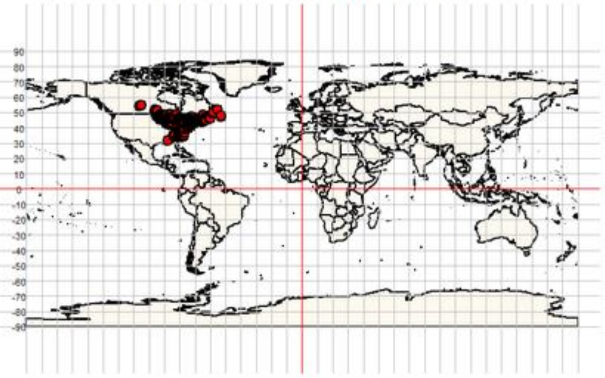

Sarracenia rosea

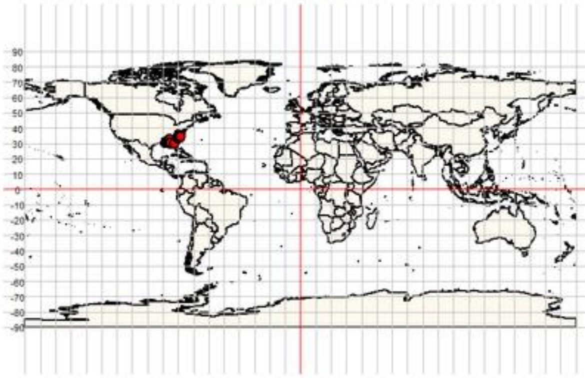

Sarracenia rubra

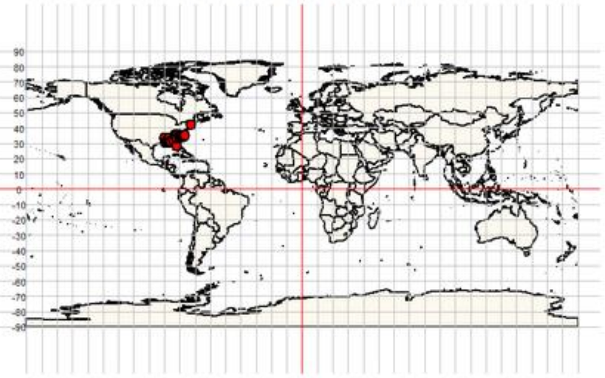

Satyria grandifolia

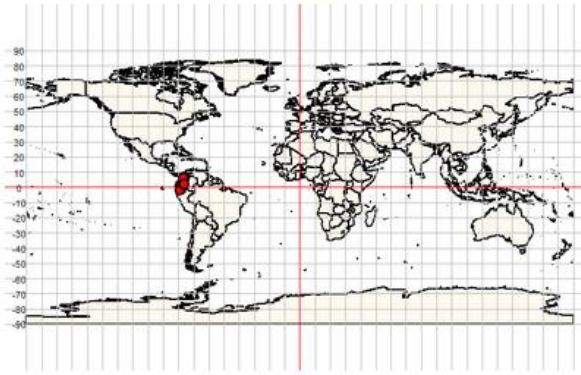

Saurauia excelsa

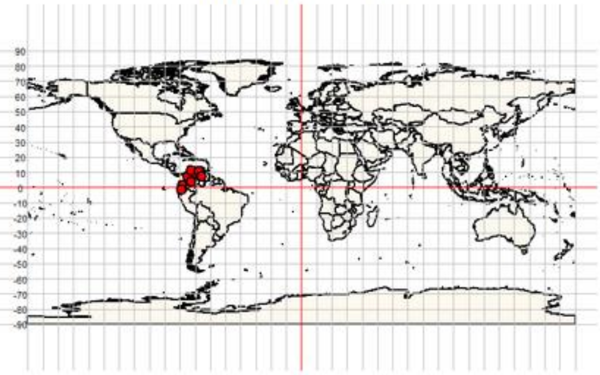

Saurauia kegeliana

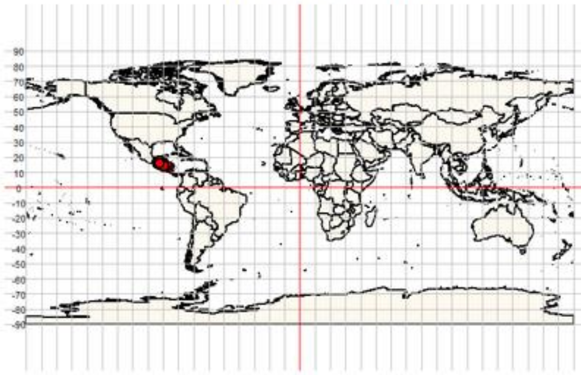

Saurauia montana

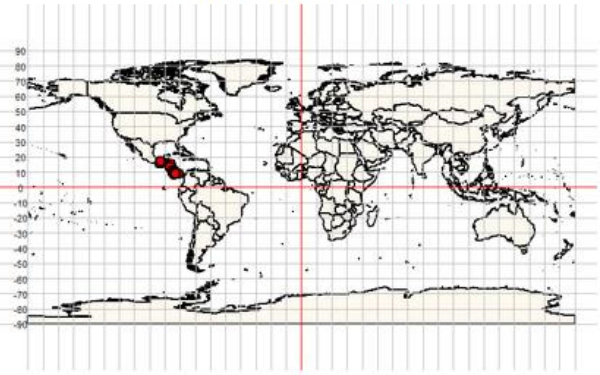

Saurauia napaulensis

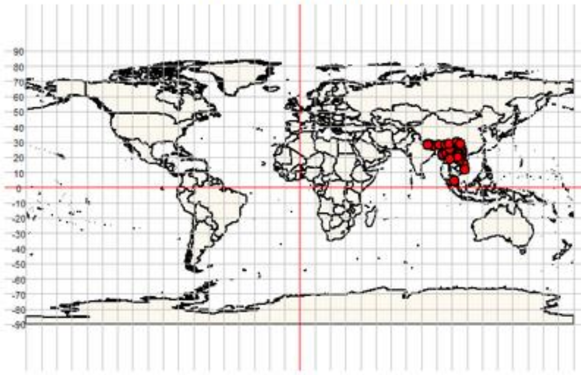

Saurauia subspinosa

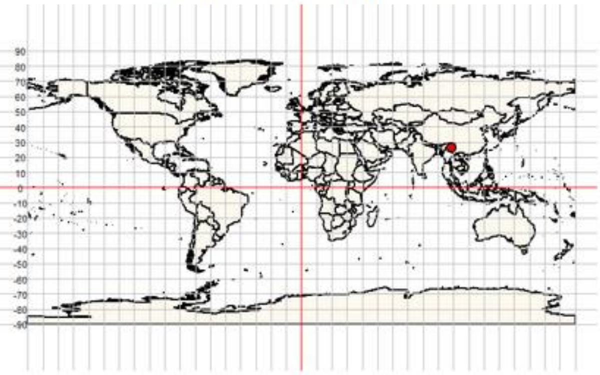

Schima superba

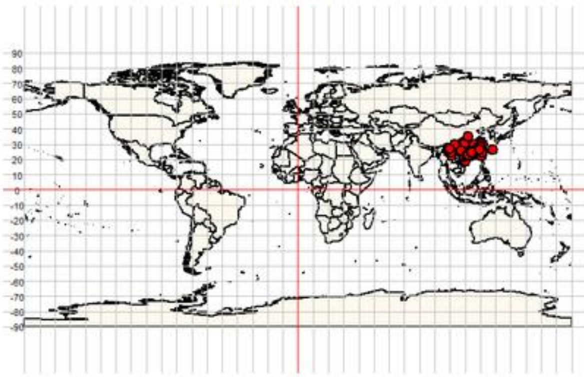

Schizocodon soldanelloides

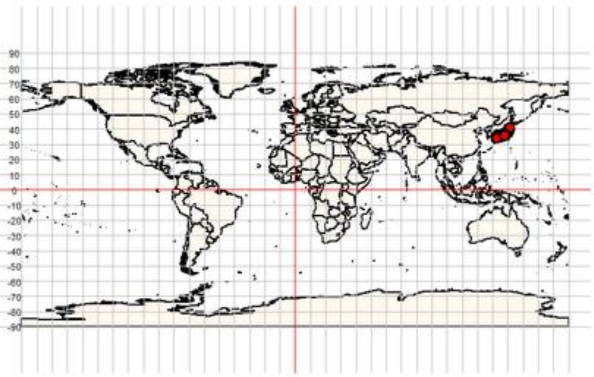

Schwartzia brasiliensis

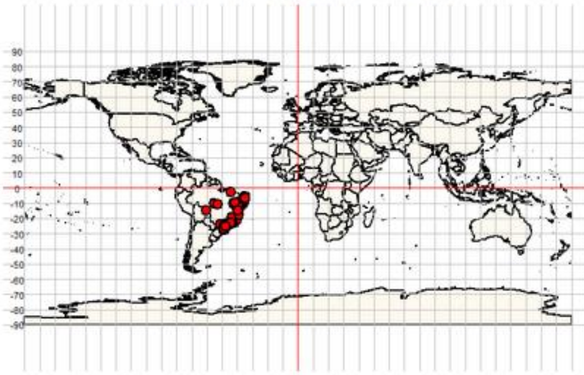

Scytopetalum klaineanum

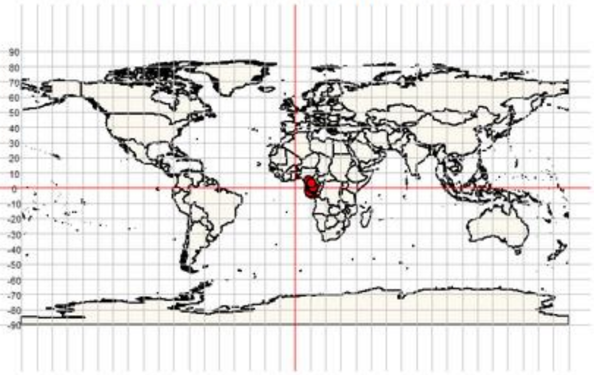

Shortia galacifolia

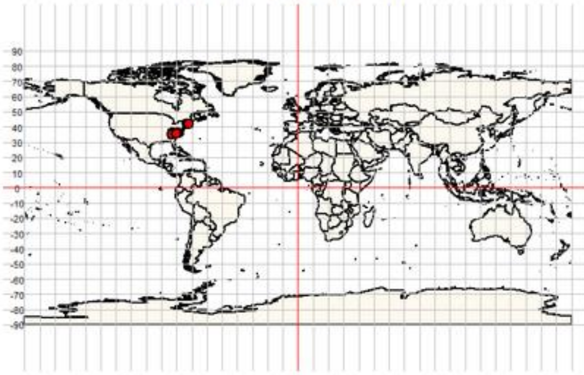

Shortia rotundifolia

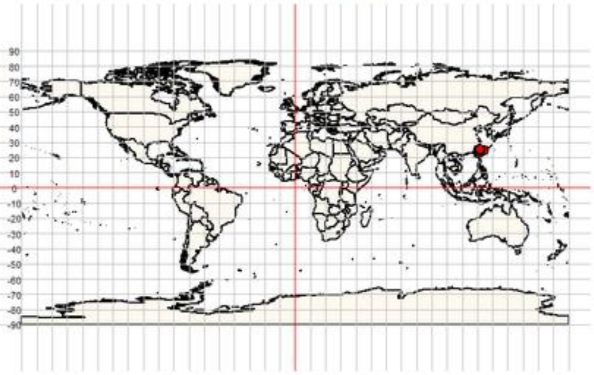

Shortia uniflora

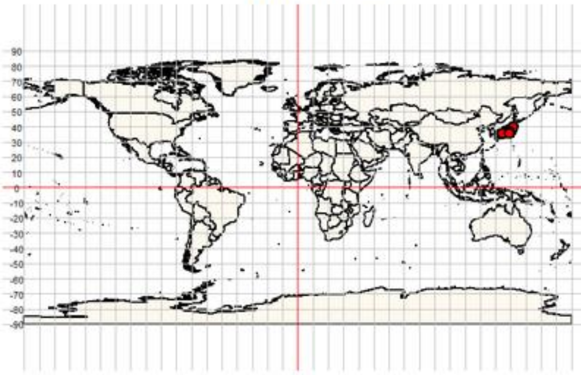

Sideroxylon horridum

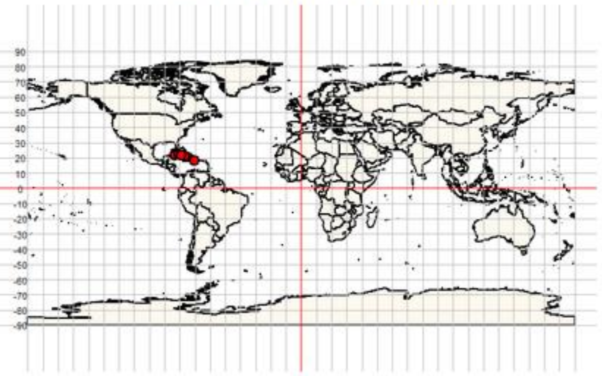

Sinojackia xylocarpa

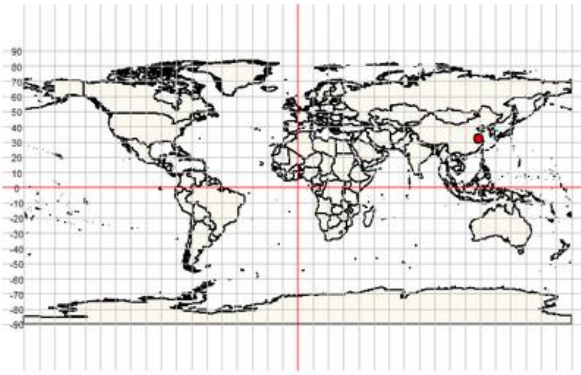

Sladenia celastrifolia

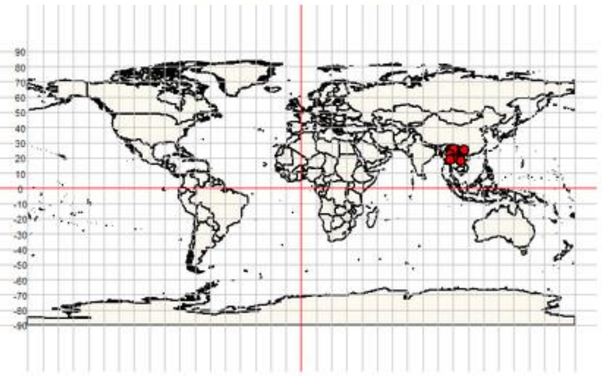

Soldanella alpina

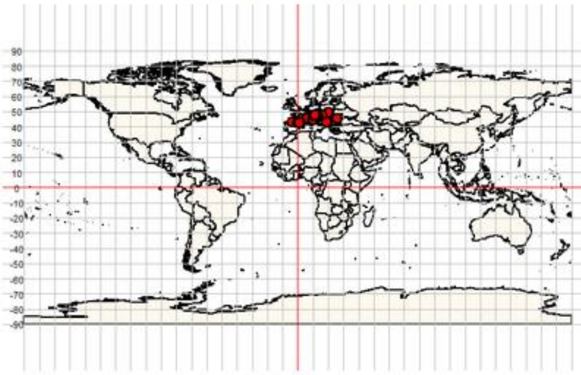

Souroubea vallicola

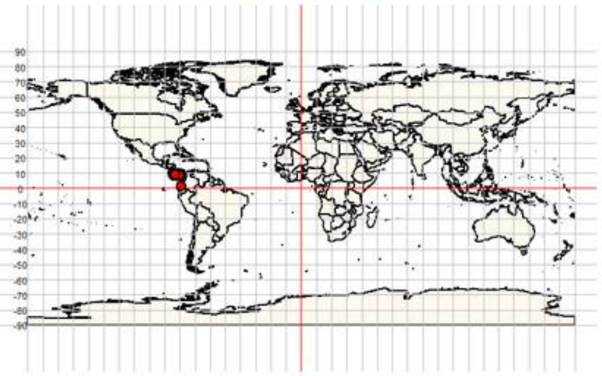

Stewartia pseudocamellia

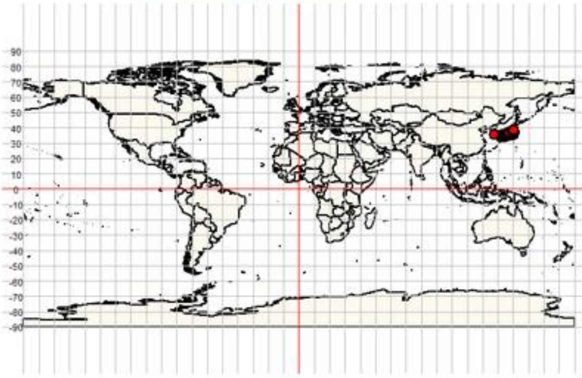

Stimpsonia chamaedryoides

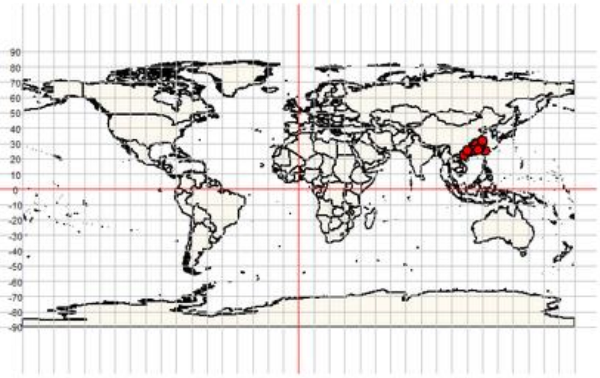

Styrax americanus

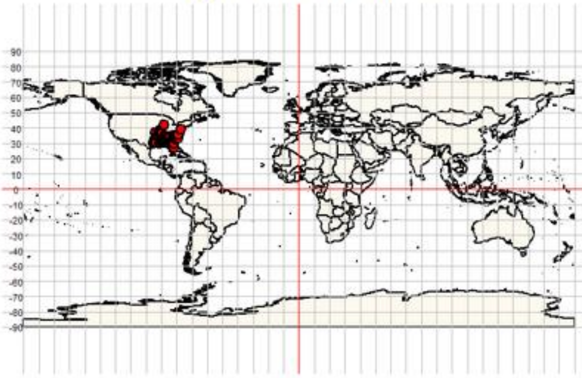

Styrax chinensis

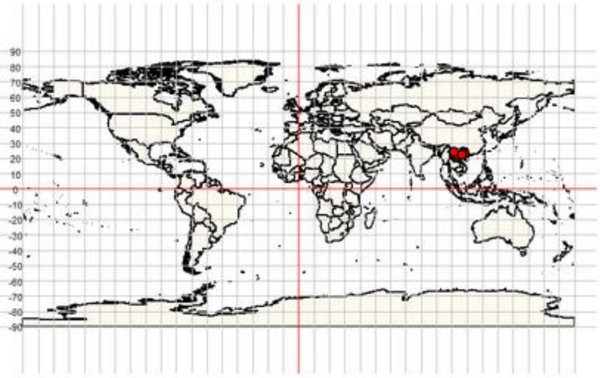

Styrax japonicus

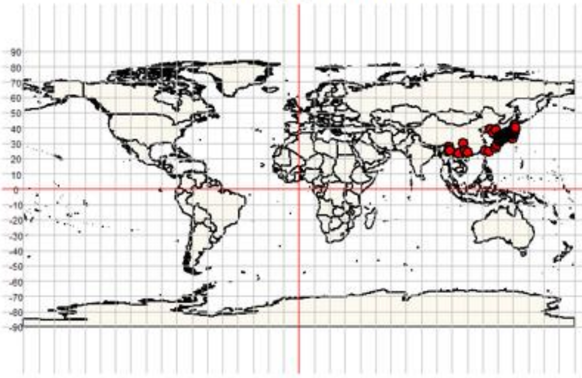

Styrax obassia

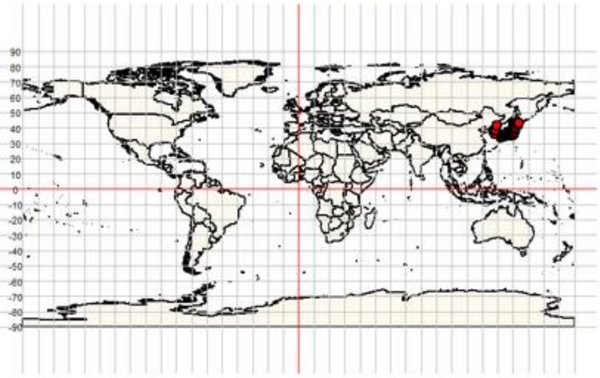

Styrax officinalis

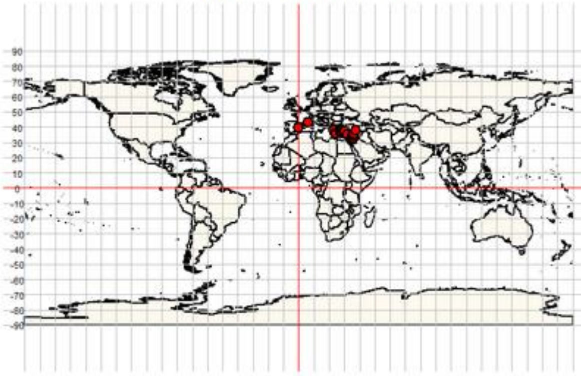

Styrax redivivus

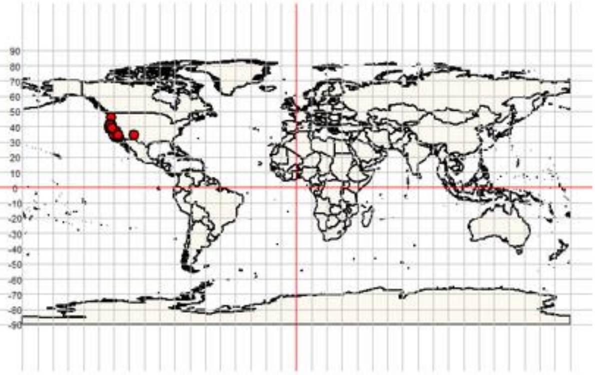

Symplococarpon purpusii

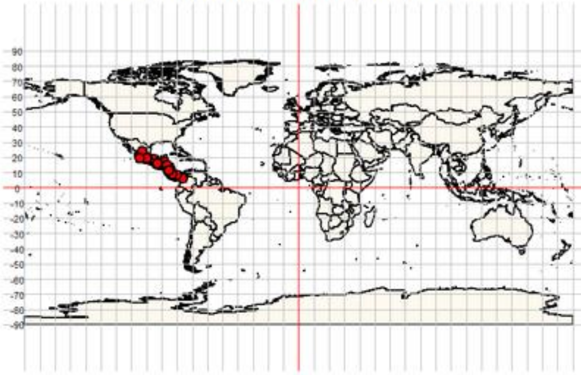

Symplocos anomala

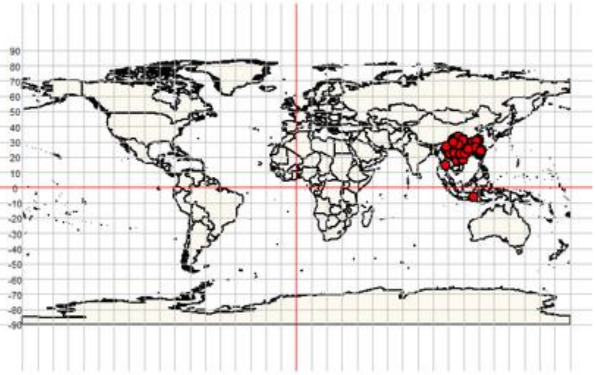

Symplocos austromexicana

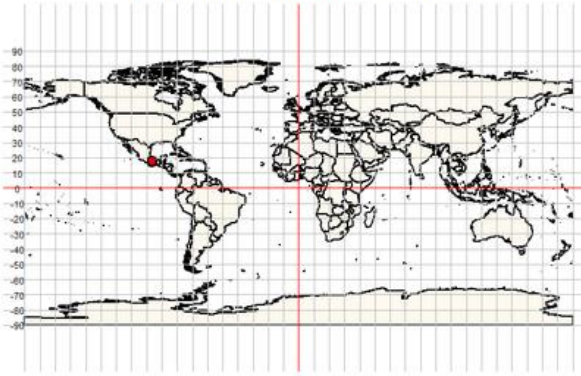

Symplocos kleinii

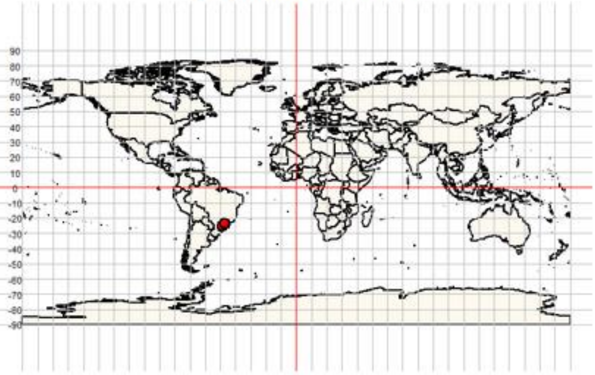

Symplocos lanata

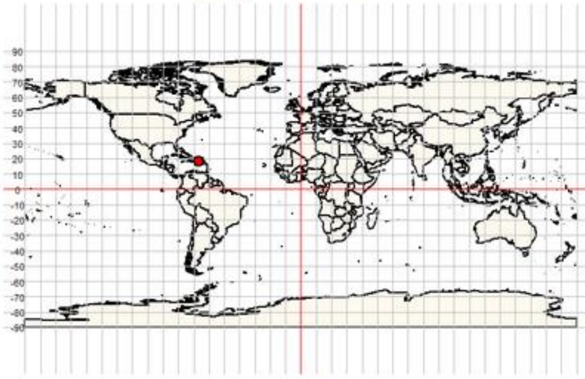

Symplocos martinicensis

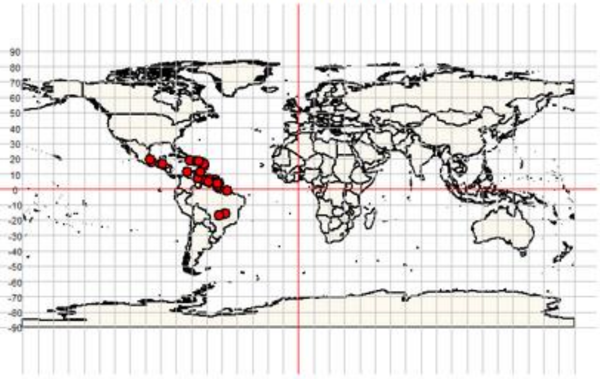

Symplocos paniculata

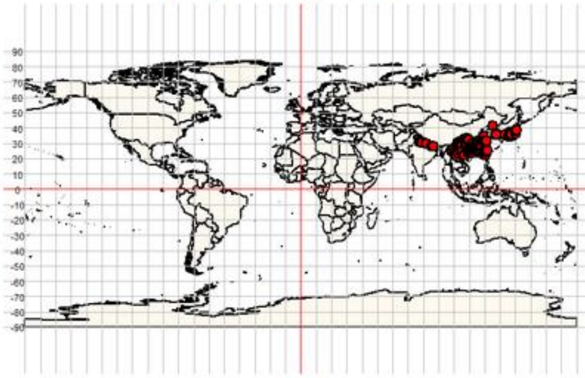

Symplocos tinctoria

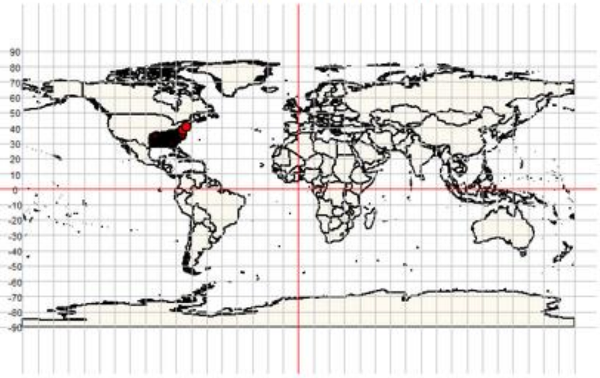

Symplocos zizyphoides

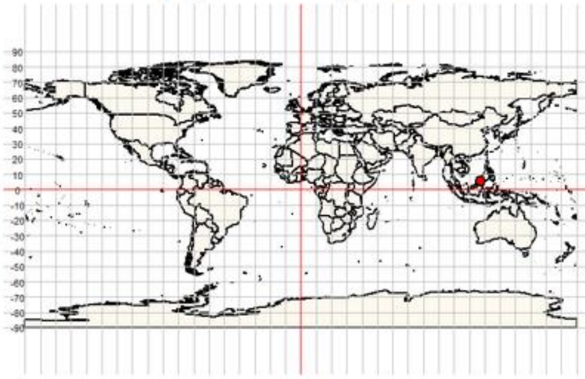

Synsepalum dulcificum

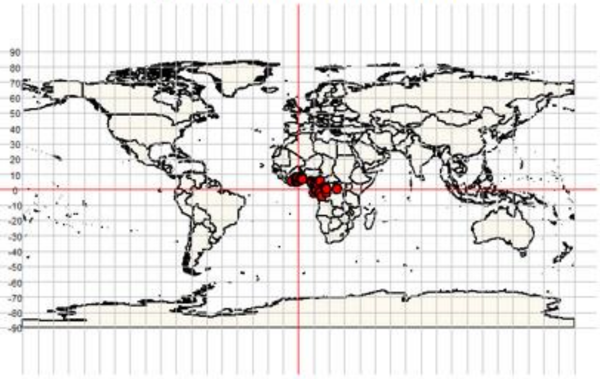

Taonabo stuebelii

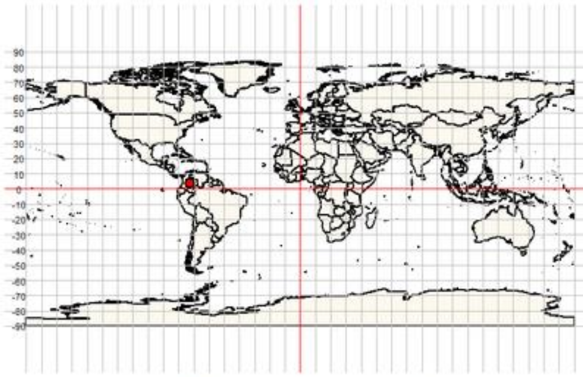

Tepuia venusta

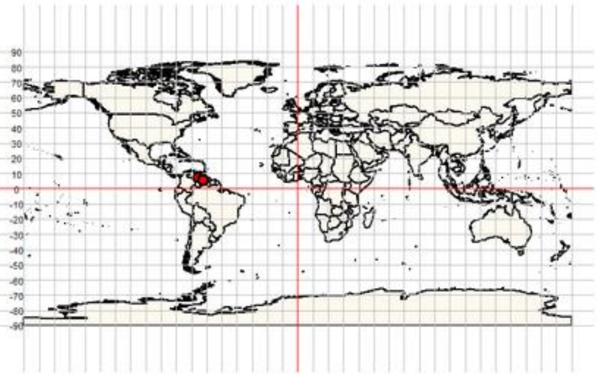

Ternstroemia gymnanthera

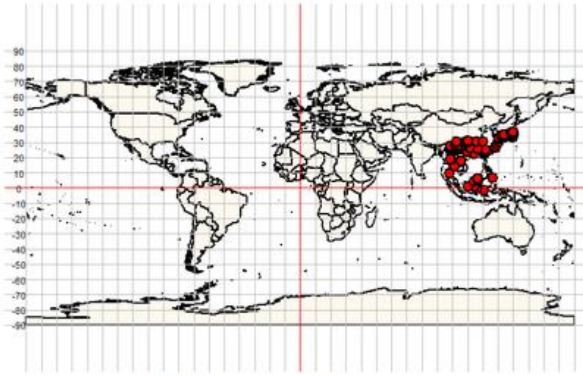

Ternstroemia stahlii

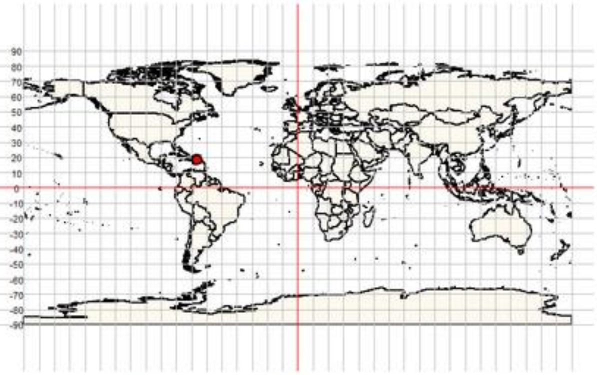

Tetramerista glabra

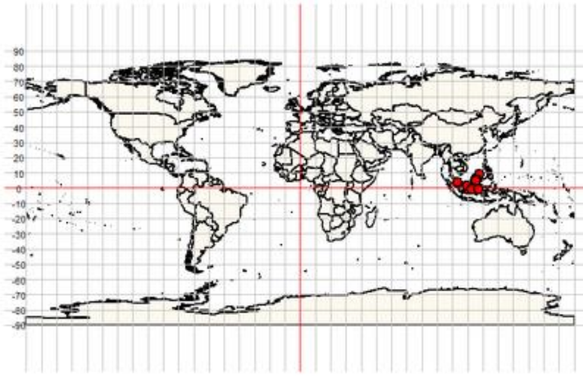

Theophrasta americana

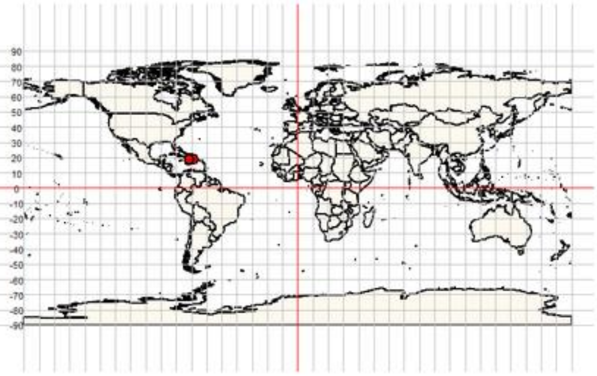

Therorhodion camtschaticum

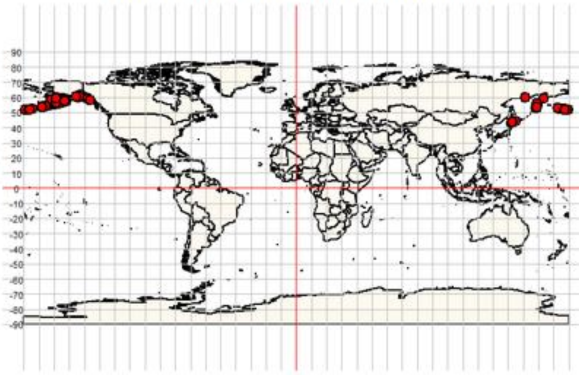

Tieghemella africana

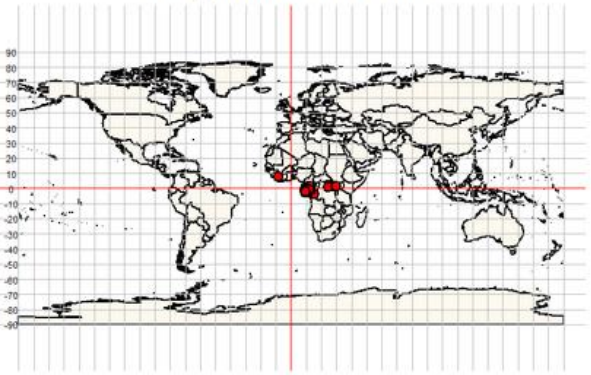

Tridesmostemon omphalocarpoides

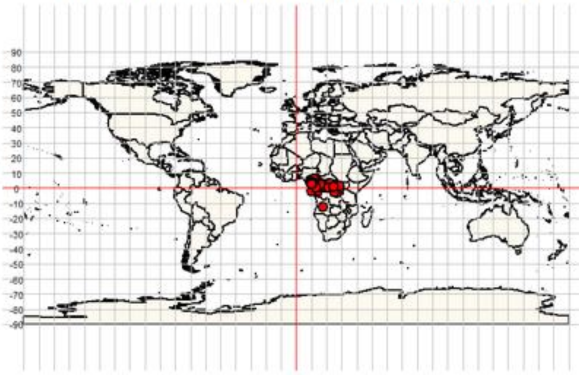

Tsebona macrantha

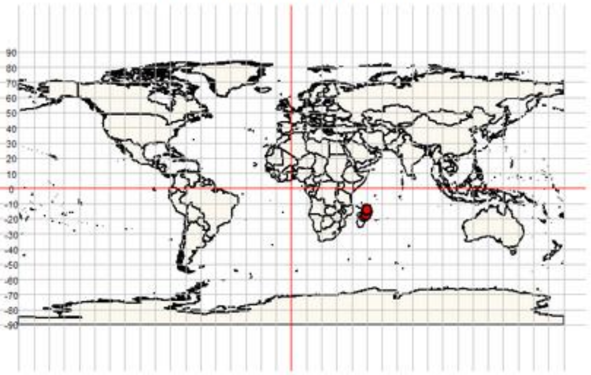

Vaccinium myrtillus

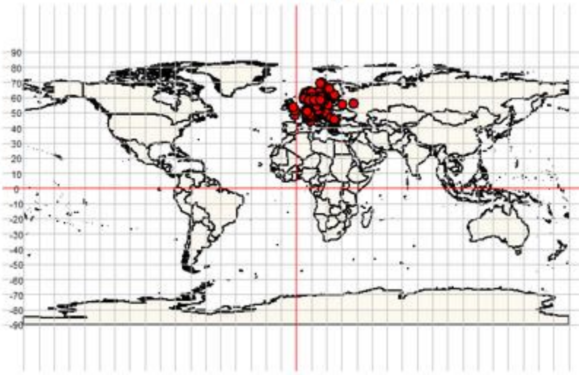

Visnea mocanera

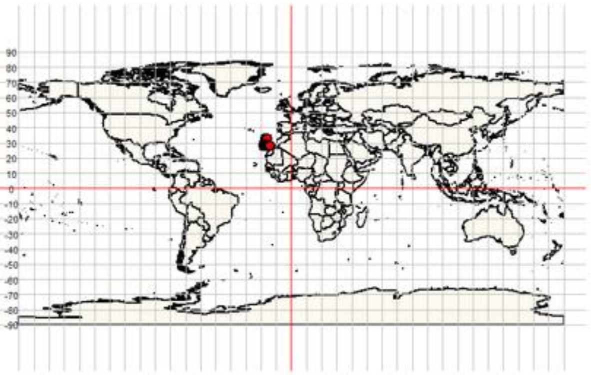

Vitaliana primuliflora

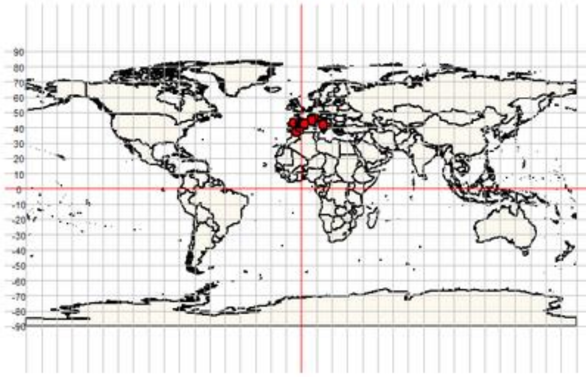

Vitellaria paradoxa

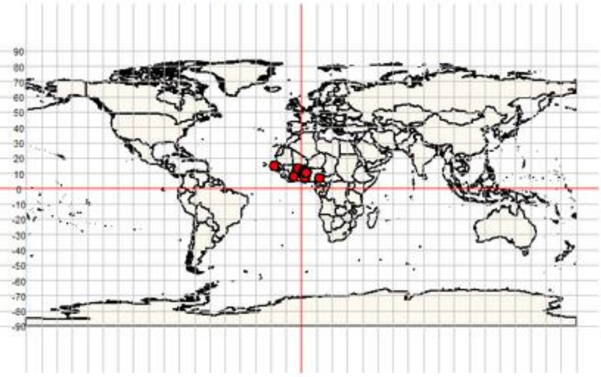

Vitellariopsis marginata

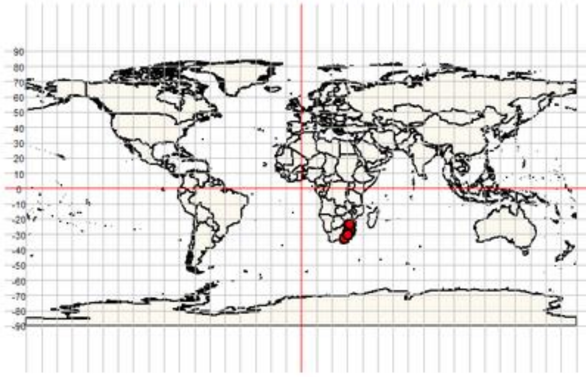

Votschia nemophila

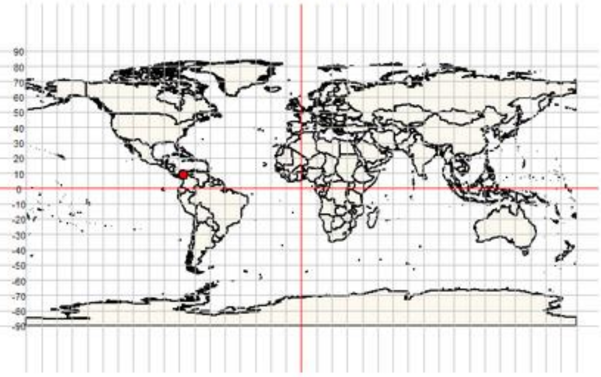

Xantolis cambodiana

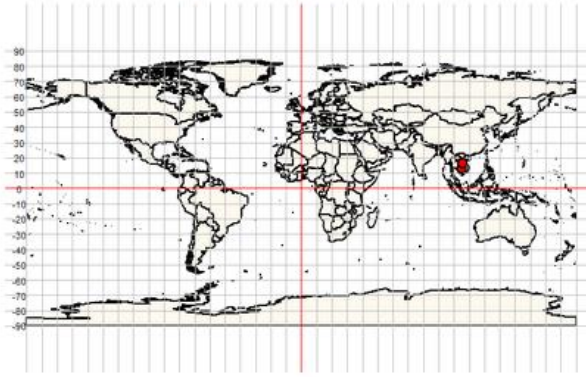

Zenobia pulverulenta

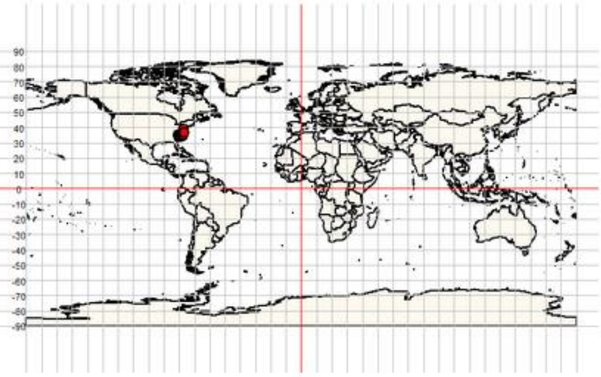

4. References

Bivand R, Lewin-Koh N. 2017. *maptools: Tools for Reading and Handling Spatial Objects.* R package version 0.9-2. https://CRAN.R-project.org/package=maptools

Chamberlain S. 2017. *rgbif: Interface to the Global 'Biodiversity' Information Facility API.* R package version 0.9.9. https://CRAN.R-project.org/package=rgbif

Chartier M, Jabbour F, Gerber S, Mitteroecker P, Sauquet H, von Balthazar M, Staedler Y, Crane PR, Schönenberger J. 2014. The floral morphospace - a modern comparative approach to study angiosperm evolution. *New Phytol.* **204**, 841–853.

Cox B, 2001. The biogeographic regions reconsidered. J. Biogeo. **28**, 511-523.

Hawkins D. 2014. *Biomeasurement: a student's guide to biological statistics*. Oxford: Oxford University Press.

Kubitzki K. 2004. *The families and genera of vascular plants. Volume 6. Flowering plants, Dicotyledons, Celastrales, Oxalidales, Rosales, Cornales, Ericales.* Berlin: Springer.

Loarie SR, Duffy PB, Hamilton H, Asner GP, Field CB, Ackerly DD. 2009. The velocity of climate change. *Nature* **462,** 1052-1055.

Oksanen JF, Blanchet G, Friendly M, Kindt R, Legendre P, McGlinn D, Minchin PR, O'Hara RB, Simpson GL, Solymos PM, Stevens HH, Szoecs E, Wagner H. 2017. *vegan: Community; Ecology Package. R package version 2.4-3.* https://CRAN.R-project.org/package=vegan

Peel, MC, Finlayson BL, Mcmahon TA. 2007. Updated world map of the Köppen-Geiger climate classification. *Hydrol. Earth Syst. Sci.* **4,** 439-473.

R Core Team. 2018. *R: A language and environment for statistical computing*. R Foundation for Statistical Computing, Vienna, Austria. (https://www.R-project.org/)

Rose JP, Kleist TJ, Löfstrand SD, Drew BT, Schönenberger J, Sytsma KJ. 2018. Phylogeny, historical biogeography, and diversification of angiosperm order Ericales suggest ancient Neotropical and East Asian connections. *Mol. Phylogenet. Evol.* **122**, 59-79.

Sauquet H. 2019. PROTEUS: *A database for recording morphological data and fossil calibrations. Version 1.27.* http://eflower.myspecies.info/proteus

Schönenberger J, Anderberg AA, Sytsma KJ, 2005. Molecular phylogenetics and patterns of floral evolution in the Ericales. *Int. J. Plant Sci.*, **166**, 265-288.
